# A Computationally Constructed lncRNA-Associated Competing Triplet Network in Clear Cell Renal Cell Carcinoma

**DOI:** 10.1155/2022/8928282

**Published:** 2022-11-17

**Authors:** Hui Zhang, Qing Ye, Zixiang Chen, Chunyi Zhao, Qian Wu, Yuting Ding, Qixiang Shao, Yangjing Zhao

**Affiliations:** ^1^Department of Laboratory Medicine, Affiliated Hospital of Jiangsu University, Zhenjiang 212013, China; ^2^Department of Pathology, The First Affiliated Hospital of USTC, Division of Life Sciences and Medicine, University of Science and Technology of China, Hefei 230036, China; ^3^Department of Laboratory Medicine, School of Medicine, Jiangsu University, Zhenjiang 212013, China; ^4^Institute of Medical Genetics and Reproductive Immunity, School of Medical Science and Laboratory Medicine, Jiangsu College of Nursing, Huai'an 223005, China

## Abstract

Long noncoding RNAs (lncRNAs) are revealed to be involved in the tumorigenesis and progression of human malignancies mediated by microRNA (miRNA) via the competing endogenous RNA (ceRNA) mechanism, a newly proposed “RNA language.” However, the lncRNA-associated competing triplet (lncACT) network among ceRNA transcripts in clear cell renal cell carcinoma (ccRCC) is currently lacking. We carried out differential expression analysis to identify aberrantly expressed lncRNAs, miRNAs, and mRNAs by analyzing the RNA-seq data of 420 ccRCC tissues and 71 noncancerous kidney tissues obtained from The Cancer Genome Atlas (TCGA). Then, a ccRCC-specific ceRNA network was built using computational algorithms, including miRcode, TargetScan, miRanda, and miRTarBase. In total, 1491 dysregulated lncRNAs were found between normal renal tissues and ccRCC (fold change > 4 and false discovery rate < 0.01). A ceRNA network that comprised of 46 DElncRNAs, 11 DEmiRNAs, and 55 DEmRNAs was established by integrating the lncRNA/miRNA and miRNA/mRNA interactions into lncACTs. Several lncRNAs were identified to be significantly associated with clinical features of ccRCC patients. Notably, four key lncRNAs (TCL6, HOTTIP, HULC, and PCGEM1) were tightly correlated with both patients' clinical characteristics and overall survival (log-rank *P* < 0.05), indicating their potential important roles in ccRCC. HOTTIP may be a potential prognostic and therapeutic molecular marker for ccRCC patients. Collectively, our results provide a comprehensive view of the lncRNA-associated ceRNA regulatory network for a better understanding of the mechanisms and prognosis biomarkers for ccRCC.

## 1. Introduction

Renal cell carcinoma (RCC) is the most lethal urinary system malignancy in adults with an increasing morbidity globally [1]. It is estimated that 76,080 new cases and 13,780 deaths from kidney malignancies occurred in the world in 2021 [2]. RCC, as a heterogeneous group of disease, is subdivided into several histological subtypes according to the different nephron cell types that tumors derived from, including clear cell RCC (ccRCC, ~75%), papillary RCC (pRCC, ~15%), and chromophobe RCC (chRCC, ~5%) [3]. ccRCC is the predominant and most malignant subtype of renal carcinoma. Although the diagnosis of ccRCC improved mainly due to the advanced imaging detection technologies, the clinical behaviors of ccRCC patients are aggressive, especially the high rate of metastatic progression [4]. Therefore, identification of the molecular mechanisms underlying ccRCC for developing diagnostic markers and therapeutic targets becomes urgently needed.

Noncoding RNAs (ncRNAs) are categorized into long ncRNAs and short ncRNAs according to their length. The noncoding RNA transcripts more than 200 nucleotides long are generally termed as “long noncoding RNAs” (lncRNAs) [5]. lncRNAs have been recognized to involve in the pathogenesis of multiple cancers by disrupting various biological processes [6]. The abnormal expressions of microRNAs (miRNAs, 20-22 nucleotide in length) participate in the oncogenesis and cancer progression [7]. In recent years, lncRNAs were verified to function as competing endogenous RNAs (ceRNAs) to communicate with other RNAs via sharing miRNA-binding sites. This lncRNA-miRNA-RNA interaction was a subclass of ceRNAs, called lncRNA-associated competing triplets (lncACTs) [8]. In 2014, Xia et al. firstly constructed a lncACT cross-talk network in gastric cancer and also established a bioinformatics-based approach to predict cancer-associated ceRNA network [9]. Subsequently, several cancer-specific ceRNA networks have also been revealed in various cancers, including hepatocellular carcinoma [10], bladder cancer [11], and thyroid carcinoma [12].

However, there are only limited studies so far on lncACTs in RCC. lncRNA MALAT1 has been identified to function as a ceRNA by mediating the MALAT1/mir-200s/ZEB2 pathway to facilitate ccRCC proliferation and metastasis [13]. lncRNA HOTAIR, an oncogene in various tumors, was also reported to act as a ceRNA to promote HIF-1*α*/AXL cascade by binding mir-217 in RCC [14]. A drug resistance-related lncRNA lncARSR disseminated sunitinib resistance by sponging mir-34/mir-449 to increase target genes expression in RCC cells [15]. Fan et al. constructed lncRNA-related ceRNA network and discovered the nomograms and related infiltrating immune cells to predict prognosis of pRCC patients [16]. However, huge genetic heterogeneity exists among different histologic subtypes of RCC [17]. In this study, differentially expressed lncRNAs, miRNAs, and mRNAs (DElncRNAs, DEmRNAs, and DEmiRNAs) were screened out of the expression profiles of a 420 ccRCC patient cohort from The Cancer Genome Atlas (TCGA). A ccRCC specific ceRNA regulatory network was also built based on the potential competing triplets of lncRNA/miRNA/mRNA predicted by computational algorithms and databases. We also identified several key lncRNAs to be associated with ccRCC progression and prognosis.

## 2. Materials and Methods

### 2.1. Patient Dataset

TCGA is a public database providing researchers open access to the multiple cancer genomic profiles for analyses and publications [18]. This study meets the freedom-to-publish criteria announced on TCGA website (https://cancergenome.nih.gov/publications/publicationguidelines). A cohort of 537 ccRCC patients obtained from TCGA was downloaded for this study. The exclusion criteria included the following: (1) patients without complete clinicopathological data, including age, gender, race, TNM stage, and pathologic stage (12 cases); (2) patients with follow-up data over 2000 days (84 cases); and (3) patients with incomplete RNA-seq or miRNA-seq data (21 cases). In total, 420 ccRCC patients (cohort T) and 71 normal samples (cohort N) were enrolled in this study. The RNA and miRNA expression data (level 3) were produced from IlluminaHiseq_RNASeq and IlluminaHiseq_miRNASeq sequencing platform and prenormalized by TCGA archive (http://cancergenome.nih.gov).

### 2.2. Construction of lncACT Cross-Talk Network

We carried out differential expression analysis with edgeR package in Bioconductor [19]. Stringent filtering criteria were all set as |log_2_FC| > 2 and FDR < 0.01 (FC: fold change; FDR: false discovery rate). Among these differentially expressed genes (DEGs), the putative interactions of miRNA-lncRNA were collected from miRcode [20]. Different miRNA-target prediction algorithms, including experimentally validated database TargetScan (http://www.targetscan.org/mamm_31/) [21], miRanda (http://www.microrna.org/microrna/home.do) [22], and miRTarBase (http://mirtarbase.mbc.nctu.edu.tw/) [23], were used to predict the miRNA target mRNAs. These tools provide miRNA-target interactions with comprehensive annotation and experimental validation. Finally, the lncRNA-associated ceRNA network of ccRCC was integrated and visualized based on the above competing triplets using Cytoscape v3.5.1 (http://www.cytoscape.org/) [24].

### 2.3. Functional Enrichment Analysis

To access functional roles of the genes in the ceRNA network, Gene Ontology (GO) was performed using Database for Annotation, Visualization and Integration Discovery (DAVID, https://david.ncifcrf.gov/) (*P* value < 0.05). Meanwhile, pathway analysis was conducted using Kyoto Encyclopedia of Genes and Genomes (KEGG) by KOBAS 3.0 (*P* value < 0.01).

### 2.4. Coexpression Analysis

Correlation test was conducted by the R software to figure out the coexpressed genes associated with HOTTIP (|cor|>0.3 and *P* value < 0.001).

### 2.5. Drug Sensitivity Analysis

Drug sensitivity analysis was carried out with pRRophetic package in Bioconductor to discover the drugs with significant differences in sensitivity between HOTTIP high and low groups (*P* value < 0.05).

### 2.6. Statistical Analysis

Unpaired *t*-test was applied to identify DEGs and the difference of DElnRNAs between different pathological subgroups. The associations between DElncRNAs expression and patients' overall survival (OS) were analyzed by univariate Cox proportional hazards regression (log-rank *P* < 0.05). Kaplan-Meier method was employed to generate overall survival curves.

## 3. Results

### 3.1. Patient Characteristics

A total of 420 patients who were pathologically diagnosed as ccRCC and 71 normal samples were enrolled in this study. The clinicopathological information of study population is summarized in Table 1. The median age was 60 years. Consistent with a previous report [25], white male individuals appeared to be the majority of RCC patients with the gender ratio (male/female) of 1.9/1 and white race ratio of 86.7%.

### 3.2. Screening Results of DEGs in ccRCC

**Figure 1:**
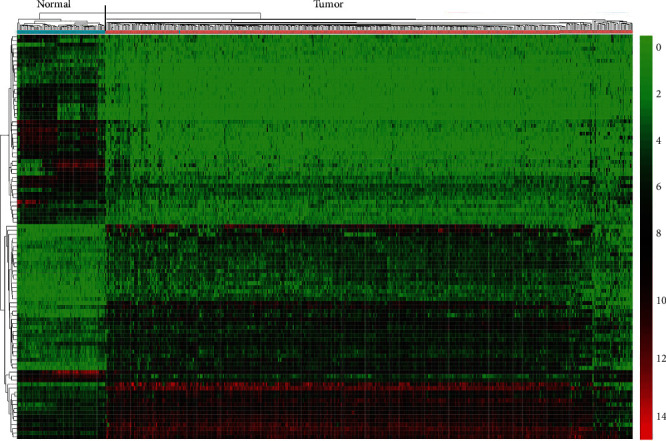
Heatmap of top 50 upregulated and top 50 downregulated DElncRNAs in clear cell renal cell carcinoma (ccRCC). Blue and red stripes represent normal samples and tumor samples, respectively. Descending normalized expression level is colored from red to green.

After screening the RNA and miRNA expression profiles by the threshold of |log_2_FC| > 2 and FDR < 0.01, we found 1491 DElncRNAs, 2368 DEmRNAs, and 53 miRNAs that aberrantly expressed between ccRCC tumor tissues and normal tissues. Among them, 989 lncRNAs, 1610 mRNAs, and 32 miRNAs were upregulated, while 502 lncRNAs, 758 mRNAs, and 21 miRNAs were downregulated in cohort T compared with cohort N. The total upregulated and downregulated lncRNAs, mRNAs, and miRNAs were listed in Table S1-6. Hierarchical clustering was further used to identify expression patterns of DEGs between two cohorts. The top 50 overexpressed and top 50 downexpressed lncRNAs were visualized in the heatmap, which showed that ccRCC tumor tissues had significantly different expression patterns from normal tissues (Figure 1 and Table S7).

### 3.3. lncACT Cross-Talk Network in ccRCC

**Figure 2:**
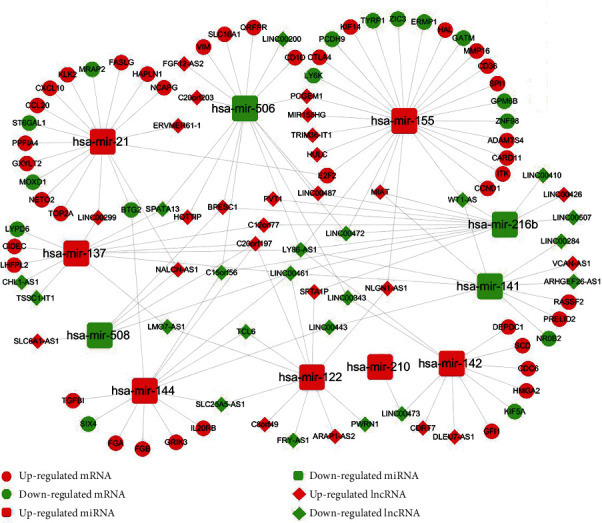
The ceRNA regulatory network of ccRCC. Expression levels and different RNA types are represented by different colors and different shapes, respectively. ceRNA: competitive endogenous RNA.

The ceRNA hypothesis is described as a complex posttranscriptional regulatory mechanism between lncRNAs and other RNAs mediated by miRNAs through sharing miRNA response elements [26]. Therefore, further analysis was performed to establish lncACT cross-talk network based on the above DEGs in ccRCC. We got 11 specific DEmiRNAs that targeted on 46 DElncRNAs by miRcode online tools, which is a lncRNA-miRNA interaction prediction database (Table 2). To further analyze these DEmiRNAs, we comprehensively considered the miRNA-mRNA interactions obtained from TargetScan, miRTarBase, and miRanda databases to enhance the predictive reliability. A total of 55 targeted mRNAs were predicted to interact with 7 DEmiRNAs and were also involved in the above 2368 DEmRNAs (Table 3). By integrating these lncRNA/miRNA and miRNA/mRNA interactions into lncACTs, the ceRNA network is constructed and visualized in Figure 2, containing 46 DElncRNAs, 11 DEmiRNAs, and 55 DEmRNAs.

### 3.4. Functional Enrichment Analysis

**Figure 3:**
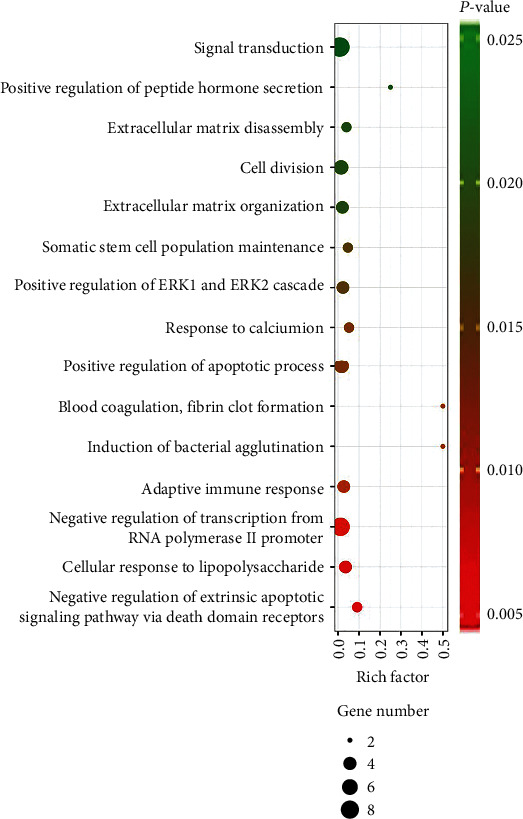
Top 15 enriched Gene Ontology biological process terms of DEmRNAs in the ceRNA network. The size of balls represents gene number, and different colors represent *P* value.

To identify the functions of the 55 DEmRNAs involved in the ceRNA network, functional analysis was performed. GO analysis revealed 26 enriched GO categories in the “biological processes” (*P* value < 0.05), top 15 of which are visualized in Figure 3. There were two apoptotic processes significantly enriched in GO terms (GO:1902042 and GO:0043065). According to *P* value < 0.01, 27 KEGG categories were selected as significantly enriched KEGG pathways. The top ten enriched pathways are listed in Table 4, including four cancer-related pathways (microRNAs in cancer, bladder cancer, transcriptional misregulation in cancer, and pathways in cancer). Cyclin D1 (CCND1) was notably involved in six of the top ten pathways, indicating its complex roles in the progress of the tumor.

### 3.5. The Clinical Relevance of DElncRNAs in ccRCC

We next analyzed the association between the 46 DElncRNAs in the ceRNA network and clinicopathological features. A total of eight lncRNAs were discriminatively expressed in different clinical feature subgroups (|log_2_FC| > 2 and FDR < 0.01) (Table 5). We found six downregulated lncRNAs (C12orf77, TCL6, C8orf49, PCGEM1, and ERVMER61-1), and two upregulated lncRNAs (HOTTIP and LINC00200) were significantly related to the progression of ccRCC. Both C12orf77 and TCL6 not only could inhibit tumor growth (T3 + T4 vs. T1 + T2) but also downexpressed in individuals with high levels of the pathologic stage, implying their negative roles in tumor development of ccRCC. HULC was identified to promote lymph node metastasis; however, low expression of HULC seemed to be correlated with high levels of tumor size, distant metastases, and pathologic stage.

**Figure 4:**
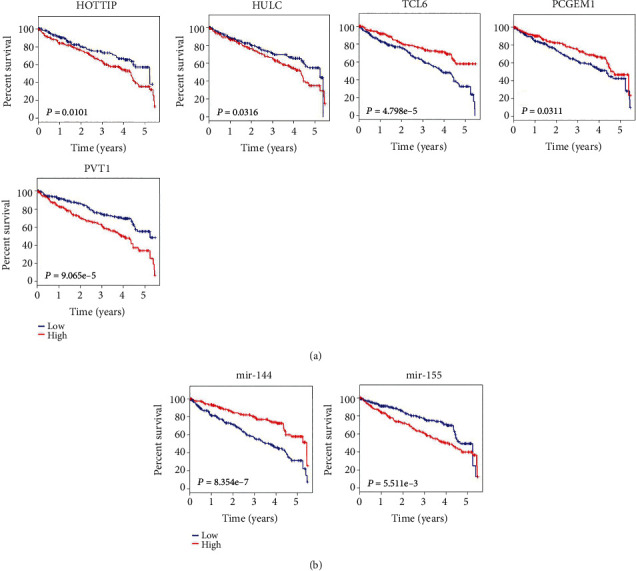
Kaplan-Meier curves for five DElncRNAs (a) and two DEmiRNAs (b) associated with overall survival. Horizontal axis, overall survival time (years); vertical axis, survival function. Patients were divided into “high” group (≥median) and “low” group (<median) according to the gene expression levels.

Subsequently, the Kaplan-Meier analysis was applied to investigate overall survival time for DElncRNAs in ccRCC patients. Among the 46 DElncRNAs involved in the lncACT network, five lncRNAs (TCL6, PCGEM1, FGF12-AS2, LINC00443, and LINC00472) were found positively associated with overall survival by univariate Cox regression analysis (log-rank *P* < 0.05), while another eight lncRNAs (HOTTIP, HULC, PVT1, WT1-AS, C20orf203, NALCN-AS1, TRIM36-IT1, and LINC00299) were negatively correlated with survival. The Kaplan-Meier curves of HOTTIP, HULC, TCL6, and PCGEM1, which also differentially expressed in clinical feature comparisons, are shown in Figure 4(a). The Kaplan-Meier curve analysis was also employed to investigate overall survival for the DEmiRNAs associated with this four lncRNAs. Notably, increased expression of mir-144, which was predicted to interact with TCL6, was positively associated with prognosis. The high expression of mir-155, which potentially targeted HULC and PCGEM1, was correlated with poor prognosis (Figure 4(b)).

### 3.6. High Expression of HOTTIP Associated with Decreased Drug Sensitivity

**Figure 5:**
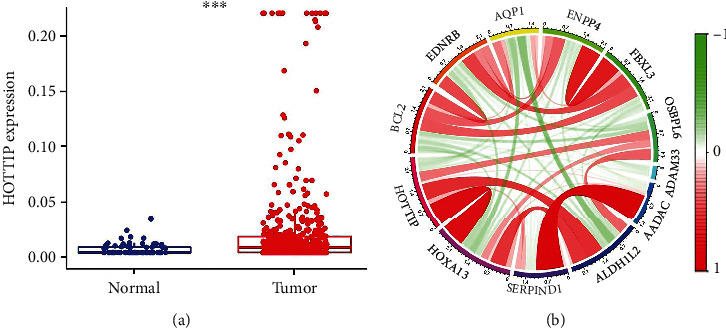
HOTTIP expression in ccRCC patients. (a) HOTTIP expression level in 420 ccRCC tumor and adjacent nontumor samples from the TCGA data determined by RNAseq. (b) The correlation circle diagram of the significantly correlated genes with HOTTIP in ccRCC. The gene names are labeled outside the circle, and the line colors indicate the relationships between genes. Red and green lines represent positive and negative relationships, respectively.

**Figure 6:**
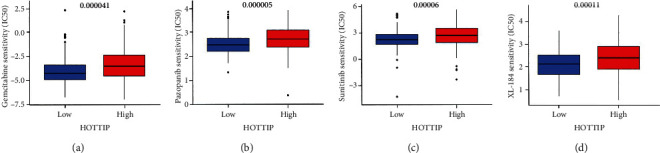
The half maximal inhibitory concentration (IC_50_) of gemcitabine (a), pazopanib (b), sunitinib (c), and XL-184 (d) in “HOTTIP high” group (≥median) and “HOTTIP low” group (<median) in ccRCC patients.

To further understand the expression of HOTTIP in ccRCC patients, transcriptome sequencing data of 420 ccRCC and 71 normal samples were extracted from the TCGA database. HOTTIP expression level was significantly higher in ccRCC patients than in normal controls (Figure 5(a)). Coexpression analysis showed that 55 genes were associated with HOTTIP expression, including 7 negatively correlated genes and 48 positively correlated genes (Table S8). The correlation circle diagram showed that HOXA13, SERPIND1, ALDH1L2, AADAC, ADAM33, and OSBPL6 were positively correlated with HOTTIP, and BCL2, EDNRB, AQP1, ENPP4, and FBXL3 were negatively correlated with HOTTIP (Figure 5(b)). Drug sensitivity analysis identified that the half maximal inhibitory concentration (IC_50_) of gemcitabine, pazopanib, sunitinib, and XL-184 in ccRCC patients with high HOTTIP expression was significantly higher than those in patients with low HOTTIP expression, indicating that patients with high HOTTIP expression were less sensitive to these treatments (Figure 6).

## 4. Discussion

Previous reports have shown that lncRNAs participated in tumorigenesis, cancer progression, and metastasis of RCC and functioned as oncogenes or tumor suppressors. Several studies have conducted genomic microarrays to reveal the expression patterns of lncRNAs based on small sample size [27, 28]. The tumor-specific lncACT cross-talk network has been previously described in chRCC [29]. However, different RCC histological subtypes encompass a wide diversity of molecular mechanisms for their tumorigenesis. Thus, there is an urgent to explore the lncRNA-associated ceRNA network in ccRCC. In the current study, we analyzed the expression profile data of ccRCC patient cohort in TCGA archive to comprehensively identify the landscape regarding how tumor-specific lncRNAs function in ccRCC. We successfully built the lncRNA-associated ceRNA network in ccRCC according to the predicted competing triplets among DElncRNAs, DEmRNAs, and DEmiRNAs.

Recent researches have demonstrated that lncRNAs could communicate with miRNAs and indirectly regulate miRNA targets via competing interactions. The lncACT interactions might actively function as valuable prognostic indicators in cancers [8]. Hence, we speculate that some specific lncACT cross-talks comprising lncRNA, miRNA, and mRNA may affect ccRCC progression. We utilized stringent criteria to identify DElncRNAs, DEmiRNAs, and DEmRNAs between ccRCC tumor tissues and normal tissues and then applied several bioinformatics strategies to increase the predictive accuracy of RNA-RNA interactions. Finally, 46 DElncRNA, 11 DEmiRNAs, and 55 DEmRNAs constituted the lncACT coexpression network in ccRCC. To explore the biological functions of these ceRNA network-involved genes, KEGG pathway analysis showed that the key DEmRNAs were significantly enriched in cancer-related pathways, implicating their vital roles in tumorigenesis. Among the 46 key DElncRNAs, four lncRNA (TCL6, HOTTIP, HULC, and PCGEM1) not only had correlations with clinical features but could also affect ccRCC patients' outcome, strongly suggesting their important roles as prognostic biomarkers for ccRCC. Consistent with our results, a recent study also verified that low expression of TCL6 was correlated with advanced clinicopathological features and poor prognosis of ccRCC patients. Furthermore, preliminary experiments have indicated TCL6 as a potential antioncogene by inhibiting proliferation and promoting apoptosis of ccRCC cell lines [30]. We predicted that TCL6 might interact with mir-144, of which the potential target genes included an antiproliferative gene BTG2. BTG2 was reported to participate in cell cycle regulation and subsequently involved in cell proliferation in carcinogenesis [31]. Therefore, it deserves further experiments to elucidate the mechanism underlying the effects of TCL6-associated competing triplets on ccRCC.

The upexpression of HOXA transcript at the distal tip (HOTTIP), as a critical oncogenic lncRNA, has been correlated with poor overall survival in various malignancies [32, 33]. We predicted that the high expression of HOTTIP with an approximate 12-fold change in ccRCC tumor tissues may promote tumor growth and a statistic shorter overall survival, which is consistent with previous studies [34, 35]. We found a significant positive correlation between HOTTIP and HOXA13 expression in ccRCC patients. It was demonstrated that HOTTIP transcriptionally regulates HOXA13 in esophageal squamous cell carcinoma cells to promote carcinogenesis and metastasis [36]. Because of the physical contiguity of HOTTIP with HOXA13, we hypothesized that HOTTIP and HOXA13 may closely coordinate to regulate the occurrence and development of ccRCC [37]. More importantly, we found that patients with high HOTTIP expression were less sensitive to clinical therapeutic drugs, including gemcitabine, pazopanib, sunitinib, and XL-184, than patients with low HOTTIP expression, indicating that high HOTTIP expression may lead to drug resistance in ccRCC patients.

HULC, a universal oncogenic lncRNA in human cancers, was reported to be strongly overexpressed in several cancer types, including hepatocellular carcinoma, gastric cancer, pancreatic cancer, and osteosarcoma [38]. However, the role of HULC in ccRCC still remains largely unclear. We predicted that the increased expression (~6 folds) of HULC in ccRCC tumor tissues might promote lymphatic metastasis and poor prognosis. CCND1 might be regulated by HULC through the interaction with mir-155 in ccRCC. Similarly, it has been previously revealed that HULC knockdown induced cell growth arrest and apoptosis through inhibiting CCND1 expression in diffuse large B-cell lymphoma cells [39]. The overexpression of PCGEM1, as a prostate-specific lncRNA, was correlated with high risk of prostate cancer [40, 41]. On the contrary, we found that the downregulation of PCGEM1 might prolong metastasis status and shorten survival time of ccRCC patients. To the best of our knowledge, this study firstly reported the potential functions of HULC, HOTTIP, and PCGEM1 in ccRCC to date. Furthermore, we also verified lncRNA PVT1 to be an oncogenic lncRNA in ccRCC. It has been reported that ccRCC has the strongest upregulated expression of PVT1 among all cancer types and served as a prognostic factor of renal cancer [42, 43].

However, since our study was conducted based on TCGA cohort by computational analysis, future studies should be designed to verify these lncACT cross-talks and their multiple functions in ccRCC progression. In conclusion, our study has built a newly identified ceRNA network of ccRCC based on hundreds of clinical specimens from TCGA. The ceRNA network discloses that many oncogenes and antioncogenes might contribute to ccRCC development, which can expand our understanding of the roles of lncACTs in tumorigenesis. Importantly, we have identified several lncRNAs to be potential prognostic factors and molecular targets for ccRCC patients.

## Figures and Tables

**Table 1:** Clinical characteristics of 420 patients with ccRCC in cohort T.

Parameter	Cohort T (*n* = 420) (%)
Age (mean ± SD^1^)	60.4 ± 12.1
Gender	
Male	275 (65.5)
Female	145 (34.5)
Race	
Asian	8 (1.9)
White	364 (86.7)
Black or African American	48 (11.4)
Pathologic stage	
Stage I	199 (47.4)
Stage II	43 (10.3)
Stage III	106 (25.2)
Stage IV	72 (17.1)
Tumor size	
T1	205 (48.8)
T2	51 (12.1)
T3	153 (36.5)
T4	11 (2.6)
Lymph node	
N0	181 (43.1)
N1	14 (3.3)
NX	225 (53.6)
Metastasis status	
M0	326 (77.6)
M1	67 (16.0)
MX	27 (6.4)

^1^Standard deviation.

**Table 2:** The 11 specific DEmiRNAs and 46 target DElncRNAs in ccRCC.

lncRNA	miRNAs	lncRNA	miRNAs
ARAP1-AS2	mir-122	LINC00461	mir-122, mir-137, mir-141, mir-144, mir-216b, mir-508
ARHGEF26-AS1	mir-141	LINC00472	mir-155, mir-216b, mir-506
BPESC1	mir-216b, mir-506, mir-508	LINC00473	mir-142, mir-210
C12orf77	mir-137, mir-216b	LINC00487	mir-216b, mir-506
C15orf56	mir-144, mir-216b, mir-506	LINC00507	mir-216b
C20orf197	mir-122, mir-137, mir-144, mir-508	LMO7-AS1	mir-122, mir-137
C20orf203	mir-506	LY86-AS1	mir-137, mir-141, mir-142, mir-155, mir-216b, mir-506
C8orf49	mir-122	MIAT	mir-141, mir-155, mir-216b
CDRT7	mir-142	MIR155HG	mir-155
CHL1-AS1	mir-137	NALCN-AS1	mir-21, mir-508
DLEU7-AS1	mir-142	NLGN1-AS1	mir-122, mir-155
ERVMER61-1	mir-21	PCGEM1	mir-155, mir-506
FGF12-AS2	mir-506	PVT1	mir-216b
FRY-AS1	mir-122	PWRN1	mir-122
HOTTIP	mir-137, mir-506	SFTA1P	mir-122, mir-216b
HULC	mir-155	SLC25A5-AS1	mir-122, mir-144
LINC00200	mir-506	SLC6A1-AS1	mir-508
LINC00284	mir-141	SPATA13	mir-137, mir-506
LINC00299	mir-137, mir-21	TCL6	mir-122, mir-144
LINC00343	mir-142, mir-506	TRIM36-IT1	mir-155
LINC00410	mir-216b	TSSC1-IT1	mir-137
LINC00426	mir-216b	VCAN-AS1	mir-141
LINC00443	mir-141, mir-144	WT1-AS	mir-141, mir-155, mir-216b

**Table 3:** The 7 DEmiRNAs and 55 target DEmRNAs in ccRCC.

miRNA	mRNAs targeted by miRNA
mir-137	CIDEC, LHFPL2, LYPD6
mir-141	NR0B2, PRELID2, RASSF2
mir-142	CDC6, DEPDC1, GFI1, HMGA2, KIF5A, SCD
mir-144	BTG2, FGA, FGB, GRIK3, IL20RB, SIX4, TGFBI
mir-155	ADAMTS4, CARD11, CCND1, CD36, CTLA4, E2F2, ERMP1, GATM, GPM6B, HAL, ITK, KIF14, LY6K, MMP16, PCDH9, SPI1, TYRP1, ZIC3, ZNF98
mir-21	BTG2, CCL20, CXCL10, E2F2, FASLG, GXYLT2, HAPLN1, KLK2, MOXD1, MRAP2, NCAPG, NETO2, PPFIA4, ST6GAL1, TOP2A
mir-506	CD1D, QRFPR, SLC16A1, VIM

**Table 4:** KEGG^1^ pathway analysis of the DEmRNAs involved in the ceRNA network.

Pathway ID	Description	*P* value	Numbers of DEmRNAs
hsa05206	MicroRNAs in cancer	6.39*E*-05	CCND1, E2F2, MMP16, VIM, HMGA2
hsa04660	T cell receptor signaling pathway	4.60*E*-04	ITK, CTLA4, CARD11
hsa04060	Cytokine-cytokine receptor interaction	5.30*E*-04	FASLG, IL20RB, CXCL10, CCL20
hsa04110	Cell cycle	7.37*E*-04	CCND1, CDC6, E2F2
hsa04152	AMPK signaling pathway	7.54*E*-04	SCD, CCND1, CD36
hsa00514	Other types of O-glycan biosynthesis	9.62*E*-04	GXYLT2, ST6GAL1
hsa05161	Hepatitis B	1.17*E*-03	FASLG, CCND1, E2F2
hsa05219	Bladder cancer	1.63*E*-03	CCND1, E2F2
hsa05202	Transcriptional misregulation in cancer	2.10*E*-03	SPI1, SIX4, HMGA2
hsa05200	Pathways in cancer	2.31*E*-03	SPI1, FASLG, CCND1, E2F2

^1^KEGG: Kyoto Encyclopedia of Genes and Genomes.

**Table 5:** The lncRNAs tightly correlated with ccRCC patients' clinical characteristics.

Comparisons	Downregulated	Upregulated
Tumor size (T3 + T4 vs. T1 + T2)	C12orf77, HULC, TCL6	HOTTIP
Lymph node (N1 vs. N0)		HULC
Metastasis status (M1 vs. M0)	C8orf49, PCGEM1, HULC ERVMER61-1	LINC00200
Pathologic stage (stage III + IV vs. stage I + II)	C12orf77, HULC, TCL6	

## Data Availability

The data used to support the findings of this study are available from the corresponding authors upon request.

## Supplementary Materials

Supplementary Materials Table S1: the list of upregulated lncRNAs in ccRCC. Table S2: the list of downregulated lncRNAs in ccRCC. Table S3: the list of upregulated mRNAs in ccRCC. Table S4: the list of downregulated mRNAs in ccRCC. Table S5: the list of upregulated miRNAs in ccRCC. Table S6: the list of downregulated miRNAs in ccRCC. Table S7: the list of top 100 dysregulated (50 upregulated and 50 downregulated) lncRNAs in consistent with Figure 1. Table S8: the list of genes coexpressed with HOTTIP in ccRCC.
