# Supplementary material for: A Computationally Constructed lncRNA-Associated Competing Triplet Network in Clear Cell Renal Cell Carcinoma
Source: Dis Markers. 2022 Nov 17;2022:8928282. doi: 10.1155/2022/8928282 (PMC9691318; doi:10.1155/2022/8928282)
Supplement: Supplementary Materials — Table S1: the list of upregulated lncRNAs in ccRCC. Table S2: the list of downregulated lncRNAs in ccRCC. Table S3: the list of upregulated mRNAs in ccRCC. Table S4: the list of downregulated mRNAs in ccRCC. Table S5: the list of upregulated miRNAs in ccRCC. Table S6: the list of downregulated miRNAs in ccRCC. Table S7: the list of top 100 dysregulated (50 upregulated and 50 downregulated) lncRNAs in consistent with Figure 1. Table S8: the list of genes coexpressed with HOTTIP in ccRCC. [file 8928282.f1.zip › 8928282.f1/Table S1 (1).docx]

Table S1. The list of up-regulated lncRNAs in ccRCC.

| **Gene symbol** | **Ensemb ID** | **Fold Change (FC)  (T/N)** | **log_2_FC (T/N)** | ***P* value** | **FDR** |
| --- | --- | --- | --- | --- | --- |
| OSTM1-AS1 | ENSG00000225174 | 351.269526 | 8.456435 | 8.38E-56 | 2.35E-54 |
| TTC21B-AS1 | ENSG00000224490 | 287.348277 | 8.166657 | 3.18E-86 | 2.22E-84 |
| RP11-485M7.3 | ENSG00000251616 | 205.904882 | 7.685834 | 2.38E-12 | 7.37E-12 |
| RP11-380J14.1 | ENSG00000204362 | 129.672744 | 7.018731 | 1.41E-59 | 4.53E-58 |
| RP5-912I13.1 | ENSG00000260426 | 111.821944 | 6.805060 | 4.17E-17 | 1.82E-16 |
| RP11-211G23.2 | ENSG00000260877 | 91.892418 | 6.521874 | 2.99E-43 | 5.20E-42 |
| RP11-586K2.1 | ENSG00000253553 | 80.292710 | 6.327197 | 4.10E-17 | 1.79E-16 |
| RP6-191P20.4 | ENSG00000236064 | 67.136467 | 6.069025 | 1.09E-54 | 2.96E-53 |
| AC073115.6 | ENSG00000237471 | 62.372312 | 5.962834 | 2.13E-48 | 4.57E-47 |
| AC079466.1 | ENSG00000266976 | 61.480970 | 5.942068 | 1.10E-17 | 5.01E-17 |
| RP11-510C10.3 | ENSG00000224149 | 56.275185 | 5.814427 | 2.55E-23 | 1.63E-22 |
| AC008060.7 | ENSG00000218672 | 55.161811 | 5.785598 | 7.86E-18 | 3.60E-17 |
| AP000439.3 | ENSG00000255774 | 53.756249 | 5.748361 | 1.77E-40 | 2.77E-39 |
| RP11-719N22.2 | ENSG00000242545 | 49.002752 | 5.614791 | 3.51E-19 | 1.76E-18 |
| RP5-884M6.1 | ENSG00000228742 | 46.023078 | 5.524286 | 4.18E-17 | 1.82E-16 |
| RP11-865I6.2 | ENSG00000254337 | 44.576030 | 5.478196 | 9.42E-31 | 9.03E-30 |
| AC068858.1 | ENSG00000254654 | 42.101431 | 5.395797 | 1.37E-47 | 2.85E-46 |
| RP11-14C10.5 | ENSG00000273585 | 41.263786 | 5.366804 | 1.98E-53 | 5.11E-52 |
| AC006262.5 | ENSG00000268621 | 41.001489 | 5.357604 | 7.52E-15 | 2.82E-14 |
| RP11-372E1.4 | ENSG00000243818 | 38.679859 | 5.273511 | 6.48E-12 | 1.96E-11 |
| RP11-510C10.2 | ENSG00000224127 | 38.671119 | 5.273185 | 6.96E-21 | 3.89E-20 |
| AC073115.7 | ENSG00000229628 | 36.637836 | 5.195262 | 8.81E-45 | 1.63E-43 |
| RP11-206M11.7 | ENSG00000244468 | 36.505157 | 5.190028 | 2.17E-18 | 1.03E-17 |
| RP11-161D15.1 | ENSG00000250708 | 33.775161 | 5.077891 | 2.26E-11 | 6.57E-11 |
| FLJ26245 | ENSG00000261122 | 33.292929 | 5.057144 | 1.48E-11 | 4.34E-11 |
| AC015977.6 | ENSG00000225378 | 33.212374 | 5.053649 | 2.84E-42 | 4.84E-41 |
| RP11-513G11.2 | ENSG00000228271 | 32.976871 | 5.043383 | 2.58E-81 | 1.60E-79 |
| LINC00462 | ENSG00000233610 | 32.014970 | 5.000675 | 9.98E-39 | 1.42E-37 |
| LINC00460 | ENSG00000233532 | 30.648403 | 4.937740 | 2.28E-20 | 1.23E-19 |
| LUCAT1 | ENSG00000248323 | 30.293279 | 4.920926 | 5.75E-46 | 1.11E-44 |
| RP1-105O18.1 | ENSG00000232299 | 30.242696 | 4.918515 | 4.48E-26 | 3.38E-25 |
| RP11-155G14.6 | ENSG00000240758 | 28.535124 | 4.834667 | 1.06E-93 | 8.61E-92 |
| CTD-3128G10.7 | ENSG00000276980 | 28.494451 | 4.832609 | 4.32E-40 | 6.57E-39 |
| GACAT2 | ENSG00000265962 | 28.337082 | 4.824619 | 2.85E-40 | 4.41E-39 |
| AP000439.1 | ENSG00000255980 | 28.090454 | 4.812008 | 9.58E-18 | 4.37E-17 |
| LINC01419 | ENSG00000253898 | 27.945687 | 4.804554 | 3.85E-08 | 8.47E-08 |
| RP11-123K3.9 | ENSG00000276727 | 27.798413 | 4.796931 | 1.47E-77 | 7.97E-76 |
| RP11-115N4.1 | ENSG00000243144 | 27.749610 | 4.794396 | 3.78E-64 | 1.43E-62 |
| RP11-454P21.1 | ENSG00000249937 | 27.698405 | 4.791731 | 2.71E-10 | 7.16E-10 |
| LINC00887 | ENSG00000214145 | 27.068592 | 4.758548 | 2.28E-70 | 1.02E-68 |
| CTC-327F10.5 | ENSG00000248362 | 26.948028 | 4.752108 | 8.48E-24 | 5.55E-23 |
| RP11-605F22.1 | ENSG00000259385 | 26.776999 | 4.742922 | 1.13E-09 | 2.83E-09 |
| EGFR-AS1 | ENSG00000224057 | 25.946116 | 4.697447 | 6.11E-30 | 5.66E-29 |
| CTD-2377D24.4 | ENSG00000242407 | 25.639555 | 4.680299 | 2.98E-12 | 9.17E-12 |
| RP11-547D24.1 | ENSG00000233542 | 25.634032 | 4.679989 | 2.61E-89 | 1.89E-87 |
| CTC-327F10.4 | ENSG00000251320 | 25.297376 | 4.660916 | 2.68E-23 | 1.71E-22 |
| SFTA1P | ENSG00000225383 | 24.855036 | 4.635466 | 1.49E-62 | 5.40E-61 |
| RP11-114M1.2 | ENSG00000236385 | 24.148199 | 4.593844 | 3.63E-15 | 1.39E-14 |
| RP11-161D15.2 | ENSG00000250043 | 24.023115 | 4.586351 | 3.25E-13 | 1.08E-12 |
| LINC01583 | ENSG00000259518 | 23.912896 | 4.579717 | 5.12E-18 | 2.37E-17 |
| ELDR | ENSG00000280890 | 23.590174 | 4.560114 | 1.97E-35 | 2.43E-34 |
| AC068492.1 | ENSG00000237262 | 22.512143 | 4.492631 | 3.42E-34 | 3.93E-33 |
| RP11-167N4.2 | ENSG00000255847 | 22.033504 | 4.461627 | 6.76E-39 | 9.68E-38 |
| CASC6 | ENSG00000224944 | 21.993741 | 4.459021 | 6.52E-14 | 2.29E-13 |
| LINC01077 | ENSG00000233456 | 21.802442 | 4.446418 | 3.82E-28 | 3.22E-27 |
| RP3-340N1.2 | ENSG00000227066 | 21.499242 | 4.426214 | 2.93E-22 | 1.75E-21 |
| RP11-529E15.1 | ENSG00000272027 | 21.481413 | 4.425017 | 7.93E-43 | 1.36E-41 |
| RP11-414H23.3 | ENSG00000251093 | 21.466276 | 4.424000 | 7.79E-28 | 6.46E-27 |
| PP12613 | ENSG00000226757 | 20.329110 | 4.345475 | 8.53E-29 | 7.47E-28 |
| RP6-91H8.3 | ENSG00000269927 | 20.277504 | 4.341808 | 3.94E-30 | 3.70E-29 |
| RP11-400N13.3 | ENSG00000232679 | 20.170507 | 4.334175 | 7.94E-14 | 2.76E-13 |
| AC114803.3 | ENSG00000230432 | 20.039946 | 4.324807 | 1.48E-43 | 2.61E-42 |
| RP11-283G6.5 | ENSG00000255750 | 19.932355 | 4.317040 | 4.30E-45 | 8.06E-44 |
| RP11-361L15.4 | ENSG00000265408 | 19.837619 | 4.310167 | 7.41E-40 | 1.12E-38 |
| RP11-19D2.2 | ENSG00000261249 | 19.708695 | 4.300760 | 1.44E-49 | 3.17E-48 |
| CTC-472C24.1 | ENSG00000249150 | 19.475983 | 4.283624 | 6.60E-22 | 3.90E-21 |
| AC005550.3 | ENSG00000237070 | 19.338159 | 4.273379 | 4.73E-14 | 1.68E-13 |
| RP5-1120P11.3 | ENSG00000231881 | 19.128399 | 4.257644 | 4.21E-43 | 7.27E-42 |
| RP11-567E21.3 | ENSG00000229821 | 19.110094 | 4.256263 | 1.32E-10 | 3.58E-10 |
| AP000797.4 | ENSG00000257067 | 19.065603 | 4.252900 | 5.06E-24 | 3.35E-23 |
| LINC00879 | ENSG00000239589 | 18.944978 | 4.243744 | 2.40E-10 | 6.39E-10 |
| SIRPG-AS1 | ENSG00000237914 | 18.827363 | 4.234759 | 2.57E-33 | 2.79E-32 |
| AC069363.1 | ENSG00000277089 | 18.722032 | 4.226665 | 8.04E-43 | 1.38E-41 |
| RP11-429B14.4 | ENSG00000261478 | 18.640510 | 4.220369 | 8.54E-19 | 4.17E-18 |
| RP11-309M7.1 | ENSG00000261298 | 18.551912 | 4.213496 | 3.43E-42 | 5.79E-41 |
| PVT1 | ENSG00000249859 | 18.508177 | 4.210091 | 2.02E-171 | 5.35E-169 |
| RP11-1038A11.2 | ENSG00000256218 | 18.345152 | 4.197327 | 1.63E-07 | 3.40E-07 |
| WI2-8325B5.1 | ENSG00000276786 | 18.241856 | 4.189181 | 4.31E-25 | 3.04E-24 |
| IL20RB-AS1 | ENSG00000249407 | 18.167341 | 4.183275 | 4.51E-17 | 1.96E-16 |
| RP11-27G22.1 | ENSG00000254626 | 18.072804 | 4.175748 | 1.65E-19 | 8.50E-19 |
| RP11-212I21.2 | ENSG00000260135 | 18.021639 | 4.171658 | 4.01E-39 | 5.77E-38 |
| RP11-631F7.1 | ENSG00000224658 | 17.999189 | 4.169860 | 1.35E-16 | 5.68E-16 |
| AC007193.6 | ENSG00000267922 | 17.876869 | 4.160022 | 4.05E-11 | 1.15E-10 |
| RP11-96B2.1 | ENSG00000255325 | 17.806483 | 4.154331 | 4.20E-11 | 1.19E-10 |
| CTD-2194D22.3 | ENSG00000249116 | 17.712399 | 4.146688 | 5.30E-08 | 1.16E-07 |
| CTB-127C13.1 | ENSG00000247699 | 17.642332 | 4.140969 | 1.65E-33 | 1.83E-32 |
| MIAT | ENSG00000225783 | 17.610921 | 4.138398 | 2.30E-46 | 4.58E-45 |
| LINC01611 | ENSG00000231776 | 17.561971 | 4.134383 | 9.04E-18 | 4.13E-17 |
| RP4-668E10.4 | ENSG00000278254 | 17.463161 | 4.126243 | 1.09E-11 | 3.22E-11 |
| RP11-255G12.3 | ENSG00000258808 | 17.459649 | 4.125953 | 5.28E-39 | 7.60E-38 |
| AL592528.1 | ENSG00000205424 | 17.280134 | 4.111043 | 6.36E-18 | 2.93E-17 |
| RP11-73M14.1 | ENSG00000256714 | 17.091158 | 4.095178 | 8.47E-18 | 3.88E-17 |
| AF121898.3 | ENSG00000253500 | 16.693436 | 4.061209 | 3.84E-12 | 1.17E-11 |
| AP004372.1 | ENSG00000238117 | 16.574151 | 4.050863 | 2.07E-17 | 9.22E-17 |
| RP3-495K2.2 | ENSG00000229720 | 16.477722 | 4.042445 | 3.02E-14 | 1.08E-13 |
| RP11-29P20.1 | ENSG00000272515 | 16.385003 | 4.034304 | 1.26E-19 | 6.54E-19 |
| RP4-764O22.1 | ENSG00000232271 | 16.372915 | 4.033239 | 2.10E-39 | 3.07E-38 |
| RP11-554D15.1 | ENSG00000223786 | 16.057016 | 4.005132 | 1.54E-10 | 4.16E-10 |
| RP11-1151B14.2 | ENSG00000267675 | 15.880989 | 3.989229 | 2.46E-31 | 2.42E-30 |
| AC006262.4 | ENSG00000269729 | 15.709343 | 3.973551 | 2.17E-15 | 8.46E-15 |
| CTB-31N19.5 | ENSG00000273956 | 15.684891 | 3.971304 | 3.50E-13 | 1.16E-12 |
| AC097713.3 | ENSG00000228162 | 15.460907 | 3.950553 | 1.62E-23 | 1.04E-22 |
| IL21-AS1 | ENSG00000227145 | 15.451375 | 3.949663 | 3.82E-19 | 1.91E-18 |
| RP13-297E16.4 | ENSG00000223511 | 15.217249 | 3.927636 | 7.32E-51 | 1.69E-49 |
| AFAP1-AS1 | ENSG00000272620 | 15.215648 | 3.927484 | 3.04E-21 | 1.74E-20 |
| RP11-44K6.4 | ENSG00000254287 | 15.085044 | 3.915047 | 5.52E-32 | 5.60E-31 |
| LINC00160 | ENSG00000230978 | 15.023215 | 3.909122 | 1.22E-20 | 6.69E-20 |
| RP11-212I21.5 | ENSG00000274508 | 15.004410 | 3.907315 | 1.39E-31 | 1.39E-30 |
| LINC00626 | ENSG00000225826 | 14.966243 | 3.903640 | 6.05E-19 | 2.99E-18 |
| RP5-988G17.1 | ENSG00000224711 | 14.923459 | 3.899510 | 3.46E-32 | 3.56E-31 |
| RP11-10J5.1 | ENSG00000226004 | 14.912421 | 3.898443 | 4.22E-25 | 2.99E-24 |
| RP11-401O9.4 | ENSG00000273388 | 14.775993 | 3.885183 | 6.62E-09 | 1.55E-08 |
| CTB-26E19.1 | ENSG00000245688 | 14.727288 | 3.880420 | 7.67E-45 | 1.42E-43 |
| RP11-496D24.2 | ENSG00000261121 | 14.692099 | 3.876969 | 5.39E-31 | 5.23E-30 |
| RP5-1172A22.1 | ENSG00000233521 | 14.665643 | 3.874368 | 2.21E-25 | 1.60E-24 |
| LINC00942 | ENSG00000249628 | 14.662356 | 3.874045 | 3.67E-17 | 1.60E-16 |
| CDKN2B-AS1 | ENSG00000240498 | 14.624806 | 3.870346 | 3.25E-90 | 2.42E-88 |
| PAQR9-AS1 | ENSG00000241570 | 14.593914 | 3.867295 | 4.07E-32 | 4.18E-31 |
| LINC00588 | ENSG00000215117 | 14.538481 | 3.861805 | 4.62E-06 | 8.51E-06 |
| KCCAT333 | ENSG00000236318 | 14.523398 | 3.860307 | 2.63E-18 | 1.24E-17 |
| RP11-471M2.3 | ENSG00000272046 | 14.511638 | 3.859138 | 2.93E-12 | 9.01E-12 |
| RP11-674N23.4 | ENSG00000272788 | 14.491716 | 3.857157 | 5.16E-38 | 7.14E-37 |
| RP11-153N17.1 | ENSG00000233960 | 14.472257 | 3.855218 | 3.29E-08 | 7.29E-08 |
| EGLN3-AS1 | ENSG00000258897 | 14.384149 | 3.846408 | 6.33E-27 | 5.00E-26 |
| RP11-429E11.2 | ENSG00000225106 | 14.382888 | 3.846281 | 2.13E-13 | 7.20E-13 |
| LINC00158 | ENSG00000185433 | 14.320825 | 3.840043 | 7.93E-40 | 1.20E-38 |
| RP11-479J7.2 | ENSG00000229588 | 14.220312 | 3.829881 | 5.60E-21 | 3.15E-20 |
| RP11-510C10.4 | ENSG00000261213 | 14.120856 | 3.819756 | 2.44E-06 | 4.61E-06 |
| AC110781.3 | ENSG00000176349 | 14.081869 | 3.815767 | 5.52E-12 | 1.67E-11 |
| RP11-180C1.1 | ENSG00000250038 | 14.022841 | 3.809707 | 1.32E-07 | 2.77E-07 |
| RP11-313P18.2 | ENSG00000259241 | 13.978173 | 3.805104 | 2.74E-09 | 6.61E-09 |
| PCSK6-AS1 | ENSG00000259764 | 13.810467 | 3.787690 | 3.39E-21 | 1.93E-20 |
| LINC00704 | ENSG00000231298 | 13.735267 | 3.779813 | 2.22E-25 | 1.61E-24 |
| TBX5-AS1 | ENSG00000255399 | 13.684469 | 3.774468 | 8.72E-13 | 2.79E-12 |
| RP11-398J5.1 | ENSG00000266711 | 13.621613 | 3.767826 | 4.03E-06 | 7.45E-06 |
| AC012613.2 | ENSG00000253406 | 13.602565 | 3.765807 | 1.08E-43 | 1.91E-42 |
| AC008991.1 | ENSG00000267683 | 13.599289 | 3.765459 | 2.69E-16 | 1.11E-15 |
| CTD-2083E4.7 | ENSG00000261434 | 13.596923 | 3.765208 | 2.00E-18 | 9.52E-18 |
| NFE4 | ENSG00000230257 | 13.584591 | 3.763899 | 5.42E-16 | 2.20E-15 |
| RP11-401O9.3 | ENSG00000264067 | 13.511774 | 3.756145 | 4.15E-10 | 1.08E-09 |
| RP11-395B7.2 | ENSG00000274993 | 13.488855 | 3.753696 | 5.74E-29 | 5.07E-28 |
| RP11-445N18.5 | ENSG00000227683 | 13.433639 | 3.747778 | 1.66E-13 | 5.66E-13 |
| AC131056.5 | ENSG00000278097 | 13.430336 | 3.747423 | 9.81E-09 | 2.27E-08 |
| RP11-142A23.1 | ENSG00000253317 | 13.400120 | 3.744174 | 8.82E-54 | 2.33E-52 |
| GS1-600G8.5 | ENSG00000235385 | 13.296508 | 3.732976 | 1.05E-16 | 4.49E-16 |
| RP11-1151B14.1 | ENSG00000276403 | 13.279670 | 3.731147 | 8.90E-26 | 6.61E-25 |
| AP000233.4 | ENSG00000222042 | 13.264180 | 3.729464 | 3.79E-19 | 1.90E-18 |
| RP11-264K23.1 | ENSG00000243574 | 13.253812 | 3.728335 | 2.42E-20 | 1.31E-19 |
| RP11-44K6.2 | ENSG00000253838 | 13.234246 | 3.726204 | 8.77E-29 | 7.67E-28 |
| C17orf77 | ENSG00000182352 | 13.210737 | 3.723639 | 2.64E-10 | 7.00E-10 |
| RP11-1259L22.1 | ENSG00000253698 | 13.197349 | 3.722176 | 3.55E-28 | 3.00E-27 |
| ERVMER61-1 | ENSG00000230426 | 13.115831 | 3.713237 | 4.38E-07 | 8.84E-07 |
| CTB-91J4.1 | ENSG00000276241 | 13.042694 | 3.705170 | 3.46E-29 | 3.09E-28 |
| CTD-2357A8.3 | ENSG00000267123 | 12.965845 | 3.696644 | 3.77E-27 | 3.00E-26 |
| RP11-568J23.8 | ENSG00000275393 | 12.928412 | 3.692473 | 2.66E-29 | 2.39E-28 |
| RP1-60O19.2 | ENSG00000229654 | 12.889702 | 3.688147 | 1.24E-33 | 1.38E-32 |
| HIF1A-AS2 | ENSG00000258667 | 12.776368 | 3.675406 | 4.17E-60 | 1.38E-58 |
| CTD-2532K18.2 | ENSG00000251144 | 12.724332 | 3.669518 | 3.25E-10 | 8.51E-10 |
| RP11-19E11.1 | ENSG00000258910 | 12.556407 | 3.650352 | 1.30E-12 | 4.10E-12 |
| GAS6-AS1 | ENSG00000233695 | 12.510547 | 3.645073 | 2.25E-83 | 1.49E-81 |
| RP11-138I17.1 | ENSG00000248869 | 12.495737 | 3.643364 | 3.99E-13 | 1.32E-12 |
| RP11-815M8.1 | ENSG00000238042 | 12.452105 | 3.638318 | 5.93E-12 | 1.79E-11 |
| RP4-564F22.6 | ENSG00000275285 | 12.397799 | 3.632012 | 7.67E-08 | 1.65E-07 |
| AC011524.1 | ENSG00000267528 | 12.285077 | 3.618835 | 4.00E-16 | 1.64E-15 |
| LINC01405 | ENSG00000185847 | 12.212829 | 3.610326 | 8.26E-13 | 2.65E-12 |
| LINC00686 | ENSG00000237687 | 12.169362 | 3.605182 | 2.50E-05 | 4.31E-05 |
| RP11-407A16.3 | ENSG00000256732 | 12.080090 | 3.594559 | 4.04E-16 | 1.65E-15 |
| RP11-14C10.3 | ENSG00000274139 | 11.960178 | 3.580167 | 1.96E-25 | 1.42E-24 |
| CTD-2527I21.15 | ENSG00000179066 | 11.947715 | 3.578663 | 1.41E-13 | 4.82E-13 |
| CTD-2171N6.1 | ENSG00000267013 | 11.947019 | 3.578579 | 3.72E-26 | 2.84E-25 |
| C2-AS1 | ENSG00000281756 | 11.928161 | 3.576300 | 1.06E-27 | 8.74E-27 |
| RP11-659P15.1 | ENSG00000255146 | 11.855836 | 3.567526 | 9.14E-40 | 1.37E-38 |
| HOTTIP | ENSG00000243766 | 11.778875 | 3.558130 | 3.17E-13 | 1.06E-12 |
| RP4-566L20.1 | ENSG00000279597 | 11.742951 | 3.553723 | 1.72E-09 | 4.23E-09 |
| DGCR9 | ENSG00000273032 | 11.724421 | 3.551445 | 4.27E-79 | 2.43E-77 |
| RP11-356I2.1 | ENSG00000234956 | 11.717059 | 3.550539 | 3.99E-17 | 1.74E-16 |
| CTD-2291D10.3 | ENSG00000269543 | 11.618009 | 3.538291 | 1.03E-12 | 3.28E-12 |
| RP11-366H4.1 | ENSG00000248370 | 11.466826 | 3.519394 | 1.18E-07 | 2.50E-07 |
| RP11-184A2.3 | ENSG00000229672 | 11.464579 | 3.519112 | 1.13E-32 | 1.19E-31 |
| AC145110.1 | ENSG00000253490 | 11.459486 | 3.518470 | 1.66E-33 | 1.83E-32 |
| LINC00944 | ENSG00000256128 | 11.415670 | 3.512944 | 2.61E-43 | 4.55E-42 |
| LINC00881 | ENSG00000241135 | 11.383512 | 3.508874 | 6.97E-33 | 7.39E-32 |
| CTD-2015G9.2 | ENSG00000261175 | 11.324581 | 3.501386 | 2.00E-53 | 5.16E-52 |
| AC005264.2 | ENSG00000267551 | 11.316244 | 3.500323 | 6.05E-61 | 2.06E-59 |
| RP11-543D5.1 | ENSG00000227947 | 11.283135 | 3.496096 | 2.55E-39 | 3.71E-38 |
| LINC00299 | ENSG00000236790 | 11.204444 | 3.485999 | 1.01E-65 | 4.03E-64 |
| RP11-866E20.3 | ENSG00000267462 | 11.189586 | 3.484085 | 2.02E-14 | 7.36E-14 |
| RP11-91J3.3 | ENSG00000250620 | 11.176383 | 3.482381 | 4.96E-16 | 2.01E-15 |
| CRAT8 | ENSG00000256643 | 11.146464 | 3.478514 | 3.22E-11 | 9.23E-11 |
| CDKN2A-AS1 | ENSG00000224854 | 11.118275 | 3.474861 | 8.34E-24 | 5.47E-23 |
| RP5-1195D24.1 | ENSG00000236985 | 11.117572 | 3.474770 | 5.37E-16 | 2.18E-15 |
| RP1-232P20.1 | ENSG00000269985 | 11.103848 | 3.472988 | 6.79E-44 | 1.21E-42 |
| LINC00487 | ENSG00000205837 | 11.038640 | 3.464491 | 1.00E-62 | 3.65E-61 |
| RP11-341G23.4 | ENSG00000257681 | 11.022218 | 3.462343 | 4.28E-47 | 8.74E-46 |
| RP3-495K2.3 | ENSG00000225879 | 10.948777 | 3.452698 | 1.64E-09 | 4.05E-09 |
| LINC00943 | ENSG00000189238 | 10.939160 | 3.451430 | 2.98E-47 | 6.12E-46 |
| RP11-161D15.3 | ENSG00000251216 | 10.914115 | 3.448123 | 4.30E-09 | 1.02E-08 |
| RP11-476K15.1 | ENSG00000266602 | 10.911622 | 3.447794 | 1.10E-11 | 3.24E-11 |
| CTD-2020K17.1 | ENSG00000267121 | 10.885857 | 3.444383 | 8.57E-52 | 2.06E-50 |
| RP11-626H12.2 | ENSG00000254605 | 10.825430 | 3.436352 | 4.76E-51 | 1.11E-49 |
| RP11-221N13.2 | ENSG00000255866 | 10.679844 | 3.416819 | 2.89E-10 | 7.61E-10 |
| RP11-326C3.2 | ENSG00000255026 | 10.660703 | 3.414231 | 2.84E-29 | 2.55E-28 |
| RP11-120K18.2 | ENSG00000260757 | 10.619777 | 3.408682 | 5.12E-25 | 3.60E-24 |
| CTD-2130O13.1 | ENSG00000267761 | 10.606893 | 3.406930 | 5.21E-13 | 1.71E-12 |
| RP11-473A10.2 | ENSG00000236648 | 10.600944 | 3.406121 | 4.30E-07 | 8.69E-07 |
| RP11-598F7.5 | ENSG00000256694 | 10.577725 | 3.402958 | 3.66E-72 | 1.72E-70 |
| RP5-983L19.2 | ENSG00000226954 | 10.537261 | 3.397428 | 3.54E-12 | 1.08E-11 |
| LINC01234 | ENSG00000249550 | 10.440456 | 3.384113 | 8.02E-11 | 2.22E-10 |
| CTD-2616J11.3 | ENSG00000254760 | 10.411804 | 3.380148 | 2.39E-41 | 3.94E-40 |
| LINC01615 | ENSG00000223485 | 10.392048 | 3.377408 | 5.84E-31 | 5.64E-30 |
| RP11-184A2.2 | ENSG00000228353 | 10.302363 | 3.364903 | 9.14E-25 | 6.34E-24 |
| LINC00678 | ENSG00000254934 | 10.232920 | 3.355146 | 9.87E-08 | 2.10E-07 |
| RP11-598F7.3 | ENSG00000256948 | 10.194235 | 3.349682 | 8.75E-32 | 8.78E-31 |
| RP11-66A2.2 | ENSG00000236497 | 10.174249 | 3.346850 | 3.54E-30 | 3.33E-29 |
| KCNMA1-AS1 | ENSG00000236467 | 10.056831 | 3.330104 | 1.32E-45 | 2.54E-44 |
| RP11-191N8.2 | ENSG00000227925 | 10.040625 | 3.327777 | 7.50E-11 | 2.08E-10 |
| RP11-1263C18.1 | ENSG00000273238 | 10.016470 | 3.324302 | 4.72E-11 | 1.33E-10 |
| LINC00838 | ENSG00000261683 | 9.903031 | 3.307870 | 4.45E-18 | 2.07E-17 |
| RP11-549L6.3 | ENSG00000228417 | 9.866716 | 3.302570 | 7.73E-32 | 7.78E-31 |
| LINC01428 | ENSG00000228888 | 9.854056 | 3.300718 | 7.78E-34 | 8.75E-33 |
| CTD-2553C6.1 | ENSG00000267650 | 9.841766 | 3.298917 | 4.65E-33 | 4.99E-32 |
| LINP1 | ENSG00000223784 | 9.840821 | 3.298779 | 9.56E-46 | 1.85E-44 |
| RP11-543G18.1 | ENSG00000259503 | 9.830189 | 3.297219 | 2.67E-20 | 1.44E-19 |
| RP11-291B21.2 | ENSG00000256039 | 9.753150 | 3.285868 | 9.10E-30 | 8.36E-29 |
| RP5-1070A16.1 | ENSG00000226053 | 9.751658 | 3.285647 | 1.20E-07 | 2.53E-07 |
| RP11-661C8.2 | ENSG00000248949 | 9.750022 | 3.285405 | 8.28E-20 | 4.35E-19 |
| LINC00475 | ENSG00000225511 | 9.737633 | 3.283571 | 1.64E-35 | 2.03E-34 |
| RP11-64D24.4 | ENSG00000256195 | 9.702989 | 3.278429 | 2.85E-39 | 4.13E-38 |
| RP11-557C18.4 | ENSG00000259631 | 9.609946 | 3.264528 | 2.82E-23 | 1.79E-22 |
| RP11-331K21.1 | ENSG00000248647 | 9.555851 | 3.256384 | 1.20E-27 | 9.82E-27 |
| CTC-340I23.2 | ENSG00000254163 | 9.500413 | 3.247990 | 2.26E-18 | 1.07E-17 |
| DGCR10 | ENSG00000273164 | 9.491200 | 3.246590 | 3.94E-59 | 1.25E-57 |
| CTD-2501M5.1 | ENSG00000253507 | 9.450826 | 3.240440 | 9.96E-13 | 3.17E-12 |
| RP11-121A14.3 | ENSG00000227200 | 9.433051 | 3.237724 | 2.48E-37 | 3.35E-36 |
| CTC-503J8.4 | ENSG00000267563 | 9.422700 | 3.236140 | 2.90E-13 | 9.70E-13 |
| RP11-774D14.1 | ENSG00000251629 | 9.382074 | 3.229907 | 2.20E-10 | 5.88E-10 |
| CTC-435M10.10 | ENSG00000268987 | 9.322737 | 3.220754 | 2.26E-15 | 8.78E-15 |
| RP11-167B3.2 | ENSG00000274312 | 9.312833 | 3.219220 | 8.64E-18 | 3.95E-17 |
| AC005150.1 | ENSG00000248431 | 9.287173 | 3.215239 | 7.35E-06 | 1.33E-05 |
| AC092484.1 | ENSG00000225107 | 9.271483 | 3.212800 | 2.27E-07 | 4.67E-07 |
| GATM-AS1 | ENSG00000275672 | 9.270835 | 3.212699 | 2.40E-53 | 6.13E-52 |
| RP11-496I9.1 | ENSG00000254815 | 9.235992 | 3.207267 | 4.93E-41 | 7.94E-40 |
| RP11-909N17.2 | ENSG00000253931 | 9.197144 | 3.201186 | 4.45E-14 | 1.58E-13 |
| LINC00705 | ENSG00000225269 | 9.179459 | 3.198409 | 1.14E-15 | 4.51E-15 |
| RP11-744D14.2 | ENSG00000260715 | 9.153285 | 3.194290 | 2.09E-10 | 5.59E-10 |
| CTD-2010I22.2 | ENSG00000248391 | 9.131605 | 3.190868 | 1.20E-10 | 3.27E-10 |
| RP11-145M4.2 | ENSG00000235215 | 9.071769 | 3.181384 | 3.09E-25 | 2.21E-24 |
| LINC00906 | ENSG00000267339 | 9.065229 | 3.180344 | 2.42E-17 | 1.07E-16 |
| C3orf67-AS1 | ENSG00000242428 | 9.062299 | 3.179877 | 1.58E-18 | 7.60E-18 |
| FOXN3-AS2 | ENSG00000259073 | 9.051194 | 3.178108 | 5.35E-13 | 1.75E-12 |
| RP11-567G11.1 | ENSG00000228952 | 9.035743 | 3.175643 | 1.96E-35 | 2.41E-34 |
| NLGN1-AS1 | ENSG00000228213 | 9.002492 | 3.170324 | 1.40E-20 | 7.66E-20 |
| CTD-2023M8.1 | ENSG00000248693 | 8.998762 | 3.169727 | 3.56E-27 | 2.84E-26 |
| RP11-486M23.2 | ENSG00000253182 | 8.997830 | 3.169577 | 1.58E-15 | 6.22E-15 |
| DLEU7-AS1 | ENSG00000237152 | 8.989768 | 3.168284 | 3.14E-43 | 5.45E-42 |
| RP11-322D14.2 | ENSG00000260145 | 8.916396 | 3.156461 | 1.71E-35 | 2.12E-34 |
| AC004988.1 | ENSG00000203446 | 8.915273 | 3.156279 | 3.33E-25 | 2.37E-24 |
| RP11-598F7.6 | ENSG00000256540 | 8.900530 | 3.153891 | 3.44E-61 | 1.18E-59 |
| LINC01163 | ENSG00000280953 | 8.868586 | 3.148704 | 2.95E-22 | 1.77E-21 |
| RP11-70D24.4 | ENSG00000275040 | 8.807786 | 3.138779 | 1.43E-27 | 1.16E-26 |
| RP11-497G19.7 | ENSG00000275898 | 8.785353 | 3.135100 | 1.14E-27 | 9.35E-27 |
| RP11-67M9.1 | ENSG00000254171 | 8.765532 | 3.131842 | 1.07E-10 | 2.94E-10 |
| RP11-84D1.2 | ENSG00000261025 | 8.753800 | 3.129909 | 1.27E-16 | 5.36E-16 |
| TRIM36-IT1 | ENSG00000250472 | 8.727680 | 3.125598 | 1.54E-11 | 4.51E-11 |
| RP11-1399P15.1 | ENSG00000273445 | 8.713892 | 3.123317 | 6.72E-21 | 3.77E-20 |
| CTD-2026K11.6 | ENSG00000203392 | 8.707507 | 3.122260 | 8.78E-64 | 3.28E-62 |
| BPESC1 | ENSG00000232416 | 8.706422 | 3.122080 | 1.34E-11 | 3.95E-11 |
| KB-1043D8.8 | ENSG00000270131 | 8.674028 | 3.116702 | 7.81E-20 | 4.10E-19 |
| RP11-440G9.1 | ENSG00000236366 | 8.666204 | 3.115400 | 9.17E-11 | 2.53E-10 |
| RP11-259N19.1 | ENSG00000272711 | 8.649228 | 3.112571 | 8.31E-66 | 3.33E-64 |
| RP11-146E13.4 | ENSG00000277128 | 8.647586 | 3.112298 | 3.46E-18 | 1.62E-17 |
| LL22NC03-63E9.3 | ENSG00000220891 | 8.644649 | 3.111807 | 1.33E-12 | 4.19E-12 |
| RP5-921G16.2 | ENSG00000241324 | 8.632001 | 3.109695 | 1.62E-14 | 5.96E-14 |
| CTD-2313F11.1 | ENSG00000240535 | 8.629266 | 3.109238 | 1.88E-31 | 1.87E-30 |
| LINC01358 | ENSG00000237352 | 8.601802 | 3.104639 | 4.35E-19 | 2.17E-18 |
| RP11-279O17.1 | ENSG00000261319 | 8.577735 | 3.100597 | 3.80E-11 | 1.08E-10 |
| RP11-314N14.1 | ENSG00000250102 | 8.573555 | 3.099894 | 1.56E-11 | 4.57E-11 |
| RP11-465L10.10 | ENSG00000204044 | 8.566620 | 3.098726 | 4.68E-35 | 5.61E-34 |
| RP11-89M20.2 | ENSG00000253524 | 8.550985 | 3.096091 | 1.36E-06 | 2.63E-06 |
| AC005757.6 | ENSG00000267044 | 8.547845 | 3.095561 | 3.75E-25 | 2.66E-24 |
| RP11-343J3.2 | ENSG00000236154 | 8.542511 | 3.094660 | 8.82E-27 | 6.91E-26 |
| RP11-255H23.4 | ENSG00000267924 | 8.530842 | 3.092688 | 3.60E-10 | 9.40E-10 |
| LINC01304 | ENSG00000237401 | 8.506175 | 3.088510 | 2.18E-11 | 6.34E-11 |
| RP5-896L10.1 | ENSG00000232825 | 8.459795 | 3.080623 | 1.34E-13 | 4.59E-13 |
| RP11-64B16.4 | ENSG00000256609 | 8.452204 | 3.079328 | 1.28E-35 | 1.60E-34 |
| CTB-186G2.1 | ENSG00000267291 | 8.405370 | 3.071311 | 9.73E-22 | 5.71E-21 |
| RP11-540K16.1 | ENSG00000237292 | 8.403278 | 3.070952 | 9.73E-27 | 7.60E-26 |
| RP11-181E10.3 | ENSG00000271590 | 8.396362 | 3.069764 | 5.18E-54 | 1.39E-52 |
| RP11-366F6.2 | ENSG00000229967 | 8.383819 | 3.067608 | 2.07E-05 | 3.60E-05 |
| RP11-643C9.2 | ENSG00000260679 | 8.353581 | 3.062395 | 4.80E-33 | 5.14E-32 |
| RP11-785G17.1 | ENSG00000270540 | 8.330547 | 3.058411 | 3.16E-28 | 2.68E-27 |
| AC002331.1 | ENSG00000236481 | 8.302216 | 3.053496 | 3.42E-20 | 1.83E-19 |
| MGC39584 | ENSG00000280081 | 8.300388 | 3.053179 | 1.64E-07 | 3.43E-07 |
| LINC01281 | ENSG00000235304 | 8.300385 | 3.053178 | 9.06E-17 | 3.86E-16 |
| RP11-218E20.5 | ENSG00000258745 | 8.295731 | 3.052369 | 3.57E-34 | 4.10E-33 |
| RP11-142M10.2 | ENSG00000229751 | 8.281276 | 3.049853 | 3.81E-25 | 2.70E-24 |
| LINC01280 | ENSG00000224391 | 8.266001 | 3.047190 | 5.67E-15 | 2.15E-14 |
| RP11-15I20.1 | ENSG00000272814 | 8.211510 | 3.037647 | 1.62E-21 | 9.43E-21 |
| RP11-12M5.3 | ENSG00000229407 | 8.165180 | 3.029485 | 3.84E-36 | 4.94E-35 |
| CTD-2278I10.1 | ENSG00000269350 | 8.155090 | 3.027701 | 1.30E-29 | 1.19E-28 |
| AC006262.6 | ENSG00000268460 | 8.146587 | 3.026196 | 3.01E-20 | 1.61E-19 |
| AC067959.1 | ENSG00000233005 | 8.103924 | 3.018621 | 1.17E-15 | 4.65E-15 |
| CDRT7 | ENSG00000259944 | 8.102626 | 3.018389 | 1.05E-09 | 2.64E-09 |
| WASF3-AS1 | ENSG00000237001 | 8.099824 | 3.017891 | 4.65E-11 | 1.31E-10 |
| RP11-489G11.3 | ENSG00000261646 | 8.067793 | 3.012174 | 3.04E-41 | 4.96E-40 |
| LINC01551 | ENSG00000186960 | 8.052353 | 3.009410 | 1.32E-10 | 3.58E-10 |
| RP11-84D1.1 | ENSG00000229162 | 8.026886 | 3.004840 | 8.08E-34 | 9.08E-33 |
| RP11-1018N14.5 | ENSG00000267452 | 7.972709 | 2.995070 | 3.42E-29 | 3.07E-28 |
| LINC01033 | ENSG00000249069 | 7.910733 | 2.983811 | 2.68E-25 | 1.92E-24 |
| KB-1460A1.1 | ENSG00000253395 | 7.906026 | 2.982953 | 7.87E-48 | 1.65E-46 |
| CTC-806A22.1 | ENSG00000250842 | 7.903181 | 2.982433 | 6.24E-34 | 7.06E-33 |
| RP11-172F10.1 | ENSG00000266268 | 7.887605 | 2.979587 | 1.05E-12 | 3.33E-12 |
| RP11-556E13.1 | ENSG00000228651 | 7.882738 | 2.978697 | 9.12E-31 | 8.75E-30 |
| CTD-2354A18.1 | ENSG00000261780 | 7.881768 | 2.978519 | 9.78E-15 | 3.64E-14 |
| RP11-767N15.1 | ENSG00000249171 | 7.776646 | 2.959148 | 1.43E-07 | 3.00E-07 |
| CTD-2527I21.9 | ENSG00000267874 | 7.776146 | 2.959055 | 9.02E-31 | 8.67E-30 |
| RP11-122C21.1 | ENSG00000253901 | 7.758974 | 2.955866 | 3.11E-10 | 8.16E-10 |
| AC073257.2 | ENSG00000237614 | 7.749208 | 2.954049 | 4.74E-41 | 7.65E-40 |
| AC004906.3 | ENSG00000237286 | 7.744689 | 2.953207 | 1.08E-21 | 6.34E-21 |
| MIR142 | ENSG00000265206 | 7.738944 | 2.952137 | 3.52E-37 | 4.73E-36 |
| LL22NC03-N14H11.1 | ENSG00000272872 | 7.725345 | 2.949599 | 1.24E-28 | 1.07E-27 |
| RP11-807H17.1 | ENSG00000227869 | 7.719077 | 2.948428 | 2.60E-28 | 2.21E-27 |
| AC007326.10 | ENSG00000280418 | 7.713350 | 2.947358 | 9.57E-15 | 3.57E-14 |
| LINC00421 | ENSG00000236834 | 7.711843 | 2.947076 | 3.06E-05 | 5.24E-05 |
| MIR210HG | ENSG00000247095 | 7.708158 | 2.946386 | 8.30E-89 | 5.98E-87 |
| LINC00928 | ENSG00000259218 | 7.693612 | 2.943661 | 2.31E-12 | 7.16E-12 |
| RP3-434P1.6 | ENSG00000228620 | 7.674506 | 2.940074 | 3.75E-23 | 2.36E-22 |
| RP11-2N1.3 | ENSG00000273664 | 7.664825 | 2.938253 | 5.00E-04 | 7.58E-04 |
| ELOVL2-AS1 | ENSG00000230314 | 7.664013 | 2.938100 | 3.99E-19 | 1.99E-18 |
| RP3-522D1.1 | ENSG00000224167 | 7.628314 | 2.931364 | 1.46E-36 | 1.90E-35 |
| RP11-663P9.1 | ENSG00000250863 | 7.628300 | 2.931362 | 2.91E-29 | 2.61E-28 |
| CTD-2562J17.2 | ENSG00000255395 | 7.605883 | 2.927116 | 7.42E-12 | 2.23E-11 |
| AC000067.1 | ENSG00000225007 | 7.604598 | 2.926872 | 2.10E-33 | 2.31E-32 |
| RP11-772C9.1 | ENSG00000249198 | 7.603896 | 2.926739 | 8.68E-11 | 2.40E-10 |
| AF064858.7 | ENSG00000232837 | 7.598147 | 2.925648 | 5.62E-22 | 3.33E-21 |
| AL132709.1 | ENSG00000230805 | 7.591450 | 2.924375 | 8.73E-06 | 1.57E-05 |
| RP11-138H10.2 | ENSG00000259761 | 7.573130 | 2.920890 | 3.74E-06 | 6.95E-06 |
| RP11-404O13.1 | ENSG00000231666 | 7.559555 | 2.918301 | 6.55E-42 | 1.10E-40 |
| AP000797.3 | ENSG00000256717 | 7.537198 | 2.914028 | 1.00E-14 | 3.73E-14 |
| LINC01151 | ENSG00000253819 | 7.524568 | 2.911609 | 1.14E-08 | 2.61E-08 |
| RP4-719C8.1 | ENSG00000238194 | 7.507801 | 2.908391 | 1.93E-08 | 4.36E-08 |
| RP11-805I24.1 | ENSG00000261177 | 7.506043 | 2.908052 | 2.47E-12 | 7.64E-12 |
| RP3-393E18.2 | ENSG00000237927 | 7.483640 | 2.903740 | 8.45E-36 | 1.07E-34 |
| RP11-145A3.1 | ENSG00000227496 | 7.471007 | 2.901303 | 1.75E-18 | 8.37E-18 |
| RP11-94A24.1 | ENSG00000255364 | 7.458476 | 2.898881 | 9.39E-08 | 2.00E-07 |
| AC133644.2 | ENSG00000280721 | 7.458164 | 2.898821 | 1.60E-55 | 4.40E-54 |
| AC131056.3 | ENSG00000274767 | 7.450407 | 2.897319 | 2.21E-22 | 1.34E-21 |
| CH507-513H4.3 | ENSG00000281181 | 7.418296 | 2.891088 | 2.34E-05 | 4.04E-05 |
| RP11-167B3.3 | ENSG00000277987 | 7.416339 | 2.890707 | 1.92E-15 | 7.52E-15 |
| RP5-1028K7.2 | ENSG00000266088 | 7.414826 | 2.890413 | 7.49E-36 | 9.51E-35 |
| AC011752.1 | ENSG00000231204 | 7.365435 | 2.880771 | 3.21E-13 | 1.07E-12 |
| RP11-495P10.5 | ENSG00000238107 | 7.351767 | 2.878091 | 8.23E-08 | 1.76E-07 |
| RP5-1092A11.5 | ENSG00000227589 | 7.338436 | 2.875473 | 8.45E-12 | 2.52E-11 |
| LINC01192 | ENSG00000241369 | 7.301432 | 2.868179 | 2.45E-10 | 6.51E-10 |
| RP11-489O18.1 | ENSG00000253988 | 7.292038 | 2.866322 | 7.65E-49 | 1.65E-47 |
| LINC00896 | ENSG00000236499 | 7.284654 | 2.864861 | 5.66E-20 | 3.00E-19 |
| RP11-1151B14.5 | ENSG00000274354 | 7.282375 | 2.864409 | 6.05E-33 | 6.46E-32 |
| MIR155HG | ENSG00000234883 | 7.281222 | 2.864181 | 2.20E-45 | 4.20E-44 |
| AC009264.1 | ENSG00000234352 | 7.235235 | 2.855040 | 7.80E-11 | 2.17E-10 |
| RP11-674P19.2 | ENSG00000264705 | 7.221738 | 2.852346 | 1.59E-37 | 2.16E-36 |
| RP11-445N18.7 | ENSG00000243349 | 7.189841 | 2.845960 | 5.90E-11 | 1.66E-10 |
| RP1-79C4.4 | ENSG00000271811 | 7.189289 | 2.845849 | 2.20E-19 | 1.12E-18 |
| CTB-140J7.2 | ENSG00000253852 | 7.174023 | 2.842782 | 1.26E-27 | 1.03E-26 |
| RP11-1151B14.4 | ENSG00000267257 | 7.161613 | 2.840285 | 5.72E-36 | 7.30E-35 |
| RP1-142L7.9 | ENSG00000270661 | 7.159537 | 2.839866 | 6.42E-24 | 4.24E-23 |
| RP11-856M7.2 | ENSG00000264843 | 7.154495 | 2.838850 | 5.36E-19 | 2.66E-18 |
| RP11-804N13.1 | ENSG00000253766 | 7.136669 | 2.835251 | 1.71E-12 | 5.35E-12 |
| AC006372.6 | ENSG00000233191 | 7.114733 | 2.830810 | 2.44E-10 | 6.50E-10 |
| RP11-528G1.2 | ENSG00000234425 | 7.109967 | 2.829843 | 1.96E-29 | 1.78E-28 |
| RP11-493L12.5 | ENSG00000257924 | 7.100004 | 2.827820 | 3.57E-17 | 1.56E-16 |
| RP11-115C10.1 | ENSG00000254607 | 7.099826 | 2.827784 | 3.49E-08 | 7.70E-08 |
| C8orf49 | ENSG00000255394 | 7.096830 | 2.827175 | 1.56E-06 | 3.01E-06 |
| RP5-984P4.6 | ENSG00000278041 | 7.087367 | 2.825250 | 3.51E-10 | 9.18E-10 |
| RP11-350G24.1 | ENSG00000258007 | 7.084912 | 2.824750 | 1.96E-09 | 4.79E-09 |
| CTC-241F20.4 | ENSG00000268186 | 7.076857 | 2.823109 | 1.65E-10 | 4.45E-10 |
| RP4-671O14.7 | ENSG00000280011 | 7.036841 | 2.814928 | 3.81E-42 | 6.44E-41 |
| RP11-1191J2.2 | ENSG00000242686 | 7.004339 | 2.808249 | 2.52E-23 | 1.61E-22 |
| RP11-414H23.2 | ENSG00000212930 | 6.999173 | 2.807184 | 6.77E-13 | 2.19E-12 |
| RP11-680B3.2 | ENSG00000240521 | 6.994182 | 2.806155 | 5.27E-10 | 1.36E-09 |
| CTD-2309O5.3 | ENSG00000261848 | 6.989657 | 2.805222 | 1.85E-18 | 8.81E-18 |
| RP13-379O24.2 | ENSG00000260542 | 6.965152 | 2.800155 | 4.61E-09 | 1.09E-08 |
| PICSAR | ENSG00000275874 | 6.963178 | 2.799746 | 9.85E-15 | 3.66E-14 |
| AC008088.4 | ENSG00000260907 | 6.948457 | 2.796693 | 3.65E-15 | 1.40E-14 |
| RP6-24A23.7 | ENSG00000261409 | 6.932471 | 2.793370 | 1.01E-04 | 1.64E-04 |
| RP11-193H5.1 | ENSG00000237250 | 6.928964 | 2.792640 | 1.81E-05 | 3.16E-05 |
| RP11-1137G4.3 | ENSG00000268845 | 6.911340 | 2.788965 | 3.73E-14 | 1.33E-13 |
| RP11-344P13.4 | ENSG00000228826 | 6.909163 | 2.788511 | 1.01E-13 | 3.50E-13 |
| CTD-2223O18.1 | ENSG00000258748 | 6.890783 | 2.784668 | 9.16E-21 | 5.07E-20 |
| LINC00544 | ENSG00000122043 | 6.884002 | 2.783248 | 2.01E-22 | 1.22E-21 |
| RP1-207H1.3 | ENSG00000231150 | 6.872931 | 2.780926 | 4.43E-12 | 1.35E-11 |
| RP11-19J5.2 | ENSG00000259309 | 6.865947 | 2.779459 | 2.23E-14 | 8.08E-14 |
| AC093627.7 | ENSG00000232325 | 6.849074 | 2.775909 | 2.23E-06 | 4.23E-06 |
| AF064858.11 | ENSG00000237721 | 6.822571 | 2.770315 | 1.43E-20 | 7.82E-20 |
| RP11-284H18.1 | ENSG00000253574 | 6.818918 | 2.769543 | 4.32E-16 | 1.76E-15 |
| RP11-336A10.5 | ENSG00000231483 | 6.806912 | 2.767000 | 9.96E-46 | 1.92E-44 |
| LINC00173 | ENSG00000196668 | 6.791817 | 2.763797 | 1.07E-40 | 1.69E-39 |
| LINC00922 | ENSG00000261742 | 6.772428 | 2.759673 | 5.91E-07 | 1.18E-06 |
| RP11-445P17.3 | ENSG00000231039 | 6.770449 | 2.759252 | 1.79E-15 | 7.00E-15 |
| RP11-352G9.1 | ENSG00000273009 | 6.769224 | 2.758990 | 2.04E-36 | 2.66E-35 |
| RP11-569G13.3 | ENSG00000275465 | 6.744191 | 2.753645 | 6.82E-13 | 2.20E-12 |
| CTD-2252P21.1 | ENSG00000257226 | 6.740714 | 2.752901 | 1.13E-28 | 9.83E-28 |
| AC020956.3 | ENSG00000278239 | 6.738283 | 2.752381 | 2.07E-10 | 5.55E-10 |
| LINC00836 | ENSG00000280809 | 6.730285 | 2.750668 | 1.21E-06 | 2.34E-06 |
| RP11-107I14.4 | ENSG00000229649 | 6.723479 | 2.749208 | 1.00E-09 | 2.51E-09 |
| LINC01501 | ENSG00000229613 | 6.713204 | 2.747002 | 8.37E-20 | 4.39E-19 |
| AC006227.1 | ENSG00000229457 | 6.700544 | 2.744278 | 4.24E-14 | 1.51E-13 |
| RP11-275I4.2 | ENSG00000259747 | 6.684829 | 2.740891 | 4.56E-14 | 1.62E-13 |
| RP11-340F14.6 | ENSG00000274029 | 6.684005 | 2.740713 | 5.22E-64 | 1.96E-62 |
| KCCAT198 | ENSG00000257642 | 6.679918 | 2.739830 | 1.36E-33 | 1.51E-32 |
| RP11-50B3.2 | ENSG00000254833 | 6.664170 | 2.736425 | 9.70E-28 | 7.98E-27 |
| LINC01305 | ENSG00000231453 | 6.663234 | 2.736222 | 1.09E-11 | 3.24E-11 |
| DARS-AS1 | ENSG00000231890 | 6.661265 | 2.735796 | 2.24E-81 | 1.41E-79 |
| RP11-616M22.3 | ENSG00000261294 | 6.657777 | 2.735041 | 7.27E-10 | 1.86E-09 |
| LINC01505 | ENSG00000234323 | 6.655109 | 2.734462 | 5.00E-11 | 1.41E-10 |
| LZTS1-AS1 | ENSG00000253733 | 6.626596 | 2.728268 | 5.10E-13 | 1.67E-12 |
| LINC00298 | ENSG00000235665 | 6.626250 | 2.728193 | 6.64E-23 | 4.14E-22 |
| RP11-543H12.1 | ENSG00000258026 | 6.625778 | 2.728090 | 1.90E-07 | 3.96E-07 |
| RP4-539M6.22 | ENSG00000273428 | 6.622763 | 2.727433 | 9.05E-35 | 1.07E-33 |
| RP11-20J15.3 | ENSG00000229116 | 6.615483 | 2.725846 | 6.67E-19 | 3.28E-18 |
| LINC01546 | ENSG00000228459 | 6.603997 | 2.723340 | 1.74E-34 | 2.03E-33 |
| SLC16A1-AS1 | ENSG00000226419 | 6.599411 | 2.722337 | 3.51E-108 | 3.76E-106 |
| RP11-455F5.5 | ENSG00000261416 | 6.593184 | 2.720975 | 2.96E-38 | 4.13E-37 |
| CTA-833B7.2 | ENSG00000183822 | 6.581193 | 2.718349 | 1.31E-17 | 5.92E-17 |
| AC012593.1 | ENSG00000226994 | 6.569433 | 2.715769 | 5.21E-27 | 4.13E-26 |
| RP5-1011O1.3 | ENSG00000236069 | 6.551776 | 2.711886 | 1.20E-06 | 2.33E-06 |
| RP11-319E12.2 | ENSG00000251459 | 6.550576 | 2.711622 | 2.86E-24 | 1.92E-23 |
| AC073218.3 | ENSG00000232153 | 6.535809 | 2.708366 | 2.05E-46 | 4.10E-45 |
| CTB-33O18.1 | ENSG00000253768 | 6.528569 | 2.706767 | 3.51E-09 | 8.40E-09 |
| AP001604.3 | ENSG00000231236 | 6.520875 | 2.705066 | 1.61E-20 | 8.78E-20 |
| LINC00524 | ENSG00000259023 | 6.516358 | 2.704066 | 3.68E-07 | 7.46E-07 |
| RP4-644L1.2 | ENSG00000229771 | 6.485033 | 2.697114 | 3.23E-23 | 2.05E-22 |
| RP11-35J23.1 | ENSG00000229418 | 6.480262 | 2.696052 | 5.61E-06 | 1.02E-05 |
| RP11-3P22.2 | ENSG00000225718 | 6.462682 | 2.692133 | 2.75E-14 | 9.90E-14 |
| RP1-142L7.5 | ENSG00000237234 | 6.456361 | 2.690721 | 3.08E-41 | 5.00E-40 |
| PSORS1C3 | ENSG00000204528 | 6.449373 | 2.689159 | 6.37E-36 | 8.10E-35 |
| RP11-1149M10.2 | ENSG00000253214 | 6.429496 | 2.684706 | 2.23E-26 | 1.71E-25 |
| RP11-332J15.3 | ENSG00000251365 | 6.397492 | 2.677506 | 9.68E-33 | 1.02E-31 |
| RP11-958J22.1 | ENSG00000254746 | 6.392359 | 2.676348 | 1.29E-07 | 2.71E-07 |
| RP11-327I22.5 | ENSG00000278849 | 6.391660 | 2.676191 | 1.17E-08 | 2.69E-08 |
| RP11-196G11.2 | ENSG00000260911 | 6.379976 | 2.673551 | 2.12E-53 | 5.44E-52 |
| AC079767.4 | ENSG00000224137 | 6.378217 | 2.673153 | 1.88E-24 | 1.28E-23 |
| AC084117.3 | ENSG00000256006 | 6.369979 | 2.671289 | 1.96E-39 | 2.89E-38 |
| LINC00313 | ENSG00000185186 | 6.368683 | 2.670995 | 4.35E-16 | 1.77E-15 |
| DPP9-AS1 | ENSG00000205790 | 6.362023 | 2.669486 | 1.73E-56 | 5.10E-55 |
| AP002954.4 | ENSG00000255422 | 6.359069 | 2.668816 | 2.14E-27 | 1.73E-26 |
| AC156455.1 | ENSG00000256546 | 6.355240 | 2.667947 | 1.80E-44 | 3.29E-43 |
| CTD-2330K9.2 | ENSG00000230698 | 6.354452 | 2.667768 | 1.17E-09 | 2.91E-09 |
| MNX1-AS1 | ENSG00000243479 | 6.351743 | 2.667153 | 1.66E-09 | 4.09E-09 |
| LINC01587 | ENSG00000082929 | 6.347441 | 2.666175 | 9.51E-21 | 5.26E-20 |
| RP11-264E20.2 | ENSG00000270160 | 6.342568 | 2.665067 | 3.03E-28 | 2.56E-27 |
| LINC01271 | ENSG00000233077 | 6.341278 | 2.664774 | 6.30E-36 | 8.02E-35 |
| LINC01235 | ENSG00000270547 | 6.331036 | 2.662442 | 2.63E-22 | 1.58E-21 |
| PRR7-AS1 | ENSG00000246334 | 6.330564 | 2.662334 | 3.46E-46 | 6.81E-45 |
| CTD-2265O21.3 | ENSG00000267424 | 6.324373 | 2.660922 | 4.51E-07 | 9.08E-07 |
| RP11-284F21.9 | ENSG00000272068 | 6.295325 | 2.654281 | 6.69E-33 | 7.12E-32 |
| RP11-739N10.1 | ENSG00000264151 | 6.265722 | 2.647481 | 7.76E-10 | 1.97E-09 |
| SNHG12 | ENSG00000197989 | 6.265245 | 2.647371 | 1.08E-64 | 4.18E-63 |
| RP11-691H4.4 | ENSG00000267694 | 6.256469 | 2.645349 | 1.50E-15 | 5.91E-15 |
| AC019064.1 | ENSG00000237320 | 6.252202 | 2.644365 | 3.75E-13 | 1.24E-12 |
| RP11-302F12.10 | ENSG00000250541 | 6.250558 | 2.643985 | 1.45E-20 | 7.93E-20 |
| RP11-229P13.15 | ENSG00000236394 | 6.249311 | 2.643697 | 1.87E-14 | 6.84E-14 |
| AC005522.7 | ENSG00000225703 | 6.247322 | 2.643238 | 6.85E-13 | 2.21E-12 |
| AC093642.6 | ENSG00000232002 | 6.242213 | 2.642058 | 1.98E-11 | 5.76E-11 |
| RP11-67C2.2 | ENSG00000231964 | 6.236418 | 2.640718 | 1.67E-33 | 1.84E-32 |
| RP11-636O21.1 | ENSG00000267413 | 6.233780 | 2.640107 | 8.79E-15 | 3.29E-14 |
| RP4-647J21.1 | ENSG00000260997 | 6.221952 | 2.637367 | 8.31E-35 | 9.89E-34 |
| RP11-751H17.1 | ENSG00000265844 | 6.208883 | 2.634334 | 4.99E-07 | 1.00E-06 |
| RP11-861E21.1 | ENSG00000267108 | 6.200866 | 2.632470 | 2.00E-23 | 1.28E-22 |
| RP11-121A14.2 | ENSG00000235204 | 6.191665 | 2.630327 | 1.18E-51 | 2.82E-50 |
| RP11-3J1.1 | ENSG00000248238 | 6.189863 | 2.629908 | 2.07E-08 | 4.65E-08 |
| RP11-38J22.3 | ENSG00000226780 | 6.182352 | 2.628156 | 4.43E-32 | 4.54E-31 |
| RP11-217E22.5 | ENSG00000243486 | 6.174211 | 2.626255 | 5.23E-06 | 9.57E-06 |
| AC074366.3 | ENSG00000232518 | 6.162122 | 2.623427 | 3.45E-17 | 1.51E-16 |
| AC078883.3 | ENSG00000232788 | 6.143664 | 2.619099 | 2.07E-54 | 5.59E-53 |
| RP11-299G20.2 | ENSG00000259172 | 6.141785 | 2.618658 | 5.65E-37 | 7.49E-36 |
| RP11-88H10.2 | ENSG00000244358 | 6.127131 | 2.615212 | 1.10E-10 | 3.01E-10 |
| LAMA5-AS1 | ENSG00000228812 | 6.106831 | 2.610424 | 3.27E-30 | 3.08E-29 |
| AC141930.2 | ENSG00000231482 | 6.100263 | 2.608871 | 1.49E-12 | 4.69E-12 |
| AC133785.1 | ENSG00000233221 | 6.093607 | 2.607297 | 1.63E-07 | 3.42E-07 |
| AC012668.2 | ENSG00000237525 | 6.090147 | 2.606477 | 2.46E-13 | 8.26E-13 |
| RP11-166B2.7 | ENSG00000260488 | 6.082316 | 2.604621 | 1.41E-17 | 6.35E-17 |
| MTUS2-AS1 | ENSG00000179141 | 6.080727 | 2.604244 | 7.61E-12 | 2.28E-11 |
| AC073130.1 | ENSG00000237870 | 6.080666 | 2.604229 | 3.79E-22 | 2.26E-21 |
| CTD-3195I5.4 | ENSG00000262358 | 6.053630 | 2.597800 | 1.09E-12 | 3.46E-12 |
| AC109826.1 | ENSG00000226791 | 6.049480 | 2.596811 | 5.80E-54 | 1.54E-52 |
| RP3-438O4.4 | ENSG00000225676 | 6.027858 | 2.591645 | 1.44E-10 | 3.91E-10 |
| AC112715.2 | ENSG00000224132 | 6.025734 | 2.591137 | 9.12E-13 | 2.92E-12 |
| AC124944.3 | ENSG00000226155 | 6.015962 | 2.588795 | 5.05E-32 | 5.14E-31 |
| RP11-145H9.3 | ENSG00000228718 | 6.015090 | 2.588586 | 4.28E-34 | 4.89E-33 |
| HULC | ENSG00000251164 | 6.012965 | 2.588076 | 3.83E-09 | 9.14E-09 |
| RP11-1C1.4 | ENSG00000249396 | 6.009196 | 2.587172 | 2.69E-10 | 7.13E-10 |
| LINC01614 | ENSG00000230838 | 6.001455 | 2.585312 | 5.20E-22 | 3.09E-21 |
| RP11-565A3.2 | ENSG00000250590 | 5.999059 | 2.584736 | 1.36E-08 | 3.12E-08 |
| RP4-539M6.14 | ENSG00000181123 | 5.996424 | 2.584102 | 1.04E-13 | 3.60E-13 |
| RP11-247A12.7 | ENSG00000268707 | 5.995632 | 2.583912 | 9.91E-25 | 6.86E-24 |
| LINC01206 | ENSG00000242512 | 5.995089 | 2.583781 | 8.23E-12 | 2.46E-11 |
| AC096669.1 | ENSG00000225588 | 5.992352 | 2.583122 | 5.79E-14 | 2.04E-13 |
| FLJ35934 | ENSG00000220161 | 5.988620 | 2.582223 | 1.33E-35 | 1.66E-34 |
| AC092657.2 | ENSG00000237760 | 5.986932 | 2.581817 | 2.87E-08 | 6.39E-08 |
| RP11-495P10.7 | ENSG00000231196 | 5.984835 | 2.581311 | 1.50E-07 | 3.15E-07 |
| C12orf77 | ENSG00000226397 | 5.983227 | 2.580924 | 1.88E-07 | 3.92E-07 |
| RP11-452C13.1 | ENSG00000272839 | 5.980489 | 2.580264 | 2.51E-38 | 3.52E-37 |
| XXbac-BPG170G13.32 | ENSG00000272236 | 5.961077 | 2.575573 | 2.22E-25 | 1.61E-24 |
| AC007278.3 | ENSG00000234389 | 5.950311 | 2.572965 | 4.25E-31 | 4.13E-30 |
| AC064853.2 | ENSG00000236116 | 5.947724 | 2.572338 | 1.21E-05 | 2.14E-05 |
| AC097713.4 | ENSG00000231682 | 5.932476 | 2.568634 | 6.35E-18 | 2.93E-17 |
| RP11-798M19.6 | ENSG00000272870 | 5.920693 | 2.565766 | 9.53E-81 | 5.83E-79 |
| RP5-1120P11.1 | ENSG00000237686 | 5.903746 | 2.561631 | 2.67E-59 | 8.52E-58 |
| RP11-750H9.7 | ENSG00000270072 | 5.883403 | 2.556651 | 6.66E-14 | 2.33E-13 |
| AP001257.1 | ENSG00000254952 | 5.882507 | 2.556431 | 1.11E-22 | 6.84E-22 |
| AC068196.1 | ENSG00000238171 | 5.876028 | 2.554841 | 1.12E-24 | 7.74E-24 |
| LINC01152 | ENSG00000256124 | 5.875667 | 2.554753 | 3.18E-27 | 2.54E-26 |
| RP5-943J3.1 | ENSG00000231613 | 5.863126 | 2.551670 | 1.70E-30 | 1.61E-29 |
| RP11-290F5.1 | ENSG00000249096 | 5.862335 | 2.551475 | 1.49E-20 | 8.12E-20 |
| RP11-16C1.2 | ENSG00000264853 | 5.860509 | 2.551026 | 1.22E-17 | 5.52E-17 |
| CTD-2527I21.14 | ENSG00000271032 | 5.860063 | 2.550916 | 8.26E-41 | 1.32E-39 |
| INHBA-AS1 | ENSG00000224116 | 5.849736 | 2.548372 | 8.52E-20 | 4.47E-19 |
| CTD-2228K2.7 | ENSG00000225138 | 5.848078 | 2.547963 | 2.10E-26 | 1.61E-25 |
| RP11-284F21.7 | ENSG00000229953 | 5.844239 | 2.547015 | 2.68E-41 | 4.39E-40 |
| DUXAP8 | ENSG00000206195 | 5.832137 | 2.544025 | 2.58E-28 | 2.20E-27 |
| LINC00971 | ENSG00000242641 | 5.823097 | 2.541787 | 2.70E-09 | 6.52E-09 |
| CTD-2616J11.2 | ENSG00000255441 | 5.816537 | 2.540161 | 3.22E-23 | 2.04E-22 |
| LINC01630 | ENSG00000227115 | 5.813498 | 2.539407 | 4.95E-10 | 1.28E-09 |
| CH507-513H4.4 | ENSG00000280614 | 5.808963 | 2.538281 | 9.89E-05 | 1.60E-04 |
| RP11-617F23.2 | ENSG00000273691 | 5.808585 | 2.538187 | 5.10E-19 | 2.54E-18 |
| AC069394.1 | ENSG00000224231 | 5.808345 | 2.538127 | 1.65E-14 | 6.03E-14 |
| AC130469.2 | ENSG00000267986 | 5.806575 | 2.537687 | 1.97E-24 | 1.33E-23 |
| RP11-260O18.1 | ENSG00000239440 | 5.801955 | 2.536539 | 5.77E-07 | 1.15E-06 |
| KCNK4-TEX40 | ENSG00000257069 | 5.800989 | 2.536299 | 1.17E-10 | 3.20E-10 |
| OR7E47P | ENSG00000272724 | 5.796772 | 2.535250 | 6.65E-27 | 5.24E-26 |
| LINC01094 | ENSG00000251442 | 5.792551 | 2.534199 | 4.94E-44 | 8.87E-43 |
| RP11-274H2.5 | ENSG00000261051 | 5.792496 | 2.534185 | 5.96E-44 | 1.07E-42 |
| RP11-283G6.4 | ENSG00000256234 | 5.788288 | 2.533137 | 2.87E-31 | 2.81E-30 |
| AC064834.3 | ENSG00000234919 | 5.787738 | 2.533000 | 2.35E-17 | 1.04E-16 |
| LINC00707 | ENSG00000238266 | 5.785250 | 2.532379 | 3.69E-24 | 2.46E-23 |
| LINC01559 | ENSG00000180861 | 5.767925 | 2.528052 | 4.31E-08 | 9.43E-08 |
| MIR122 | ENSG00000267391 | 5.767212 | 2.527874 | 6.30E-38 | 8.69E-37 |
| RP11-1094M14.5 | ENSG00000267074 | 5.756251 | 2.525130 | 1.68E-40 | 2.64E-39 |
| RP11-57A1.1 | ENSG00000263680 | 5.754667 | 2.524732 | 6.86E-21 | 3.84E-20 |
| RP11-428C19.4 | ENSG00000255308 | 5.752735 | 2.524248 | 9.51E-11 | 2.61E-10 |
| RP11-114H24.7 | ENSG00000261244 | 5.752364 | 2.524155 | 3.86E-14 | 1.38E-13 |
| RP11-348J24.2 | ENSG00000250049 | 5.744147 | 2.522093 | 6.09E-20 | 3.22E-19 |
| RP11-58G13.1 | ENSG00000267039 | 5.744122 | 2.522086 | 1.32E-07 | 2.77E-07 |
| RP11-115D19.1 | ENSG00000251095 | 5.739900 | 2.521026 | 2.68E-09 | 6.47E-09 |
| RP11-115D19.2 | ENSG00000276542 | 5.733632 | 2.519449 | 4.16E-07 | 8.41E-07 |
| RP1-205F14P.1 | ENSG00000280445 | 5.730027 | 2.518542 | 3.20E-11 | 9.19E-11 |
| CTD-3064M3.7 | ENSG00000271959 | 5.725956 | 2.517517 | 3.83E-26 | 2.92E-25 |
| XXbac-B476C20.13 | ENSG00000234913 | 5.716691 | 2.515180 | 5.11E-25 | 3.60E-24 |
| RP11-1C1.6 | ENSG00000249166 | 5.713402 | 2.514350 | 5.15E-09 | 1.22E-08 |
| CTA-414D7.1 | ENSG00000279175 | 5.686841 | 2.507628 | 1.29E-20 | 7.07E-20 |
| RP11-332J15.4 | ENSG00000272071 | 5.663299 | 2.501643 | 4.83E-19 | 2.40E-18 |
| RP11-608O21.1 | ENSG00000248515 | 5.655147 | 2.499564 | 2.37E-05 | 4.09E-05 |
| TMEM92-AS1 | ENSG00000251179 | 5.653792 | 2.499219 | 2.36E-18 | 1.12E-17 |
| RP4-640H8.2 | ENSG00000204117 | 5.652303 | 2.498839 | 1.25E-09 | 3.11E-09 |
| AF064858.8 | ENSG00000235888 | 5.647284 | 2.497557 | 8.12E-24 | 5.33E-23 |
| RP11-178L8.5 | ENSG00000261697 | 5.635562 | 2.494560 | 1.84E-09 | 4.52E-09 |
| RP4-545L17.12 | ENSG00000274269 | 5.633353 | 2.493994 | 1.63E-31 | 1.62E-30 |
| AC008271.1 | ENSG00000231031 | 5.625869 | 2.492076 | 9.33E-09 | 2.16E-08 |
| RP11-3P17.4 | ENSG00000240567 | 5.623629 | 2.491502 | 4.61E-20 | 2.45E-19 |
| PCAT5 | ENSG00000280719 | 5.620397 | 2.490672 | 2.04E-11 | 5.95E-11 |
| AC012462.2 | ENSG00000225166 | 5.618889 | 2.490285 | 1.51E-12 | 4.73E-12 |
| AC106873.4 | ENSG00000228368 | 5.614374 | 2.489125 | 4.52E-11 | 1.28E-10 |
| RP11-83C7.1 | ENSG00000248227 | 5.608400 | 2.487589 | 1.96E-08 | 4.42E-08 |
| RP11-982M15.8 | ENSG00000260792 | 5.605476 | 2.486837 | 3.86E-31 | 3.77E-30 |
| RP11-701H16.4 | ENSG00000266954 | 5.604497 | 2.486585 | 4.78E-30 | 4.47E-29 |
| RP11-568J23.4 | ENSG00000269898 | 5.598459 | 2.485030 | 4.39E-13 | 1.45E-12 |
| AC004603.4 | ENSG00000225877 | 5.593349 | 2.483712 | 1.53E-07 | 3.21E-07 |
| CTD-2589M5.5 | ENSG00000254639 | 5.589689 | 2.482768 | 2.67E-14 | 9.62E-14 |
| CTB-138E5.1 | ENSG00000249639 | 5.585560 | 2.481702 | 1.09E-10 | 2.98E-10 |
| L3MBTL4-AS1 | ENSG00000264707 | 5.582299 | 2.480859 | 8.10E-53 | 2.04E-51 |
| RP11-429E11.3 | ENSG00000179253 | 5.549358 | 2.472321 | 5.76E-18 | 2.66E-17 |
| CTB-50L17.5 | ENSG00000267255 | 5.544544 | 2.471069 | 8.39E-14 | 2.92E-13 |
| AC114877.3 | ENSG00000234165 | 5.543636 | 2.470833 | 3.27E-09 | 7.85E-09 |
| RP11-181G12.4 | ENSG00000234396 | 5.540014 | 2.469890 | 1.73E-17 | 7.76E-17 |
| CTD-2195B23.3 | ENSG00000269652 | 5.534726 | 2.468512 | 2.43E-25 | 1.75E-24 |
| RP1-209A6.1 | ENSG00000233358 | 5.533579 | 2.468213 | 1.43E-07 | 3.01E-07 |
| RP11-70D24.3 | ENSG00000277214 | 5.531188 | 2.467589 | 3.83E-37 | 5.12E-36 |
| RP11-300M24.1 | ENSG00000227455 | 5.520785 | 2.464873 | 1.29E-07 | 2.71E-07 |
| C15orf54 | ENSG00000175746 | 5.511563 | 2.462461 | 4.29E-29 | 3.80E-28 |
| AC008067.2 | ENSG00000237031 | 5.508353 | 2.461621 | 1.13E-07 | 2.39E-07 |
| AC008268.1 | ENSG00000235584 | 5.503479 | 2.460344 | 2.75E-06 | 5.18E-06 |
| RP11-982M15.7 | ENSG00000258736 | 5.498915 | 2.459147 | 1.09E-15 | 4.33E-15 |
| RP11-383J24.1 | ENSG00000253227 | 5.489486 | 2.456671 | 5.32E-08 | 1.16E-07 |
| RP5-916L7.2 | ENSG00000256276 | 5.487414 | 2.456126 | 9.80E-19 | 4.76E-18 |
| XXbac-BPG299F13.14 | ENSG00000271821 | 5.487300 | 2.456096 | 1.65E-42 | 2.83E-41 |
| GRPEL2-AS1 | ENSG00000253618 | 5.474257 | 2.452663 | 2.07E-31 | 2.04E-30 |
| ATP11A-AS1 | ENSG00000232684 | 5.473356 | 2.452426 | 1.14E-17 | 5.17E-17 |
| AC096558.1 | ENSG00000228655 | 5.472934 | 2.452314 | 9.29E-20 | 4.85E-19 |
| NALCN-AS1 | ENSG00000233009 | 5.471334 | 2.451893 | 8.99E-11 | 2.48E-10 |
| MAFA-AS1 | ENSG00000254338 | 5.465133 | 2.450256 | 5.64E-09 | 1.33E-08 |
| ARHGAP26-AS1 | ENSG00000226272 | 5.464026 | 2.449964 | 5.43E-22 | 3.22E-21 |
| RP11-421P23.2 | ENSG00000254040 | 5.462938 | 2.449677 | 2.80E-21 | 1.61E-20 |
| RP1-71H24.1 | ENSG00000257452 | 5.461735 | 2.449359 | 2.58E-13 | 8.63E-13 |
| LINC00861 | ENSG00000245164 | 5.461432 | 2.449279 | 1.41E-40 | 2.21E-39 |
| CTD-2287O16.4 | ENSG00000272265 | 5.458219 | 2.448430 | 1.59E-20 | 8.65E-20 |
| AC003088.1 | ENSG00000226965 | 5.455084 | 2.447601 | 6.81E-13 | 2.20E-12 |
| AC011899.9 | ENSG00000233038 | 5.454933 | 2.447561 | 3.22E-40 | 4.96E-39 |
| CITF22-62D4.1 | ENSG00000268818 | 5.445445 | 2.445050 | 7.03E-10 | 1.80E-09 |
| RP11-5L12.1 | ENSG00000255097 | 5.441883 | 2.444106 | 1.15E-10 | 3.14E-10 |
| RP11-422J15.1 | ENSG00000249618 | 5.432397 | 2.441589 | 2.93E-08 | 6.52E-08 |
| RP11-20I20.4 | ENSG00000273179 | 5.431146 | 2.441257 | 2.99E-26 | 2.29E-25 |
| ITGB2-AS1 | ENSG00000227039 | 5.429679 | 2.440867 | 1.95E-35 | 2.40E-34 |
| RP11-640N11.2 | ENSG00000250493 | 5.428481 | 2.440549 | 3.49E-11 | 9.97E-11 |
| RP11-258F22.2 | ENSG00000260137 | 5.418283 | 2.437836 | 1.00E-15 | 4.00E-15 |
| RP13-60M5.2 | ENSG00000228189 | 5.413829 | 2.436649 | 7.34E-11 | 2.04E-10 |
| RP13-884E18.4 | ENSG00000250777 | 5.400283 | 2.433035 | 3.59E-10 | 9.39E-10 |
| RP11-148B3.2 | ENSG00000258346 | 5.399078 | 2.432713 | 1.91E-16 | 7.97E-16 |
| RP11-264E20.1 | ENSG00000255465 | 5.395453 | 2.431744 | 9.30E-27 | 7.28E-26 |
| RP11-395B7.4 | ENSG00000227053 | 5.394253 | 2.431423 | 3.02E-22 | 1.81E-21 |
| RP11-514D23.1 | ENSG00000268532 | 5.367027 | 2.424123 | 8.77E-08 | 1.87E-07 |
| RP11-126O1.2 | ENSG00000267579 | 5.362205 | 2.422826 | 2.21E-14 | 8.01E-14 |
| FOXC2-AS1 | ENSG00000260944 | 5.357264 | 2.421496 | 2.81E-13 | 9.40E-13 |
| RP11-73G16.1 | ENSG00000251249 | 5.352177 | 2.420126 | 7.85E-09 | 1.83E-08 |
| AC116614.1 | ENSG00000235688 | 5.345724 | 2.418385 | 2.66E-07 | 5.46E-07 |
| CASC20 | ENSG00000229876 | 5.340832 | 2.417064 | 2.03E-08 | 4.57E-08 |
| RP11-16E12.2 | ENSG00000259772 | 5.337536 | 2.416174 | 7.08E-19 | 3.48E-18 |
| RP11-282I1.1 | ENSG00000230131 | 5.328584 | 2.413752 | 7.00E-22 | 4.13E-21 |
| RP11-856M7.1 | ENSG00000260779 | 5.328024 | 2.413600 | 7.16E-13 | 2.31E-12 |
| RP11-321P16.1 | ENSG00000276923 | 5.319598 | 2.411317 | 3.55E-16 | 1.46E-15 |
| LA16c-390H2.4 | ENSG00000262312 | 5.315309 | 2.410154 | 3.29E-40 | 5.06E-39 |
| CTD-2013N17.4 | ENSG00000267703 | 5.301765 | 2.406473 | 8.18E-13 | 2.63E-12 |
| AC003003.5 | ENSG00000188477 | 5.294181 | 2.404407 | 2.63E-25 | 1.89E-24 |
| RP11-302L19.3 | ENSG00000273100 | 5.292248 | 2.403881 | 1.04E-34 | 1.22E-33 |
| LINC01272 | ENSG00000224397 | 5.283035 | 2.401367 | 4.49E-32 | 4.59E-31 |
| RP11-713C5.1 | ENSG00000265579 | 5.282692 | 2.401273 | 5.04E-15 | 1.92E-14 |
| AC112721.1 | ENSG00000222022 | 5.281783 | 2.401025 | 2.99E-07 | 6.11E-07 |
| LINC00868 | ENSG00000267535 | 5.272968 | 2.398615 | 2.61E-10 | 6.92E-10 |
| LINC01067 | ENSG00000236053 | 5.271809 | 2.398298 | 7.30E-07 | 1.44E-06 |
| RP4-536B24.3 | ENSG00000260177 | 5.259214 | 2.394847 | 2.40E-09 | 5.84E-09 |
| RP11-112L6.3 | ENSG00000237595 | 5.249675 | 2.392228 | 2.12E-34 | 2.47E-33 |
| LINC00710 | ENSG00000229240 | 5.245849 | 2.391176 | 7.24E-08 | 1.56E-07 |
| PHKA2-AS1 | ENSG00000237836 | 5.242934 | 2.390374 | 2.09E-52 | 5.13E-51 |
| LINC01526 | ENSG00000224995 | 5.241052 | 2.389857 | 8.12E-15 | 3.04E-14 |
| AC092667.2 | ENSG00000230393 | 5.240072 | 2.389587 | 8.75E-26 | 6.51E-25 |
| RP5-858L17.1 | ENSG00000276649 | 5.238142 | 2.389055 | 8.30E-45 | 1.54E-43 |
| RP11-552M14.1 | ENSG00000249645 | 5.237990 | 2.389013 | 5.02E-13 | 1.65E-12 |
| RP11-1026M7.2 | ENSG00000249109 | 5.235122 | 2.388223 | 3.23E-19 | 1.63E-18 |
| RP11-115J16.2 | ENSG00000254237 | 5.234940 | 2.388173 | 8.90E-07 | 1.74E-06 |
| RP5-1185I7.1 | ENSG00000232756 | 5.213831 | 2.382344 | 2.92E-08 | 6.51E-08 |
| RP11-2O17.2 | ENSG00000248529 | 5.213132 | 2.382150 | 7.22E-13 | 2.32E-12 |
| LINC01429 | ENSG00000227964 | 5.211160 | 2.381604 | 3.98E-08 | 8.74E-08 |
| AC018742.1 | ENSG00000229621 | 5.200472 | 2.378643 | 2.18E-09 | 5.31E-09 |
| RP11-263E1.1 | ENSG00000257325 | 5.196611 | 2.377571 | 3.27E-08 | 7.25E-08 |
| RP1-55C23.7 | ENSG00000234484 | 5.196324 | 2.377491 | 3.16E-15 | 1.22E-14 |
| RP11-96A15.1 | ENSG00000278464 | 5.173576 | 2.371162 | 4.70E-09 | 1.11E-08 |
| AC023669.1 | ENSG00000229459 | 5.170361 | 2.370265 | 8.32E-08 | 1.78E-07 |
| RP11-417L19.2 | ENSG00000256955 | 5.168294 | 2.369688 | 8.91E-10 | 2.25E-09 |
| SALRNA1 | ENSG00000258952 | 5.167729 | 2.369530 | 5.83E-13 | 1.90E-12 |
| RP11-45A17.4 | ENSG00000273521 | 5.164971 | 2.368760 | 1.40E-40 | 2.20E-39 |
| LINC00272 | ENSG00000203729 | 5.163276 | 2.368287 | 1.34E-13 | 4.58E-13 |
| CTC-329D1.3 | ENSG00000253965 | 5.150889 | 2.364821 | 7.92E-10 | 2.01E-09 |
| KCCAT211 | ENSG00000231574 | 5.143744 | 2.362819 | 9.13E-20 | 4.78E-19 |
| C20orf203 | ENSG00000198547 | 5.135061 | 2.360381 | 2.61E-23 | 1.67E-22 |
| KB-1980E6.3 | ENSG00000253633 | 5.132991 | 2.359800 | 7.54E-10 | 1.92E-09 |
| RP11-113I22.1 | ENSG00000251443 | 5.128124 | 2.358431 | 2.81E-10 | 7.43E-10 |
| RP11-550H2.1 | ENSG00000225605 | 5.126370 | 2.357937 | 4.82E-08 | 1.05E-07 |
| CTD-2542L18.1 | ENSG00000260417 | 5.125098 | 2.357580 | 1.24E-15 | 4.91E-15 |
| MYO16-AS1 | ENSG00000236242 | 5.118112 | 2.355612 | 2.73E-07 | 5.60E-07 |
| TSPEAR-AS2 | ENSG00000182912 | 5.117917 | 2.355557 | 1.06E-12 | 3.35E-12 |
| RP1-140K8.5 | ENSG00000260604 | 5.105825 | 2.352144 | 1.04E-17 | 4.72E-17 |
| PCED1B-AS1 | ENSG00000247774 | 5.097375 | 2.349754 | 1.68E-48 | 3.61E-47 |
| RP1-288H2.2 | ENSG00000257989 | 5.090343 | 2.347763 | 4.10E-34 | 4.70E-33 |
| RP13-714J12.1 | ENSG00000274373 | 5.087940 | 2.347082 | 7.72E-14 | 2.69E-13 |
| RP11-102G14.1 | ENSG00000277050 | 5.087747 | 2.347027 | 1.60E-31 | 1.59E-30 |
| RP1-29C18.8 | ENSG00000235111 | 5.086727 | 2.346738 | 1.83E-32 | 1.91E-31 |
| RP11-552D4.1 | ENSG00000228058 | 5.084869 | 2.346211 | 3.32E-19 | 1.67E-18 |
| RP11-61I13.3 | ENSG00000235033 | 5.084263 | 2.346039 | 4.96E-22 | 2.95E-21 |
| RP11-405M12.3 | ENSG00000278607 | 5.082847 | 2.345637 | 1.11E-22 | 6.83E-22 |
| CTC-254B4.1 | ENSG00000251027 | 5.082132 | 2.345434 | 2.42E-06 | 4.57E-06 |
| ASAP1-IT2 | ENSG00000280543 | 5.081248 | 2.345183 | 6.13E-30 | 5.67E-29 |
| LINC01105 | ENSG00000232044 | 5.080923 | 2.345091 | 2.00E-05 | 3.49E-05 |
| RP11-367G18.1 | ENSG00000230943 | 5.072696 | 2.342753 | 1.71E-18 | 8.17E-18 |
| CTD-2376I20.1 | ENSG00000258560 | 5.066494 | 2.340988 | 1.14E-15 | 4.52E-15 |
| RP11-73M11.3 | ENSG00000277545 | 5.063741 | 2.340204 | 3.83E-16 | 1.57E-15 |
| AC092635.1 | ENSG00000229370 | 5.063299 | 2.340078 | 1.94E-05 | 3.38E-05 |
| RP11-276E17.2 | ENSG00000234142 | 5.045481 | 2.334992 | 1.24E-15 | 4.89E-15 |
| RP11-108P20.2 | ENSG00000267501 | 5.041920 | 2.333973 | 3.74E-16 | 1.54E-15 |
| CTA-384D8.34 | ENSG00000273272 | 5.039709 | 2.333340 | 1.09E-18 | 5.30E-18 |
| RP1-63G5.7 | ENSG00000237862 | 5.038667 | 2.333042 | 5.44E-09 | 1.28E-08 |
| RP11-595B24.2 | ENSG00000263588 | 5.036803 | 2.332508 | 1.28E-07 | 2.70E-07 |
| RP11-81H14.1 | ENSG00000273824 | 5.027355 | 2.329800 | 1.17E-22 | 7.19E-22 |
| RP11-6O2.4 | ENSG00000261054 | 5.025456 | 2.329255 | 3.21E-18 | 1.51E-17 |
| RP11-145M4.1 | ENSG00000241042 | 5.020318 | 2.327779 | 4.35E-15 | 1.66E-14 |
| CTD-2118P12.1 | ENSG00000259786 | 5.005313 | 2.323460 | 5.09E-09 | 1.20E-08 |
| XXbac-B461K10.4 | ENSG00000093100 | 4.999758 | 2.321858 | 3.36E-45 | 6.36E-44 |
| RP11-789C17.1 | ENSG00000265413 | 4.998415 | 2.321471 | 1.30E-59 | 4.18E-58 |
| RP5-1112D6.4 | ENSG00000230177 | 4.993975 | 2.320188 | 3.00E-40 | 4.64E-39 |
| LINC00664 | ENSG00000268658 | 4.979899 | 2.316117 | 1.05E-20 | 5.82E-20 |
| RP11-1029J19.2 | ENSG00000259166 | 4.966211 | 2.312146 | 4.05E-05 | 6.84E-05 |
| RP11-678G15.2 | ENSG00000266767 | 4.962278 | 2.311003 | 1.66E-19 | 8.54E-19 |
| RP11-753H16.5 | ENSG00000258086 | 4.954918 | 2.308861 | 3.28E-27 | 2.62E-26 |
| RP11-566K11.7 | ENSG00000267048 | 4.954489 | 2.308736 | 1.04E-23 | 6.77E-23 |
| RP1-167G20.1 | ENSG00000248150 | 4.952526 | 2.308164 | 3.28E-13 | 1.09E-12 |
| RP11-284F21.10 | ENSG00000272405 | 4.952260 | 2.308087 | 3.89E-33 | 4.19E-32 |
| RP11-429J17.5 | ENSG00000254548 | 4.948964 | 2.307127 | 4.85E-15 | 1.85E-14 |
| VCAN-AS1 | ENSG00000249835 | 4.942129 | 2.305133 | 4.18E-18 | 1.95E-17 |
| RP11-201E8.1 | ENSG00000244464 | 4.941372 | 2.304912 | 2.99E-08 | 6.64E-08 |
| RP11-124N14.3 | ENSG00000234961 | 4.940755 | 2.304732 | 1.72E-41 | 2.84E-40 |
| RP11-434E6.4 | ENSG00000277559 | 4.929086 | 2.301320 | 2.53E-39 | 3.69E-38 |
| RP11-429A20.4 | ENSG00000256654 | 4.928619 | 2.301184 | 7.67E-06 | 1.38E-05 |
| RP5-940J5.3 | ENSG00000255966 | 4.923676 | 2.299736 | 1.73E-52 | 4.26E-51 |
| CTA-384D8.35 | ENSG00000272666 | 4.921144 | 2.298994 | 1.65E-30 | 1.56E-29 |
| RP11-763F8.1 | ENSG00000249049 | 4.916093 | 2.297512 | 7.44E-17 | 3.19E-16 |
| RP1-102K2.6 | ENSG00000232530 | 4.915700 | 2.297397 | 6.85E-12 | 2.06E-11 |
| LINC01480 | ENSG00000270164 | 4.912449 | 2.296442 | 1.79E-37 | 2.42E-36 |
| AC006145.4 | ENSG00000223770 | 4.909990 | 2.295720 | 1.96E-07 | 4.07E-07 |
| RP11-932O9.10 | ENSG00000269974 | 4.909926 | 2.295701 | 2.37E-24 | 1.60E-23 |
| RP11-388C12.5 | ENSG00000263321 | 4.909655 | 2.295622 | 9.95E-27 | 7.76E-26 |
| RP11-267L5.1 | ENSG00000254339 | 4.905869 | 2.294509 | 1.83E-05 | 3.21E-05 |
| TRG-AS1 | ENSG00000281103 | 4.903077 | 2.293687 | 1.85E-49 | 4.08E-48 |
| TRBV11-2 | ENSG00000241657 | 4.900551 | 2.292944 | 3.18E-16 | 1.31E-15 |
| AC006272.2 | ENSG00000268316 | 4.894847 | 2.291264 | 5.54E-32 | 5.61E-31 |
| LINC01522 | ENSG00000237423 | 4.893436 | 2.290848 | 3.19E-10 | 8.38E-10 |
| RP11-283C24.1 | ENSG00000264215 | 4.890139 | 2.289875 | 4.22E-15 | 1.61E-14 |
| RP11-478P10.1 | ENSG00000249584 | 4.882506 | 2.287622 | 3.99E-12 | 1.22E-11 |
| LINC01426 | ENSG00000234380 | 4.881402 | 2.287296 | 3.22E-17 | 1.42E-16 |
| CTD-2363C16.1 | ENSG00000253811 | 4.876233 | 2.285767 | 1.05E-09 | 2.63E-09 |
| RP4-712E4.1 | ENSG00000226172 | 4.869002 | 2.283626 | 3.17E-11 | 9.10E-11 |
| CTD-2571L23.8 | ENSG00000268746 | 4.859727 | 2.280875 | 2.73E-31 | 2.69E-30 |
| RP1-167A14.2 | ENSG00000227598 | 4.858260 | 2.280440 | 4.67E-38 | 6.48E-37 |
| CTD-2263F21.1 | ENSG00000251257 | 4.857608 | 2.280246 | 1.27E-16 | 5.37E-16 |
| RP11-58A18.1 | ENSG00000261161 | 4.856413 | 2.279891 | 5.27E-11 | 1.48E-10 |
| RP11-662G23.1 | ENSG00000254251 | 4.853066 | 2.278896 | 1.14E-07 | 2.41E-07 |
| RP11-267N12.1 | ENSG00000232892 | 4.848643 | 2.277581 | 8.48E-28 | 7.02E-27 |
| AC159540.1 | ENSG00000230606 | 4.838316 | 2.274505 | 1.42E-38 | 2.00E-37 |
| RP11-75L1.1 | ENSG00000256582 | 4.832644 | 2.272813 | 7.52E-21 | 4.19E-20 |
| GRID1-AS1 | ENSG00000234942 | 4.828831 | 2.271674 | 1.12E-10 | 3.06E-10 |
| AP000997.1 | ENSG00000255599 | 4.827073 | 2.271149 | 2.98E-09 | 7.17E-09 |
| RP11-42O4.2 | ENSG00000278367 | 4.826639 | 2.271019 | 1.07E-26 | 8.35E-26 |
| AC067945.4 | ENSG00000231858 | 4.826314 | 2.270922 | 1.13E-34 | 1.33E-33 |
| AC017104.2 | ENSG00000233538 | 4.823796 | 2.270169 | 6.53E-14 | 2.29E-13 |
| AC091814.3 | ENSG00000231560 | 4.820358 | 2.269140 | 2.27E-20 | 1.23E-19 |
| RP3-404F18.5 | ENSG00000237903 | 4.820344 | 2.269136 | 2.36E-19 | 1.20E-18 |
| RP11-724M22.1 | ENSG00000251523 | 4.816819 | 2.268081 | 5.14E-07 | 1.03E-06 |
| RP11-476D10.1 | ENSG00000260943 | 4.814873 | 2.267498 | 3.67E-17 | 1.60E-16 |
| RP11-609N14.1 | ENSG00000260756 | 4.803801 | 2.264176 | 2.39E-08 | 5.34E-08 |
| RP11-893F2.5 | ENSG00000249406 | 4.796812 | 2.262076 | 3.65E-16 | 1.50E-15 |
| AC007362.3 | ENSG00000225216 | 4.784438 | 2.258349 | 3.56E-18 | 1.67E-17 |
| RP13-297E16.5 | ENSG00000234622 | 4.783528 | 2.258075 | 6.86E-11 | 1.91E-10 |
| AC007750.5 | ENSG00000236841 | 4.781560 | 2.257481 | 2.71E-23 | 1.72E-22 |
| CTD-2282P23.2 | ENSG00000274441 | 4.780394 | 2.257129 | 5.28E-13 | 1.73E-12 |
| LINC01440 | ENSG00000235166 | 4.777876 | 2.256369 | 6.89E-07 | 1.36E-06 |
| CTD-2024I7.13 | ENSG00000246422 | 4.777593 | 2.256284 | 8.67E-13 | 2.78E-12 |
| RP3-413H6.2 | ENSG00000234427 | 4.764999 | 2.252476 | 1.04E-12 | 3.30E-12 |
| RP4-569D19.8 | ENSG00000273044 | 4.759273 | 2.250741 | 8.16E-13 | 2.62E-12 |
| SOX21-AS1 | ENSG00000227640 | 4.756643 | 2.249944 | 1.18E-13 | 4.06E-13 |
| CTD-2562J17.4 | ENSG00000255136 | 4.751530 | 2.248392 | 9.63E-11 | 2.65E-10 |
| MIR296 | ENSG00000268649 | 4.750753 | 2.248156 | 6.06E-14 | 2.13E-13 |
| LINC01620 | ENSG00000168746 | 4.749412 | 2.247749 | 3.05E-12 | 9.37E-12 |
| RP11-472N13.2 | ENSG00000223834 | 4.734812 | 2.243307 | 1.13E-33 | 1.26E-32 |
| CTD-2201E18.5 | ENSG00000271788 | 4.734344 | 2.243164 | 7.63E-34 | 8.59E-33 |
| RP11-15M15.2 | ENSG00000227705 | 4.733990 | 2.243057 | 4.25E-09 | 1.01E-08 |
| AC104820.2 | ENSG00000234663 | 4.727261 | 2.241004 | 5.63E-23 | 3.52E-22 |
| AC091153.4 | ENSG00000235085 | 4.724343 | 2.240114 | 4.44E-13 | 1.46E-12 |
| LINC00862 | ENSG00000203721 | 4.713055 | 2.236663 | 6.66E-13 | 2.16E-12 |
| CTA-228A9.3 | ENSG00000272720 | 4.710520 | 2.235886 | 3.94E-37 | 5.27E-36 |
| LINC00589 | ENSG00000251191 | 4.709237 | 2.235493 | 5.10E-15 | 1.94E-14 |
| LINC01502 | ENSG00000237339 | 4.708604 | 2.235299 | 7.41E-06 | 1.34E-05 |
| RP11-9N12.2 | ENSG00000278981 | 4.707056 | 2.234825 | 1.12E-07 | 2.38E-07 |
| LINC01492 | ENSG00000225564 | 4.702588 | 2.233455 | 1.91E-08 | 4.32E-08 |
| RAPGEF4-AS1 | ENSG00000228016 | 4.698640 | 2.232243 | 2.09E-21 | 1.21E-20 |
| RP11-44N11.2 | ENSG00000272384 | 4.697335 | 2.231842 | 5.46E-28 | 4.56E-27 |
| RP11-837J7.4 | ENSG00000277299 | 4.696826 | 2.231686 | 4.47E-32 | 4.57E-31 |
| ASMTL-AS1 | ENSG00000236017 | 4.691269 | 2.229978 | 4.02E-26 | 3.05E-25 |
| RP1-170O19.17 | ENSG00000253308 | 4.683747 | 2.227663 | 6.19E-06 | 1.12E-05 |
| RP11-41O4.2 | ENSG00000266278 | 4.681760 | 2.227051 | 6.67E-10 | 1.71E-09 |
| AC078842.3 | ENSG00000228031 | 4.672577 | 2.224218 | 8.84E-10 | 2.23E-09 |
| AC091177.1 | ENSG00000230113 | 4.672560 | 2.224213 | 2.73E-28 | 2.32E-27 |
| ABHD11-AS1 | ENSG00000225969 | 4.666617 | 2.222377 | 3.53E-24 | 2.36E-23 |
| C20orf197 | ENSG00000176659 | 4.655948 | 2.219075 | 3.55E-45 | 6.69E-44 |
| RP1-16A9.1 | ENSG00000253983 | 4.655816 | 2.219034 | 5.37E-17 | 2.32E-16 |
| LINC00632 | ENSG00000203930 | 4.652123 | 2.217889 | 3.19E-15 | 1.23E-14 |
| RP11-776H12.1 | ENSG00000226476 | 4.649404 | 2.217046 | 2.18E-09 | 5.32E-09 |
| AJ239322.1 | ENSG00000235615 | 4.643439 | 2.215194 | 2.14E-07 | 4.42E-07 |
| RP11-437L7.1 | ENSG00000260850 | 4.636626 | 2.213075 | 6.69E-08 | 1.44E-07 |
| LINC00895 | ENSG00000281548 | 4.629806 | 2.210952 | 2.94E-08 | 6.53E-08 |
| EML2-AS1 | ENSG00000267757 | 4.616521 | 2.206806 | 2.71E-10 | 7.16E-10 |
| RP11-192P3.4 | ENSG00000272381 | 4.610334 | 2.204871 | 8.98E-24 | 5.88E-23 |
| CTA-243E7.4 | ENSG00000279085 | 4.604786 | 2.203134 | 2.30E-17 | 1.02E-16 |
| AC018685.1 | ENSG00000234929 | 4.602737 | 2.202492 | 2.86E-07 | 5.86E-07 |
| AC002044.4 | ENSG00000272330 | 4.594319 | 2.199851 | 1.69E-13 | 5.75E-13 |
| RP11-346L1.2 | ENSG00000253344 | 4.579421 | 2.195165 | 1.49E-07 | 3.12E-07 |
| RP5-859D4.3 | ENSG00000228482 | 4.572733 | 2.193057 | 9.70E-07 | 1.90E-06 |
| RP11-327F22.6 | ENSG00000270120 | 4.570534 | 2.192363 | 5.60E-16 | 2.27E-15 |
| RP1-144F13.3 | ENSG00000225903 | 4.567108 | 2.191281 | 2.33E-13 | 7.86E-13 |
| AC092675.3 | ENSG00000222000 | 4.567086 | 2.191274 | 4.83E-09 | 1.14E-08 |
| RP11-39M21.1 | ENSG00000259481 | 4.560775 | 2.189279 | 1.75E-10 | 4.73E-10 |
| LINC00426 | ENSG00000238121 | 4.550878 | 2.186145 | 2.70E-32 | 2.78E-31 |
| RP11-426C22.4 | ENSG00000259807 | 4.545820 | 2.184541 | 1.40E-23 | 9.03E-23 |
| RP11-59O6.3 | ENSG00000235880 | 4.545413 | 2.184411 | 8.53E-08 | 1.82E-07 |
| RP11-513G11.4 | ENSG00000225742 | 4.542757 | 2.183568 | 1.56E-21 | 9.09E-21 |
| AC005387.2 | ENSG00000269191 | 4.541492 | 2.183166 | 9.58E-38 | 1.31E-36 |
| PCAT29 | ENSG00000259641 | 4.532296 | 2.180242 | 1.17E-23 | 7.55E-23 |
| RP11-16E23.3 | ENSG00000261544 | 4.526423 | 2.178371 | 1.20E-11 | 3.53E-11 |
| RP11-53B2.1 | ENSG00000267529 | 4.515741 | 2.174963 | 1.71E-18 | 8.19E-18 |
| LINC01260 | ENSG00000132832 | 4.513354 | 2.174200 | 1.03E-22 | 6.38E-22 |
| RP11-818F20.5 | ENSG00000257732 | 4.508655 | 2.172697 | 2.40E-10 | 6.39E-10 |
| RP11-1334A24.6 | ENSG00000248996 | 4.507623 | 2.172367 | 5.26E-45 | 9.83E-44 |
| RP11-332J15.1 | ENSG00000249734 | 4.506944 | 2.172149 | 7.15E-13 | 2.31E-12 |
| AC107218.3 | ENSG00000197585 | 4.506800 | 2.172103 | 9.34E-18 | 4.26E-17 |
| RP11-359E10.1 | ENSG00000270607 | 4.505939 | 2.171828 | 4.52E-30 | 4.23E-29 |
| RP11-707A18.1 | ENSG00000250125 | 4.503301 | 2.170983 | 2.46E-05 | 4.24E-05 |
| RP11-85O21.5 | ENSG00000234921 | 4.493625 | 2.167880 | 2.63E-07 | 5.39E-07 |
| RP11-632K5.3 | ENSG00000246211 | 4.492253 | 2.167439 | 7.73E-13 | 2.48E-12 |
| RP11-354P11.8 | ENSG00000266987 | 4.491797 | 2.167293 | 2.11E-12 | 6.55E-12 |
| TCF4-AS2 | ENSG00000267402 | 4.479047 | 2.163192 | 1.46E-06 | 2.82E-06 |
| XXbac-BPG27H4.8 | ENSG00000237923 | 4.478779 | 2.163105 | 1.73E-07 | 3.62E-07 |
| AC003092.1 | ENSG00000236453 | 4.477585 | 2.162721 | 4.89E-06 | 8.98E-06 |
| AC091729.8 | ENSG00000226291 | 4.477509 | 2.162696 | 3.61E-10 | 9.44E-10 |
| RP11-242J7.1 | ENSG00000254233 | 4.474850 | 2.161839 | 3.81E-07 | 7.71E-07 |
| RP11-696F12.1 | ENSG00000250971 | 4.470871 | 2.160556 | 9.31E-10 | 2.34E-09 |
| RP1-228P16.4 | ENSG00000257985 | 4.469271 | 2.160040 | 1.67E-09 | 4.11E-09 |
| CTB-35F21.3 | ENSG00000251387 | 4.466033 | 2.158994 | 3.23E-15 | 1.25E-14 |
| AP000997.2 | ENSG00000260254 | 4.464373 | 2.158458 | 3.92E-08 | 8.62E-08 |
| AC009518.4 | ENSG00000224865 | 4.456930 | 2.156050 | 2.33E-06 | 4.42E-06 |
| LINC01146 | ENSG00000258867 | 4.452442 | 2.154597 | 1.16E-25 | 8.53E-25 |
| RP11-365O16.6 | ENSG00000237101 | 4.451770 | 2.154379 | 7.91E-47 | 1.60E-45 |
| RP11-25K21.6 | ENSG00000273112 | 4.450949 | 2.154113 | 1.15E-13 | 3.96E-13 |
| RP3-455J7.4 | ENSG00000241666 | 4.444888 | 2.152147 | 9.01E-26 | 6.68E-25 |
| RP11-297C4.2 | ENSG00000261346 | 4.444186 | 2.151919 | 1.29E-12 | 4.07E-12 |
| RP11-572O17.1 | ENSG00000270195 | 4.443004 | 2.151535 | 2.09E-31 | 2.07E-30 |
| RP11-356J5.12 | ENSG00000250303 | 4.441088 | 2.150913 | 4.59E-37 | 6.11E-36 |
| RP3-395M20.3 | ENSG00000229393 | 4.435372 | 2.149055 | 1.22E-10 | 3.34E-10 |
| RP11-711K1.7 | ENSG00000255790 | 4.434585 | 2.148799 | 6.53E-05 | 1.08E-04 |
| RP11-202G18.1 | ENSG00000227531 | 4.432814 | 2.148223 | 1.13E-11 | 3.35E-11 |
| CTD-2587H19.3 | ENSG00000269873 | 4.423950 | 2.145335 | 3.78E-19 | 1.89E-18 |
| CTD-2528A14.5 | ENSG00000269066 | 4.423355 | 2.145141 | 2.09E-13 | 7.08E-13 |
| AC147651.4 | ENSG00000237181 | 4.419666 | 2.143937 | 8.02E-41 | 1.28E-39 |
| LINC01021 | ENSG00000250337 | 4.418006 | 2.143395 | 5.23E-11 | 1.47E-10 |
| AC092580.4 | ENSG00000235576 | 4.416082 | 2.142767 | 4.06E-30 | 3.81E-29 |
| HP09025 | ENSG00000267719 | 4.409829 | 2.140723 | 7.77E-14 | 2.71E-13 |
| RP11-861E21.2 | ENSG00000267199 | 4.400683 | 2.137727 | 6.83E-38 | 9.38E-37 |
| AC016700.2 | ENSG00000233060 | 4.399065 | 2.137197 | 3.28E-13 | 1.09E-12 |
| RP11-563N6.6 | ENSG00000228403 | 4.393961 | 2.135522 | 1.80E-12 | 5.61E-12 |
| RP13-192B19.2 | ENSG00000260585 | 4.389587 | 2.134085 | 1.07E-14 | 3.97E-14 |
| RP11-445O3.3 | ENSG00000260763 | 4.387921 | 2.133537 | 2.23E-06 | 4.22E-06 |
| RP11-310H4.3 | ENSG00000231394 | 4.385464 | 2.132730 | 9.86E-13 | 3.14E-12 |
| RP11-472G21.2 | ENSG00000230526 | 4.383992 | 2.132245 | 2.33E-16 | 9.66E-16 |
| RP11-115H15.2 | ENSG00000258337 | 4.377422 | 2.130082 | 8.71E-09 | 2.02E-08 |
| RP11-177B4.2 | ENSG00000260120 | 4.355620 | 2.122878 | 4.27E-10 | 1.11E-09 |
| RP11-867G23.12 | ENSG00000254756 | 4.351486 | 2.121508 | 8.23E-18 | 3.77E-17 |
| AC097382.5 | ENSG00000187904 | 4.351224 | 2.121421 | 7.21E-13 | 2.32E-12 |
| LINC00824 | ENSG00000254275 | 4.350391 | 2.121145 | 2.28E-12 | 7.05E-12 |
| RP5-968J1.1 | ENSG00000230839 | 4.341705 | 2.118262 | 1.10E-23 | 7.12E-23 |
| AC015849.16 | ENSG00000270977 | 4.341241 | 2.118107 | 5.61E-25 | 3.94E-24 |
| USP30-AS1 | ENSG00000256262 | 4.339667 | 2.117584 | 4.92E-30 | 4.59E-29 |
| AP003774.1 | ENSG00000236935 | 4.331062 | 2.114721 | 4.02E-26 | 3.05E-25 |
| RP5-1096D14.3 | ENSG00000256706 | 4.321885 | 2.111661 | 5.84E-14 | 2.06E-13 |
| RP11-274H2.3 | ENSG00000240032 | 4.321119 | 2.111405 | 3.95E-23 | 2.49E-22 |
| RP5-998N21.4 | ENSG00000234571 | 4.314938 | 2.109340 | 8.88E-23 | 5.50E-22 |
| FAM30A | ENSG00000226777 | 4.306380 | 2.106476 | 6.05E-11 | 1.70E-10 |
| RP1-46F2.3 | ENSG00000258240 | 4.305535 | 2.106192 | 5.96E-06 | 1.09E-05 |
| RP5-1031D4.2 | ENSG00000232591 | 4.302207 | 2.105077 | 4.76E-23 | 2.98E-22 |
| RP11-227G15.9 | ENSG00000264083 | 4.299932 | 2.104314 | 6.45E-24 | 4.25E-23 |
| RP11-848P1.3 | ENSG00000265743 | 4.294191 | 2.102386 | 2.07E-69 | 8.96E-68 |
| BX255923.3 | ENSG00000276462 | 4.286364 | 2.099754 | 1.58E-11 | 4.61E-11 |
| RP11-642C5.1 | ENSG00000271156 | 4.285514 | 2.099468 | 4.02E-14 | 1.43E-13 |
| AC006369.2 | ENSG00000236213 | 4.282934 | 2.098599 | 2.43E-28 | 2.08E-27 |
| LINC01433 | ENSG00000230176 | 4.280949 | 2.097931 | 9.22E-19 | 4.49E-18 |
| RP11-785D18.3 | ENSG00000277247 | 4.279559 | 2.097462 | 1.14E-10 | 3.13E-10 |
| RP11-524N5.1 | ENSG00000276778 | 4.279461 | 2.097429 | 7.72E-15 | 2.90E-14 |
| XX-C00717C00720L.1 | ENSG00000279182 | 4.278283 | 2.097032 | 4.58E-24 | 3.04E-23 |
| CASC11 | ENSG00000249375 | 4.277947 | 2.096919 | 3.99E-32 | 4.09E-31 |
| RP11-261P9.4 | ENSG00000254419 | 4.276606 | 2.096466 | 2.31E-24 | 1.56E-23 |
| RP11-523O18.5 | ENSG00000233665 | 4.272556 | 2.095099 | 3.14E-29 | 2.82E-28 |
| AF131215.4 | ENSG00000254556 | 4.271027 | 2.094583 | 9.86E-10 | 2.48E-09 |
| RP11-8L2.1 | ENSG00000250546 | 4.268579 | 2.093756 | 5.11E-06 | 9.37E-06 |
| RP11-318A15.8 | ENSG00000277382 | 4.268490 | 2.093726 | 2.89E-12 | 8.90E-12 |
| RP5-1029K10.2 | ENSG00000249906 | 4.265312 | 2.092651 | 1.57E-14 | 5.77E-14 |
| RP11-310H4.2 | ENSG00000233977 | 4.262406 | 2.091668 | 1.29E-12 | 4.07E-12 |
| RP11-338E21.2 | ENSG00000225195 | 4.260921 | 2.091165 | 2.70E-10 | 7.16E-10 |
| RP11-124N19.3 | ENSG00000262198 | 4.258767 | 2.090436 | 5.02E-14 | 1.78E-13 |
| RP11-222K16.2 | ENSG00000272282 | 4.256118 | 2.089538 | 8.43E-29 | 7.38E-28 |
| RP11-81H14.2 | ENSG00000251301 | 4.254654 | 2.089042 | 1.02E-15 | 4.07E-15 |
| RP11-326C3.14 | ENSG00000270105 | 4.253252 | 2.088566 | 4.03E-15 | 1.55E-14 |
| RP1-244F24.1 | ENSG00000271857 | 4.251724 | 2.088048 | 1.07E-23 | 6.93E-23 |
| RP13-494C23.1 | ENSG00000251009 | 4.248447 | 2.086936 | 1.44E-22 | 8.81E-22 |
| RP1-142L7.8 | ENSG00000271208 | 4.248301 | 2.086886 | 4.95E-11 | 1.40E-10 |
| CTB-73N10.1 | ENSG00000248125 | 4.246598 | 2.086308 | 1.83E-14 | 6.67E-14 |
| RP11-556O9.2 | ENSG00000264513 | 4.243788 | 2.085353 | 8.85E-12 | 2.63E-11 |
| RP11-344E13.4 | ENSG00000266369 | 4.232306 | 2.081444 | 3.00E-07 | 6.12E-07 |
| KB-173C10.1 | ENSG00000253842 | 4.232204 | 2.081409 | 5.54E-09 | 1.31E-08 |
| GS1-174L6.4 | ENSG00000224691 | 4.230544 | 2.080843 | 1.76E-14 | 6.42E-14 |
| CTD-2035E11.5 | ENSG00000272144 | 4.226250 | 2.079378 | 1.17E-39 | 1.73E-38 |
| CTD-2034I4.2 | ENSG00000259594 | 4.224347 | 2.078728 | 4.18E-09 | 9.95E-09 |
| RP11-314M24.1 | ENSG00000240241 | 4.222613 | 2.078136 | 2.69E-04 | 4.19E-04 |
| RP11-54A4.2 | ENSG00000237781 | 4.222424 | 2.078071 | 2.52E-29 | 2.28E-28 |
| RP13-1016M1.2 | ENSG00000272783 | 4.222295 | 2.078027 | 4.66E-17 | 2.02E-16 |
| AC000036.4 | ENSG00000225929 | 4.218417 | 2.076702 | 1.55E-17 | 6.96E-17 |
| RP11-274H2.2 | ENSG00000243415 | 4.216283 | 2.075972 | 1.03E-22 | 6.34E-22 |
| PIK3CD-AS1 | ENSG00000179840 | 4.204072 | 2.071787 | 9.33E-25 | 6.47E-24 |
| RP11-480A16.1 | ENSG00000260261 | 4.203342 | 2.071537 | 2.96E-34 | 3.41E-33 |
| RP11-462B18.2 | ENSG00000231193 | 4.203057 | 2.071439 | 4.37E-11 | 1.24E-10 |
| RP11-497H17.1 | ENSG00000262663 | 4.199564 | 2.070239 | 3.43E-29 | 3.07E-28 |
| PCGEM1 | ENSG00000227418 | 4.198345 | 2.069821 | 1.97E-05 | 3.42E-05 |
| RP11-488C13.6 | ENSG00000259081 | 4.197639 | 2.069578 | 1.22E-35 | 1.53E-34 |
| RP11-881L2.1 | ENSG00000266541 | 4.196533 | 2.069198 | 9.52E-14 | 3.29E-13 |
| RP11-1250I15.3 | ENSG00000271771 | 4.195000 | 2.068671 | 3.52E-10 | 9.21E-10 |
| ARAP1-AS2 | ENSG00000245148 | 4.191140 | 2.067343 | 1.05E-29 | 9.60E-29 |
| CTD-2530N21.5 | ENSG00000177725 | 4.187326 | 2.066029 | 1.12E-11 | 3.31E-11 |
| RP5-1039K5.16 | ENSG00000222044 | 4.186776 | 2.065840 | 2.98E-14 | 1.07E-13 |
| SLC6A1-AS1 | ENSG00000232287 | 4.185321 | 2.065338 | 1.03E-20 | 5.70E-20 |
| PLA2G4C-AS1 | ENSG00000269420 | 4.184297 | 2.064985 | 8.34E-28 | 6.91E-27 |
| LINC00706 | ENSG00000281186 | 4.184042 | 2.064897 | 1.71E-05 | 3.00E-05 |
| RP11-42A4.1 | ENSG00000248749 | 4.176952 | 2.062450 | 2.58E-09 | 6.25E-09 |
| RP11-6B19.1 | ENSG00000255565 | 4.175171 | 2.061835 | 2.11E-06 | 4.02E-06 |
| RP11-328K4.1 | ENSG00000248740 | 4.172071 | 2.060764 | 5.44E-04 | 8.22E-04 |
| RP11-737O24.1 | ENSG00000266397 | 4.171570 | 2.060591 | 4.00E-16 | 1.64E-15 |
| RP11-459O16.8 | ENSG00000276128 | 4.169911 | 2.060017 | 1.75E-10 | 4.71E-10 |
| AC017060.1 | ENSG00000236039 | 4.164095 | 2.058003 | 2.31E-13 | 7.79E-13 |
| RP11-439C15.4 | ENSG00000253764 | 4.158368 | 2.056017 | 6.54E-27 | 5.16E-26 |
| FGF12-AS2 | ENSG00000230126 | 4.156396 | 2.055333 | 1.17E-16 | 4.97E-16 |
| RP11-133F8.2 | ENSG00000249776 | 4.154273 | 2.054596 | 1.03E-12 | 3.29E-12 |
| CTC-523E23.5 | ENSG00000269086 | 4.151723 | 2.053710 | 8.51E-48 | 1.78E-46 |
| RP3-477O4.14 | ENSG00000230155 | 4.150102 | 2.053147 | 1.03E-48 | 2.21E-47 |
| RP11-616M22.5 | ENSG00000260182 | 4.149884 | 2.053071 | 3.06E-10 | 8.06E-10 |
| DKFZP434L187 | ENSG00000225930 | 4.148590 | 2.052621 | 1.96E-06 | 3.74E-06 |
| RP11-384C4.6 | ENSG00000230063 | 4.142858 | 2.050627 | 6.63E-08 | 1.43E-07 |
| AC008746.12 | ENSG00000267838 | 4.127086 | 2.045123 | 8.80E-35 | 1.04E-33 |
| RP11-244M2.1 | ENSG00000267374 | 4.126706 | 2.044991 | 1.10E-21 | 6.46E-21 |
| RP11-404F10.2 | ENSG00000228863 | 4.124408 | 2.044187 | 1.24E-19 | 6.43E-19 |
| KCNMB2-AS1 | ENSG00000237978 | 4.122364 | 2.043472 | 7.24E-13 | 2.33E-12 |
| CTC-453G23.5 | ENSG00000269534 | 4.117221 | 2.041671 | 4.76E-25 | 3.36E-24 |
| RP11-2L8.1 | ENSG00000225790 | 4.116083 | 2.041272 | 6.34E-14 | 2.23E-13 |
| TRPM2-AS | ENSG00000230061 | 4.115449 | 2.041050 | 3.14E-21 | 1.79E-20 |
| CTD-2196E14.5 | ENSG00000261266 | 4.115286 | 2.040993 | 1.40E-12 | 4.42E-12 |
| AC010894.5 | ENSG00000237798 | 4.114076 | 2.040568 | 6.68E-14 | 2.34E-13 |
| RP5-1077H22.1 | ENSG00000278343 | 4.107156 | 2.038140 | 2.34E-09 | 5.69E-09 |
| RP1-149A16.12 | ENSG00000234626 | 4.105132 | 2.037429 | 1.66E-10 | 4.47E-10 |
| AC002480.2 | ENSG00000238033 | 4.101972 | 2.036318 | 1.15E-08 | 2.63E-08 |
| RP11-525K10.3 | ENSG00000259867 | 4.100873 | 2.035931 | 1.66E-32 | 1.73E-31 |
| LINC01150 | ENSG00000229671 | 4.099734 | 2.035530 | 3.84E-29 | 3.42E-28 |
| AC009784.3 | ENSG00000232053 | 4.096429 | 2.034367 | 2.22E-11 | 6.44E-11 |
| RP11-302F12.3 | ENSG00000248545 | 4.092574 | 2.033008 | 2.06E-05 | 3.57E-05 |
| RP11-893F2.6 | ENSG00000251085 | 4.092010 | 2.032810 | 7.39E-10 | 1.88E-09 |
| RP13-516M14.10 | ENSG00000275888 | 4.090776 | 2.032375 | 4.76E-33 | 5.10E-32 |
| LINC00973 | ENSG00000240476 | 4.089204 | 2.031820 | 3.35E-05 | 5.72E-05 |
| AC015933.2 | ENSG00000227279 | 4.087458 | 2.031204 | 1.47E-26 | 1.14E-25 |
| MNX1-AS2 | ENSG00000235029 | 4.085242 | 2.030421 | 1.38E-09 | 3.42E-09 |
| AC108676.1 | ENSG00000244675 | 4.083737 | 2.029890 | 2.75E-24 | 1.85E-23 |
| RP11-61G19.1 | ENSG00000249334 | 4.082612 | 2.029493 | 5.29E-06 | 9.67E-06 |
| AC005306.3 | ENSG00000267283 | 4.081260 | 2.029015 | 2.99E-27 | 2.40E-26 |
| CTD-2537I9.13 | ENSG00000267096 | 4.078762 | 2.028131 | 1.15E-26 | 8.96E-26 |
| RP4-665J23.1 | ENSG00000233593 | 4.074985 | 2.026795 | 1.27E-28 | 1.10E-27 |
| RP11-357N13.3 | ENSG00000261630 | 4.070199 | 2.025099 | 7.51E-17 | 3.22E-16 |
| AP001055.6 | ENSG00000225331 | 4.066186 | 2.023676 | 2.09E-24 | 1.42E-23 |
| LRP1-AS | ENSG00000259125 | 4.065827 | 2.023549 | 7.05E-13 | 2.27E-12 |
| RP11-867G23.1 | ENSG00000254855 | 4.060879 | 2.021792 | 2.69E-16 | 1.11E-15 |
| RP11-425D17.2 | ENSG00000273989 | 4.060314 | 2.021591 | 1.35E-08 | 3.08E-08 |
| CH17-360D5.2 | ENSG00000276850 | 4.060290 | 2.021583 | 2.29E-05 | 3.96E-05 |
| RP11-344N10.2 | ENSG00000237768 | 4.059798 | 2.021408 | 2.61E-12 | 8.05E-12 |
| RP11-149I2.4 | ENSG00000266446 | 4.056659 | 2.020292 | 6.86E-11 | 1.92E-10 |
| RP3-460G2.2 | ENSG00000234147 | 4.055924 | 2.020031 | 6.07E-16 | 2.45E-15 |
| RP11-387H17.6 | ENSG00000265799 | 4.055742 | 2.019966 | 1.40E-08 | 3.19E-08 |
| RP11-10N16.2 | ENSG00000230023 | 4.054809 | 2.019634 | 5.48E-09 | 1.29E-08 |
| RP11-60A8.1 | ENSG00000249743 | 4.052888 | 2.018950 | 8.36E-12 | 2.50E-11 |
| RP11-327F22.1 | ENSG00000261644 | 4.052559 | 2.018833 | 6.11E-33 | 6.52E-32 |
| YEATS2-AS1 | ENSG00000233885 | 4.052144 | 2.018685 | 1.88E-50 | 4.28E-49 |
| RP11-483L5.1 | ENSG00000255462 | 4.050168 | 2.017982 | 3.79E-20 | 2.02E-19 |
| XXyac-YX65C7_A.3 | ENSG00000233085 | 4.049761 | 2.017837 | 1.41E-07 | 2.97E-07 |
| RP1-278C19.8 | ENSG00000275936 | 4.049388 | 2.017704 | 1.13E-21 | 6.60E-21 |
| RP11-39M21.2 | ENSG00000259575 | 4.046383 | 2.016633 | 1.46E-13 | 4.97E-13 |
| TSPEAR-AS1 | ENSG00000235890 | 4.042135 | 2.015118 | 9.50E-11 | 2.61E-10 |
| AC002306.1 | ENSG00000259242 | 4.041065 | 2.014736 | 9.07E-11 | 2.50E-10 |
| RP11-312J18.6 | ENSG00000232188 | 4.039873 | 2.014310 | 3.88E-04 | 5.95E-04 |
| CTD-3195I5.3 | ENSG00000262692 | 4.039650 | 2.014230 | 2.75E-33 | 2.98E-32 |
| RP11-307O13.1 | ENSG00000261728 | 4.036247 | 2.013015 | 8.12E-12 | 2.43E-11 |
| AC137932.6 | ENSG00000261253 | 4.032192 | 2.011564 | 1.33E-36 | 1.73E-35 |
| RP11-617B3.2 | ENSG00000255548 | 4.030673 | 2.011021 | 7.68E-17 | 3.29E-16 |
| RP1-158P9.1 | ENSG00000235612 | 4.030657 | 2.011015 | 1.50E-17 | 6.77E-17 |
| RP11-867G23.13 | ENSG00000254458 | 4.030377 | 2.010915 | 3.46E-12 | 1.06E-11 |
| RP11-678G15.1 | ENSG00000264596 | 4.028920 | 2.010393 | 3.10E-11 | 8.92E-11 |
| RP5-899E9.1 | ENSG00000273341 | 4.028879 | 2.010378 | 3.60E-39 | 5.19E-38 |
| AP000476.1 | ENSG00000237484 | 4.026669 | 2.009587 | 1.27E-25 | 9.35E-25 |
| RP11-115J16.1 | ENSG00000254235 | 4.025765 | 2.009263 | 1.67E-12 | 5.23E-12 |
| CTB-181F24.1 | ENSG00000254066 | 4.023307 | 2.008382 | 2.82E-06 | 5.30E-06 |
| RP11-219E7.3 | ENSG00000259130 | 4.020111 | 2.007235 | 3.96E-18 | 1.85E-17 |
| CTC-344H19.4 | ENSG00000268401 | 4.019586 | 2.007047 | 4.36E-15 | 1.66E-14 |
| RP11-462L8.1 | ENSG00000229656 | 4.017134 | 2.006167 | 5.11E-15 | 1.94E-14 |
| RP11-809C18.3 | ENSG00000225140 | 4.015778 | 2.005679 | 1.42E-10 | 3.84E-10 |
| LINC00165 | ENSG00000261706 | 4.015066 | 2.005424 | 2.31E-09 | 5.62E-09 |
| AC002059.10 | ENSG00000273216 | 4.010034 | 2.003614 | 1.26E-26 | 9.74E-26 |
| AC011286.1 | ENSG00000232328 | 4.009639 | 2.003472 | 9.51E-06 | 1.70E-05 |
| RP1-90G24.11 | ENSG00000273325 | 4.009470 | 2.003412 | 1.11E-15 | 4.41E-15 |
| RP11-455F5.6 | ENSG00000275371 | 4.007350 | 2.002649 | 5.73E-26 | 4.31E-25 |
| RP11-1028N23.4 | ENSG00000257407 | 4.006703 | 2.002416 | 7.71E-07 | 1.52E-06 |
| CTD-3032J10.3 | ENSG00000269480 | 4.003747 | 2.001351 | 1.01E-13 | 3.48E-13 |
| LINC00221 | ENSG00000270816 | 4.001461 | 2.000527 | 1.89E-04 | 2.98E-04 |
| RP11-826N14.2 | ENSG00000248469 | 4.001167 | 2.000421 | 7.01E-13 | 2.26E-12 |
