# Supplementary material for: A Computationally Constructed lncRNA-Associated Competing Triplet Network in Clear Cell Renal Cell Carcinoma
Source: Dis Markers. 2022 Nov 17;2022:8928282. doi: 10.1155/2022/8928282 (PMC9691318; doi:10.1155/2022/8928282)
Supplement: Supplementary Materials — Table S1: the list of upregulated lncRNAs in ccRCC. Table S2: the list of downregulated lncRNAs in ccRCC. Table S3: the list of upregulated mRNAs in ccRCC. Table S4: the list of downregulated mRNAs in ccRCC. Table S5: the list of upregulated miRNAs in ccRCC. Table S6: the list of downregulated miRNAs in ccRCC. Table S7: the list of top 100 dysregulated (50 upregulated and 50 downregulated) lncRNAs in consistent with Figure 1. Table S8: the list of genes coexpressed with HOTTIP in ccRCC. [file 8928282.f1.zip › 8928282.f1/Table S2 (1).docx]

Table S2. The list of down-regulated lncRNAs in ccRCC.

| **Gene symbol** | **Ensemb ID** | **Fold Change (FC)  (T/N)** | **log_2_FC (T/N)** | ***P* value** | **FDR** |
| --- | --- | --- | --- | --- | --- |
| RP11-850F7.7 | ENSG00000257634 | 0.002682 | -8.542613 | 1.15E-199 | 5.45E-197 |
| CTD-2247C11.5 | ENSG00000250529 | 0.003465 | -8.172892 | 1.38E-111 | 1.57E-109 |
| RP11-386B13.4 | ENSG00000248517 | 0.003566 | -8.131515 | 5.94E-195 | 2.67E-192 |
| RP11-476H24.1 | ENSG00000236408 | 0.003635 | -8.103830 | 2.32E-136 | 3.86E-134 |
| RP11-390N6.1 | ENSG00000257191 | 0.005364 | -7.542428 | 3.44E-263 | 6.18E-260 |
| RP11-317M11.1 | ENSG00000249341 | 0.006728 | -7.215664 | 1.38E-173 | 3.75E-171 |
| RP11-531H8.2 | ENSG00000254789 | 0.007294 | -7.099025 | 2.66E-231 | 2.66E-228 |
| LINC01571 | ENSG00000260057 | 0.007556 | -7.048085 | 2.27E-188 | 8.87E-186 |
| RP11-208K4.1 | ENSG00000259754 | 0.008217 | -6.927097 | 3.56E-182 | 1.19E-179 |
| RP11-516J2.1 | ENSG00000258402 | 0.008277 | -6.916751 | 1.02E-186 | 3.66E-184 |
| RP11-116O18.1 | ENSG00000266968 | 0.008812 | -6.826246 | 2.96E-209 | 1.66E-206 |
| LINC01543 | ENSG00000263862 | 0.008833 | -6.822857 | 5.75E-152 | 1.33E-149 |
| RP11-61L19.2 | ENSG00000273335 | 0.008938 | -6.805884 | 0.00E+00 | 0.00E+00 |
| RP11-195B3.1 | ENSG00000227338 | 0.009468 | -6.722788 | 7.78E-181 | 2.50E-178 |
| RP11-142A12.1 | ENSG00000260580 | 0.010000 | -6.643928 | 6.60E-233 | 7.42E-230 |
| LINC01055 | ENSG00000235366 | 0.010302 | -6.600976 | 2.52E-150 | 5.66E-148 |
| LINC01020 | ENSG00000215231 | 0.010370 | -6.591480 | 4.17E-187 | 1.56E-184 |
| AC068138.1 | ENSG00000235070 | 0.010541 | -6.567793 | 7.21E-140 | 1.30E-137 |
| AC124944.5 | ENSG00000223783 | 0.011167 | -6.484667 | 6.48E-122 | 8.70E-120 |
| AC005616.2 | ENSG00000267423 | 0.011719 | -6.414973 | 1.08E-129 | 1.64E-127 |
| LINC01378 | ENSG00000236922 | 0.011734 | -6.413139 | 6.43E-205 | 3.40E-202 |
| AC107057.1 | ENSG00000232835 | 0.011850 | -6.398984 | 2.45E-102 | 2.35E-100 |
| RP11-752D24.2 | ENSG00000248115 | 0.012198 | -6.357185 | 8.90E-221 | 6.16E-218 |
| RP11-575B7.3 | ENSG00000238242 | 0.013252 | -6.237664 | 3.55E-157 | 8.64E-155 |
| RP11-586L23.1 | ENSG00000248747 | 0.013589 | -6.201369 | 1.83E-109 | 2.03E-107 |
| RP11-3G21.1 | ENSG00000254575 | 0.013716 | -6.187997 | 7.13E-122 | 9.44E-120 |
| RP11-527L4.6 | ENSG00000267420 | 0.014085 | -6.149654 | 2.28E-215 | 1.47E-212 |
| AP000696.2 | ENSG00000231324 | 0.016384 | -5.931540 | 2.47E-237 | 3.17E-234 |
| CTA-392C11.1 | ENSG00000253802 | 0.017599 | -5.828375 | 3.97E-75 | 2.03E-73 |
| LINC00864 | ENSG00000228055 | 0.018062 | -5.790862 | 9.07E-150 | 1.90E-147 |
| RP5-1100I6.1 | ENSG00000236151 | 0.018082 | -5.789320 | 1.08E-91 | 8.28E-90 |
| CTD-2007H18.1 | ENSG00000259730 | 0.018423 | -5.762327 | 3.16E-185 | 1.09E-182 |
| RP5-1018K9.1 | ENSG00000237707 | 0.019178 | -5.704410 | 5.96E-281 | 1.34E-277 |
| RP11-527L4.2 | ENSG00000261514 | 0.019393 | -5.688312 | 6.89E-140 | 1.26E-137 |
| RP11-14K3.7 | ENSG00000259791 | 0.020072 | -5.638665 | 5.06E-92 | 3.96E-90 |
| RP11-550H2.2 | ENSG00000230027 | 0.020871 | -5.582370 | 2.68E-112 | 3.13E-110 |
| RP1-137K24.1 | ENSG00000248486 | 0.021221 | -5.558359 | 5.23E-221 | 3.92E-218 |
| LINC00982 | ENSG00000177133 | 0.021746 | -5.523133 | 1.53E-126 | 2.23E-124 |
| RP13-259N13.2 | ENSG00000203565 | 0.022551 | -5.470636 | 1.46E-89 | 1.08E-87 |
| CTD-2008P7.9 | ENSG00000267259 | 0.024963 | -5.324081 | 4.25E-49 | 9.26E-48 |
| BMPR1B-AS1 | ENSG00000249599 | 0.027565 | -5.181009 | 2.09E-100 | 1.94E-98 |
| RP11-310P5.2 | ENSG00000250385 | 0.029104 | -5.102628 | 2.88E-70 | 1.27E-68 |
| RP11-16D22.2 | ENSG00000271850 | 0.029778 | -5.069622 | 3.30E-57 | 9.84E-56 |
| RP11-120I21.2 | ENSG00000254202 | 0.030433 | -5.038231 | 3.09E-62 | 1.10E-60 |
| AC006960.7 | ENSG00000237400 | 0.030605 | -5.030071 | 6.93E-103 | 6.71E-101 |
| LINC01555 | ENSG00000180869 | 0.031018 | -5.010746 | 8.42E-155 | 1.99E-152 |
| RP11-379F12.4 | ENSG00000232638 | 0.031106 | -5.006678 | 6.36E-69 | 2.71E-67 |
| RP4-655J12.4 | ENSG00000233154 | 0.031717 | -4.978605 | 6.82E-304 | 2.05E-300 |
| GATA3-AS1 | ENSG00000197308 | 0.031829 | -4.973510 | 1.39E-70 | 6.25E-69 |
| KB-1562D12.1 | ENSG00000254024 | 0.032053 | -4.963399 | 3.15E-100 | 2.90E-98 |
| RP11-132E11.2 | ENSG00000237153 | 0.032887 | -4.926342 | 7.98E-50 | 1.77E-48 |
| RP11-132N15.3 | ENSG00000224187 | 0.032987 | -4.921961 | 1.01E-64 | 3.92E-63 |
| RP11-89B16.1 | ENSG00000249706 | 0.033520 | -4.898845 | 1.59E-189 | 6.52E-187 |
| AC068535.3 | ENSG00000225765 | 0.033602 | -4.895295 | 3.54E-71 | 1.64E-69 |
| RP11-245J24.1 | ENSG00000238276 | 0.033840 | -4.885128 | 2.40E-62 | 8.64E-61 |
| RP11-63A11.1 | ENSG00000250781 | 0.033977 | -4.879303 | 1.94E-79 | 1.13E-77 |
| CTD-2626G11.2 | ENSG00000268416 | 0.034489 | -4.857730 | 4.80E-68 | 1.97E-66 |
| RP1-38C16.2 | ENSG00000233534 | 0.034582 | -4.853845 | 1.65E-104 | 1.67E-102 |
| RP11-536G4.1 | ENSG00000258292 | 0.034755 | -4.846634 | 1.55E-221 | 1.27E-218 |
| RP11-573D15.8 | ENSG00000197099 | 0.035068 | -4.833717 | 8.17E-224 | 7.35E-221 |
| RP11-714L20.1 | ENSG00000251632 | 0.035136 | -4.830900 | 2.77E-147 | 5.53E-145 |
| AC105398.3 | ENSG00000229224 | 0.035860 | -4.801480 | 5.04E-71 | 2.31E-69 |
| RP11-18H21.3 | ENSG00000251377 | 0.036262 | -4.785398 | 1.55E-159 | 3.87E-157 |
| RP11-545A16.1 | ENSG00000261250 | 0.036452 | -4.777875 | 7.18E-112 | 8.28E-110 |
| HNF4A-AS1 | ENSG00000229005 | 0.037376 | -4.741748 | 1.95E-103 | 1.91E-101 |
| AP000697.6 | ENSG00000224269 | 0.037400 | -4.740818 | 8.28E-82 | 5.29E-80 |
| RP3-429O6.1 | ENSG00000223342 | 0.037997 | -4.717968 | 1.12E-85 | 7.54E-84 |
| WSPAR | ENSG00000249073 | 0.039469 | -4.663122 | 5.56E-193 | 2.38E-190 |
| RP11-35J10.7 | ENSG00000276668 | 0.039681 | -4.655413 | 3.44E-58 | 1.03E-56 |
| RP11-22C11.2 | ENSG00000261437 | 0.039704 | -4.654577 | 1.46E-91 | 1.11E-89 |
| RP1-56K13.5 | ENSG00000266588 | 0.040661 | -4.620221 | 2.02E-176 | 5.87E-174 |
| RP1-58B11.1 | ENSG00000260972 | 0.041522 | -4.589991 | 2.21E-76 | 1.16E-74 |
| LINC00379 | ENSG00000229557 | 0.043921 | -4.508932 | 1.02E-85 | 6.98E-84 |
| RP11-55L3.1 | ENSG00000250791 | 0.044122 | -4.502369 | 3.01E-45 | 5.71E-44 |
| RP11-21A7A.2 | ENSG00000256824 | 0.045953 | -4.443693 | 1.29E-97 | 1.14E-95 |
| RP11-35J10.6 | ENSG00000275612 | 0.046109 | -4.438794 | 2.03E-51 | 4.79E-50 |
| F11-AS1 | ENSG00000251165 | 0.046159 | -4.437233 | 1.79E-108 | 1.94E-106 |
| LINC00645 | ENSG00000258548 | 0.046982 | -4.411760 | 2.40E-52 | 5.88E-51 |
| AC099552.3 | ENSG00000228521 | 0.048340 | -4.370633 | 5.74E-31 | 5.56E-30 |
| RP11-445F12.1 | ENSG00000277268 | 0.050443 | -4.309208 | 4.82E-46 | 9.41E-45 |
| RP11-469H8.6 | ENSG00000257588 | 0.051730 | -4.272854 | 6.75E-33 | 7.17E-32 |
| NPSR1-AS1 | ENSG00000197085 | 0.052639 | -4.247736 | 1.40E-60 | 4.69E-59 |
| RP11-359N11.1 | ENSG00000230631 | 0.052727 | -4.245321 | 5.27E-70 | 2.31E-68 |
| RP11-320H14.1 | ENSG00000260878 | 0.053026 | -4.237162 | 9.67E-29 | 8.44E-28 |
| SSTR5-AS1 | ENSG00000261713 | 0.053870 | -4.214385 | 4.67E-64 | 1.76E-62 |
| RP11-622A1.2 | ENSG00000250436 | 0.054673 | -4.193015 | 3.84E-62 | 1.36E-60 |
| AC099552.4 | ENSG00000217825 | 0.055036 | -4.183481 | 2.11E-56 | 6.14E-55 |
| LINC01544 | ENSG00000260440 | 0.055452 | -4.172627 | 9.55E-60 | 3.11E-58 |
| AC003090.1 | ENSG00000223561 | 0.056038 | -4.157453 | 3.20E-160 | 8.23E-158 |
| SCHLAP1 | ENSG00000281131 | 0.056112 | -4.155543 | 1.59E-65 | 6.29E-64 |
| LINC00551 | ENSG00000272274 | 0.056668 | -4.141312 | 2.33E-139 | 4.03E-137 |
| RP11-457K10.1 | ENSG00000242029 | 0.056994 | -4.133048 | 9.38E-80 | 5.52E-78 |
| CTB-79E8.2 | ENSG00000253445 | 0.058408 | -4.097695 | 1.89E-121 | 2.46E-119 |
| RP1-80N2.2 | ENSG00000226281 | 0.058593 | -4.093138 | 9.50E-86 | 6.52E-84 |
| PROX1-AS1 | ENSG00000230461 | 0.059168 | -4.079038 | 4.10E-62 | 1.45E-60 |
| AC012123.1 | ENSG00000228835 | 0.060383 | -4.049721 | 5.25E-122 | 7.16E-120 |
| FAM167A-AS1 | ENSG00000184608 | 0.060444 | -4.048263 | 1.44E-80 | 8.74E-79 |
| RP11-21A7A.4 | ENSG00000256481 | 0.060538 | -4.046008 | 6.64E-61 | 2.25E-59 |
| FOXCUT | ENSG00000280916 | 0.060870 | -4.038128 | 5.01E-150 | 1.07E-147 |
| RP11-415C15.1 | ENSG00000251080 | 0.061774 | -4.016861 | 3.51E-78 | 1.95E-76 |
| RP1-81D8.3 | ENSG00000224477 | 0.062935 | -3.990003 | 2.44E-50 | 5.51E-49 |
| AL161668.5 | ENSG00000258604 | 0.064500 | -3.954561 | 2.48E-70 | 1.11E-68 |
| RP11-90P13.1 | ENSG00000261795 | 0.064809 | -3.947673 | 3.28E-129 | 4.92E-127 |
| RP11-128L5.1 | ENSG00000253266 | 0.064944 | -3.944665 | 1.35E-139 | 2.38E-137 |
| RP11-46C24.3 | ENSG00000205015 | 0.065406 | -3.934428 | 3.02E-42 | 5.14E-41 |
| AC074286.1 | ENSG00000213963 | 0.065570 | -3.930829 | 0.00E+00 | 0.00E+00 |
| RP11-95P13.2 | ENSG00000238232 | 0.065799 | -3.925801 | 3.09E-44 | 5.60E-43 |
| PWRN3 | ENSG00000260760 | 0.066681 | -3.906581 | 1.99E-57 | 5.96E-56 |
| RP11-128P17.2 | ENSG00000267051 | 0.067899 | -3.880471 | 3.05E-42 | 5.18E-41 |
| RP4-735C1.4 | ENSG00000241720 | 0.069680 | -3.843111 | 6.77E-142 | 1.30E-139 |
| RP11-115H13.1 | ENSG00000273906 | 0.069885 | -3.838876 | 5.74E-46 | 1.11E-44 |
| AC019181.2 | ENSG00000233255 | 0.070182 | -3.832763 | 3.87E-65 | 1.52E-63 |
| RP11-816J8.1 | ENSG00000258868 | 0.070361 | -3.829088 | 1.08E-108 | 1.19E-106 |
| RP11-494M8.4 | ENSG00000254951 | 0.070588 | -3.824426 | 1.68E-93 | 1.35E-91 |
| CTD-2008P7.8 | ENSG00000266830 | 0.071041 | -3.815197 | 2.84E-40 | 4.41E-39 |
| SRGAP3-AS4 | ENSG00000235830 | 0.071109 | -3.813826 | 1.72E-99 | 1.55E-97 |
| FAM215A | ENSG00000267496 | 0.071384 | -3.808260 | 3.16E-178 | 9.47E-176 |
| RP11-128P17.1 | ENSG00000267425 | 0.071857 | -3.798737 | 1.06E-35 | 1.32E-34 |
| RP4-547N15.3 | ENSG00000275678 | 0.072445 | -3.786967 | 1.82E-82 | 1.18E-80 |
| RP11-108E14.1 | ENSG00000253300 | 0.072720 | -3.781496 | 1.06E-57 | 3.17E-56 |
| LINC00473 | ENSG00000223414 | 0.073019 | -3.775584 | 4.49E-48 | 9.54E-47 |
| RP5-1024C24.1 | ENSG00000254489 | 0.073870 | -3.758865 | 8.87E-46 | 1.72E-44 |
| RP11-470M17.2 | ENSG00000254143 | 0.073941 | -3.757472 | 1.35E-86 | 9.49E-85 |
| LINC00871 | ENSG00000258700 | 0.074200 | -3.752441 | 1.11E-28 | 9.62E-28 |
| RP11-74M11.2 | ENSG00000248300 | 0.074284 | -3.750813 | 6.33E-94 | 5.18E-92 |
| RP3-380B4.1 | ENSG00000229639 | 0.074457 | -3.747446 | 2.77E-67 | 1.12E-65 |
| LINC00461 | ENSG00000245526 | 0.074589 | -3.744892 | 6.98E-56 | 1.96E-54 |
| LINC01351 | ENSG00000237457 | 0.075343 | -3.730384 | 3.64E-40 | 5.57E-39 |
| RP11-131N11.4 | ENSG00000254271 | 0.075417 | -3.728969 | 2.98E-125 | 4.20E-123 |
| RP11-354P11.2 | ENSG00000266120 | 0.075803 | -3.721601 | 6.86E-61 | 2.31E-59 |
| PTCSC3 | ENSG00000259104 | 0.075919 | -3.719403 | 1.57E-55 | 4.34E-54 |
| RP11-61O1.1 | ENSG00000259097 | 0.076703 | -3.704569 | 4.31E-95 | 3.63E-93 |
| RP11-476M19.2 | ENSG00000257048 | 0.076714 | -3.704362 | 1.03E-54 | 2.82E-53 |
| RP11-999E24.3 | ENSG00000259969 | 0.076929 | -3.700325 | 4.19E-150 | 9.21E-148 |
| RP11-128P17.4 | ENSG00000273119 | 0.077599 | -3.687825 | 4.25E-27 | 3.38E-26 |
| RP11-536I6.2 | ENSG00000255021 | 0.078291 | -3.675002 | 1.01E-47 | 2.11E-46 |
| RP11-433J22.3 | ENSG00000234190 | 0.080271 | -3.638974 | 1.19E-65 | 4.73E-64 |
| RP3-523K23.2 | ENSG00000261116 | 0.080899 | -3.627740 | 3.43E-37 | 4.60E-36 |
| AC016735.2 | ENSG00000231826 | 0.081045 | -3.625133 | 6.99E-96 | 5.93E-94 |
| LINC01598 | ENSG00000250432 | 0.084473 | -3.565362 | 6.59E-238 | 9.89E-235 |
| RP4-568C11.4 | ENSG00000274173 | 0.084603 | -3.563141 | 1.39E-31 | 1.39E-30 |
| RP11-528A4.2 | ENSG00000229155 | 0.085350 | -3.550459 | 3.71E-96 | 3.18E-94 |
| LINC00410 | ENSG00000231674 | 0.086548 | -3.530359 | 4.83E-43 | 8.32E-42 |
| CTD-2004A9.1 | ENSG00000250885 | 0.086858 | -3.525205 | 9.40E-47 | 1.89E-45 |
| RP11-536C5.2 | ENSG00000225279 | 0.086902 | -3.524469 | 4.91E-136 | 7.90E-134 |
| RP11-451B8.1 | ENSG00000239572 | 0.087281 | -3.518185 | 9.16E-105 | 9.36E-103 |
| RP11-266E6.3 | ENSG00000271901 | 0.087748 | -3.510487 | 2.21E-35 | 2.71E-34 |
| RP11-5P4.3 | ENSG00000229225 | 0.088067 | -3.505254 | 3.45E-15 | 1.33E-14 |
| AC004901.1 | ENSG00000232072 | 0.088485 | -3.498430 | 5.68E-52 | 1.39E-50 |
| RP11-113O24.3 | ENSG00000223716 | 0.088518 | -3.497886 | 1.76E-53 | 4.58E-52 |
| CHL1-AS2 | ENSG00000224318 | 0.088953 | -3.490809 | 8.40E-26 | 6.26E-25 |
| RP11-25E2.1 | ENSG00000257958 | 0.089563 | -3.480950 | 4.48E-86 | 3.10E-84 |
| LINC00443 | ENSG00000230156 | 0.090105 | -3.472252 | 3.88E-136 | 6.34E-134 |
| RP11-321G12.1 | ENSG00000259459 | 0.090579 | -3.464674 | 1.49E-178 | 4.64E-176 |
| LINC00845 | ENSG00000227244 | 0.090864 | -3.460151 | 1.36E-75 | 7.05E-74 |
| CTB-1I21.1 | ENSG00000250284 | 0.091224 | -3.454445 | 2.33E-25 | 1.68E-24 |
| AC011239.1 | ENSG00000224361 | 0.091656 | -3.447628 | 9.88E-59 | 3.08E-57 |
| RP11-754N21.1 | ENSG00000258084 | 0.091918 | -3.443502 | 6.31E-43 | 1.08E-41 |
| AP000345.1 | ENSG00000178248 | 0.092947 | -3.427454 | 1.28E-67 | 5.25E-66 |
| RP11-211N11.5 | ENSG00000234393 | 0.093641 | -3.416715 | 8.72E-200 | 4.36E-197 |
| RP11-94C24.13 | ENSG00000275897 | 0.093793 | -3.414384 | 9.12E-136 | 1.44E-133 |
| RP11-44F14.2 | ENSG00000261804 | 0.094432 | -3.404586 | 1.07E-43 | 1.90E-42 |
| RP11-478J18.2 | ENSG00000274895 | 0.094666 | -3.401012 | 4.33E-31 | 4.21E-30 |
| C15orf56 | ENSG00000176753 | 0.094674 | -3.400887 | 1.18E-53 | 3.08E-52 |
| LINC00380 | ENSG00000234625 | 0.094879 | -3.397760 | 4.17E-63 | 1.53E-61 |
| CTC-340D7.1 | ENSG00000249335 | 0.095422 | -3.389530 | 1.69E-80 | 1.02E-78 |
| RP5-881L22.6 | ENSG00000233376 | 0.095734 | -3.384824 | 7.26E-47 | 1.47E-45 |
| RP11-180C16.1 | ENSG00000273489 | 0.095756 | -3.384493 | 4.61E-213 | 2.76E-210 |
| RP11-879F14.1 | ENSG00000267175 | 0.096279 | -3.376633 | 7.06E-72 | 3.29E-70 |
| RP4-655J12.5 | ENSG00000230381 | 0.096749 | -3.369611 | 2.60E-89 | 1.89E-87 |
| RP3-368B9.2 | ENSG00000250681 | 0.097338 | -3.360850 | 1.44E-47 | 2.99E-46 |
| RP11-734K21.5 | ENSG00000261104 | 0.097909 | -3.352411 | 1.50E-22 | 9.16E-22 |
| RP11-366L20.2 | ENSG00000197301 | 0.098975 | -3.336799 | 2.23E-58 | 6.75E-57 |
| LINC00955 | ENSG00000216560 | 0.099748 | -3.325565 | 6.50E-41 | 1.04E-39 |
| AC103563.8 | ENSG00000233850 | 0.099913 | -3.323182 | 2.32E-61 | 8.04E-60 |
| RRS1-AS1 | ENSG00000246145 | 0.100664 | -3.312385 | 7.16E-126 | 1.02E-123 |
| NAV2-AS3 | ENSG00000254542 | 0.101325 | -3.302934 | 7.98E-48 | 1.67E-46 |
| LINC00284 | ENSG00000233725 | 0.102715 | -3.283280 | 3.99E-42 | 6.73E-41 |
| RP11-475O23.2 | ENSG00000243384 | 0.103239 | -3.275938 | 4.47E-77 | 2.38E-75 |
| RP11-146N18.1 | ENSG00000267134 | 0.103545 | -3.271666 | 7.04E-20 | 3.71E-19 |
| RP11-563M4.1 | ENSG00000249752 | 0.103558 | -3.271489 | 2.21E-68 | 9.21E-67 |
| AE000662.93 | ENSG00000259054 | 0.103909 | -3.266614 | 1.03E-87 | 7.38E-86 |
| RP11-238K6.1 | ENSG00000253288 | 0.105061 | -3.250697 | 6.65E-61 | 2.25E-59 |
| RP11-706C16.7 | ENSG00000253196 | 0.105110 | -3.250022 | 6.25E-40 | 9.46E-39 |
| CTD-2315E11.1 | ENSG00000259685 | 0.106034 | -3.237403 | 1.72E-32 | 1.79E-31 |
| RP11-339D23.1 | ENSG00000225598 | 0.106160 | -3.235682 | 1.18E-24 | 8.14E-24 |
| RP11-794G24.1 | ENSG00000256443 | 0.106454 | -3.231705 | 3.40E-57 | 1.01E-55 |
| FAM230C | ENSG00000279516 | 0.107186 | -3.221813 | 2.89E-20 | 1.55E-19 |
| RP11-567M16.2 | ENSG00000267409 | 0.107330 | -3.219880 | 5.56E-46 | 1.08E-44 |
| RP11-77I22.2 | ENSG00000246331 | 0.108231 | -3.207814 | 2.47E-98 | 2.20E-96 |
| RP5-881L22.5 | ENSG00000226812 | 0.108651 | -3.202231 | 7.50E-32 | 7.57E-31 |
| CLDN10-AS1 | ENSG00000223392 | 0.108902 | -3.198898 | 7.99E-108 | 8.46E-106 |
| RP11-728F11.4 | ENSG00000254528 | 0.109917 | -3.185514 | 8.53E-74 | 4.17E-72 |
| RP11-360O19.4 | ENSG00000237685 | 0.110945 | -3.172083 | 9.06E-59 | 2.84E-57 |
| RP11-392O17.1 | ENSG00000228536 | 0.110961 | -3.171870 | 5.50E-69 | 2.36E-67 |
| RP11-641D5.2 | ENSG00000241479 | 0.112343 | -3.154013 | 5.27E-75 | 2.66E-73 |
| RP1-154K9.2 | ENSG00000231772 | 0.112441 | -3.152757 | 2.94E-34 | 3.39E-33 |
| AC013463.2 | ENSG00000236283 | 0.113145 | -3.143756 | 3.67E-74 | 1.81E-72 |
| AC005082.12 | ENSG00000226816 | 0.113173 | -3.143394 | 3.10E-56 | 8.88E-55 |
| RP11-66B24.5 | ENSG00000259579 | 0.114078 | -3.131903 | 1.39E-78 | 7.84E-77 |
| LINC01606 | ENSG00000253301 | 0.114126 | -3.131296 | 1.46E-15 | 5.75E-15 |
| LINC00602 | ENSG00000281832 | 0.114470 | -3.126955 | 4.07E-32 | 4.18E-31 |
| RP11-393N21.2 | ENSG00000235200 | 0.114884 | -3.121753 | 1.23E-52 | 3.06E-51 |
| LINC00864 | ENSG00000264404 | 0.115818 | -3.110069 | 1.60E-34 | 1.87E-33 |
| AC024592.9 | ENSG00000267709 | 0.115864 | -3.109496 | 3.39E-49 | 7.40E-48 |
| LINC01561 | ENSG00000177234 | 0.115881 | -3.109285 | 3.53E-35 | 4.25E-34 |
| PLS3-AS1 | ENSG00000271826 | 0.116855 | -3.097205 | 7.84E-145 | 1.53E-142 |
| RP11-120K24.3 | ENSG00000267868 | 0.117257 | -3.092249 | 8.10E-51 | 1.87E-49 |
| AC010884.1 | ENSG00000224509 | 0.117302 | -3.091698 | 3.64E-110 | 4.10E-108 |
| RP11-44F14.8 | ENSG00000262714 | 0.118982 | -3.071185 | 2.16E-38 | 3.04E-37 |
| TARID | ENSG00000227954 | 0.119348 | -3.066749 | 1.91E-121 | 2.46E-119 |
| RP11-362F19.1 | ENSG00000248810 | 0.119988 | -3.059034 | 5.17E-78 | 2.86E-76 |
| AC099684.1 | ENSG00000228133 | 0.119991 | -3.059006 | 5.18E-32 | 5.27E-31 |
| XXbac-BPG254F23.7 | ENSG00000232080 | 0.119992 | -3.058989 | 7.74E-14 | 2.70E-13 |
| RP1-78O14.1 | ENSG00000257894 | 0.121191 | -3.044648 | 4.12E-75 | 2.10E-73 |
| LINC00051 | ENSG00000254008 | 0.121404 | -3.042112 | 2.21E-32 | 2.29E-31 |
| RP4-737E23.2 | ENSG00000230387 | 0.121491 | -3.041081 | 1.26E-55 | 3.51E-54 |
| RP13-895J2.6 | ENSG00000277011 | 0.121732 | -3.038221 | 3.95E-23 | 2.48E-22 |
| PCAT14 | ENSG00000280623 | 0.122323 | -3.031229 | 1.44E-27 | 1.17E-26 |
| AC124861.1 | ENSG00000227479 | 0.122576 | -3.028249 | 1.90E-56 | 5.57E-55 |
| LINC01612 | ENSG00000250266 | 0.122856 | -3.024961 | 1.49E-14 | 5.49E-14 |
| RP11-384F7.2 | ENSG00000239268 | 0.123056 | -3.022617 | 1.04E-17 | 4.72E-17 |
| RP11-31F19.1 | ENSG00000226403 | 0.123273 | -3.020075 | 2.01E-68 | 8.42E-67 |
| AC005281.2 | ENSG00000225606 | 0.124674 | -3.003771 | 2.75E-46 | 5.46E-45 |
| RP11-89K21.1 | ENSG00000259439 | 0.124722 | -3.003216 | 2.20E-34 | 2.56E-33 |
| TCL6 | ENSG00000187621 | 0.124722 | -3.003208 | 5.68E-36 | 7.26E-35 |
| AC096574.5 | ENSG00000227107 | 0.124844 | -3.001804 | 2.83E-127 | 4.18E-125 |
| AC011516.2 | ENSG00000268038 | 0.127481 | -2.971644 | 3.80E-14 | 1.36E-13 |
| CTD-2227I18.1 | ENSG00000249236 | 0.127574 | -2.970597 | 1.24E-56 | 3.66E-55 |
| RP11-524H19.2 | ENSG00000224984 | 0.127974 | -2.966074 | 9.76E-16 | 3.90E-15 |
| AC022431.3 | ENSG00000234553 | 0.128224 | -2.963261 | 1.89E-64 | 7.23E-63 |
| RP11-675F6.3 | ENSG00000253361 | 0.129220 | -2.952102 | 1.15E-39 | 1.71E-38 |
| LINC01497 | ENSG00000237560 | 0.129481 | -2.949185 | 1.79E-25 | 1.30E-24 |
| AC026167.1 | ENSG00000226022 | 0.130352 | -2.939516 | 1.04E-24 | 7.21E-24 |
| RP11-363J20.1 | ENSG00000258957 | 0.130355 | -2.939479 | 2.38E-174 | 6.69E-172 |
| RP11-554A11.4 | ENSG00000261625 | 0.131991 | -2.921489 | 2.42E-64 | 9.22E-63 |
| COL18A1-AS1 | ENSG00000183535 | 0.132554 | -2.915353 | 1.17E-77 | 6.36E-76 |
| LINC01224 | ENSG00000269416 | 0.133437 | -2.905765 | 2.15E-51 | 5.06E-50 |
| SEMA3B-AS1 | ENSG00000232352 | 0.133723 | -2.902679 | 4.54E-50 | 1.02E-48 |
| HOXB-AS3 | ENSG00000233101 | 0.134365 | -2.895772 | 3.04E-68 | 1.26E-66 |
| RP1-168L15.5 | ENSG00000261420 | 0.134912 | -2.889913 | 3.80E-138 | 6.44E-136 |
| AC093326.1 | ENSG00000223985 | 0.135079 | -2.888127 | 4.52E-19 | 2.25E-18 |
| RP11-354K4.2 | ENSG00000271945 | 0.137325 | -2.864329 | 6.37E-15 | 2.40E-14 |
| WT1-AS | ENSG00000183242 | 0.139101 | -2.845800 | 1.00E-34 | 1.18E-33 |
| LY86-AS1 | ENSG00000216863 | 0.140158 | -2.834878 | 6.63E-70 | 2.90E-68 |
| RP11-734K21.2 | ENSG00000242136 | 0.140615 | -2.830177 | 8.77E-14 | 3.05E-13 |
| FAM3D-AS1 | ENSG00000244383 | 0.140820 | -2.828075 | 4.41E-49 | 9.59E-48 |
| RP11-659E9.2 | ENSG00000240915 | 0.140895 | -2.827312 | 1.91E-37 | 2.59E-36 |
| RP11-486L19.2 | ENSG00000250685 | 0.141316 | -2.823003 | 1.45E-46 | 2.90E-45 |
| RP11-78C3.1 | ENSG00000248112 | 0.141377 | -2.822380 | 6.69E-35 | 7.98E-34 |
| CTA-398F10.2 | ENSG00000254153 | 0.142725 | -2.808692 | 3.26E-25 | 2.33E-24 |
| RP11-142G1.3 | ENSG00000260975 | 0.142914 | -2.806783 | 1.45E-15 | 5.71E-15 |
| RP11-482D24.3 | ENSG00000257918 | 0.142915 | -2.806774 | 7.40E-117 | 9.00E-115 |
| LINC01517 | ENSG00000232624 | 0.143041 | -2.805496 | 8.98E-36 | 1.13E-34 |
| RP11-370F5.4 | ENSG00000203364 | 0.143528 | -2.800599 | 1.31E-63 | 4.87E-62 |
| RP11-552E20.1 | ENSG00000227131 | 0.144257 | -2.793289 | 1.97E-33 | 2.17E-32 |
| AC103563.9 | ENSG00000231062 | 0.145364 | -2.782257 | 1.34E-34 | 1.57E-33 |
| AC002401.1 | ENSG00000236472 | 0.145699 | -2.778939 | 1.07E-39 | 1.60E-38 |
| MYCNOS | ENSG00000233718 | 0.146617 | -2.769880 | 4.56E-56 | 1.30E-54 |
| AC093802.1 | ENSG00000220256 | 0.147647 | -2.759776 | 2.38E-33 | 2.60E-32 |
| PWRN1 | ENSG00000259905 | 0.149253 | -2.744165 | 2.60E-29 | 2.35E-28 |
| RP11-480D4.2 | ENSG00000249159 | 0.149927 | -2.737671 | 4.72E-104 | 4.72E-102 |
| RP11-404P21.3 | ENSG00000258793 | 0.150545 | -2.731729 | 6.12E-30 | 5.66E-29 |
| CTD-2515H24.2 | ENSG00000276772 | 0.150744 | -2.729832 | 3.50E-85 | 2.35E-83 |
| RP4-710M3.2 | ENSG00000255480 | 0.150800 | -2.729289 | 1.16E-16 | 4.91E-16 |
| RP1-67A8.3 | ENSG00000234206 | 0.150862 | -2.728694 | 7.99E-50 | 1.77E-48 |
| LINC00343 | ENSG00000226620 | 0.151302 | -2.724497 | 1.89E-35 | 2.33E-34 |
| PGM5-AS1 | ENSG00000225655 | 0.151400 | -2.723562 | 9.72E-30 | 8.91E-29 |
| AC007255.8 | ENSG00000223813 | 0.151412 | -2.723452 | 3.07E-90 | 2.31E-88 |
| RP11-554A11.9 | ENSG00000259799 | 0.151664 | -2.721048 | 3.46E-79 | 1.99E-77 |
| RP5-1065P14.2 | ENSG00000236975 | 0.151741 | -2.720319 | 1.18E-32 | 1.24E-31 |
| VWA8-AS1 | ENSG00000278338 | 0.152293 | -2.715079 | 3.68E-121 | 4.66E-119 |
| RP11-373E16.3 | ENSG00000244738 | 0.152558 | -2.712571 | 3.46E-30 | 3.26E-29 |
| RP11-445F12.2 | ENSG00000276707 | 0.153266 | -2.705889 | 1.01E-16 | 4.29E-16 |
| RP11-311F12.1 | ENSG00000262681 | 0.153686 | -2.701942 | 2.00E-66 | 8.02E-65 |
| LINC00237 | ENSG00000225127 | 0.153707 | -2.701747 | 1.12E-40 | 1.78E-39 |
| AC144831.3 | ENSG00000274370 | 0.154031 | -2.698707 | 3.07E-72 | 1.45E-70 |
| AC144831.1 | ENSG00000261888 | 0.154487 | -2.694439 | 6.18E-76 | 3.23E-74 |
| AC005301.8 | ENSG00000235343 | 0.154660 | -2.692828 | 3.26E-11 | 9.35E-11 |
| AC027119.1 | ENSG00000229642 | 0.156391 | -2.676770 | 5.79E-17 | 2.49E-16 |
| LINC01589 | ENSG00000238120 | 0.156450 | -2.676230 | 5.60E-41 | 9.00E-40 |
| RP11-671P2.1 | ENSG00000264263 | 0.157085 | -2.670387 | 3.51E-48 | 7.48E-47 |
| RP11-509A17.3 | ENSG00000258654 | 0.157540 | -2.666214 | 3.36E-37 | 4.52E-36 |
| MCF2L-AS1 | ENSG00000235280 | 0.157898 | -2.662938 | 3.10E-50 | 6.97E-49 |
| TRPC7-AS2 | ENSG00000250947 | 0.158538 | -2.657096 | 1.01E-28 | 8.74E-28 |
| RP11-2E11.5 | ENSG00000259920 | 0.159194 | -2.651139 | 4.11E-48 | 8.75E-47 |
| RP4-541C22.5 | ENSG00000255202 | 0.159385 | -2.649413 | 7.47E-38 | 1.02E-36 |
| RP1-206D15.6 | ENSG00000213062 | 0.159970 | -2.644131 | 2.49E-67 | 1.01E-65 |
| RP11-536G4.2 | ENSG00000258343 | 0.160109 | -2.642878 | 1.49E-62 | 5.40E-61 |
| TSSC1-IT1 | ENSG00000224885 | 0.161495 | -2.630437 | 1.38E-51 | 3.30E-50 |
| CTD-2377O17.1 | ENSG00000271714 | 0.161596 | -2.629538 | 5.92E-13 | 1.92E-12 |
| SPATA13 | ENSG00000228741 | 0.161623 | -2.629294 | 2.37E-81 | 1.48E-79 |
| LMO7-AS1 | ENSG00000261105 | 0.161845 | -2.627314 | 2.62E-62 | 9.39E-61 |
| RP3-466P17.1 | ENSG00000270638 | 0.162977 | -2.617256 | 4.63E-149 | 9.48E-147 |
| LA16c-329F2.1 | ENSG00000261399 | 0.163277 | -2.614609 | 1.64E-27 | 1.33E-26 |
| CTC-273B12.10 | ENSG00000269814 | 0.163477 | -2.612837 | 4.04E-38 | 5.62E-37 |
| RP11-587P21.2 | ENSG00000257893 | 0.163823 | -2.609791 | 1.86E-17 | 8.31E-17 |
| PP7080 | ENSG00000188242 | 0.163903 | -2.609081 | 6.11E-95 | 5.10E-93 |
| AC093627.11 | ENSG00000239715 | 0.163976 | -2.608446 | 3.80E-17 | 1.66E-16 |
| RP13-650J16.1 | ENSG00000264569 | 0.164079 | -2.607536 | 4.20E-20 | 2.23E-19 |
| RP11-297L17.2 | ENSG00000260963 | 0.164089 | -2.607452 | 4.75E-50 | 1.06E-48 |
| RP4-813D12.3 | ENSG00000226308 | 0.164141 | -2.606995 | 1.18E-22 | 7.21E-22 |
| LYPLAL1-AS1 | ENSG00000228063 | 0.164265 | -2.605903 | 4.48E-121 | 5.60E-119 |
| RP11-10L7.1 | ENSG00000246375 | 0.165047 | -2.599054 | 2.80E-62 | 9.99E-61 |
| LINC01159 | ENSG00000229743 | 0.165313 | -2.596727 | 6.76E-106 | 6.99E-104 |
| AC025811.3 | ENSG00000270947 | 0.165346 | -2.596436 | 4.58E-23 | 2.88E-22 |
| RP11-480D4.1 | ENSG00000245729 | 0.166795 | -2.583851 | 3.24E-29 | 2.90E-28 |
| RP11-554A11.5 | ENSG00000261276 | 0.167040 | -2.581733 | 2.32E-30 | 2.20E-29 |
| RP11-396O20.1 | ENSG00000254695 | 0.167952 | -2.573882 | 2.91E-21 | 1.67E-20 |
| RP11-5P4.1 | ENSG00000229537 | 0.168610 | -2.568239 | 8.23E-10 | 2.09E-09 |
| UCA1 | ENSG00000214049 | 0.168678 | -2.567658 | 1.71E-18 | 8.18E-18 |
| RP11-531A24.3 | ENSG00000260838 | 0.170550 | -2.551730 | 4.65E-20 | 2.47E-19 |
| RP11-390E23.3 | ENSG00000230442 | 0.170946 | -2.548387 | 2.52E-25 | 1.81E-24 |
| LINC01031 | ENSG00000232077 | 0.171122 | -2.546907 | 3.41E-51 | 7.97E-50 |
| RP11-25G10.2 | ENSG00000232739 | 0.171241 | -2.545898 | 2.57E-101 | 2.41E-99 |
| RP11-758N13.1 | ENSG00000259721 | 0.171244 | -2.545877 | 4.88E-18 | 2.27E-17 |
| CTC-498J12.1 | ENSG00000250237 | 0.171320 | -2.545238 | 5.51E-49 | 1.19E-47 |
| MESTIT1 | ENSG00000272701 | 0.171370 | -2.544816 | 7.17E-39 | 1.03E-37 |
| LINC01317 | ENSG00000203386 | 0.171714 | -2.541921 | 1.99E-58 | 6.05E-57 |
| CALML3-AS1 | ENSG00000205488 | 0.172848 | -2.532421 | 1.18E-40 | 1.87E-39 |
| RP11-881M11.1 | ENSG00000256944 | 0.173018 | -2.531007 | 5.87E-35 | 7.02E-34 |
| RP11-565P22.2 | ENSG00000227094 | 0.173024 | -2.530952 | 1.65E-18 | 7.91E-18 |
| AP001046.6 | ENSG00000225637 | 0.173128 | -2.530090 | 7.40E-26 | 5.53E-25 |
| AC026471.6 | ENSG00000260740 | 0.175245 | -2.512554 | 2.55E-39 | 3.71E-38 |
| RP11-725G5.2 | ENSG00000258630 | 0.175786 | -2.508111 | 1.14E-52 | 2.83E-51 |
| UG0898H09 | ENSG00000274956 | 0.176994 | -2.498227 | 1.79E-16 | 7.46E-16 |
| RP11-285G1.9 | ENSG00000223462 | 0.177390 | -2.495002 | 8.80E-53 | 2.21E-51 |
| RP11-12L8.1 | ENSG00000228127 | 0.177971 | -2.490285 | 5.35E-42 | 8.98E-41 |
| RP11-95P13.1 | ENSG00000230024 | 0.178552 | -2.485586 | 2.03E-51 | 4.79E-50 |
| CTD-2023N9.1 | ENSG00000250961 | 0.179011 | -2.481879 | 5.50E-31 | 5.33E-30 |
| AC004066.3 | ENSG00000250522 | 0.179806 | -2.475485 | 6.45E-30 | 5.94E-29 |
| RP4-684O24.5 | ENSG00000233896 | 0.180013 | -2.473827 | 2.84E-35 | 3.44E-34 |
| RP11-456H18.2 | ENSG00000229896 | 0.180357 | -2.471076 | 1.98E-79 | 1.14E-77 |
| DLG1-AS1 | ENSG00000227375 | 0.180610 | -2.469052 | 7.41E-61 | 2.49E-59 |
| RP11-511B23.2 | ENSG00000257322 | 0.181538 | -2.461654 | 1.92E-82 | 1.24E-80 |
| CTB-107G13.1 | ENSG00000234715 | 0.182070 | -2.457438 | 5.02E-40 | 7.62E-39 |
| RP11-254F7.3 | ENSG00000271787 | 0.182373 | -2.455037 | 5.34E-80 | 3.16E-78 |
| AC090505.6 | ENSG00000237222 | 0.182942 | -2.450538 | 5.55E-29 | 4.90E-28 |
| RP1-167O22.1 | ENSG00000279082 | 0.183220 | -2.448348 | 2.55E-38 | 3.56E-37 |
| RP11-626P14.1 | ENSG00000257845 | 0.183461 | -2.446452 | 1.51E-18 | 7.28E-18 |
| AC013460.1 | ENSG00000229727 | 0.183463 | -2.446437 | 2.55E-58 | 7.71E-57 |
| AP000344.3 | ENSG00000234928 | 0.184385 | -2.439206 | 6.47E-33 | 6.89E-32 |
| LINC00472 | ENSG00000233237 | 0.184669 | -2.436983 | 3.73E-119 | 4.60E-117 |
| CTD-2297D10.1 | ENSG00000250866 | 0.184831 | -2.435721 | 1.42E-12 | 4.49E-12 |
| RP11-643M14.1 | ENSG00000259251 | 0.185471 | -2.430737 | 1.20E-103 | 1.19E-101 |
| LINC00507 | ENSG00000256193 | 0.185899 | -2.427409 | 6.53E-12 | 1.97E-11 |
| RP13-616I3.1 | ENSG00000272989 | 0.185996 | -2.426654 | 8.75E-44 | 1.56E-42 |
| LINC01621 | ENSG00000235357 | 0.187348 | -2.416207 | 1.92E-30 | 1.82E-29 |
| RP11-700H6.2 | ENSG00000251665 | 0.187378 | -2.415978 | 1.51E-32 | 1.57E-31 |
| RP13-895J2.3 | ENSG00000256542 | 0.187628 | -2.414051 | 4.18E-28 | 3.51E-27 |
| RP11-675F6.4 | ENSG00000254100 | 0.188176 | -2.409849 | 6.36E-24 | 4.20E-23 |
| RP11-482D24.2 | ENSG00000257711 | 0.190530 | -2.391909 | 1.03E-53 | 2.71E-52 |
| SRGAP3-AS2 | ENSG00000228723 | 0.191714 | -2.382970 | 6.34E-19 | 3.13E-18 |
| RP4-555D20.4 | ENSG00000272121 | 0.192536 | -2.376798 | 1.73E-13 | 5.87E-13 |
| CTB-49A3.4 | ENSG00000250244 | 0.192644 | -2.375988 | 8.66E-10 | 2.19E-09 |
| RP11-834C11.4 | ENSG00000250742 | 0.193147 | -2.372232 | 4.21E-97 | 3.68E-95 |
| LINC01510 | ENSG00000231210 | 0.193476 | -2.369775 | 2.78E-31 | 2.73E-30 |
| AC004862.6 | ENSG00000232667 | 0.194008 | -2.365813 | 4.22E-14 | 1.50E-13 |
| RP11-480D4.6 | ENSG00000272049 | 0.194053 | -2.365475 | 1.36E-79 | 7.92E-78 |
| ARHGEF26-AS1 | ENSG00000243069 | 0.194636 | -2.361150 | 1.50E-38 | 2.12E-37 |
| CTD-2545H1.2 | ENSG00000262445 | 0.195233 | -2.356728 | 1.08E-26 | 8.40E-26 |
| RP11-554L12.1 | ENSG00000258144 | 0.195505 | -2.354725 | 6.15E-16 | 2.49E-15 |
| RP11-438B23.2 | ENSG00000260412 | 0.195840 | -2.352250 | 1.15E-19 | 6.00E-19 |
| RP11-579D7.4 | ENSG00000257660 | 0.196168 | -2.349836 | 2.67E-69 | 1.15E-67 |
| RP5-875O13.1 | ENSG00000233421 | 0.197368 | -2.341040 | 3.34E-28 | 2.83E-27 |
| CTD-2297D10.2 | ENSG00000250579 | 0.197439 | -2.340521 | 1.19E-24 | 8.19E-24 |
| LINC00652 | ENSG00000179935 | 0.197560 | -2.339639 | 6.24E-113 | 7.39E-111 |
| LINC00885 | ENSG00000224652 | 0.197573 | -2.339541 | 1.66E-15 | 6.49E-15 |
| LINC00200 | ENSG00000229205 | 0.197748 | -2.338262 | 5.87E-13 | 1.91E-12 |
| RP11-449J21.5 | ENSG00000267128 | 0.197819 | -2.337744 | 4.63E-46 | 9.06E-45 |
| RP11-697E22.3 | ENSG00000274244 | 0.198227 | -2.334777 | 1.56E-15 | 6.12E-15 |
| BVES-AS1 | ENSG00000203808 | 0.198417 | -2.333395 | 1.84E-53 | 4.77E-52 |
| RP1-7G5.6 | ENSG00000249094 | 0.199385 | -2.326374 | 2.88E-30 | 2.71E-29 |
| LINC01541 | ENSG00000260676 | 0.200552 | -2.317953 | 2.19E-12 | 6.79E-12 |
| RP11-2I17.4 | ENSG00000260173 | 0.200602 | -2.317594 | 2.35E-72 | 1.11E-70 |
| RP11-460I13.2 | ENSG00000227050 | 0.200645 | -2.317282 | 4.38E-47 | 8.91E-46 |
| RP11-96D1.6 | ENSG00000261469 | 0.201984 | -2.307688 | 6.74E-31 | 6.50E-30 |
| RP11-843A23.1 | ENSG00000255084 | 0.202167 | -2.306378 | 3.52E-40 | 5.40E-39 |
| AC098828.2 | ENSG00000234378 | 0.202199 | -2.306150 | 2.62E-23 | 1.67E-22 |
| RP11-358M14.2 | ENSG00000228322 | 0.202382 | -2.304849 | 5.78E-56 | 1.63E-54 |
| RP11-168K11.3 | ENSG00000233817 | 0.202477 | -2.304170 | 7.35E-62 | 2.57E-60 |
| RP4-794I6.4 | ENSG00000277287 | 0.202511 | -2.303925 | 1.41E-60 | 4.71E-59 |
| PCAT18 | ENSG00000265369 | 0.202588 | -2.303380 | 8.35E-52 | 2.01E-50 |
| RP11-44M6.1 | ENSG00000225807 | 0.203865 | -2.294313 | 1.70E-26 | 1.32E-25 |
| RP11-396O20.2 | ENSG00000254645 | 0.205651 | -2.281729 | 2.45E-16 | 1.01E-15 |
| PP14571 | ENSG00000218416 | 0.205961 | -2.279557 | 1.41E-41 | 2.33E-40 |
| AC034110.1 | ENSG00000276397 | 0.206141 | -2.278296 | 2.17E-21 | 1.26E-20 |
| RP5-952N6.1 | ENSG00000229051 | 0.206802 | -2.273676 | 1.04E-22 | 6.44E-22 |
| RP3-333A15.2 | ENSG00000269933 | 0.206869 | -2.273209 | 1.29E-30 | 1.23E-29 |
| GAS1RR | ENSG00000226237 | 0.206875 | -2.273172 | 1.73E-68 | 7.25E-67 |
| RP4-543J13.1 | ENSG00000232895 | 0.207759 | -2.267014 | 7.27E-24 | 4.78E-23 |
| RP11-467P9.1 | ENSG00000272735 | 0.207820 | -2.266593 | 2.55E-56 | 7.37E-55 |
| CTB-171A8.1 | ENSG00000266903 | 0.208122 | -2.264498 | 5.63E-62 | 1.98E-60 |
| KB-1471A8.1 | ENSG00000245330 | 0.208608 | -2.261131 | 1.25E-82 | 8.24E-81 |
| RP11-351M16.3 | ENSG00000237128 | 0.208680 | -2.260632 | 3.30E-23 | 2.09E-22 |
| RP1-10C16.1 | ENSG00000235736 | 0.210057 | -2.251151 | 6.19E-51 | 1.43E-49 |
| SLC25A5-AS1 | ENSG00000224281 | 0.210220 | -2.250029 | 1.68E-140 | 3.15E-138 |
| LINC00454 | ENSG00000226921 | 0.210829 | -2.245855 | 2.87E-18 | 1.35E-17 |
| U47924.27 | ENSG00000257084 | 0.211170 | -2.243524 | 9.38E-09 | 2.17E-08 |
| LINC00052 | ENSG00000259527 | 0.211196 | -2.243342 | 5.28E-12 | 1.60E-11 |
| RP11-109J4.1 | ENSG00000253417 | 0.211472 | -2.241462 | 7.56E-29 | 6.65E-28 |
| RP11-459C13.1 | ENSG00000263312 | 0.211554 | -2.240905 | 7.91E-15 | 2.97E-14 |
| CTA-929C8.6 | ENSG00000226741 | 0.211783 | -2.239345 | 2.30E-10 | 6.15E-10 |
| CTD-3162L10.3 | ENSG00000266973 | 0.212303 | -2.235805 | 1.32E-41 | 2.20E-40 |
| RP11-184M15.2 | ENSG00000248802 | 0.212709 | -2.233045 | 3.74E-26 | 2.85E-25 |
| RP11-106M7.1 | ENSG00000228484 | 0.212916 | -2.231647 | 2.65E-49 | 5.81E-48 |
| RP11-888D10.3 | ENSG00000264964 | 0.213233 | -2.229494 | 5.50E-54 | 1.47E-52 |
| RP5-1056H1.2 | ENSG00000278192 | 0.213511 | -2.227621 | 5.53E-29 | 4.88E-28 |
| IGF2BP2-AS1 | ENSG00000163915 | 0.213567 | -2.227238 | 2.56E-22 | 1.54E-21 |
| RP11-849I19.1 | ENSG00000263146 | 0.213704 | -2.226314 | 4.74E-07 | 9.51E-07 |
| LNX1-AS2 | ENSG00000248494 | 0.214016 | -2.224208 | 8.52E-39 | 1.21E-37 |
| LINC01018 | ENSG00000250056 | 0.214627 | -2.220094 | 1.25E-18 | 6.03E-18 |
| RP11-141O11.2 | ENSG00000249352 | 0.215005 | -2.217557 | 1.09E-46 | 2.19E-45 |
| RP4-594I10.3 | ENSG00000264443 | 0.215498 | -2.214251 | 2.42E-26 | 1.86E-25 |
| RP4-646N3.1 | ENSG00000235806 | 0.216304 | -2.208870 | 7.29E-60 | 2.40E-58 |
| RP11-506E9.3 | ENSG00000261745 | 0.216883 | -2.205010 | 1.88E-16 | 7.85E-16 |
| GLIS3-AS1 | ENSG00000237009 | 0.216884 | -2.205005 | 2.92E-36 | 3.78E-35 |
| AC018730.4 | ENSG00000239587 | 0.217981 | -2.197723 | 3.01E-41 | 4.92E-40 |
| RP1-170O19.24 | ENSG00000270182 | 0.218169 | -2.196481 | 1.80E-35 | 2.23E-34 |
| AF067845.1 | ENSG00000260721 | 0.218834 | -2.192094 | 2.72E-19 | 1.38E-18 |
| CHRM3-AS2 | ENSG00000233355 | 0.219759 | -2.186006 | 9.27E-55 | 2.54E-53 |
| LINC00307 | ENSG00000227342 | 0.219967 | -2.184639 | 2.85E-07 | 5.84E-07 |
| FRY-AS1 | ENSG00000237637 | 0.219985 | -2.184522 | 2.63E-64 | 9.99E-63 |
| SLC14A2-AS1 | ENSG00000267097 | 0.220254 | -2.182757 | 9.11E-28 | 7.52E-27 |
| LA16c-444G7.1 | ENSG00000261273 | 0.220566 | -2.180720 | 1.03E-18 | 4.98E-18 |
| TRPC7-AS1 | ENSG00000248211 | 0.220791 | -2.179250 | 6.75E-25 | 4.71E-24 |
| LINC01182 | ENSG00000250634 | 0.221349 | -2.175603 | 5.49E-34 | 6.24E-33 |
| LINC01447 | ENSG00000236078 | 0.221702 | -2.173303 | 1.33E-25 | 9.75E-25 |
| MGC32805 | ENSG00000250328 | 0.221821 | -2.172531 | 2.09E-14 | 7.59E-14 |
| CHL1-AS1 | ENSG00000234661 | 0.222002 | -2.171357 | 6.49E-29 | 5.71E-28 |
| RP13-452N2.1 | ENSG00000242048 | 0.223062 | -2.164484 | 1.21E-28 | 1.05E-27 |
| RP11-107I14.2 | ENSG00000225208 | 0.223180 | -2.163723 | 4.99E-18 | 2.32E-17 |
| CASC22 | ENSG00000260887 | 0.225386 | -2.149530 | 7.22E-10 | 1.84E-09 |
| RP1-80N2.3 | ENSG00000261211 | 0.226104 | -2.144943 | 2.11E-74 | 1.05E-72 |
| LA16c-325D7.1 | ENSG00000263325 | 0.226523 | -2.142273 | 1.59E-14 | 5.83E-14 |
| TMEM246-AS1 | ENSG00000225376 | 0.226996 | -2.139260 | 3.55E-23 | 2.24E-22 |
| LINC00919 | ENSG00000260268 | 0.227663 | -2.135026 | 7.70E-07 | 1.52E-06 |
| RP13-895J2.2 | ENSG00000256943 | 0.227809 | -2.134103 | 7.59E-19 | 3.72E-18 |
| RP11-496B10.3 | ENSG00000239774 | 0.228453 | -2.130033 | 4.45E-40 | 6.76E-39 |
| RP3-325F22.5 | ENSG00000272189 | 0.228462 | -2.129974 | 3.98E-73 | 1.91E-71 |
| RP11-13N12.1 | ENSG00000253496 | 0.228797 | -2.127862 | 1.01E-24 | 7.01E-24 |
| AC004485.3 | ENSG00000228944 | 0.228855 | -2.127492 | 2.02E-20 | 1.10E-19 |
| RP11-369C8.1 | ENSG00000258616 | 0.229094 | -2.125989 | 1.38E-06 | 2.67E-06 |
| RP11-13P5.1 | ENSG00000224478 | 0.229720 | -2.122052 | 4.17E-26 | 3.17E-25 |
| RP11-441F2.5 | ENSG00000272372 | 0.230006 | -2.120255 | 9.98E-31 | 9.54E-30 |
| IFT74-AS1 | ENSG00000234676 | 0.231537 | -2.110688 | 2.13E-38 | 3.00E-37 |
| RP11-541P9.3 | ENSG00000250061 | 0.231693 | -2.109711 | 1.23E-33 | 1.38E-32 |
| RP11-385N23.1 | ENSG00000230121 | 0.231777 | -2.109190 | 3.25E-19 | 1.64E-18 |
| RP11-298I3.1 | ENSG00000257285 | 0.231832 | -2.108848 | 1.05E-130 | 1.62E-128 |
| RP11-37L2.1 | ENSG00000234535 | 0.231868 | -2.108623 | 4.69E-54 | 1.26E-52 |
| RP11-157L3.12 | ENSG00000275239 | 0.231923 | -2.108279 | 9.50E-14 | 3.29E-13 |
| XXyac-YM21GA2.3 | ENSG00000233884 | 0.232074 | -2.107345 | 3.46E-24 | 2.32E-23 |
| TTLL11-IT1 | ENSG00000237548 | 0.232229 | -2.106380 | 2.40E-45 | 4.57E-44 |
| RP11-124N2.1 | ENSG00000250012 | 0.232595 | -2.104105 | 4.90E-79 | 2.78E-77 |
| RP11-285G1.2 | ENSG00000234504 | 0.232653 | -2.103747 | 5.55E-51 | 1.29E-49 |
| RP5-834N19.1 | ENSG00000232650 | 0.232946 | -2.101935 | 1.06E-33 | 1.18E-32 |
| AC011747.6 | ENSG00000231083 | 0.234253 | -2.093858 | 1.69E-25 | 1.23E-24 |
| RP11-353N14.7 | ENSG00000275516 | 0.234430 | -2.092774 | 3.61E-33 | 3.90E-32 |
| RP11-816J6.3 | ENSG00000269889 | 0.235251 | -2.087724 | 6.88E-59 | 2.17E-57 |
| AC000068.5 | ENSG00000185065 | 0.235615 | -2.085498 | 8.74E-100 | 7.94E-98 |
| CTD-2014B16.3 | ENSG00000258847 | 0.236241 | -2.081670 | 1.09E-36 | 1.43E-35 |
| RP11-103H7.3 | ENSG00000253530 | 0.236968 | -2.077238 | 4.71E-21 | 2.66E-20 |
| RP11-398E10.1 | ENSG00000258998 | 0.237187 | -2.075904 | 1.08E-12 | 3.43E-12 |
| ENO1-AS1 | ENSG00000230679 | 0.237297 | -2.075237 | 1.19E-58 | 3.70E-57 |
| RP11-156L14.1 | ENSG00000265702 | 0.237391 | -2.074665 | 8.75E-28 | 7.24E-27 |
| RP5-933B4.1 | ENSG00000224326 | 0.238172 | -2.069925 | 7.67E-21 | 4.27E-20 |
| RP11-293M10.1 | ENSG00000258740 | 0.239413 | -2.062426 | 4.17E-09 | 9.94E-09 |
| RP11-766F14.1 | ENSG00000248676 | 0.239654 | -2.060978 | 6.32E-15 | 2.38E-14 |
| RP11-342K6.4 | ENSG00000270996 | 0.240534 | -2.055685 | 9.74E-82 | 6.17E-80 |
| LINC00271 | ENSG00000231028 | 0.240715 | -2.054599 | 4.03E-94 | 3.33E-92 |
| RP11-817J15.3 | ENSG00000281655 | 0.240815 | -2.054005 | 9.66E-08 | 2.06E-07 |
| RP11-445O3.1 | ENSG00000231291 | 0.241410 | -2.050441 | 1.83E-18 | 8.75E-18 |
| CTD-3193O13.1 | ENSG00000260500 | 0.241838 | -2.047885 | 7.52E-37 | 9.92E-36 |
| RP11-120K24.5 | ENSG00000269376 | 0.241846 | -2.047839 | 3.54E-18 | 1.66E-17 |
| RP11-31I22.3 | ENSG00000265125 | 0.241979 | -2.047044 | 1.64E-22 | 1.00E-21 |
| RP11-316M20.1 | ENSG00000267790 | 0.242262 | -2.045360 | 2.10E-09 | 5.12E-09 |
| LL22NC03-104C7.1 | ENSG00000236054 | 0.243259 | -2.039435 | 8.12E-30 | 7.47E-29 |
| RP4-781B1.5 | ENSG00000276223 | 0.243843 | -2.035973 | 4.45E-16 | 1.81E-15 |
| AC004221.2 | ENSG00000267778 | 0.244040 | -2.034808 | 6.56E-32 | 6.62E-31 |
| RP11-311H10.4 | ENSG00000223678 | 0.244086 | -2.034537 | 6.56E-34 | 7.42E-33 |
| F10-AS1 | ENSG00000231882 | 0.244411 | -2.032617 | 1.70E-23 | 1.09E-22 |
| AL121578.2 | ENSG00000259977 | 0.244769 | -2.030507 | 3.70E-15 | 1.42E-14 |
| RP11-13K12.2 | ENSG00000267665 | 0.244935 | -2.029529 | 4.59E-21 | 2.60E-20 |
| RP11-10J21.4 | ENSG00000253307 | 0.245236 | -2.027754 | 3.14E-49 | 6.88E-48 |
| KCNH1-IT1 | ENSG00000234233 | 0.245334 | -2.027180 | 1.87E-11 | 5.47E-11 |
| CTB-43E15.1 | ENSG00000253959 | 0.245336 | -2.027172 | 8.36E-11 | 2.31E-10 |
| RP11-123K19.1 | ENSG00000224842 | 0.245376 | -2.026936 | 1.84E-09 | 4.52E-09 |
| RP11-28H5.2 | ENSG00000271983 | 0.245517 | -2.026105 | 8.17E-35 | 9.74E-34 |
| AF131215.8 | ENSG00000270076 | 0.245912 | -2.023787 | 1.23E-39 | 1.81E-38 |
| MGC27382 | ENSG00000237413 | 0.246148 | -2.022404 | 3.91E-56 | 1.12E-54 |
| RP11-141M3.6 | ENSG00000273328 | 0.246492 | -2.020389 | 1.72E-33 | 1.89E-32 |
| AC005220.3 | ENSG00000236352 | 0.246978 | -2.017543 | 2.00E-26 | 1.55E-25 |
| LINC00540 | ENSG00000276476 | 0.247032 | -2.017230 | 9.86E-20 | 5.14E-19 |
| CTD-2330J20.2 | ENSG00000259285 | 0.247372 | -2.015244 | 7.16E-14 | 2.51E-13 |
| RP11-442O1.3 | ENSG00000275088 | 0.247388 | -2.015150 | 3.20E-44 | 5.78E-43 |
| MRGPRF-AS1 | ENSG00000256508 | 0.247686 | -2.013416 | 5.59E-22 | 3.32E-21 |
| RP1-232L24.3 | ENSG00000231533 | 0.247792 | -2.012800 | 4.33E-17 | 1.88E-16 |
| RP11-349H17.2 | ENSG00000275805 | 0.247865 | -2.012372 | 1.65E-34 | 1.93E-33 |
| XXbac-B33L19.12 | ENSG00000278044 | 0.248345 | -2.009583 | 3.60E-12 | 1.10E-11 |
| RP1-90J20.8 | ENSG00000224846 | 0.248525 | -2.008538 | 7.42E-16 | 2.98E-15 |
| RP11-10K17.6 | ENSG00000267070 | 0.249097 | -2.005218 | 3.65E-36 | 4.70E-35 |
| ST3GAL6-AS1 | ENSG00000239445 | 0.249499 | -2.002896 | 3.11E-39 | 4.50E-38 |
