# Supplementary material for: A Computationally Constructed lncRNA-Associated Competing Triplet Network in Clear Cell Renal Cell Carcinoma
Source: Dis Markers. 2022 Nov 17;2022:8928282. doi: 10.1155/2022/8928282 (PMC9691318; doi:10.1155/2022/8928282)
Supplement: Supplementary Materials — Table S1: the list of upregulated lncRNAs in ccRCC. Table S2: the list of downregulated lncRNAs in ccRCC. Table S3: the list of upregulated mRNAs in ccRCC. Table S4: the list of downregulated mRNAs in ccRCC. Table S5: the list of upregulated miRNAs in ccRCC. Table S6: the list of downregulated miRNAs in ccRCC. Table S7: the list of top 100 dysregulated (50 upregulated and 50 downregulated) lncRNAs in consistent with Figure 1. Table S8: the list of genes coexpressed with HOTTIP in ccRCC. [file 8928282.f1.zip › 8928282.f1/Table S3 (1).docx]

Table S3. The list of up-regulated mRNAs in ccRCC.

| **Gene symbol** | **Ensemb ID** | **Fold Change (FC)  (T/N)** | **log_2_FC (T/N)** | ***P* value** | **FDR** |
| --- | --- | --- | --- | --- | --- |
| GSG1L2 | ENSG00000214978 | 1297.477323 | 10.341494 | 2.37E-27 | 1.36E-26 |
| MUC17 | ENSG00000169876 | 567.907336 | 9.149512 | 5.38E-25 | 2.76E-24 |
| SLC18A3 | ENSG00000187714 | 479.263816 | 8.904676 | 3.75E-21 | 1.58E-20 |
| CFHR5 | ENSG00000134389 | 405.731665 | 8.664382 | 1.90E-12 | 4.98E-12 |
| PAEP | ENSG00000122133 | 298.558854 | 8.221872 | 1.20E-22 | 5.45E-22 |
| S100G | ENSG00000169906 | 265.722999 | 8.053779 | 6.75E-17 | 2.29E-16 |
| HP | ENSG00000257017 | 239.587377 | 7.904408 | 5.17E-24 | 2.51E-23 |
| RTL1 | ENSG00000254656 | 226.438089 | 7.822973 | 1.37E-13 | 3.86E-13 |
| TBX5 | ENSG00000089225 | 163.781797 | 7.355631 | 8.93E-27 | 4.99E-26 |
| BIRC7 | ENSG00000101197 | 155.276181 | 7.278693 | 4.94E-47 | 7.31E-46 |
| CRP | ENSG00000132693 | 153.324927 | 7.260448 | 5.22E-23 | 2.40E-22 |
| MYEOV | ENSG00000172927 | 145.056695 | 7.180473 | 2.31E-56 | 4.96E-55 |
| HHATL | ENSG00000010282 | 135.270484 | 7.079703 | 6.67E-17 | 2.26E-16 |
| FDCSP | ENSG00000181617 | 118.139523 | 6.884348 | 7.59E-13 | 2.04E-12 |
| APOA4 | ENSG00000110244 | 111.906143 | 6.806145 | 1.76E-10 | 4.07E-10 |
| PRSS38 | ENSG00000185888 | 110.936613 | 6.793592 | 1.91E-12 | 5.01E-12 |
| F13B | ENSG00000143278 | 107.994413 | 6.754813 | 4.83E-16 | 1.56E-15 |
| PAGE2 | ENSG00000234068 | 107.413103 | 6.747026 | 3.48E-14 | 1.01E-13 |
| CHAT | ENSG00000070748 | 105.132780 | 6.716069 | 3.36E-15 | 1.03E-14 |
| ITIH1 | ENSG00000055957 | 103.551192 | 6.694200 | 1.68E-23 | 7.97E-23 |
| OR2A4 | ENSG00000180658 | 102.967081 | 6.686039 | 3.39E-63 | 9.61E-62 |
| GOLGA6L2 | ENSG00000174450 | 89.913778 | 6.490470 | 1.04E-21 | 4.51E-21 |
| SSX1 | ENSG00000126752 | 88.233536 | 6.463255 | 1.69E-10 | 3.93E-10 |
| DCAF4L2 | ENSG00000176566 | 85.591293 | 6.419392 | 6.90E-10 | 1.54E-09 |
| PLPPR5 | ENSG00000117598 | 84.269481 | 6.396938 | 1.19E-44 | 1.54E-43 |
| KLK4 | ENSG00000167749 | 80.432869 | 6.329713 | 5.04E-14 | 1.45E-13 |
| CYP2A6 | ENSG00000255974 | 79.608518 | 6.314851 | 1.04E-20 | 4.29E-20 |
| DOC2A | ENSG00000149927 | 71.776184 | 6.165433 | 9.33E-77 | 4.29E-75 |
| ORM2 | ENSG00000228278 | 70.968695 | 6.149111 | 2.39E-16 | 7.82E-16 |
| MAGEB1 | ENSG00000214107 | 67.905446 | 6.085455 | 3.27E-10 | 7.45E-10 |
| SLC6A3 | ENSG00000142319 | 67.135540 | 6.069005 | 3.66E-46 | 5.19E-45 |
| RPTN | ENSG00000215853 | 66.331432 | 6.051621 | 9.64E-15 | 2.88E-14 |
| C10orf99 | ENSG00000188373 | 65.905027 | 6.042317 | 1.93E-24 | 9.60E-24 |
| RP11-231I13.2 | ENSG00000242120 | 65.479259 | 6.032966 | 7.48E-10 | 1.66E-09 |
| PADI1 | ENSG00000142623 | 63.237778 | 5.982715 | 2.26E-33 | 1.75E-32 |
| PAGE2B | ENSG00000238269 | 60.698272 | 5.923584 | 1.92E-16 | 6.31E-16 |
| CA9 | ENSG00000107159 | 60.075922 | 5.908715 | 2.20E-74 | 9.30E-73 |
| TNFAIP6 | ENSG00000123610 | 59.060130 | 5.884113 | 5.14E-76 | 2.30E-74 |
| SAA2-SAA4 | ENSG00000255071 | 58.532014 | 5.871154 | 6.33E-14 | 1.81E-13 |
| NTSR2 | ENSG00000169006 | 57.965384 | 5.857120 | 1.36E-11 | 3.38E-11 |
| ORM1 | ENSG00000229314 | 57.452463 | 5.844297 | 1.55E-13 | 4.34E-13 |
| GUCA2B | ENSG00000044012 | 57.450281 | 5.844242 | 7.24E-31 | 4.96E-30 |
| BAAT | ENSG00000136881 | 55.736964 | 5.800563 | 5.15E-22 | 2.26E-21 |
| HSF4 | ENSG00000102878 | 55.527755 | 5.795137 | 4.58E-88 | 3.07E-86 |
| FABP7 | ENSG00000164434 | 55.392862 | 5.791628 | 1.53E-28 | 9.35E-28 |
| MCHR1 | ENSG00000128285 | 55.012381 | 5.781684 | 4.17E-41 | 4.64E-40 |
| CCL18 | ENSG00000275385 | 54.960508 | 5.780323 | 3.84E-40 | 4.07E-39 |
| LHX8 | ENSG00000162624 | 53.418953 | 5.739280 | 2.15E-21 | 9.17E-21 |
| FABP6 | ENSG00000170231 | 52.921281 | 5.725776 | 1.05E-59 | 2.53E-58 |
| IGFN1 | ENSG00000163395 | 52.780172 | 5.721924 | 4.26E-19 | 1.61E-18 |
| TEX15 | ENSG00000133863 | 51.522609 | 5.687134 | 1.92E-33 | 1.49E-32 |
| F9 | ENSG00000101981 | 50.561341 | 5.659963 | 1.04E-09 | 2.29E-09 |
| OPN4 | ENSG00000122375 | 49.401558 | 5.626485 | 3.07E-39 | 3.10E-38 |
| APOA5 | ENSG00000110243 | 48.996321 | 5.614602 | 5.35E-10 | 1.20E-09 |
| PTHLH | ENSG00000087494 | 46.920233 | 5.552138 | 4.62E-38 | 4.46E-37 |
| SAA1 | ENSG00000173432 | 46.192689 | 5.529593 | 4.47E-19 | 1.69E-18 |
| SOX11 | ENSG00000176887 | 45.464787 | 5.506678 | 3.09E-63 | 8.76E-62 |
| MOG | ENSG00000204655 | 45.397996 | 5.504557 | 6.50E-14 | 1.86E-13 |
| KCNU1 | ENSG00000215262 | 45.084938 | 5.494574 | 1.43E-15 | 4.46E-15 |
| CHRNA1 | ENSG00000138435 | 44.455956 | 5.474305 | 6.69E-40 | 6.99E-39 |
| NDUFA4L2 | ENSG00000185633 | 44.429999 | 5.473462 | 1.21E-85 | 7.41E-84 |
| C8A | ENSG00000157131 | 44.111794 | 5.463093 | 2.21E-09 | 4.77E-09 |
| PIP | ENSG00000159763 | 43.166053 | 5.431825 | 1.09E-12 | 2.89E-12 |
| MMP13 | ENSG00000137745 | 43.118076 | 5.430221 | 4.37E-14 | 1.26E-13 |
| FAM57B | ENSG00000149926 | 41.867608 | 5.387763 | 3.04E-29 | 1.92E-28 |
| MBL2 | ENSG00000165471 | 41.768760 | 5.384352 | 5.20E-13 | 1.41E-12 |
| IBSP | ENSG00000029559 | 40.828713 | 5.351512 | 6.55E-30 | 4.30E-29 |
| SCGB3A2 | ENSG00000164265 | 39.306446 | 5.296694 | 9.11E-31 | 6.21E-30 |
| TMPRSS11A | ENSG00000187054 | 39.046570 | 5.287124 | 3.34E-17 | 1.15E-16 |
| FAM153C | ENSG00000204677 | 37.421794 | 5.225807 | 6.78E-46 | 9.41E-45 |
| SAA2 | ENSG00000134339 | 36.912864 | 5.206052 | 1.94E-18 | 7.08E-18 |
| EPYC | ENSG00000083782 | 36.875136 | 5.204576 | 2.97E-20 | 1.20E-19 |
| UGT2B15 | ENSG00000196620 | 36.576075 | 5.192828 | 4.98E-13 | 1.35E-12 |
| OMG | ENSG00000126861 | 36.413352 | 5.186396 | 1.90E-26 | 1.04E-25 |
| NPTX2 | ENSG00000106236 | 36.364743 | 5.184468 | 8.33E-36 | 7.19E-35 |
| REG1B | ENSG00000172023 | 36.316852 | 5.182567 | 3.04E-15 | 9.38E-15 |
| KISS1R | ENSG00000116014 | 36.285002 | 5.181301 | 4.11E-37 | 3.77E-36 |
| C8B | ENSG00000021852 | 36.262200 | 5.180395 | 1.74E-08 | 3.53E-08 |
| PNCK | ENSG00000130822 | 35.788875 | 5.161439 | 9.52E-38 | 9.00E-37 |
| GABRD | ENSG00000187730 | 35.725899 | 5.158898 | 4.98E-94 | 3.79E-92 |
| MYH13 | ENSG00000006788 | 35.614791 | 5.154405 | 4.59E-20 | 1.84E-19 |
| APOC1 | ENSG00000130208 | 35.334384 | 5.143001 | 2.10E-73 | 8.63E-72 |
| ADAMTS20 | ENSG00000173157 | 35.295488 | 5.141412 | 1.26E-29 | 8.19E-29 |
| LHX9 | ENSG00000143355 | 35.198121 | 5.137427 | 8.62E-13 | 2.31E-12 |
| SULT2A1 | ENSG00000105398 | 35.142003 | 5.135125 | 8.91E-10 | 1.97E-09 |
| CYP2J2 | ENSG00000134716 | 34.862280 | 5.123595 | 1.69E-61 | 4.40E-60 |
| CHIT1 | ENSG00000133063 | 34.556125 | 5.110870 | 7.51E-30 | 4.91E-29 |
| ST8SIA3 | ENSG00000177511 | 34.333504 | 5.101545 | 9.68E-12 | 2.44E-11 |
| TMEM155 | ENSG00000164112 | 34.140161 | 5.093398 | 3.12E-67 | 1.02E-65 |
| HTR6 | ENSG00000158748 | 33.446475 | 5.063782 | 5.40E-30 | 3.55E-29 |
| PAGE1 | ENSG00000068985 | 33.248840 | 5.055232 | 8.08E-08 | 1.57E-07 |
| KRT25 | ENSG00000204897 | 33.212186 | 5.053641 | 1.26E-18 | 4.65E-18 |
| CDKN2A | ENSG00000147889 | 33.127628 | 5.049963 | 7.78E-93 | 5.84E-91 |
| GRIK3 | ENSG00000163873 | 32.885043 | 5.039360 | 1.57E-72 | 6.21E-71 |
| RUFY4 | ENSG00000188282 | 32.787667 | 5.035081 | 4.85E-49 | 7.79E-48 |
| NKX2-5 | ENSG00000183072 | 32.768190 | 5.034224 | 1.58E-16 | 5.22E-16 |
| NMRK2 | ENSG00000077009 | 32.424236 | 5.019001 | 2.83E-10 | 6.48E-10 |
| CASP14 | ENSG00000105141 | 32.036247 | 5.001633 | 4.34E-07 | 8.04E-07 |
| ANGPTL4 | ENSG00000167772 | 31.271298 | 4.966767 | 5.21E-86 | 3.25E-84 |
| RAB42 | ENSG00000188060 | 31.138412 | 4.960623 | 2.34E-63 | 6.71E-62 |
| AICDA | ENSG00000111732 | 31.000238 | 4.954207 | 2.78E-42 | 3.28E-41 |
| LYPD4 | ENSG00000273111 | 30.999901 | 4.954192 | 6.48E-16 | 2.07E-15 |
| HS3ST2 | ENSG00000122254 | 30.592264 | 4.935095 | 7.75E-50 | 1.30E-48 |
| C5orf46 | ENSG00000178776 | 30.343962 | 4.923338 | 3.27E-23 | 1.52E-22 |
| RTP2 | ENSG00000198471 | 30.093615 | 4.911386 | 3.10E-37 | 2.86E-36 |
| COL23A1 | ENSG00000050767 | 29.664753 | 4.890678 | 1.29E-78 | 6.39E-77 |
| OR10Q1 | ENSG00000180475 | 29.636280 | 4.889292 | 1.53E-30 | 1.03E-29 |
| HTRA4 | ENSG00000169495 | 29.279721 | 4.871830 | 1.52E-61 | 3.95E-60 |
| CXCL5 | ENSG00000163735 | 28.817071 | 4.848852 | 2.31E-23 | 1.08E-22 |
| ITGAD | ENSG00000156886 | 28.110321 | 4.813028 | 7.29E-62 | 1.94E-60 |
| ABCB5 | ENSG00000004846 | 27.703924 | 4.792018 | 2.63E-16 | 8.61E-16 |
| MAGEC2 | ENSG00000046774 | 27.652384 | 4.789332 | 1.08E-05 | 1.82E-05 |
| LBP | ENSG00000129988 | 27.567953 | 4.784920 | 2.36E-16 | 7.72E-16 |
| MAGEB2 | ENSG00000099399 | 27.371448 | 4.774600 | 1.07E-06 | 1.93E-06 |
| TERT | ENSG00000164362 | 27.177211 | 4.764326 | 2.53E-17 | 8.78E-17 |
| SAA4 | ENSG00000148965 | 26.966513 | 4.753097 | 1.18E-14 | 3.53E-14 |
| KERA | ENSG00000139330 | 26.232543 | 4.713286 | 8.40E-15 | 2.53E-14 |
| CD5L | ENSG00000073754 | 26.183564 | 4.710590 | 2.69E-25 | 1.40E-24 |
| HPX | ENSG00000110169 | 26.154575 | 4.708991 | 1.80E-27 | 1.04E-26 |
| NKX6-1 | ENSG00000163623 | 26.008556 | 4.700914 | 8.16E-11 | 1.93E-10 |
| LGI4 | ENSG00000153902 | 25.811306 | 4.689931 | 1.42E-43 | 1.76E-42 |
| PAGE5 | ENSG00000158639 | 25.488831 | 4.671793 | 8.16E-11 | 1.93E-10 |
| TFAP2D | ENSG00000008197 | 25.270087 | 4.659359 | 9.93E-14 | 2.81E-13 |
| SCGN | ENSG00000079689 | 25.095483 | 4.649356 | 4.21E-34 | 3.39E-33 |
| HILPDA | ENSG00000135245 | 24.985985 | 4.643047 | 1.03E-104 | 1.06E-102 |
| AADAC | ENSG00000114771 | 24.541679 | 4.617162 | 1.49E-12 | 3.94E-12 |
| SST | ENSG00000157005 | 24.242873 | 4.599489 | 3.64E-15 | 1.12E-14 |
| FEZF2 | ENSG00000153266 | 24.153754 | 4.594176 | 5.26E-08 | 1.04E-07 |
| PMCH | ENSG00000183395 | 24.057577 | 4.588419 | 6.72E-35 | 5.59E-34 |
| SDS | ENSG00000135094 | 23.860932 | 4.576578 | 5.61E-62 | 1.50E-60 |
| TAT | ENSG00000198650 | 23.721045 | 4.568096 | 2.38E-16 | 7.80E-16 |
| ACTL6B | ENSG00000077080 | 23.713247 | 4.567621 | 7.56E-15 | 2.28E-14 |
| KCNK9 | ENSG00000169427 | 23.556672 | 4.558064 | 9.45E-46 | 1.31E-44 |
| DNAH11 | ENSG00000105877 | 23.472300 | 4.552887 | 5.26E-54 | 1.03E-52 |
| ENPP3 | ENSG00000154269 | 23.425009 | 4.549978 | 6.07E-66 | 1.93E-64 |
| DMRT3 | ENSG00000064218 | 23.192330 | 4.535576 | 1.61E-15 | 5.02E-15 |
| FIBCD1 | ENSG00000130720 | 23.109649 | 4.530423 | 1.26E-26 | 6.96E-26 |
| NR2E1 | ENSG00000112333 | 23.087672 | 4.529051 | 2.04E-20 | 8.33E-20 |
| PLA2G4D | ENSG00000159337 | 23.057585 | 4.527169 | 5.39E-26 | 2.90E-25 |
| UGT2B10 | ENSG00000109181 | 22.782603 | 4.509861 | 3.91E-10 | 8.87E-10 |
| MUC12 | ENSG00000205277 | 22.276059 | 4.477422 | 3.28E-41 | 3.67E-40 |
| IFNG | ENSG00000111537 | 22.263134 | 4.476585 | 7.31E-40 | 7.61E-39 |
| UGT1A4 | ENSG00000244474 | 22.146173 | 4.468985 | 1.22E-15 | 3.85E-15 |
| SLC1A6 | ENSG00000105143 | 22.103708 | 4.466217 | 1.46E-13 | 4.10E-13 |
| SLC35G3 | ENSG00000164729 | 22.079166 | 4.464614 | 1.49E-16 | 4.95E-16 |
| TF | ENSG00000091513 | 21.947894 | 4.456011 | 6.67E-17 | 2.26E-16 |
| KRT20 | ENSG00000171431 | 21.906093 | 4.453260 | 1.69E-10 | 3.91E-10 |
| POU4F1 | ENSG00000152192 | 21.884809 | 4.451858 | 2.51E-16 | 8.21E-16 |
| IGFL2 | ENSG00000204866 | 21.723121 | 4.441160 | 2.09E-25 | 1.10E-24 |
| SOHLH1 | ENSG00000165643 | 21.452408 | 4.423068 | 9.14E-12 | 2.30E-11 |
| TMEM145 | ENSG00000167619 | 21.340413 | 4.415516 | 1.17E-33 | 9.17E-33 |
| SOX1 | ENSG00000182968 | 21.236924 | 4.408503 | 2.21E-12 | 5.77E-12 |
| CYP2A7 | ENSG00000198077 | 21.234829 | 4.408361 | 2.33E-13 | 6.46E-13 |
| SCG3 | ENSG00000104112 | 21.206299 | 4.406421 | 7.99E-17 | 2.69E-16 |
| LCN1 | ENSG00000160349 | 21.083071 | 4.398013 | 5.54E-27 | 3.13E-26 |
| ADAMDEC1 | ENSG00000134028 | 20.794996 | 4.378165 | 6.19E-36 | 5.36E-35 |
| LHX2 | ENSG00000106689 | 20.781909 | 4.377256 | 4.24E-22 | 1.87E-21 |
| TMPRSS11E | ENSG00000087128 | 20.526209 | 4.359395 | 2.67E-08 | 5.36E-08 |
| CD70 | ENSG00000125726 | 20.317928 | 4.344681 | 5.75E-34 | 4.58E-33 |
| CXCL13 | ENSG00000156234 | 20.228797 | 4.338339 | 1.33E-27 | 7.75E-27 |
| IGFBP1 | ENSG00000146678 | 20.214942 | 4.337350 | 8.97E-17 | 3.01E-16 |
| MMP9 | ENSG00000100985 | 20.213028 | 4.337214 | 9.61E-32 | 6.90E-31 |
| GOLGA6L7P | ENSG00000261649 | 20.128611 | 4.331176 | 5.87E-17 | 2.00E-16 |
| TMEM225 | ENSG00000204300 | 20.002656 | 4.322120 | 2.99E-14 | 8.70E-14 |
| KCNN1 | ENSG00000105642 | 19.976086 | 4.320202 | 3.34E-43 | 4.08E-42 |
| PASD1 | ENSG00000166049 | 19.880322 | 4.313269 | 3.57E-05 | 5.80E-05 |
| LYZL1 | ENSG00000120563 | 19.841689 | 4.310463 | 1.17E-22 | 5.30E-22 |
| ALOX15B | ENSG00000179593 | 19.806150 | 4.307877 | 1.08E-45 | 1.48E-44 |
| SEC14L3 | ENSG00000100012 | 19.670188 | 4.297939 | 3.26E-23 | 1.52E-22 |
| SIGLEC8 | ENSG00000105366 | 19.587597 | 4.291868 | 1.72E-61 | 4.47E-60 |
| NXPH4 | ENSG00000182379 | 19.533762 | 4.287898 | 1.43E-58 | 3.30E-57 |
| ANGPTL8 | ENSG00000130173 | 19.497032 | 4.285183 | 8.91E-18 | 3.16E-17 |
| BARX1 | ENSG00000131668 | 19.381747 | 4.276627 | 1.22E-14 | 3.62E-14 |
| GATA4 | ENSG00000136574 | 19.365134 | 4.275390 | 5.40E-11 | 1.29E-10 |
| SNCB | ENSG00000074317 | 19.254154 | 4.267098 | 9.78E-11 | 2.30E-10 |
| DMP1 | ENSG00000152592 | 19.150035 | 4.259275 | 4.88E-27 | 2.76E-26 |
| CDCA2 | ENSG00000184661 | 18.984613 | 4.246759 | 5.79E-74 | 2.42E-72 |
| LECT1 | ENSG00000136110 | 18.955526 | 4.244547 | 1.32E-11 | 3.28E-11 |
| AJ239318.1 | ENSG00000280108 | 18.953003 | 4.244355 | 1.39E-09 | 3.04E-09 |
| EGLN3 | ENSG00000129521 | 18.940212 | 4.243381 | 1.51E-115 | 1.92E-113 |
| AL008723.1 | ENSG00000279219 | 18.747794 | 4.228649 | 3.96E-34 | 3.19E-33 |
| TFR2 | ENSG00000106327 | 18.700749 | 4.225024 | 8.81E-33 | 6.64E-32 |
| PLK5 | ENSG00000185988 | 18.519545 | 4.210977 | 3.67E-11 | 8.91E-11 |
| CDHR1 | ENSG00000148600 | 18.294927 | 4.193372 | 4.38E-32 | 3.20E-31 |
| PGLYRP2 | ENSG00000161031 | 18.242446 | 4.189227 | 2.37E-20 | 9.63E-20 |
| DEFB118 | ENSG00000131068 | 18.232439 | 4.188436 | 1.90E-13 | 5.29E-13 |
| CNPY1 | ENSG00000146910 | 18.164575 | 4.183056 | 1.85E-21 | 7.93E-21 |
| LAIR2 | ENSG00000167618 | 18.037026 | 4.172890 | 5.19E-30 | 3.42E-29 |
| GAGE1 | ENSG00000205777 | 17.943271 | 4.165371 | 2.44E-06 | 4.29E-06 |
| ARX | ENSG00000004848 | 17.773065 | 4.151621 | 1.11E-13 | 3.13E-13 |
| SERPINA12 | ENSG00000165953 | 17.546312 | 4.133096 | 1.16E-15 | 3.67E-15 |
| AHNAK2 | ENSG00000185567 | 17.461108 | 4.126073 | 2.19E-68 | 7.70E-67 |
| AKR1D1 | ENSG00000122787 | 17.065180 | 4.092984 | 7.24E-15 | 2.18E-14 |
| STMN2 | ENSG00000104435 | 17.063731 | 4.092861 | 2.20E-13 | 6.11E-13 |
| TREM2 | ENSG00000095970 | 17.022382 | 4.089361 | 1.14E-87 | 7.46E-86 |
| ITPKA | ENSG00000137825 | 17.007940 | 4.088137 | 4.59E-32 | 3.34E-31 |
| TNFRSF9 | ENSG00000049249 | 16.933811 | 4.081835 | 1.36E-47 | 2.06E-46 |
| SCG2 | ENSG00000171951 | 16.916253 | 4.080338 | 5.55E-35 | 4.63E-34 |
| CFHR2 | ENSG00000080910 | 16.886904 | 4.077833 | 4.66E-07 | 8.63E-07 |
| ADAM18 | ENSG00000168619 | 16.833390 | 4.073254 | 9.41E-17 | 3.16E-16 |
| CDH4 | ENSG00000179242 | 16.693493 | 4.061214 | 5.62E-36 | 4.87E-35 |
| TNNT3 | ENSG00000130595 | 16.658964 | 4.058227 | 2.86E-18 | 1.04E-17 |
| CFAP74 | ENSG00000142609 | 16.529092 | 4.046936 | 1.57E-84 | 9.17E-83 |
| BECN2 | ENSG00000196289 | 16.416236 | 4.037051 | 1.54E-16 | 5.09E-16 |
| IL20RB | ENSG00000174564 | 16.211298 | 4.018928 | 2.22E-21 | 9.49E-21 |
| PGF | ENSG00000119630 | 16.210300 | 4.018839 | 7.78E-42 | 8.96E-41 |
| VSIG1 | ENSG00000101842 | 16.200247 | 4.017944 | 3.59E-59 | 8.48E-58 |
| SSX3 | ENSG00000165584 | 16.130094 | 4.011683 | 6.07E-11 | 1.45E-10 |
| SLC35G4 | ENSG00000236396 | 15.992247 | 3.999301 | 5.32E-11 | 1.27E-10 |
| LRIT2 | ENSG00000204033 | 15.841931 | 3.985676 | 1.34E-20 | 5.50E-20 |
| NNMT | ENSG00000166741 | 15.837482 | 3.985271 | 2.75E-67 | 9.03E-66 |
| SPINK13 | ENSG00000214510 | 15.693859 | 3.972128 | 7.03E-27 | 3.95E-26 |
| SLC38A8 | ENSG00000166558 | 15.646843 | 3.967800 | 9.77E-09 | 2.02E-08 |
| CACNG2 | ENSG00000166862 | 15.583380 | 3.961936 | 4.50E-13 | 1.22E-12 |
| STC2 | ENSG00000113739 | 15.577163 | 3.961361 | 4.26E-81 | 2.29E-79 |
| B4GALNT1 | ENSG00000135454 | 15.566416 | 3.960365 | 1.62E-27 | 9.34E-27 |
| CATSPERD | ENSG00000174898 | 15.556269 | 3.959424 | 3.74E-16 | 1.21E-15 |
| PRAC2 | ENSG00000229637 | 15.497572 | 3.953970 | 4.29E-13 | 1.17E-12 |
| NLRP13 | ENSG00000173572 | 15.396414 | 3.944523 | 2.70E-13 | 7.44E-13 |
| SPAG4 | ENSG00000061656 | 15.232985 | 3.929127 | 1.31E-114 | 1.61E-112 |
| TNFSF14 | ENSG00000125735 | 15.195961 | 3.925616 | 3.20E-44 | 4.06E-43 |
| GOLGA7B | ENSG00000155265 | 15.190081 | 3.925058 | 1.39E-53 | 2.69E-52 |
| FGF21 | ENSG00000105550 | 15.174229 | 3.923551 | 2.41E-12 | 6.27E-12 |
| HES5 | ENSG00000197921 | 15.108468 | 3.917285 | 1.86E-37 | 1.73E-36 |
| HAMP | ENSG00000105697 | 15.106942 | 3.917140 | 1.92E-38 | 1.88E-37 |
| PSG8 | ENSG00000124467 | 14.958514 | 3.902895 | 1.28E-08 | 2.62E-08 |
| LGALS12 | ENSG00000133317 | 14.938632 | 3.900976 | 1.18E-27 | 6.86E-27 |
| CRX | ENSG00000105392 | 14.913482 | 3.898545 | 4.74E-17 | 1.62E-16 |
| ZP1 | ENSG00000149506 | 14.877588 | 3.895069 | 7.96E-21 | 3.31E-20 |
| SERPIND1 | ENSG00000099937 | 14.761587 | 3.883776 | 3.71E-13 | 1.01E-12 |
| DCAF8L2 | ENSG00000189186 | 14.651602 | 3.872986 | 2.31E-08 | 4.67E-08 |
| APOL5 | ENSG00000128313 | 14.492049 | 3.857190 | 1.41E-48 | 2.21E-47 |
| INHBB | ENSG00000163083 | 14.484081 | 3.856396 | 1.12E-69 | 4.09E-68 |
| AMZ1 | ENSG00000174945 | 14.419860 | 3.849985 | 3.22E-41 | 3.60E-40 |
| F2 | ENSG00000180210 | 14.402895 | 3.848287 | 2.66E-13 | 7.32E-13 |
| GALNTL5 | ENSG00000106648 | 14.388758 | 3.846870 | 7.56E-15 | 2.28E-14 |
| TNIP3 | ENSG00000050730 | 14.378186 | 3.845810 | 7.12E-38 | 6.78E-37 |
| TSPY2 | ENSG00000168757 | 14.362467 | 3.844232 | 3.36E-06 | 5.85E-06 |
| C19orf67 | ENSG00000188032 | 14.249923 | 3.832882 | 1.46E-26 | 8.04E-26 |
| PSG2 | ENSG00000242221 | 14.205405 | 3.828368 | 5.26E-13 | 1.42E-12 |
| CTAGE9 | ENSG00000236761 | 14.180662 | 3.825853 | 6.09E-43 | 7.36E-42 |
| CT45A10 | ENSG00000269586 | 14.175730 | 3.825351 | 7.18E-07 | 1.31E-06 |
| SCARB1 | ENSG00000073060 | 14.120336 | 3.819702 | 3.37E-94 | 2.58E-92 |
| C1QL1 | ENSG00000131094 | 14.107415 | 3.818382 | 3.61E-25 | 1.87E-24 |
| FAM9A | ENSG00000183304 | 14.087066 | 3.816299 | 1.20E-16 | 4.00E-16 |
| PSG6 | ENSG00000170848 | 14.052598 | 3.812765 | 3.32E-13 | 9.10E-13 |
| TMEM74B | ENSG00000125895 | 14.022982 | 3.809721 | 1.37E-86 | 8.67E-85 |
| HLA-G | ENSG00000204632 | 14.004971 | 3.807867 | 2.20E-45 | 2.98E-44 |
| LHX3 | ENSG00000107187 | 13.885658 | 3.795524 | 9.46E-09 | 1.95E-08 |
| DMRTC2 | ENSG00000142025 | 13.851998 | 3.792022 | 9.69E-11 | 2.28E-10 |
| CT45A1 | ENSG00000268940 | 13.834762 | 3.790226 | 9.42E-09 | 1.95E-08 |
| CPB2 | ENSG00000080618 | 13.822898 | 3.788988 | 1.68E-12 | 4.43E-12 |
| TMEM91 | ENSG00000142046 | 13.820130 | 3.788699 | 5.26E-77 | 2.46E-75 |
| MYOD1 | ENSG00000129152 | 13.776961 | 3.784186 | 3.43E-16 | 1.11E-15 |
| DNAJB13 | ENSG00000187726 | 13.726502 | 3.778892 | 1.31E-36 | 1.17E-35 |
| PSORS1C2 | ENSG00000204538 | 13.714953 | 3.777678 | 8.99E-45 | 1.17E-43 |
| TTC24 | ENSG00000187862 | 13.650119 | 3.770842 | 1.61E-35 | 1.37E-34 |
| ARRDC5 | ENSG00000205784 | 13.563769 | 3.761686 | 9.05E-49 | 1.43E-47 |
| DNAJC5B | ENSG00000147570 | 13.528110 | 3.757888 | 8.00E-52 | 1.47E-50 |
| OR2AT4 | ENSG00000171561 | 13.378946 | 3.741893 | 7.70E-14 | 2.19E-13 |
| OLIG2 | ENSG00000205927 | 13.374456 | 3.741408 | 1.24E-11 | 3.10E-11 |
| SEMA5B | ENSG00000082684 | 13.117606 | 3.713433 | 5.00E-81 | 2.68E-79 |
| ZAN | ENSG00000146839 | 13.042668 | 3.705167 | 7.40E-14 | 2.11E-13 |
| PLA2G7 | ENSG00000146070 | 13.005504 | 3.701050 | 3.64E-54 | 7.19E-53 |
| ANO4 | ENSG00000151572 | 12.873551 | 3.686338 | 2.95E-39 | 2.99E-38 |
| WFDC10B | ENSG00000182931 | 12.846124 | 3.683261 | 1.61E-19 | 6.25E-19 |
| AVPR1B | ENSG00000198049 | 12.810823 | 3.679291 | 3.79E-42 | 4.44E-41 |
| CES4A | ENSG00000172824 | 12.792939 | 3.677276 | 7.65E-45 | 1.01E-43 |
| PAQR9 | ENSG00000188582 | 12.772537 | 3.674973 | 1.81E-29 | 1.16E-28 |
| PGGHG | ENSG00000142102 | 12.707746 | 3.667636 | 1.81E-41 | 2.05E-40 |
| KRT81 | ENSG00000205426 | 12.648649 | 3.660911 | 1.37E-29 | 8.88E-29 |
| CLEC12B | ENSG00000256660 | 12.600311 | 3.655387 | 1.70E-38 | 1.67E-37 |
| COL11A1 | ENSG00000060718 | 12.583815 | 3.653497 | 3.36E-18 | 1.22E-17 |
| UGT1A3 | ENSG00000243135 | 12.559161 | 3.650668 | 7.34E-24 | 3.54E-23 |
| PRSS53 | ENSG00000151006 | 12.498619 | 3.643697 | 2.04E-49 | 3.35E-48 |
| COL5A3 | ENSG00000080573 | 12.490014 | 3.642703 | 3.11E-94 | 2.40E-92 |
| ODF3B | ENSG00000177989 | 12.483114 | 3.641906 | 2.14E-83 | 1.22E-81 |
| RBM46 | ENSG00000151962 | 12.476475 | 3.641139 | 7.13E-33 | 5.40E-32 |
| SLCO1B1 | ENSG00000134538 | 12.421843 | 3.634807 | 9.04E-07 | 1.64E-06 |
| CP | ENSG00000047457 | 12.413474 | 3.633835 | 6.93E-29 | 4.30E-28 |
| KRT72 | ENSG00000170486 | 12.412252 | 3.633693 | 5.76E-25 | 2.95E-24 |
| CD8A | ENSG00000153563 | 12.378760 | 3.629795 | 2.83E-47 | 4.22E-46 |
| SLC17A4 | ENSG00000146039 | 12.310463 | 3.621813 | 8.60E-29 | 5.31E-28 |
| JAKMIP1 | ENSG00000152969 | 12.297374 | 3.620278 | 1.06E-50 | 1.86E-49 |
| GALP | ENSG00000197487 | 12.281587 | 3.618425 | 8.74E-11 | 2.06E-10 |
| ZNF705G | ENSG00000215372 | 12.251019 | 3.614830 | 8.82E-11 | 2.08E-10 |
| CXCL9 | ENSG00000138755 | 12.241002 | 3.613650 | 5.56E-44 | 6.99E-43 |
| GRIA4 | ENSG00000152578 | 12.207051 | 3.609643 | 4.08E-19 | 1.55E-18 |
| SLC30A10 | ENSG00000196660 | 12.193096 | 3.607993 | 5.89E-12 | 1.50E-11 |
| LAG3 | ENSG00000089692 | 12.175868 | 3.605953 | 3.89E-40 | 4.11E-39 |
| PLA2G2D | ENSG00000117215 | 12.121409 | 3.599485 | 4.29E-22 | 1.89E-21 |
| EPS8L3 | ENSG00000198758 | 12.086854 | 3.595367 | 7.21E-17 | 2.44E-16 |
| SPIC | ENSG00000166211 | 12.079135 | 3.594445 | 8.22E-26 | 4.39E-25 |
| ANTXRL | ENSG00000274209 | 12.061690 | 3.592360 | 2.28E-08 | 4.60E-08 |
| NKG7 | ENSG00000105374 | 12.039094 | 3.589655 | 5.76E-60 | 1.41E-58 |
| NEUROD4 | ENSG00000123307 | 12.038887 | 3.589630 | 1.14E-07 | 2.20E-07 |
| TM4SF19 | ENSG00000145107 | 12.026984 | 3.588203 | 9.50E-26 | 5.06E-25 |
| DMRT1 | ENSG00000137090 | 12.002224 | 3.585230 | 2.90E-14 | 8.45E-14 |
| CDH7 | ENSG00000081138 | 11.998382 | 3.584768 | 1.06E-09 | 2.34E-09 |
| INHBE | ENSG00000139269 | 11.989786 | 3.583734 | 5.39E-26 | 2.90E-25 |
| TMEM179 | ENSG00000258986 | 11.976491 | 3.582133 | 1.06E-21 | 4.59E-21 |
| KRT6B | ENSG00000185479 | 11.963451 | 3.580562 | 8.89E-10 | 1.97E-09 |
| BTBD16 | ENSG00000138152 | 11.940598 | 3.577803 | 4.68E-50 | 7.93E-49 |
| ESM1 | ENSG00000164283 | 11.919477 | 3.575249 | 9.87E-72 | 3.79E-70 |
| LRRC14B | ENSG00000185028 | 11.848512 | 3.566634 | 6.85E-19 | 2.57E-18 |
| KLK2 | ENSG00000167751 | 11.833926 | 3.564857 | 4.07E-10 | 9.23E-10 |
| GBP5 | ENSG00000154451 | 11.812462 | 3.562238 | 4.92E-50 | 8.33E-49 |
| ST8SIA4 | ENSG00000113532 | 11.742534 | 3.553672 | 1.03E-95 | 8.16E-94 |
| HAPLN1 | ENSG00000145681 | 11.738343 | 3.553157 | 1.43E-37 | 1.34E-36 |
| FASLG | ENSG00000117560 | 11.721072 | 3.551033 | 2.07E-53 | 3.99E-52 |
| REG3A | ENSG00000172016 | 11.689089 | 3.547091 | 7.69E-09 | 1.60E-08 |
| GIP | ENSG00000159224 | 11.679719 | 3.545934 | 2.60E-10 | 5.97E-10 |
| SLC10A1 | ENSG00000100652 | 11.661994 | 3.543743 | 1.95E-15 | 6.05E-15 |
| EOMES | ENSG00000163508 | 11.639346 | 3.540938 | 5.08E-49 | 8.13E-48 |
| UGT2B4 | ENSG00000156096 | 11.636258 | 3.540555 | 1.77E-07 | 3.37E-07 |
| SEZ6L | ENSG00000100095 | 11.614099 | 3.537805 | 6.54E-18 | 2.34E-17 |
| FATE1 | ENSG00000147378 | 11.572705 | 3.532654 | 2.72E-66 | 8.76E-65 |
| GTSF1L | ENSG00000124196 | 11.553600 | 3.530271 | 9.36E-21 | 3.88E-20 |
| FGA | ENSG00000171560 | 11.547525 | 3.529512 | 7.25E-10 | 1.61E-09 |
| OBP2A | ENSG00000122136 | 11.547039 | 3.529451 | 8.11E-23 | 3.70E-22 |
| HOXB13 | ENSG00000159184 | 11.527900 | 3.527058 | 6.29E-11 | 1.50E-10 |
| ADAMTS14 | ENSG00000138316 | 11.525600 | 3.526770 | 3.82E-39 | 3.85E-38 |
| MUC3A | ENSG00000169894 | 11.520502 | 3.526132 | 8.18E-49 | 1.30E-47 |
| IGFBP3 | ENSG00000146674 | 11.519211 | 3.525970 | 1.81E-73 | 7.50E-72 |
| TNFSF9 | ENSG00000125657 | 11.510506 | 3.524879 | 8.76E-59 | 2.04E-57 |
| LY6H | ENSG00000176956 | 11.455677 | 3.517991 | 7.48E-28 | 4.40E-27 |
| WISP2 | ENSG00000064205 | 11.429016 | 3.514629 | 1.05E-20 | 4.34E-20 |
| VEGFA | ENSG00000112715 | 11.426055 | 3.514255 | 9.85E-98 | 8.24E-96 |
| ZNF683 | ENSG00000176083 | 11.410480 | 3.512288 | 3.60E-48 | 5.55E-47 |
| GDF6 | ENSG00000156466 | 11.402628 | 3.511295 | 5.97E-39 | 5.97E-38 |
| DLX5 | ENSG00000105880 | 11.384208 | 3.508962 | 3.30E-68 | 1.14E-66 |
| PIK3R6 | ENSG00000276231 | 11.351804 | 3.504850 | 1.17E-62 | 3.20E-61 |
| KLRK1 | ENSG00000213809 | 11.340636 | 3.503430 | 2.39E-40 | 2.55E-39 |
| CXorf65 | ENSG00000204165 | 11.324702 | 3.501401 | 4.12E-37 | 3.78E-36 |
| SLC17A9 | ENSG00000101194 | 11.320236 | 3.500832 | 3.42E-46 | 4.86E-45 |
| CXCL11 | ENSG00000169248 | 11.273824 | 3.494905 | 1.96E-41 | 2.21E-40 |
| C6orf223 | ENSG00000181577 | 11.265245 | 3.493807 | 7.70E-48 | 1.18E-46 |
| ABCB11 | ENSG00000073734 | 11.251775 | 3.492081 | 2.62E-11 | 6.40E-11 |
| LAT | ENSG00000213658 | 11.218920 | 3.487862 | 4.27E-45 | 5.69E-44 |
| KRT32 | ENSG00000108759 | 11.211298 | 3.486881 | 3.53E-27 | 2.01E-26 |
| LOX | ENSG00000113083 | 11.205369 | 3.486118 | 7.07E-36 | 6.12E-35 |
| C17orf74 | ENSG00000184560 | 11.193015 | 3.484527 | 6.81E-30 | 4.46E-29 |
| KRT36 | ENSG00000126337 | 11.168863 | 3.481410 | 3.85E-44 | 4.88E-43 |
| ATP8B3 | ENSG00000130270 | 11.133639 | 3.476853 | 6.94E-34 | 5.50E-33 |
| HK2 | ENSG00000159399 | 11.100917 | 3.472607 | 1.33E-84 | 7.84E-83 |
| NOL3 | ENSG00000140939 | 11.096206 | 3.471995 | 3.18E-131 | 6.08E-129 |
| PITX1 | ENSG00000069011 | 11.063475 | 3.467733 | 1.24E-12 | 3.29E-12 |
| TEX11 | ENSG00000120498 | 11.054965 | 3.466623 | 3.47E-24 | 1.70E-23 |
| CDH8 | ENSG00000150394 | 11.017758 | 3.461759 | 1.65E-45 | 2.25E-44 |
| NR0B1 | ENSG00000169297 | 11.007161 | 3.460371 | 7.04E-07 | 1.29E-06 |
| C4BPA | ENSG00000123838 | 11.000673 | 3.459520 | 7.56E-11 | 1.79E-10 |
| RFX8 | ENSG00000196460 | 10.980136 | 3.456824 | 9.12E-51 | 1.60E-49 |
| HEATR9 | ENSG00000270379 | 10.969580 | 3.455436 | 1.51E-33 | 1.18E-32 |
| LGALS4 | ENSG00000171747 | 10.967887 | 3.455214 | 4.50E-15 | 1.37E-14 |
| GAD2 | ENSG00000136750 | 10.929494 | 3.450155 | 4.48E-17 | 1.54E-16 |
| POU5F1 | ENSG00000204531 | 10.921381 | 3.449083 | 2.42E-61 | 6.21E-60 |
| MT3 | ENSG00000087250 | 10.901509 | 3.446456 | 1.65E-19 | 6.40E-19 |
| SCN10A | ENSG00000185313 | 10.873899 | 3.442797 | 9.23E-20 | 3.63E-19 |
| AGBL1 | ENSG00000166748 | 10.843603 | 3.438772 | 1.57E-13 | 4.38E-13 |
| EFCAB3 | ENSG00000172421 | 10.821744 | 3.435861 | 2.86E-29 | 1.81E-28 |
| ATP2B2 | ENSG00000157087 | 10.816496 | 3.435161 | 1.88E-36 | 1.68E-35 |
| SIRPG | ENSG00000089012 | 10.816116 | 3.435111 | 1.25E-41 | 1.42E-40 |
| C3 | ENSG00000125730 | 10.792943 | 3.432016 | 9.69E-43 | 1.16E-41 |
| SPP2 | ENSG00000072080 | 10.778923 | 3.430141 | 1.33E-08 | 2.72E-08 |
| CD8B | ENSG00000172116 | 10.770045 | 3.428952 | 5.76E-41 | 6.35E-40 |
| HOXD13 | ENSG00000128714 | 10.717263 | 3.421865 | 7.78E-28 | 4.57E-27 |
| GABRG2 | ENSG00000113327 | 10.657313 | 3.413772 | 1.26E-07 | 2.43E-07 |
| PRG3 | ENSG00000156575 | 10.649900 | 3.412768 | 1.78E-09 | 3.86E-09 |
| EPO | ENSG00000130427 | 10.629851 | 3.410050 | 8.52E-12 | 2.15E-11 |
| RNASET2 | ENSG00000026297 | 10.623553 | 3.409195 | 2.81E-62 | 7.61E-61 |
| ADAM7 | ENSG00000069206 | 10.597270 | 3.405621 | 5.07E-09 | 1.07E-08 |
| PDCD1 | ENSG00000188389 | 10.566252 | 3.401392 | 2.09E-35 | 1.77E-34 |
| MAGEC3 | ENSG00000165509 | 10.553433 | 3.399641 | 1.28E-12 | 3.39E-12 |
| MSLNL | ENSG00000162006 | 10.552149 | 3.399465 | 4.80E-10 | 1.08E-09 |
| NKAIN1 | ENSG00000084628 | 10.548230 | 3.398929 | 1.79E-32 | 1.33E-31 |
| CXCL10 | ENSG00000169245 | 10.539418 | 3.397723 | 1.63E-44 | 2.10E-43 |
| CACNA1B | ENSG00000148408 | 10.524944 | 3.395741 | 1.30E-12 | 3.45E-12 |
| UNC5A | ENSG00000113763 | 10.508305 | 3.393458 | 9.48E-34 | 7.48E-33 |
| IFITM5 | ENSG00000206013 | 10.495737 | 3.391732 | 5.17E-16 | 1.66E-15 |
| SLITRK5 | ENSG00000165300 | 10.466298 | 3.387679 | 2.90E-25 | 1.51E-24 |
| CHRND | ENSG00000135902 | 10.462789 | 3.387196 | 6.86E-25 | 3.50E-24 |
| KCNK17 | ENSG00000124780 | 10.437887 | 3.383758 | 4.46E-16 | 1.44E-15 |
| ANKRD33 | ENSG00000167612 | 10.417273 | 3.380906 | 5.39E-21 | 2.26E-20 |
| STRA8 | ENSG00000146857 | 10.416238 | 3.380762 | 4.65E-46 | 6.54E-45 |
| CSN3 | ENSG00000171209 | 10.395013 | 3.377820 | 5.48E-08 | 1.08E-07 |
| SLC17A2 | ENSG00000112337 | 10.352094 | 3.371851 | 2.08E-27 | 1.19E-26 |
| SERPINA9 | ENSG00000170054 | 10.351105 | 3.371713 | 1.59E-14 | 4.70E-14 |
| CLVS2 | ENSG00000146352 | 10.324153 | 3.367951 | 4.83E-19 | 1.82E-18 |
| FCGR3A | ENSG00000203747 | 10.322962 | 3.367785 | 9.01E-71 | 3.37E-69 |
| ITGAX | ENSG00000140678 | 10.286077 | 3.362621 | 8.68E-75 | 3.71E-73 |
| ICOS | ENSG00000163600 | 10.242047 | 3.356432 | 4.86E-43 | 5.90E-42 |
| TNFRSF4 | ENSG00000186827 | 10.217107 | 3.352915 | 2.39E-75 | 1.05E-73 |
| C2orf83 | ENSG00000042304 | 10.191331 | 3.349271 | 4.59E-12 | 1.18E-11 |
| CD27 | ENSG00000139193 | 10.174332 | 3.346862 | 2.77E-40 | 2.95E-39 |
| INHA | ENSG00000123999 | 10.170239 | 3.346282 | 2.47E-14 | 7.22E-14 |
| KLK12 | ENSG00000186474 | 10.162725 | 3.345215 | 7.80E-10 | 1.73E-09 |
| THEG | ENSG00000105549 | 10.117909 | 3.338839 | 6.46E-13 | 1.74E-12 |
| GZMK | ENSG00000113088 | 10.114331 | 3.338329 | 1.94E-37 | 1.80E-36 |
| TEX13B | ENSG00000170925 | 10.075430 | 3.332770 | 8.03E-14 | 2.28E-13 |
| PADI3 | ENSG00000142619 | 10.065407 | 3.331334 | 7.18E-09 | 1.49E-08 |
| MTCP1 | ENSG00000214827 | 10.029320 | 3.326152 | 1.99E-84 | 1.16E-82 |
| CCL5 | ENSG00000271503 | 9.951485 | 3.314912 | 4.26E-54 | 8.39E-53 |
| CALY | ENSG00000130643 | 9.919913 | 3.310328 | 2.60E-13 | 7.17E-13 |
| ADCY8 | ENSG00000155897 | 9.913295 | 3.309365 | 1.29E-13 | 3.62E-13 |
| PRND | ENSG00000171864 | 9.910706 | 3.308988 | 3.53E-21 | 1.49E-20 |
| ELSPBP1 | ENSG00000169393 | 9.907948 | 3.308586 | 1.82E-06 | 3.23E-06 |
| B4GALNT4 | ENSG00000182272 | 9.879128 | 3.304384 | 7.18E-19 | 2.69E-18 |
| OR2H1 | ENSG00000204688 | 9.857189 | 3.301176 | 1.27E-10 | 2.96E-10 |
| OR5C1 | ENSG00000148215 | 9.855468 | 3.300924 | 5.50E-11 | 1.32E-10 |
| GBX2 | ENSG00000168505 | 9.821579 | 3.295955 | 1.34E-26 | 7.43E-26 |
| MYO3A | ENSG00000095777 | 9.813383 | 3.294751 | 3.98E-60 | 9.82E-59 |
| PKD2L1 | ENSG00000107593 | 9.755970 | 3.286285 | 6.64E-32 | 4.81E-31 |
| MS4A14 | ENSG00000166928 | 9.752540 | 3.285778 | 4.24E-60 | 1.05E-58 |
| FCRL4 | ENSG00000163518 | 9.740252 | 3.283959 | 6.37E-11 | 1.52E-10 |
| KLRF2 | ENSG00000256797 | 9.737920 | 3.283614 | 1.23E-17 | 4.32E-17 |
| DCSTAMP | ENSG00000164935 | 9.716033 | 3.280367 | 1.14E-32 | 8.55E-32 |
| NETO2 | ENSG00000171208 | 9.667419 | 3.273131 | 1.60E-96 | 1.28E-94 |
| ELOVL2 | ENSG00000197977 | 9.647726 | 3.270189 | 2.73E-39 | 2.77E-38 |
| C16orf92 | ENSG00000167194 | 9.632739 | 3.267946 | 1.53E-10 | 3.57E-10 |
| BPIFB1 | ENSG00000125999 | 9.623021 | 3.266490 | 2.75E-12 | 7.13E-12 |
| CYP2A13 | ENSG00000197838 | 9.618286 | 3.265780 | 7.30E-10 | 1.62E-09 |
| RLBP1 | ENSG00000140522 | 9.605667 | 3.263886 | 4.87E-12 | 1.25E-11 |
| IDO1 | ENSG00000131203 | 9.598454 | 3.262802 | 8.53E-45 | 1.12E-43 |
| CD7 | ENSG00000173762 | 9.598016 | 3.262736 | 1.71E-37 | 1.60E-36 |
| KRT84 | ENSG00000161849 | 9.595637 | 3.262379 | 6.28E-19 | 2.36E-18 |
| ENO2 | ENSG00000111674 | 9.584571 | 3.260714 | 5.47E-84 | 3.15E-82 |
| DCLK3 | ENSG00000163673 | 9.573395 | 3.259031 | 7.82E-47 | 1.14E-45 |
| DLX1 | ENSG00000144355 | 9.538400 | 3.253747 | 3.37E-46 | 4.79E-45 |
| TGM5 | ENSG00000104055 | 9.517579 | 3.250595 | 1.99E-12 | 5.21E-12 |
| CD68 | ENSG00000129226 | 9.510279 | 3.249488 | 2.32E-64 | 6.92E-63 |
| SORCS3 | ENSG00000156395 | 9.506427 | 3.248903 | 1.96E-15 | 6.09E-15 |
| LHFPL5 | ENSG00000197753 | 9.500564 | 3.248013 | 6.10E-19 | 2.29E-18 |
| GNLY | ENSG00000115523 | 9.464337 | 3.242501 | 1.30E-59 | 3.14E-58 |
| KPNA7 | ENSG00000185467 | 9.457878 | 3.241517 | 2.08E-31 | 1.47E-30 |
| PROP1 | ENSG00000175325 | 9.457783 | 3.241502 | 4.78E-12 | 1.23E-11 |
| EN1 | ENSG00000163064 | 9.454863 | 3.241056 | 7.66E-22 | 3.34E-21 |
| CDK5R2 | ENSG00000171450 | 9.433877 | 3.237851 | 1.98E-13 | 5.51E-13 |
| CAPN12 | ENSG00000182472 | 9.430596 | 3.237349 | 3.00E-51 | 5.38E-50 |
| CSRP3 | ENSG00000129170 | 9.428134 | 3.236972 | 7.55E-14 | 2.15E-13 |
| CPA4 | ENSG00000128510 | 9.397141 | 3.232222 | 1.25E-13 | 3.51E-13 |
| CTHRC1 | ENSG00000164932 | 9.391989 | 3.231431 | 5.26E-32 | 3.82E-31 |
| IGF2BP3 | ENSG00000136231 | 9.378686 | 3.229386 | 9.56E-17 | 3.21E-16 |
| TPD52L3 | ENSG00000170777 | 9.374274 | 3.228707 | 8.83E-07 | 1.60E-06 |
| PPFIA4 | ENSG00000143847 | 9.357927 | 3.226189 | 8.03E-44 | 1.00E-42 |
| CA1 | ENSG00000133742 | 9.350144 | 3.224989 | 5.45E-13 | 1.47E-12 |
| PTPRN | ENSG00000054356 | 9.338306 | 3.223161 | 1.39E-15 | 4.35E-15 |
| FAM153A | ENSG00000170074 | 9.314507 | 3.219479 | 1.67E-22 | 7.53E-22 |
| ONECUT3 | ENSG00000205922 | 9.293187 | 3.216173 | 5.74E-17 | 1.95E-16 |
| DTHD1 | ENSG00000197057 | 9.270530 | 3.212652 | 4.43E-36 | 3.86E-35 |
| CDR1 | ENSG00000184258 | 9.265512 | 3.211871 | 2.33E-21 | 9.90E-21 |
| CXCR3 | ENSG00000186810 | 9.255851 | 3.210366 | 1.96E-44 | 2.51E-43 |
| NPIPA7 | ENSG00000214967 | 9.234409 | 3.207020 | 5.08E-08 | 1.00E-07 |
| TRIB3 | ENSG00000101255 | 9.233623 | 3.206897 | 8.99E-50 | 1.50E-48 |
| MSC | ENSG00000178860 | 9.192939 | 3.200526 | 2.93E-44 | 3.74E-43 |
| UBD | ENSG00000213886 | 9.184360 | 3.199179 | 2.73E-39 | 2.77E-38 |
| FAM163A | ENSG00000143340 | 9.147454 | 3.193370 | 2.02E-53 | 3.89E-52 |
| TCN1 | ENSG00000134827 | 9.131360 | 3.190830 | 1.73E-10 | 4.00E-10 |
| C1orf127 | ENSG00000175262 | 9.127673 | 3.190247 | 6.21E-68 | 2.11E-66 |
| TP73 | ENSG00000078900 | 9.104210 | 3.186534 | 1.92E-41 | 2.17E-40 |
| GZMA | ENSG00000145649 | 9.097285 | 3.185436 | 3.70E-53 | 7.04E-52 |
| CD177 | ENSG00000204936 | 9.077569 | 3.182306 | 2.27E-11 | 5.57E-11 |
| AGBL1 | ENSG00000273540 | 9.051349 | 3.178133 | 4.36E-07 | 8.08E-07 |
| TUBA3C | ENSG00000198033 | 9.033402 | 3.175269 | 1.07E-07 | 2.07E-07 |
| GCG | ENSG00000115263 | 9.023034 | 3.173613 | 9.05E-07 | 1.64E-06 |
| MROH2A | ENSG00000185038 | 9.021752 | 3.173408 | 2.25E-15 | 6.96E-15 |
| DDX53 | ENSG00000184735 | 9.012768 | 3.171970 | 5.85E-08 | 1.15E-07 |
| CTD-2521M24.13 | ENSG00000269720 | 9.003794 | 3.170533 | 1.74E-26 | 9.54E-26 |
| CST7 | ENSG00000077984 | 8.987925 | 3.167988 | 9.38E-52 | 1.72E-50 |
| NPIPB13 | ENSG00000198064 | 8.986831 | 3.167812 | 5.33E-27 | 3.01E-26 |
| HSD17B3 | ENSG00000130948 | 8.984879 | 3.167499 | 5.07E-32 | 3.69E-31 |
| ZDHHC22 | ENSG00000177108 | 8.975782 | 3.166038 | 1.55E-13 | 4.33E-13 |
| H2BFM | ENSG00000101812 | 8.968083 | 3.164800 | 7.32E-08 | 1.43E-07 |
| OBP2B | ENSG00000171102 | 8.963163 | 3.164008 | 9.87E-28 | 5.77E-27 |
| LINC00890 | ENSG00000260802 | 8.942343 | 3.160653 | 1.23E-07 | 2.36E-07 |
| OR9Q1 | ENSG00000186509 | 8.936873 | 3.159770 | 1.56E-23 | 7.41E-23 |
| ADAM2 | ENSG00000104755 | 8.931137 | 3.158844 | 1.12E-06 | 2.01E-06 |
| LILRB4 | ENSG00000186818 | 8.905104 | 3.154632 | 1.51E-61 | 3.95E-60 |
| CATSPER4 | ENSG00000188782 | 8.885887 | 3.151516 | 1.68E-09 | 3.65E-09 |
| TMEM45A | ENSG00000181458 | 8.865188 | 3.148151 | 1.67E-36 | 1.48E-35 |
| NANOS2 | ENSG00000188425 | 8.846130 | 3.145047 | 1.10E-10 | 2.58E-10 |
| ANKRD60 | ENSG00000124227 | 8.838744 | 3.143841 | 5.68E-08 | 1.12E-07 |
| C16orf74 | ENSG00000154102 | 8.812401 | 3.139535 | 9.86E-33 | 7.41E-32 |
| TRPA1 | ENSG00000104321 | 8.783756 | 3.134838 | 8.28E-37 | 7.51E-36 |
| NMU | ENSG00000109255 | 8.772679 | 3.133017 | 1.71E-17 | 6.00E-17 |
| SCD | ENSG00000099194 | 8.753647 | 3.129884 | 2.47E-68 | 8.65E-67 |
| AIM2 | ENSG00000163568 | 8.740134 | 3.127655 | 1.14E-35 | 9.82E-35 |
| RGS1 | ENSG00000090104 | 8.715116 | 3.123520 | 5.22E-56 | 1.11E-54 |
| MAGEA10 | ENSG00000124260 | 8.704866 | 3.121822 | 1.00E-06 | 1.81E-06 |
| C7orf33 | ENSG00000170279 | 8.688259 | 3.119067 | 2.72E-13 | 7.49E-13 |
| FCGR1A | ENSG00000150337 | 8.686458 | 3.118768 | 3.33E-62 | 8.96E-61 |
| GZMH | ENSG00000100450 | 8.686119 | 3.118712 | 5.34E-59 | 1.25E-57 |
| UBE2C | ENSG00000175063 | 8.682730 | 3.118149 | 1.64E-35 | 1.40E-34 |
| GAS2L3 | ENSG00000139354 | 8.681380 | 3.117924 | 3.18E-72 | 1.25E-70 |
| ADH7 | ENSG00000196344 | 8.676318 | 3.117083 | 8.35E-11 | 1.97E-10 |
| PRIMA1 | ENSG00000175785 | 8.664072 | 3.115045 | 3.70E-19 | 1.41E-18 |
| CST2 | ENSG00000170369 | 8.663739 | 3.114990 | 5.71E-14 | 1.64E-13 |
| SLCO1B3 | ENSG00000111700 | 8.661360 | 3.114594 | 2.18E-06 | 3.83E-06 |
| PSG3 | ENSG00000221826 | 8.661208 | 3.114568 | 1.42E-10 | 3.30E-10 |
| PERM1 | ENSG00000187642 | 8.656071 | 3.113712 | 1.85E-29 | 1.19E-28 |
| TBC1D26 | ENSG00000255104 | 8.653759 | 3.113327 | 6.51E-21 | 2.72E-20 |
| RP11-872D17.8 | ENSG00000254979 | 8.647600 | 3.112300 | 2.52E-51 | 4.54E-50 |
| ADH1A | ENSG00000187758 | 8.642156 | 3.111391 | 1.38E-07 | 2.64E-07 |
| BATF | ENSG00000156127 | 8.637146 | 3.110555 | 1.22E-50 | 2.12E-49 |
| TRIM63 | ENSG00000158022 | 8.636430 | 3.110435 | 3.86E-09 | 8.19E-09 |
| TREML1 | ENSG00000161911 | 8.627462 | 3.108936 | 1.25E-58 | 2.89E-57 |
| VSX1 | ENSG00000100987 | 8.621290 | 3.107904 | 1.27E-20 | 5.22E-20 |
| SMIM23 | ENSG00000185662 | 8.617230 | 3.107224 | 6.29E-17 | 2.14E-16 |
| CSAG1 | ENSG00000198930 | 8.603094 | 3.104856 | 2.76E-13 | 7.61E-13 |
| RTP5 | ENSG00000188011 | 8.593513 | 3.103248 | 1.52E-25 | 8.03E-25 |
| MS4A4E | ENSG00000214787 | 8.589547 | 3.102582 | 6.36E-46 | 8.87E-45 |
| ANGPT2 | ENSG00000091879 | 8.583866 | 3.101628 | 4.00E-61 | 1.02E-59 |
| NPIPB5 | ENSG00000243716 | 8.549089 | 3.095771 | 2.33E-36 | 2.07E-35 |
| SAPCD1 | ENSG00000228727 | 8.539039 | 3.094074 | 2.45E-30 | 1.63E-29 |
| PDCL2 | ENSG00000163440 | 8.527016 | 3.092041 | 7.15E-11 | 1.70E-10 |
| MUC16 | ENSG00000181143 | 8.514558 | 3.089932 | 1.43E-14 | 4.25E-14 |
| UGT1A5 | ENSG00000240224 | 8.513688 | 3.089784 | 8.83E-13 | 2.36E-12 |
| CTSW | ENSG00000172543 | 8.501603 | 3.087735 | 6.68E-52 | 1.23E-50 |
| NKX2-3 | ENSG00000119919 | 8.488230 | 3.085464 | 4.57E-10 | 1.03E-09 |
| LINC00521 | ENSG00000175699 | 8.481780 | 3.084367 | 5.82E-10 | 1.30E-09 |
| HHLA2 | ENSG00000114455 | 8.477793 | 3.083689 | 3.06E-27 | 1.75E-26 |
| CGREF1 | ENSG00000138028 | 8.434448 | 3.076294 | 6.46E-48 | 9.90E-47 |
| TUBA3D | ENSG00000075886 | 8.430837 | 3.075676 | 2.26E-12 | 5.91E-12 |
| ATG9B | ENSG00000181652 | 8.412130 | 3.072471 | 3.25E-33 | 2.50E-32 |
| TBX15 | ENSG00000092607 | 8.404393 | 3.071144 | 1.66E-37 | 1.55E-36 |
| SLC16A3 | ENSG00000141526 | 8.395292 | 3.069580 | 2.66E-95 | 2.09E-93 |
| IL2RB | ENSG00000100385 | 8.385578 | 3.067910 | 9.39E-72 | 3.61E-70 |
| TBC1D26 | ENSG00000214946 | 8.383375 | 3.067531 | 3.86E-20 | 1.55E-19 |
| DAZ1 | ENSG00000188120 | 8.378638 | 3.066716 | 1.65E-05 | 2.75E-05 |
| C1QL4 | ENSG00000186897 | 8.364170 | 3.064222 | 2.34E-18 | 8.53E-18 |
| PSG9 | ENSG00000183668 | 8.348213 | 3.061467 | 1.91E-05 | 3.17E-05 |
| AL589743.1 | ENSG00000279508 | 8.346761 | 3.061216 | 1.68E-27 | 9.68E-27 |
| CRTAM | ENSG00000109943 | 8.343568 | 3.060664 | 4.60E-46 | 6.47E-45 |
| CD2 | ENSG00000116824 | 8.342079 | 3.060407 | 1.32E-48 | 2.07E-47 |
| KREMEN2 | ENSG00000131650 | 8.339491 | 3.059959 | 1.55E-27 | 8.99E-27 |
| LRRC71 | ENSG00000160838 | 8.329482 | 3.058227 | 5.27E-36 | 4.58E-35 |
| FMR1NB | ENSG00000176988 | 8.278471 | 3.049364 | 1.60E-10 | 3.71E-10 |
| MS4A15 | ENSG00000166961 | 8.264176 | 3.046871 | 8.63E-17 | 2.90E-16 |
| MGARP | ENSG00000137463 | 8.258854 | 3.045942 | 4.46E-20 | 1.78E-19 |
| DAND5 | ENSG00000179284 | 8.245402 | 3.043590 | 1.86E-27 | 1.07E-26 |
| ZIC5 | ENSG00000139800 | 8.239755 | 3.042602 | 1.44E-08 | 2.95E-08 |
| PRG2 | ENSG00000186652 | 8.232681 | 3.041362 | 9.82E-29 | 6.05E-28 |
| VSTM2A | ENSG00000170419 | 8.229908 | 3.040876 | 1.24E-06 | 2.23E-06 |
| HIST1H2AI | ENSG00000196747 | 8.206422 | 3.036753 | 6.46E-23 | 2.96E-22 |
| HORMAD1 | ENSG00000143452 | 8.200623 | 3.035734 | 6.88E-30 | 4.50E-29 |
| GIMD1 | ENSG00000250298 | 8.196415 | 3.034993 | 1.36E-05 | 2.28E-05 |
| CD300LF | ENSG00000186074 | 8.194457 | 3.034648 | 2.91E-74 | 1.22E-72 |
| TNFRSF18 | ENSG00000186891 | 8.192039 | 3.034223 | 2.72E-44 | 3.48E-43 |
| SIRPD | ENSG00000125900 | 8.158629 | 3.028327 | 1.14E-38 | 1.13E-37 |
| DCAF8L1 | ENSG00000226372 | 8.155249 | 3.027729 | 9.24E-06 | 1.57E-05 |
| PSORS1C1 | ENSG00000204540 | 8.140144 | 3.025054 | 8.98E-66 | 2.81E-64 |
| PSG1 | ENSG00000231924 | 8.111322 | 3.019937 | 2.35E-12 | 6.13E-12 |
| SMIM9 | ENSG00000203870 | 8.094218 | 3.016892 | 2.79E-21 | 1.18E-20 |
| CTLA4 | ENSG00000163599 | 8.078598 | 3.014105 | 1.29E-35 | 1.10E-34 |
| REG1A | ENSG00000115386 | 8.072875 | 3.013083 | 6.33E-12 | 1.61E-11 |
| CD300A | ENSG00000167851 | 8.057921 | 3.010408 | 2.97E-77 | 1.40E-75 |
| PYHIN1 | ENSG00000163564 | 8.053320 | 3.009584 | 7.88E-50 | 1.32E-48 |
| TYMP | ENSG00000025708 | 8.028972 | 3.005215 | 9.72E-79 | 4.84E-77 |
| CEACAM20 | ENSG00000273777 | 8.023391 | 3.004212 | 8.09E-16 | 2.57E-15 |
| ZAP70 | ENSG00000115085 | 8.020837 | 3.003753 | 2.93E-53 | 5.61E-52 |
| NKX6-3 | ENSG00000165066 | 8.008597 | 3.001550 | 1.68E-11 | 4.18E-11 |
| NKX2-2 | ENSG00000125820 | 8.002258 | 3.000407 | 1.66E-08 | 3.38E-08 |
| PCSK6 | ENSG00000140479 | 7.990817 | 2.998343 | 4.62E-40 | 4.87E-39 |
| RGSL1 | ENSG00000121446 | 7.974775 | 2.995444 | 2.32E-22 | 1.03E-21 |
| GABRE | ENSG00000102287 | 7.971593 | 2.994868 | 5.26E-38 | 5.06E-37 |
| FGG | ENSG00000171557 | 7.967519 | 2.994131 | 3.51E-07 | 6.55E-07 |
| P4HA3 | ENSG00000149380 | 7.961350 | 2.993013 | 3.00E-34 | 2.42E-33 |
| SIGLECL1 | ENSG00000179213 | 7.945972 | 2.990224 | 1.49E-10 | 3.46E-10 |
| MMP12 | ENSG00000262406 | 7.936323 | 2.988471 | 4.14E-09 | 8.75E-09 |
| PRLHR | ENSG00000119973 | 7.927559 | 2.986877 | 7.14E-15 | 2.15E-14 |
| CHRNA6 | ENSG00000147434 | 7.922853 | 2.986020 | 1.76E-28 | 1.07E-27 |
| SIGLEC10 | ENSG00000142512 | 7.921049 | 2.985692 | 4.84E-63 | 1.36E-61 |
| UGT1A10 | ENSG00000242515 | 7.918555 | 2.985237 | 6.93E-08 | 1.36E-07 |
| C1orf61 | ENSG00000125462 | 7.890359 | 2.980091 | 4.04E-20 | 1.62E-19 |
| GPR42 | ENSG00000126251 | 7.875912 | 2.977447 | 1.45E-12 | 3.83E-12 |
| FAM26F | ENSG00000188820 | 7.863138 | 2.975105 | 1.25E-50 | 2.18E-49 |
| MMP16 | ENSG00000156103 | 7.861592 | 2.974821 | 1.52E-33 | 1.19E-32 |
| SPATA21 | ENSG00000187144 | 7.861581 | 2.974820 | 3.15E-20 | 1.27E-19 |
| LILRB1 | ENSG00000104972 | 7.844260 | 2.971637 | 1.42E-97 | 1.17E-95 |
| DQX1 | ENSG00000144045 | 7.840070 | 2.970867 | 2.18E-14 | 6.38E-14 |
| CIDEC | ENSG00000187288 | 7.835306 | 2.969990 | 1.30E-09 | 2.84E-09 |
| PTCRA | ENSG00000171611 | 7.833224 | 2.969606 | 8.23E-49 | 1.31E-47 |
| ZBP1 | ENSG00000124256 | 7.830601 | 2.969123 | 5.99E-42 | 6.94E-41 |
| CCL25 | ENSG00000131142 | 7.830271 | 2.969062 | 4.51E-18 | 1.63E-17 |
| GAL3ST1 | ENSG00000128242 | 7.821278 | 2.967404 | 1.23E-51 | 2.25E-50 |
| SLA2 | ENSG00000101082 | 7.816581 | 2.966538 | 8.06E-55 | 1.63E-53 |
| APOBEC3H | ENSG00000100298 | 7.811946 | 2.965682 | 7.31E-62 | 1.94E-60 |
| C3orf22 | ENSG00000180697 | 7.804657 | 2.964335 | 1.14E-28 | 7.00E-28 |
| RASD2 | ENSG00000100302 | 7.789976 | 2.961619 | 4.51E-64 | 1.33E-62 |
| SPATA3 | ENSG00000173699 | 7.789611 | 2.961551 | 3.10E-09 | 6.61E-09 |
| CASP5 | ENSG00000137757 | 7.788743 | 2.961391 | 1.86E-45 | 2.54E-44 |
| GOLGA8A | ENSG00000175265 | 7.761219 | 2.956283 | 2.06E-30 | 1.38E-29 |
| C1QB | ENSG00000173369 | 7.761158 | 2.956272 | 1.13E-61 | 2.97E-60 |
| NLRP5 | ENSG00000171487 | 7.755492 | 2.955218 | 4.76E-09 | 1.00E-08 |
| PRKCG | ENSG00000126583 | 7.753803 | 2.954904 | 2.93E-14 | 8.54E-14 |
| POU5F2 | ENSG00000248483 | 7.752358 | 2.954635 | 7.57E-21 | 3.15E-20 |
| TROAP | ENSG00000135451 | 7.750986 | 2.954380 | 1.24E-41 | 1.42E-40 |
| HIST1H2BM | ENSG00000273703 | 7.749416 | 2.954088 | 1.75E-13 | 4.87E-13 |
| SERPINC1 | ENSG00000117601 | 7.748643 | 2.953944 | 3.41E-08 | 6.80E-08 |
| DIRAS2 | ENSG00000165023 | 7.740609 | 2.952447 | 3.08E-45 | 4.13E-44 |
| LIM2 | ENSG00000105370 | 7.733580 | 2.951137 | 2.19E-25 | 1.15E-24 |
| HCG27 | ENSG00000206344 | 7.716850 | 2.948012 | 1.04E-48 | 1.64E-47 |
| TIGIT | ENSG00000181847 | 7.698826 | 2.944638 | 1.13E-38 | 1.12E-37 |
| KIAA0319 | ENSG00000137261 | 7.667912 | 2.938834 | 1.01E-32 | 7.61E-32 |
| COL1A1 | ENSG00000108821 | 7.660802 | 2.937496 | 3.89E-29 | 2.45E-28 |
| ABCC12 | ENSG00000140798 | 7.653522 | 2.936124 | 4.58E-09 | 9.65E-09 |
| CD3D | ENSG00000167286 | 7.641505 | 2.933857 | 1.89E-42 | 2.24E-41 |
| GAL3ST4 | ENSG00000197093 | 7.636006 | 2.932818 | 6.06E-80 | 3.15E-78 |
| PARVG | ENSG00000138964 | 7.628680 | 2.931434 | 6.06E-81 | 3.24E-79 |
| IRX4 | ENSG00000113430 | 7.628180 | 2.931339 | 1.74E-07 | 3.32E-07 |
| IGLL5 | ENSG00000254709 | 7.625248 | 2.930784 | 3.04E-13 | 8.34E-13 |
| PRAME | ENSG00000185686 | 7.620973 | 2.929975 | 4.91E-15 | 1.49E-14 |
| KLK15 | ENSG00000174562 | 7.619743 | 2.929742 | 1.61E-04 | 2.49E-04 |
| PLXDC1 | ENSG00000161381 | 7.618027 | 2.929417 | 6.93E-76 | 3.09E-74 |
| CD1A | ENSG00000158477 | 7.602823 | 2.926535 | 2.10E-16 | 6.90E-16 |
| OTOA | ENSG00000155719 | 7.589752 | 2.924053 | 8.60E-55 | 1.74E-53 |
| TMIGD3 | ENSG00000121933 | 7.567264 | 2.919772 | 1.94E-71 | 7.43E-70 |
| CCR5 | ENSG00000160791 | 7.565496 | 2.919435 | 4.67E-49 | 7.51E-48 |
| DMBX1 | ENSG00000197587 | 7.542408 | 2.915025 | 2.26E-08 | 4.57E-08 |
| CARD17 | ENSG00000255221 | 7.537953 | 2.914173 | 7.08E-28 | 4.17E-27 |
| PNPLA5 | ENSG00000100341 | 7.535319 | 2.913669 | 5.53E-09 | 1.16E-08 |
| FCGR1B | ENSG00000198019 | 7.518546 | 2.910454 | 1.82E-61 | 4.72E-60 |
| QRFPR | ENSG00000186867 | 7.513988 | 2.909579 | 7.29E-40 | 7.60E-39 |
| MSH4 | ENSG00000057468 | 7.494070 | 2.905749 | 4.86E-32 | 3.54E-31 |
| TGFBI | ENSG00000120708 | 7.493920 | 2.905721 | 1.47E-19 | 5.70E-19 |
| COX4I2 | ENSG00000131055 | 7.487098 | 2.904407 | 2.21E-38 | 2.16E-37 |
| KRT33A | ENSG00000006059 | 7.487068 | 2.904401 | 1.32E-11 | 3.29E-11 |
| C5orf58 | ENSG00000234511 | 7.474971 | 2.902068 | 3.08E-48 | 4.76E-47 |
| MSMB | ENSG00000263639 | 7.462793 | 2.899716 | 6.17E-09 | 1.29E-08 |
| TRIM72 | ENSG00000177238 | 7.460337 | 2.899241 | 6.76E-12 | 1.72E-11 |
| STH | ENSG00000256762 | 7.453174 | 2.897855 | 1.43E-16 | 4.75E-16 |
| DNASE2B | ENSG00000137976 | 7.446843 | 2.896629 | 3.22E-26 | 1.74E-25 |
| SLC5A5 | ENSG00000105641 | 7.443252 | 2.895933 | 2.70E-21 | 1.15E-20 |
| KIF18B | ENSG00000186185 | 7.426465 | 2.892676 | 8.17E-38 | 7.75E-37 |
| PATL2 | ENSG00000229474 | 7.419864 | 2.891393 | 2.30E-56 | 4.94E-55 |
| RP4-576H24.4 | ENSG00000260861 | 7.404695 | 2.888440 | 4.01E-19 | 1.52E-18 |
| C15orf53 | ENSG00000175779 | 7.388470 | 2.885276 | 9.29E-25 | 4.70E-24 |
| FCRL3 | ENSG00000160856 | 7.387741 | 2.885133 | 9.79E-30 | 6.38E-29 |
| TNNI3 | ENSG00000129991 | 7.385304 | 2.884657 | 9.29E-09 | 1.92E-08 |
| AKR1B10 | ENSG00000198074 | 7.379609 | 2.883544 | 2.47E-08 | 4.98E-08 |
| AIPL1 | ENSG00000129221 | 7.371404 | 2.881939 | 1.90E-12 | 4.98E-12 |
| C12orf40 | ENSG00000180116 | 7.366731 | 2.881025 | 2.53E-12 | 6.59E-12 |
| KIR3DX1 | ENSG00000104970 | 7.359429 | 2.879594 | 7.65E-29 | 4.73E-28 |
| SPINK6 | ENSG00000178172 | 7.357095 | 2.879136 | 6.57E-08 | 1.29E-07 |
| GNRH1 | ENSG00000147437 | 7.352329 | 2.878201 | 3.05E-49 | 4.95E-48 |
| AC007326.1 | ENSG00000279560 | 7.344404 | 2.876645 | 2.45E-16 | 8.02E-16 |
| ARG1 | ENSG00000118520 | 7.342379 | 2.876248 | 8.78E-17 | 2.95E-16 |
| SLAMF7 | ENSG00000026751 | 7.333487 | 2.874499 | 4.31E-39 | 4.33E-38 |
| MYBL2 | ENSG00000101057 | 7.333421 | 2.874486 | 8.15E-32 | 5.89E-31 |
| PSTPIP1 | ENSG00000140368 | 7.319893 | 2.871823 | 1.83E-52 | 3.41E-51 |
| FFAR4 | ENSG00000186188 | 7.319224 | 2.871691 | 3.45E-46 | 4.90E-45 |
| SLC26A10 | ENSG00000135502 | 7.311976 | 2.870261 | 1.40E-26 | 7.71E-26 |
| SAG | ENSG00000130561 | 7.287259 | 2.865376 | 1.11E-16 | 3.71E-16 |
| SMTNL1 | ENSG00000214872 | 7.283244 | 2.864581 | 4.73E-31 | 3.26E-30 |
| RFX6 | ENSG00000185002 | 7.272868 | 2.862524 | 7.95E-11 | 1.88E-10 |
| AMELX | ENSG00000125363 | 7.270859 | 2.862126 | 3.20E-09 | 6.82E-09 |
| PLIN2 | ENSG00000147872 | 7.260742 | 2.860117 | 9.33E-52 | 1.71E-50 |
| CCDC78 | ENSG00000162004 | 7.258364 | 2.859644 | 3.27E-25 | 1.70E-24 |
| FKBP10 | ENSG00000141756 | 7.246494 | 2.857283 | 8.84E-57 | 1.93E-55 |
| SLN | ENSG00000170290 | 7.238635 | 2.855718 | 1.84E-08 | 3.73E-08 |
| C8orf22 | ENSG00000168333 | 7.235009 | 2.854995 | 4.43E-07 | 8.21E-07 |
| DNTT | ENSG00000107447 | 7.232488 | 2.854492 | 4.76E-06 | 8.22E-06 |
| LRRTM4 | ENSG00000176204 | 7.219765 | 2.851952 | 1.38E-13 | 3.88E-13 |
| STRC | ENSG00000242866 | 7.213226 | 2.850645 | 6.53E-21 | 2.73E-20 |
| RSPO4 | ENSG00000101282 | 7.210793 | 2.850158 | 8.08E-16 | 2.57E-15 |
| AMH | ENSG00000104899 | 7.210738 | 2.850147 | 1.69E-21 | 7.24E-21 |
| P2RX3 | ENSG00000109991 | 7.196766 | 2.847349 | 7.76E-28 | 4.56E-27 |
| TRIM9 | ENSG00000100505 | 7.191398 | 2.846272 | 1.13E-58 | 2.62E-57 |
| KRT77 | ENSG00000189182 | 7.166867 | 2.841343 | 2.72E-08 | 5.45E-08 |
| PIK3R5 | ENSG00000141506 | 7.147807 | 2.837501 | 1.58E-81 | 8.68E-80 |
| CDC25C | ENSG00000158402 | 7.138500 | 2.835621 | 4.21E-41 | 4.68E-40 |
| BEST4 | ENSG00000142959 | 7.134257 | 2.834763 | 8.97E-25 | 4.54E-24 |
| ADH4 | ENSG00000198099 | 7.126613 | 2.833217 | 4.10E-06 | 7.09E-06 |
| UBASH3A | ENSG00000160185 | 7.117711 | 2.831413 | 1.80E-44 | 2.31E-43 |
| C8orf74 | ENSG00000171060 | 7.097913 | 2.827395 | 3.15E-12 | 8.14E-12 |
| HPCA | ENSG00000121905 | 7.096444 | 2.827096 | 1.88E-45 | 2.56E-44 |
| BRINP2 | ENSG00000198797 | 7.094297 | 2.826660 | 1.08E-10 | 2.54E-10 |
| NME8 | ENSG00000086288 | 7.091462 | 2.826083 | 9.03E-55 | 1.82E-53 |
| TMEM92 | ENSG00000167105 | 7.091314 | 2.826053 | 2.12E-19 | 8.17E-19 |
| CLEC12A | ENSG00000172322 | 7.088766 | 2.825534 | 3.34E-46 | 4.75E-45 |
| CXCR4 | ENSG00000121966 | 7.085172 | 2.824803 | 9.44E-95 | 7.35E-93 |
| NEIL3 | ENSG00000109674 | 7.067430 | 2.821186 | 6.73E-37 | 6.12E-36 |
| ARHGAP22 | ENSG00000128805 | 7.062469 | 2.820173 | 7.23E-97 | 5.89E-95 |
| TBC1D3D | ENSG00000274419 | 7.051646 | 2.817960 | 1.83E-06 | 3.23E-06 |
| CSF3R | ENSG00000119535 | 7.049977 | 2.817619 | 6.25E-60 | 1.52E-58 |
| PTPRH | ENSG00000080031 | 7.048147 | 2.817244 | 1.00E-13 | 2.84E-13 |
| KRBA1 | ENSG00000133619 | 7.036345 | 2.814826 | 2.07E-82 | 1.16E-80 |
| CDSN | ENSG00000204539 | 7.030507 | 2.813629 | 1.17E-24 | 5.87E-24 |
| CADM3 | ENSG00000162706 | 7.028345 | 2.813185 | 6.48E-15 | 1.96E-14 |
| TRPM2 | ENSG00000142185 | 7.027188 | 2.812947 | 1.96E-73 | 8.09E-72 |
| 4-Mar | ENSG00000144583 | 7.018011 | 2.811062 | 3.63E-39 | 3.67E-38 |
| PRTN3 | ENSG00000196415 | 7.010010 | 2.809417 | 1.36E-14 | 4.03E-14 |
| HBM | ENSG00000206177 | 7.009671 | 2.809347 | 1.60E-08 | 3.26E-08 |
| HRH2 | ENSG00000113749 | 7.006505 | 2.808695 | 2.73E-28 | 1.64E-27 |
| AURKB | ENSG00000178999 | 7.005909 | 2.808572 | 2.55E-38 | 2.48E-37 |
| IQCA1L | ENSG00000278685 | 7.001302 | 2.807623 | 5.84E-14 | 1.68E-13 |
| TRIML2 | ENSG00000179046 | 6.997256 | 2.806789 | 2.59E-09 | 5.55E-09 |
| STXBP5L | ENSG00000145087 | 6.993548 | 2.806025 | 1.12E-07 | 2.17E-07 |
| DLX4 | ENSG00000108813 | 6.982801 | 2.803806 | 3.22E-32 | 2.37E-31 |
| CCR8 | ENSG00000179934 | 6.981379 | 2.803512 | 8.06E-25 | 4.09E-24 |
| AQP8 | ENSG00000103375 | 6.970468 | 2.801255 | 7.09E-21 | 2.95E-20 |
| C20orf141 | ENSG00000258713 | 6.959947 | 2.799076 | 9.50E-09 | 1.96E-08 |
| TEX19 | ENSG00000182459 | 6.951904 | 2.797408 | 2.46E-09 | 5.28E-09 |
| CILP2 | ENSG00000160161 | 6.950364 | 2.797089 | 2.74E-14 | 8.01E-14 |
| KRTAP5-10 | ENSG00000204572 | 6.945059 | 2.795987 | 3.03E-28 | 1.82E-27 |
| UTS2 | ENSG00000049247 | 6.943074 | 2.795575 | 1.31E-20 | 5.41E-20 |
| HLA-DQB2 | ENSG00000232629 | 6.927742 | 2.792385 | 1.51E-35 | 1.29E-34 |
| ADD2 | ENSG00000075340 | 6.927452 | 2.792325 | 1.68E-26 | 9.23E-26 |
| CARD14 | ENSG00000141527 | 6.925609 | 2.791941 | 2.54E-26 | 1.38E-25 |
| VWCE | ENSG00000167992 | 6.905973 | 2.787845 | 3.74E-28 | 2.24E-27 |
| PPEF1 | ENSG00000086717 | 6.905964 | 2.787843 | 1.47E-30 | 9.91E-30 |
| CD84 | ENSG00000066294 | 6.903775 | 2.787385 | 2.52E-49 | 4.10E-48 |
| MMP26 | ENSG00000167346 | 6.902326 | 2.787083 | 1.28E-06 | 2.29E-06 |
| UGT1A1 | ENSG00000241635 | 6.897429 | 2.786059 | 1.27E-17 | 4.48E-17 |
| BHLHE41 | ENSG00000123095 | 6.894787 | 2.785506 | 7.23E-78 | 3.53E-76 |
| MYO1A | ENSG00000166866 | 6.889895 | 2.784482 | 4.17E-27 | 2.37E-26 |
| CST1 | ENSG00000170373 | 6.881827 | 2.782792 | 7.29E-10 | 1.62E-09 |
| CSPG4 | ENSG00000173546 | 6.878234 | 2.782038 | 1.09E-65 | 3.40E-64 |
| PLA2G5 | ENSG00000127472 | 6.873112 | 2.780963 | 1.46E-30 | 9.86E-30 |
| PDIA2 | ENSG00000185615 | 6.869816 | 2.780271 | 1.98E-11 | 4.89E-11 |
| C1QC | ENSG00000159189 | 6.854844 | 2.777124 | 4.31E-59 | 1.01E-57 |
| HAL | ENSG00000084110 | 6.853904 | 2.776926 | 3.83E-19 | 1.46E-18 |
| SH2D2A | ENSG00000027869 | 6.842982 | 2.774625 | 2.53E-45 | 3.41E-44 |
| SLAMF8 | ENSG00000158714 | 6.836559 | 2.773270 | 6.75E-45 | 8.88E-44 |
| SPANXB1 | ENSG00000227234 | 6.830913 | 2.772078 | 6.78E-05 | 1.08E-04 |
| CYP7A1 | ENSG00000167910 | 6.823248 | 2.770459 | 1.47E-10 | 3.42E-10 |
| ARHGEF39 | ENSG00000137135 | 6.822976 | 2.770401 | 2.37E-108 | 2.62E-106 |
| TMPRSS9 | ENSG00000178297 | 6.820357 | 2.769847 | 3.70E-36 | 3.23E-35 |
| LOXL2 | ENSG00000134013 | 6.805726 | 2.766749 | 3.40E-43 | 4.15E-42 |
| FOXB2 | ENSG00000204612 | 6.803364 | 2.766248 | 1.87E-10 | 4.33E-10 |
| GJC1 | ENSG00000182963 | 6.800712 | 2.765686 | 8.45E-75 | 3.62E-73 |
| SPDEF | ENSG00000124664 | 6.800642 | 2.765671 | 3.01E-13 | 8.27E-13 |
| ASCL1 | ENSG00000139352 | 6.796927 | 2.764883 | 3.87E-10 | 8.77E-10 |
| AGAP2 | ENSG00000135439 | 6.791601 | 2.763752 | 9.19E-95 | 7.19E-93 |
| ADGRE1 | ENSG00000174837 | 6.791407 | 2.763710 | 5.39E-42 | 6.26E-41 |
| OLFML2B | ENSG00000162745 | 6.785482 | 2.762451 | 2.40E-59 | 5.69E-58 |
| E2F8 | ENSG00000129173 | 6.777693 | 2.760794 | 3.04E-39 | 3.08E-38 |
| PARP15 | ENSG00000173200 | 6.749628 | 2.754808 | 1.22E-46 | 1.77E-45 |
| ADM | ENSG00000148926 | 6.747257 | 2.754301 | 2.94E-71 | 1.12E-69 |
| CD72 | ENSG00000137101 | 6.747121 | 2.754272 | 2.69E-57 | 5.93E-56 |
| CCER2 | ENSG00000262484 | 6.742230 | 2.753226 | 1.69E-25 | 8.88E-25 |
| SLC37A2 | ENSG00000134955 | 6.732870 | 2.751222 | 2.47E-65 | 7.57E-64 |
| ASPHD1 | ENSG00000174939 | 6.730116 | 2.750631 | 5.31E-43 | 6.43E-42 |
| SP9 | ENSG00000217236 | 6.700962 | 2.744368 | 1.16E-05 | 1.95E-05 |
| ITIH3 | ENSG00000162267 | 6.692557 | 2.742558 | 5.67E-14 | 1.63E-13 |
| EBF2 | ENSG00000221818 | 6.687075 | 2.741375 | 4.02E-46 | 5.69E-45 |
| RASAL3 | ENSG00000105122 | 6.671711 | 2.738057 | 4.05E-83 | 2.30E-81 |
| PRR30 | ENSG00000186143 | 6.670045 | 2.737697 | 1.84E-07 | 3.50E-07 |
| HJURP | ENSG00000123485 | 6.669370 | 2.737550 | 1.30E-37 | 1.22E-36 |
| SIRPB1 | ENSG00000101307 | 6.667905 | 2.737233 | 4.03E-37 | 3.70E-36 |
| MEP1A | ENSG00000112818 | 6.666514 | 2.736933 | 4.66E-18 | 1.68E-17 |
| SIT1 | ENSG00000137078 | 6.634626 | 2.730015 | 4.87E-37 | 4.44E-36 |
| RUNX3 | ENSG00000020633 | 6.618692 | 2.726546 | 5.72E-62 | 1.52E-60 |
| OR56B1 | ENSG00000181023 | 6.613619 | 2.725440 | 4.73E-16 | 1.53E-15 |
| CXCR6 | ENSG00000172215 | 6.607783 | 2.724166 | 4.13E-46 | 5.85E-45 |
| YPEL4 | ENSG00000166793 | 6.604045 | 2.723350 | 8.87E-61 | 2.23E-59 |
| SLC1A3 | ENSG00000079215 | 6.602923 | 2.723105 | 7.00E-51 | 1.23E-49 |
| LHCGR | ENSG00000138039 | 6.601234 | 2.722736 | 1.85E-06 | 3.27E-06 |
| MYO1G | ENSG00000136286 | 6.594297 | 2.721219 | 1.42E-54 | 2.85E-53 |
| ETV7 | ENSG00000010030 | 6.583705 | 2.718900 | 6.24E-60 | 1.52E-58 |
| C11orf21 | ENSG00000110665 | 6.582679 | 2.718675 | 3.32E-50 | 5.68E-49 |
| TPSG1 | ENSG00000116176 | 6.573203 | 2.716596 | 1.79E-13 | 5.00E-13 |
| CREB3L3 | ENSG00000060566 | 6.569589 | 2.715803 | 6.13E-17 | 2.08E-16 |
| NOG | ENSG00000183691 | 6.568428 | 2.715548 | 6.77E-16 | 2.16E-15 |
| OLFML2A | ENSG00000185585 | 6.562588 | 2.714265 | 3.58E-61 | 9.12E-60 |
| ZNF716 | ENSG00000182111 | 6.556694 | 2.712969 | 4.28E-10 | 9.69E-10 |
| PKMYT1 | ENSG00000127564 | 6.553884 | 2.712350 | 1.08E-48 | 1.71E-47 |
| CYP21A2 | ENSG00000231852 | 6.547458 | 2.710935 | 4.86E-27 | 2.76E-26 |
| CD3E | ENSG00000198851 | 6.546205 | 2.710659 | 5.00E-41 | 5.54E-40 |
| FCHO1 | ENSG00000130475 | 6.542009 | 2.709734 | 2.22E-47 | 3.33E-46 |
| PLAC8L1 | ENSG00000173261 | 6.540804 | 2.709468 | 2.24E-39 | 2.28E-38 |
| RPA4 | ENSG00000204086 | 6.537829 | 2.708812 | 5.14E-32 | 3.74E-31 |
| P2RX7 | ENSG00000089041 | 6.532318 | 2.707595 | 3.11E-92 | 2.31E-90 |
| TYROBP | ENSG00000011600 | 6.521294 | 2.705158 | 3.97E-72 | 1.55E-70 |
| HK3 | ENSG00000160883 | 6.513019 | 2.703326 | 6.84E-56 | 1.44E-54 |
| PRF1 | ENSG00000180644 | 6.504480 | 2.701434 | 3.61E-61 | 9.19E-60 |
| NKPD1 | ENSG00000179846 | 6.498907 | 2.700197 | 1.55E-36 | 1.38E-35 |
| LAMA4 | ENSG00000112769 | 6.494219 | 2.699156 | 2.35E-76 | 1.06E-74 |
| CCL4 | ENSG00000275302 | 6.482204 | 2.696484 | 1.43E-47 | 2.17E-46 |
| IGSF6 | ENSG00000140749 | 6.480741 | 2.696159 | 2.08E-69 | 7.50E-68 |
| CRNN | ENSG00000143536 | 6.480139 | 2.696025 | 8.41E-10 | 1.86E-09 |
| CTAGE6 | ENSG00000271321 | 6.477459 | 2.695428 | 3.58E-24 | 1.75E-23 |
| DOK3 | ENSG00000146094 | 6.473744 | 2.694600 | 4.43E-91 | 3.22E-89 |
| CTC-435M10.3 | ENSG00000255730 | 6.467900 | 2.693297 | 1.61E-41 | 1.83E-40 |
| GRM4 | ENSG00000124493 | 6.459999 | 2.691534 | 6.78E-16 | 2.16E-15 |
| SIGLEC12 | ENSG00000254521 | 6.447153 | 2.688662 | 2.47E-20 | 1.00E-19 |
| ACAN | ENSG00000157766 | 6.440963 | 2.687276 | 6.93E-31 | 4.75E-30 |
| IL21R | ENSG00000103522 | 6.436601 | 2.686299 | 2.15E-49 | 3.52E-48 |
| UCN | ENSG00000163794 | 6.423700 | 2.683405 | 8.23E-47 | 1.20E-45 |
| LTA | ENSG00000226979 | 6.423259 | 2.683306 | 3.93E-38 | 3.80E-37 |
| MXD3 | ENSG00000213347 | 6.422360 | 2.683103 | 2.01E-54 | 4.02E-53 |
| CA6 | ENSG00000131686 | 6.422302 | 2.683090 | 4.19E-22 | 1.85E-21 |
| RETN | ENSG00000104918 | 6.416768 | 2.681847 | 1.59E-21 | 6.84E-21 |
| SHOX2 | ENSG00000168779 | 6.415663 | 2.681598 | 9.15E-16 | 2.90E-15 |
| PAQR6 | ENSG00000160781 | 6.415326 | 2.681523 | 9.28E-35 | 7.67E-34 |
| CTSE | ENSG00000196188 | 6.401395 | 2.678386 | 1.32E-13 | 3.70E-13 |
| GZMB | ENSG00000100453 | 6.400990 | 2.678295 | 9.22E-47 | 1.35E-45 |
| LAIR1 | ENSG00000167613 | 6.399661 | 2.677995 | 4.42E-69 | 1.59E-67 |
| NCR1 | ENSG00000189430 | 6.396682 | 2.677324 | 3.06E-44 | 3.89E-43 |
| VNN3 | ENSG00000093134 | 6.390767 | 2.675989 | 6.51E-22 | 2.84E-21 |
| OR11A1 | ENSG00000204694 | 6.386800 | 2.675093 | 1.29E-09 | 2.81E-09 |
| CCDC88B | ENSG00000168071 | 6.379579 | 2.673461 | 5.52E-62 | 1.47E-60 |
| SLAMF9 | ENSG00000162723 | 6.366983 | 2.670610 | 1.04E-10 | 2.44E-10 |
| CPLX2 | ENSG00000145920 | 6.359992 | 2.669025 | 5.82E-06 | 9.98E-06 |
| 14-Sep | ENSG00000154997 | 6.347825 | 2.666262 | 3.84E-10 | 8.71E-10 |
| SULT4A1 | ENSG00000130540 | 6.347005 | 2.666076 | 9.16E-12 | 2.31E-11 |
| TLX3 | ENSG00000164438 | 6.334138 | 2.663148 | 1.79E-05 | 2.96E-05 |
| BCL2A1 | ENSG00000140379 | 6.333445 | 2.662990 | 8.92E-37 | 8.07E-36 |
| MUC5B | ENSG00000117983 | 6.329236 | 2.662031 | 6.72E-13 | 1.81E-12 |
| TNNT1 | ENSG00000105048 | 6.328900 | 2.661955 | 2.98E-09 | 6.37E-09 |
| XCL2 | ENSG00000143185 | 6.328597 | 2.661886 | 4.80E-37 | 4.39E-36 |
| LIPI | ENSG00000188992 | 6.325084 | 2.661085 | 1.33E-10 | 3.11E-10 |
| OSCAR | ENSG00000170909 | 6.321460 | 2.660258 | 6.06E-67 | 1.98E-65 |
| IL17REL | ENSG00000188263 | 6.316608 | 2.659150 | 1.36E-24 | 6.81E-24 |
| HMHB1 | ENSG00000158497 | 6.313566 | 2.658455 | 6.64E-18 | 2.37E-17 |
| C1QA | ENSG00000173372 | 6.303505 | 2.656154 | 1.47E-54 | 2.94E-53 |
| SHOX | ENSG00000185960 | 6.297197 | 2.654710 | 3.34E-13 | 9.13E-13 |
| ETV3L | ENSG00000253831 | 6.293899 | 2.653954 | 3.48E-15 | 1.07E-14 |
| ISL2 | ENSG00000159556 | 6.290448 | 2.653163 | 5.51E-15 | 1.67E-14 |
| IGLL1 | ENSG00000128322 | 6.285458 | 2.652018 | 9.93E-11 | 2.34E-10 |
| DLX6 | ENSG00000006377 | 6.281915 | 2.651204 | 4.04E-39 | 4.06E-38 |
| AC016549.1 | ENSG00000281325 | 6.276523 | 2.649966 | 1.74E-15 | 5.44E-15 |
| JSRP1 | ENSG00000167476 | 6.271397 | 2.648787 | 5.98E-20 | 2.37E-19 |
| PHOX2B | ENSG00000109132 | 6.270672 | 2.648620 | 2.25E-07 | 4.25E-07 |
| CLEC1B | ENSG00000165682 | 6.270164 | 2.648503 | 3.18E-38 | 3.09E-37 |
| PYDC1 | ENSG00000169900 | 6.264096 | 2.647106 | 1.44E-09 | 3.14E-09 |
| SLFN12L | ENSG00000205045 | 6.260446 | 2.646265 | 4.40E-45 | 5.86E-44 |
| BARX2 | ENSG00000043039 | 6.250022 | 2.643861 | 1.96E-50 | 3.39E-49 |
| PODNL1 | ENSG00000132000 | 6.248828 | 2.643586 | 3.41E-20 | 1.37E-19 |
| TBPL2 | ENSG00000182521 | 6.245823 | 2.642892 | 1.60E-12 | 4.21E-12 |
| HIST1H3H | ENSG00000278828 | 6.245575 | 2.642834 | 6.78E-27 | 3.82E-26 |
| ZNF80 | ENSG00000174255 | 6.244404 | 2.642564 | 2.26E-26 | 1.24E-25 |
| CTC-360G5.8 | ENSG00000269547 | 6.239561 | 2.641445 | 1.09E-21 | 4.72E-21 |
| IL12RB1 | ENSG00000096996 | 6.223283 | 2.637676 | 6.88E-63 | 1.92E-61 |
| VWF | ENSG00000110799 | 6.222388 | 2.637468 | 1.03E-42 | 1.23E-41 |
| CARMIL2 | ENSG00000159753 | 6.219187 | 2.636726 | 3.49E-35 | 2.94E-34 |
| KLHL6 | ENSG00000172578 | 6.218020 | 2.636455 | 1.23E-63 | 3.59E-62 |
| LRRC74B | ENSG00000187905 | 6.211269 | 2.634888 | 1.85E-11 | 4.57E-11 |
| HOXA13 | ENSG00000106031 | 6.208094 | 2.634150 | 1.88E-16 | 6.21E-16 |
| C1QL2 | ENSG00000144119 | 6.206811 | 2.633852 | 2.07E-06 | 3.64E-06 |
| C19orf33 | ENSG00000167644 | 6.203093 | 2.632988 | 3.50E-26 | 1.90E-25 |
| CD247 | ENSG00000198821 | 6.198461 | 2.631910 | 1.07E-59 | 2.60E-58 |
| OTOF | ENSG00000115155 | 6.188749 | 2.629648 | 1.22E-35 | 1.04E-34 |
| ARHGAP9 | ENSG00000123329 | 6.188103 | 2.629497 | 2.27E-60 | 5.65E-59 |
| IFNK | ENSG00000147896 | 6.182928 | 2.628290 | 2.50E-14 | 7.31E-14 |
| UHRF1 | ENSG00000276043 | 6.182741 | 2.628247 | 6.76E-46 | 9.38E-45 |
| SLC10A6 | ENSG00000145283 | 6.175650 | 2.626591 | 2.27E-39 | 2.32E-38 |
| EFCAB8 | ENSG00000215529 | 6.166843 | 2.624532 | 4.73E-19 | 1.79E-18 |
| IL12RB2 | ENSG00000081985 | 6.165492 | 2.624216 | 1.89E-15 | 5.88E-15 |
| CLEC2D | ENSG00000069493 | 6.158962 | 2.622687 | 3.19E-68 | 1.10E-66 |
| ADCY2 | ENSG00000078295 | 6.151608 | 2.620964 | 4.91E-18 | 1.77E-17 |
| NMB | ENSG00000197696 | 6.149446 | 2.620456 | 2.10E-35 | 1.78E-34 |
| TBC1D3E | ENSG00000278599 | 6.148965 | 2.620344 | 3.55E-08 | 7.08E-08 |
| SERPINA7 | ENSG00000123561 | 6.147119 | 2.619910 | 1.14E-08 | 2.35E-08 |
| ALPI | ENSG00000163295 | 6.142615 | 2.618853 | 2.57E-09 | 5.52E-09 |
| ZNF395 | ENSG00000186918 | 6.139192 | 2.618049 | 1.19E-89 | 8.33E-88 |
| RGS20 | ENSG00000147509 | 6.118799 | 2.613248 | 5.91E-20 | 2.35E-19 |
| TBC1D10C | ENSG00000175463 | 6.117284 | 2.612891 | 2.63E-51 | 4.73E-50 |
| HLX | ENSG00000136630 | 6.116893 | 2.612799 | 3.40E-63 | 9.62E-62 |
| GOLGA6B | ENSG00000215186 | 6.108811 | 2.610892 | 3.37E-12 | 8.70E-12 |
| TMPRSS11F | ENSG00000198092 | 6.099080 | 2.608592 | 3.68E-11 | 8.92E-11 |
| LTB4R | ENSG00000213903 | 6.098566 | 2.608470 | 5.25E-68 | 1.80E-66 |
| RGS5 | ENSG00000143248 | 6.091358 | 2.606764 | 5.93E-43 | 7.17E-42 |
| WDR97 | ENSG00000179698 | 6.087473 | 2.605844 | 1.98E-30 | 1.33E-29 |
| ITIH4 | ENSG00000055955 | 6.084332 | 2.605099 | 7.08E-20 | 2.80E-19 |
| IKZF3 | ENSG00000161405 | 6.080932 | 2.604293 | 2.33E-38 | 2.27E-37 |
| TPRG1 | ENSG00000188001 | 6.075195 | 2.602931 | 1.76E-29 | 1.13E-28 |
| CCL4L2 | ENSG00000276070 | 6.075088 | 2.602905 | 4.46E-28 | 2.66E-27 |
| DLX2 | ENSG00000115844 | 6.068448 | 2.601328 | 3.85E-27 | 2.19E-26 |
| PTH | ENSG00000152266 | 6.067564 | 2.601118 | 1.54E-07 | 2.96E-07 |
| SIGLEC1 | ENSG00000088827 | 6.064719 | 2.600441 | 4.78E-46 | 6.72E-45 |
| AANAT | ENSG00000129673 | 6.059887 | 2.599291 | 2.70E-40 | 2.88E-39 |
| VIM | ENSG00000026025 | 6.057160 | 2.598642 | 7.73E-116 | 1.00E-113 |
| TMC8 | ENSG00000167895 | 6.057088 | 2.598624 | 2.06E-61 | 5.30E-60 |
| IQGAP3 | ENSG00000183856 | 6.057020 | 2.598608 | 1.65E-44 | 2.12E-43 |
| ATP1A3 | ENSG00000105409 | 6.046164 | 2.596020 | 1.17E-19 | 4.56E-19 |
| ACTL8 | ENSG00000117148 | 6.024002 | 2.590722 | 3.04E-05 | 4.95E-05 |
| FCER1G | ENSG00000158869 | 6.007274 | 2.586710 | 3.78E-68 | 1.30E-66 |
| FOXM1 | ENSG00000111206 | 5.999805 | 2.584916 | 1.36E-40 | 1.46E-39 |
| DCST1 | ENSG00000163357 | 5.995481 | 2.583876 | 3.07E-27 | 1.76E-26 |
| FGB | ENSG00000171564 | 5.995278 | 2.583827 | 8.42E-07 | 1.53E-06 |
| THBS4 | ENSG00000113296 | 5.992154 | 2.583075 | 7.11E-16 | 2.26E-15 |
| CENPA | ENSG00000115163 | 5.981777 | 2.580574 | 6.31E-34 | 5.01E-33 |
| BIRC5 | ENSG00000089685 | 5.981181 | 2.580430 | 2.08E-38 | 2.04E-37 |
| TSPAN16 | ENSG00000130167 | 5.980558 | 2.580280 | 2.37E-22 | 1.06E-21 |
| PLEKHN1 | ENSG00000187583 | 5.973992 | 2.578695 | 9.66E-50 | 1.61E-48 |
| LILRA6 | ENSG00000244482 | 5.963023 | 2.576044 | 1.78E-53 | 3.43E-52 |
| LPCAT1 | ENSG00000153395 | 5.959091 | 2.575092 | 4.81E-76 | 2.15E-74 |
| CALB2 | ENSG00000172137 | 5.955749 | 2.574283 | 9.47E-21 | 3.92E-20 |
| ITGAL | ENSG00000005844 | 5.954160 | 2.573898 | 1.97E-55 | 4.08E-54 |
| CD3G | ENSG00000160654 | 5.953906 | 2.573836 | 1.02E-37 | 9.65E-37 |
| RTEL1-TNFRSF6B | ENSG00000026036 | 5.947491 | 2.572281 | 3.96E-43 | 4.82E-42 |
| LAPTM5 | ENSG00000162511 | 5.944079 | 2.571453 | 1.26E-72 | 5.02E-71 |
| CCL20 | ENSG00000115009 | 5.937049 | 2.569746 | 4.98E-15 | 1.52E-14 |
| TNFSF13B | ENSG00000102524 | 5.934730 | 2.569182 | 1.43E-45 | 1.96E-44 |
| MUC22 | ENSG00000261272 | 5.930988 | 2.568272 | 6.46E-16 | 2.07E-15 |
| C4orf47 | ENSG00000205129 | 5.929291 | 2.567860 | 4.76E-62 | 1.27E-60 |
| SYT8 | ENSG00000149043 | 5.916466 | 2.564736 | 2.68E-08 | 5.39E-08 |
| GP9 | ENSG00000169704 | 5.914245 | 2.564194 | 1.75E-24 | 8.74E-24 |
| PILRB | ENSG00000121716 | 5.912442 | 2.563754 | 5.36E-25 | 2.75E-24 |
| SLAMF6 | ENSG00000162739 | 5.902874 | 2.561418 | 1.38E-39 | 1.42E-38 |
| MYO1F | ENSG00000142347 | 5.900792 | 2.560909 | 3.14E-75 | 1.36E-73 |
| NPFFR1 | ENSG00000148734 | 5.898430 | 2.560331 | 4.61E-20 | 1.84E-19 |
| NR5A1 | ENSG00000136931 | 5.896479 | 2.559854 | 1.27E-06 | 2.27E-06 |
| FCRL6 | ENSG00000181036 | 5.892700 | 2.558929 | 8.83E-50 | 1.47E-48 |
| OR51E1 | ENSG00000180785 | 5.889810 | 2.558221 | 8.46E-44 | 1.06E-42 |
| RPE65 | ENSG00000116745 | 5.888637 | 2.557934 | 1.53E-23 | 7.26E-23 |
| NPIPA3 | ENSG00000224712 | 5.884039 | 2.556807 | 1.99E-18 | 7.28E-18 |
| APOC2 | ENSG00000234906 | 5.880099 | 2.555840 | 6.57E-19 | 2.46E-18 |
| MNX1 | ENSG00000130675 | 5.876481 | 2.554953 | 3.85E-15 | 1.18E-14 |
| GSDMC | ENSG00000147697 | 5.848384 | 2.548038 | 4.48E-10 | 1.01E-09 |
| MAGEA6 | ENSG00000197172 | 5.848248 | 2.548004 | 3.39E-03 | 4.68E-03 |
| JAK3 | ENSG00000105639 | 5.846708 | 2.547625 | 1.43E-57 | 3.20E-56 |
| ZIC2 | ENSG00000043355 | 5.843936 | 2.546940 | 3.49E-07 | 6.52E-07 |
| KIR2DL3 | ENSG00000243772 | 5.840076 | 2.545987 | 7.57E-35 | 6.28E-34 |
| IGLON5 | ENSG00000142549 | 5.821155 | 2.541305 | 9.48E-09 | 1.96E-08 |
| NETO1 | ENSG00000166342 | 5.812840 | 2.539243 | 3.54E-19 | 1.35E-18 |
| TBX21 | ENSG00000073861 | 5.808965 | 2.538281 | 1.37E-61 | 3.59E-60 |
| SLFN14 | ENSG00000236320 | 5.803912 | 2.537026 | 2.86E-24 | 1.41E-23 |
| GFI1 | ENSG00000162676 | 5.803058 | 2.536813 | 5.69E-43 | 6.89E-42 |
| KLRC2 | ENSG00000205809 | 5.800410 | 2.536155 | 6.06E-22 | 2.65E-21 |
| NLRP8 | ENSG00000179709 | 5.797700 | 2.535481 | 2.99E-08 | 5.98E-08 |
| LILRB3 | ENSG00000204577 | 5.784809 | 2.532269 | 6.52E-61 | 1.65E-59 |
| KCTD19 | ENSG00000168676 | 5.784107 | 2.532094 | 9.44E-29 | 5.82E-28 |
| OR52N4 | ENSG00000181074 | 5.782247 | 2.531630 | 3.27E-29 | 2.06E-28 |
| PADI6 | ENSG00000276747 | 5.777240 | 2.530380 | 2.87E-12 | 7.46E-12 |
| CYP2F1 | ENSG00000197446 | 5.775834 | 2.530029 | 2.65E-11 | 6.49E-11 |
| SLCO1C1 | ENSG00000139155 | 5.775667 | 2.529987 | 1.18E-49 | 1.94E-48 |
| CCR6 | ENSG00000112486 | 5.766672 | 2.527739 | 1.97E-23 | 9.31E-23 |
| PRRT2 | ENSG00000167371 | 5.766623 | 2.527727 | 7.13E-38 | 6.79E-37 |
| CD96 | ENSG00000153283 | 5.766381 | 2.527666 | 2.19E-44 | 2.80E-43 |
| WFDC13 | ENSG00000168634 | 5.761956 | 2.526559 | 5.04E-13 | 1.37E-12 |
| C14orf180 | ENSG00000184601 | 5.756042 | 2.525077 | 1.05E-10 | 2.47E-10 |
| TREML4 | ENSG00000188056 | 5.755232 | 2.524874 | 1.68E-13 | 4.70E-13 |
| TNNI2 | ENSG00000130598 | 5.751723 | 2.523994 | 1.35E-40 | 1.45E-39 |
| SSX5 | ENSG00000165583 | 5.749100 | 2.523336 | 1.85E-07 | 3.52E-07 |
| SH2D1A | ENSG00000183918 | 5.739319 | 2.520879 | 1.02E-34 | 8.45E-34 |
| MARCO | ENSG00000019169 | 5.734341 | 2.519628 | 1.29E-17 | 4.54E-17 |
| PRAM1 | ENSG00000133246 | 5.729216 | 2.518338 | 4.88E-59 | 1.15E-57 |
| C11orf86 | ENSG00000173237 | 5.724102 | 2.517049 | 5.31E-09 | 1.11E-08 |
| GSTA3 | ENSG00000174156 | 5.722353 | 2.516608 | 2.06E-06 | 3.64E-06 |
| SASH3 | ENSG00000122122 | 5.716542 | 2.515143 | 6.83E-64 | 2.00E-62 |
| LRRTM1 | ENSG00000162951 | 5.709020 | 2.513243 | 3.29E-04 | 4.96E-04 |
| CPNE7 | ENSG00000178773 | 5.704052 | 2.511987 | 4.91E-17 | 1.68E-16 |
| IFNL1 | ENSG00000182393 | 5.698298 | 2.510531 | 9.70E-16 | 3.07E-15 |
| FGD2 | ENSG00000146192 | 5.686502 | 2.507541 | 3.32E-58 | 7.55E-57 |
| RAD54L | ENSG00000085999 | 5.684938 | 2.507145 | 6.34E-41 | 6.96E-40 |
| IL37 | ENSG00000125571 | 5.684581 | 2.507054 | 2.33E-13 | 6.45E-13 |
| CH17-360D5.1 | ENSG00000264717 | 5.682533 | 2.506534 | 1.04E-09 | 2.29E-09 |
| NOD2 | ENSG00000167207 | 5.672322 | 2.503939 | 2.47E-46 | 3.54E-45 |
| SLC12A5 | ENSG00000124140 | 5.670604 | 2.503502 | 2.89E-43 | 3.54E-42 |
| LINGO3 | ENSG00000220008 | 5.665727 | 2.502261 | 9.33E-37 | 8.44E-36 |
| KRT17 | ENSG00000128422 | 5.659638 | 2.500710 | 2.87E-09 | 6.13E-09 |
| SCNN1D | ENSG00000162572 | 5.655954 | 2.499770 | 9.00E-32 | 6.48E-31 |
| CRYGS | ENSG00000213139 | 5.653180 | 2.499063 | 1.86E-31 | 1.31E-30 |
| ANKRD30BL | ENSG00000163046 | 5.644375 | 2.496814 | 2.46E-08 | 4.95E-08 |
| C4orf50 | ENSG00000181215 | 5.637729 | 2.495114 | 1.42E-25 | 7.50E-25 |
| SAP30 | ENSG00000164105 | 5.628106 | 2.492649 | 4.84E-124 | 8.07E-122 |
| ADAMTS7 | ENSG00000136378 | 5.626654 | 2.492277 | 1.52E-68 | 5.39E-67 |
| MELK | ENSG00000165304 | 5.624263 | 2.491664 | 1.08E-39 | 1.11E-38 |
| HLA-F | ENSG00000204642 | 5.622427 | 2.491193 | 2.21E-85 | 1.34E-83 |
| PSD2 | ENSG00000146005 | 5.620713 | 2.490753 | 3.46E-28 | 2.08E-27 |
| LY86 | ENSG00000112799 | 5.619729 | 2.490501 | 1.91E-61 | 4.93E-60 |
| CABP2 | ENSG00000167791 | 5.612280 | 2.488587 | 1.54E-04 | 2.39E-04 |
| KIF20A | ENSG00000112984 | 5.609477 | 2.487866 | 5.32E-35 | 4.44E-34 |
| MROH9 | ENSG00000117501 | 5.608979 | 2.487738 | 2.21E-16 | 7.25E-16 |
| RNASE2 | ENSG00000169385 | 5.608002 | 2.487487 | 1.54E-30 | 1.04E-29 |
| BRDT | ENSG00000137948 | 5.606083 | 2.486993 | 2.59E-13 | 7.13E-13 |
| HOXC12 | ENSG00000123407 | 5.604733 | 2.486646 | 1.40E-16 | 4.65E-16 |
| SNX20 | ENSG00000167208 | 5.603847 | 2.486417 | 1.71E-48 | 2.68E-47 |
| SPACA3 | ENSG00000141316 | 5.595504 | 2.484268 | 1.39E-08 | 2.84E-08 |
| CPB1 | ENSG00000153002 | 5.592919 | 2.483601 | 4.72E-12 | 1.21E-11 |
| S1PR5 | ENSG00000180739 | 5.586572 | 2.481963 | 8.90E-55 | 1.80E-53 |
| CDK3 | ENSG00000250506 | 5.586317 | 2.481897 | 4.94E-33 | 3.75E-32 |
| LILRA4 | ENSG00000239961 | 5.585571 | 2.481705 | 3.72E-24 | 1.82E-23 |
| CORO1A | ENSG00000102879 | 5.585545 | 2.481698 | 5.94E-57 | 1.30E-55 |
| TENM1 | ENSG00000009694 | 5.580402 | 2.480369 | 1.23E-36 | 1.11E-35 |
| ATP2A1 | ENSG00000196296 | 5.577052 | 2.479503 | 3.96E-32 | 2.89E-31 |
| CIB4 | ENSG00000157884 | 5.569522 | 2.477554 | 1.56E-16 | 5.15E-16 |
| SLC11A1 | ENSG00000018280 | 5.564563 | 2.476268 | 1.00E-44 | 1.30E-43 |
| PTTG1 | ENSG00000164611 | 5.562526 | 2.475740 | 2.60E-43 | 3.19E-42 |
| SH2D5 | ENSG00000189410 | 5.561528 | 2.475481 | 6.40E-38 | 6.12E-37 |
| SLC2A14 | ENSG00000173262 | 5.557316 | 2.474388 | 4.95E-22 | 2.18E-21 |
| KIR2DL1 | ENSG00000125498 | 5.556975 | 2.474300 | 1.13E-33 | 8.90E-33 |
| GS1-393G12.13 | ENSG00000271698 | 5.555855 | 2.474009 | 1.41E-15 | 4.41E-15 |
| RBPJL | ENSG00000124232 | 5.551739 | 2.472940 | 3.68E-11 | 8.93E-11 |
| STAMBPL1 | ENSG00000138134 | 5.549811 | 2.472439 | 5.42E-88 | 3.61E-86 |
| ABCA12 | ENSG00000144452 | 5.546169 | 2.471492 | 6.40E-26 | 3.43E-25 |
| ADSSL1 | ENSG00000185100 | 5.541925 | 2.470387 | 2.84E-33 | 2.18E-32 |
| HAPLN3 | ENSG00000140511 | 5.539346 | 2.469716 | 1.58E-51 | 2.86E-50 |
| EFNA3 | ENSG00000143590 | 5.538047 | 2.469377 | 2.00E-58 | 4.58E-57 |
| CD80 | ENSG00000121594 | 5.537050 | 2.469118 | 4.10E-40 | 4.33E-39 |
| RIMBP3C | ENSG00000183246 | 5.534180 | 2.468370 | 1.88E-23 | 8.86E-23 |
| APLN | ENSG00000171388 | 5.528760 | 2.466956 | 4.27E-49 | 6.87E-48 |
| MAP4K1 | ENSG00000104814 | 5.524887 | 2.465945 | 2.14E-48 | 3.33E-47 |
| KIR2DL4 | ENSG00000189013 | 5.518277 | 2.464218 | 3.64E-35 | 3.06E-34 |
| KCNT1 | ENSG00000107147 | 5.517637 | 2.464051 | 9.00E-28 | 5.27E-27 |
| CPA6 | ENSG00000165078 | 5.516160 | 2.463664 | 8.04E-35 | 6.67E-34 |
| KRT33B | ENSG00000131738 | 5.513008 | 2.462840 | 1.98E-10 | 4.57E-10 |
| RNASE3 | ENSG00000169397 | 5.512374 | 2.462674 | 2.33E-23 | 1.09E-22 |
| CEP55 | ENSG00000138180 | 5.507696 | 2.461449 | 7.46E-38 | 7.09E-37 |
| DLL4 | ENSG00000128917 | 5.505582 | 2.460895 | 5.46E-54 | 1.07E-52 |
| TLX1 | ENSG00000107807 | 5.498750 | 2.459104 | 4.40E-08 | 8.72E-08 |
| TMPRSS6 | ENSG00000187045 | 5.494376 | 2.457956 | 2.19E-14 | 6.42E-14 |
| SAP25 | ENSG00000205307 | 5.490076 | 2.456826 | 5.94E-21 | 2.49E-20 |
| LYZ | ENSG00000090382 | 5.485969 | 2.455747 | 4.31E-31 | 2.98E-30 |
| PFKFB4 | ENSG00000114268 | 5.480735 | 2.454369 | 8.31E-68 | 2.81E-66 |
| PRR7 | ENSG00000131188 | 5.478741 | 2.453844 | 2.10E-39 | 2.15E-38 |
| TPBGL | ENSG00000261594 | 5.477881 | 2.453618 | 5.79E-22 | 2.54E-21 |
| CDHR4 | ENSG00000187492 | 5.477208 | 2.453441 | 1.61E-12 | 4.24E-12 |
| CCDC154 | ENSG00000197599 | 5.476985 | 2.453382 | 8.90E-24 | 4.27E-23 |
| TIFAB | ENSG00000255833 | 5.470774 | 2.451745 | 8.94E-25 | 4.53E-24 |
| HIST1H2AM | ENSG00000278677 | 5.467702 | 2.450935 | 7.64E-25 | 3.89E-24 |
| XCL1 | ENSG00000143184 | 5.462349 | 2.449522 | 1.46E-31 | 1.04E-30 |
| AKR1B15 | ENSG00000227471 | 5.461382 | 2.449266 | 8.66E-06 | 1.47E-05 |
| E2F2 | ENSG00000007968 | 5.458953 | 2.448624 | 8.02E-51 | 1.41E-49 |
| CTNNA2 | ENSG00000066032 | 5.456022 | 2.447849 | 1.82E-06 | 3.22E-06 |
| IL27 | ENSG00000197272 | 5.451790 | 2.446730 | 6.95E-33 | 5.26E-32 |
| CDH23 | ENSG00000107736 | 5.450811 | 2.446471 | 2.87E-29 | 1.82E-28 |
| GTSE1 | ENSG00000075218 | 5.447963 | 2.445717 | 3.38E-43 | 4.13E-42 |
| NPIPB11 | ENSG00000254206 | 5.447827 | 2.445681 | 2.88E-27 | 1.65E-26 |
| MS4A6E | ENSG00000166926 | 5.446827 | 2.445416 | 3.50E-15 | 1.08E-14 |
| PNMA2 | ENSG00000240694 | 5.440424 | 2.443719 | 4.20E-37 | 3.85E-36 |
| HIST1H2BF | ENSG00000277224 | 5.437277 | 2.442884 | 9.85E-14 | 2.79E-13 |
| GPR35 | ENSG00000178623 | 5.429627 | 2.440853 | 2.60E-23 | 1.22E-22 |
| APOC3 | ENSG00000110245 | 5.411456 | 2.436017 | 3.37E-04 | 5.07E-04 |
| SPINK7 | ENSG00000145879 | 5.409798 | 2.435575 | 4.67E-09 | 9.84E-09 |
| DSG3 | ENSG00000134757 | 5.409082 | 2.435384 | 1.27E-08 | 2.59E-08 |
| SIX1 | ENSG00000126778 | 5.408183 | 2.435144 | 2.79E-39 | 2.83E-38 |
| ALPPL2 | ENSG00000163286 | 5.403760 | 2.433963 | 7.01E-05 | 1.11E-04 |
| CCDC144A | ENSG00000170160 | 5.401026 | 2.433234 | 1.23E-10 | 2.87E-10 |
| GPR174 | ENSG00000147138 | 5.392961 | 2.431078 | 3.24E-26 | 1.76E-25 |
| P2RY1 | ENSG00000169860 | 5.390366 | 2.430383 | 6.03E-59 | 1.41E-57 |
| IL2 | ENSG00000109471 | 5.387123 | 2.429515 | 5.61E-19 | 2.11E-18 |
| APBB1IP | ENSG00000077420 | 5.385671 | 2.429126 | 2.57E-50 | 4.41E-49 |
| RNF175 | ENSG00000145428 | 5.382994 | 2.428409 | 4.39E-42 | 5.13E-41 |
| LY6G5B | ENSG00000240053 | 5.366608 | 2.424010 | 4.42E-35 | 3.71E-34 |
| LZTS1 | ENSG00000061337 | 5.364266 | 2.423381 | 2.58E-60 | 6.39E-59 |
| PPP2R2C | ENSG00000074211 | 5.364006 | 2.423311 | 1.07E-11 | 2.68E-11 |
| HLA-DQA2 | ENSG00000237541 | 5.362589 | 2.422930 | 2.63E-19 | 1.01E-18 |
| IFI30 | ENSG00000216490 | 5.356909 | 2.421401 | 3.52E-36 | 3.08E-35 |
| CGB7 | ENSG00000196337 | 5.353586 | 2.420506 | 1.65E-22 | 7.43E-22 |
| CD6 | ENSG00000013725 | 5.349159 | 2.419312 | 1.39E-48 | 2.18E-47 |
| WNT1 | ENSG00000125084 | 5.342801 | 2.417596 | 2.84E-22 | 1.26E-21 |
| FAM64A | ENSG00000129195 | 5.336312 | 2.415843 | 1.56E-37 | 1.45E-36 |
| TESPA1 | ENSG00000135426 | 5.332166 | 2.414722 | 3.79E-40 | 4.02E-39 |
| ISG20 | ENSG00000172183 | 5.327119 | 2.413355 | 1.21E-62 | 3.30E-61 |
| ITGB2 | ENSG00000160255 | 5.326136 | 2.413089 | 1.69E-60 | 4.21E-59 |
| KRT14 | ENSG00000186847 | 5.324566 | 2.412664 | 7.10E-10 | 1.58E-09 |
| MS4A7 | ENSG00000166927 | 5.322151 | 2.412009 | 1.98E-59 | 4.74E-58 |
| BGLAP | ENSG00000242252 | 5.320997 | 2.411697 | 1.92E-30 | 1.29E-29 |
| TPX2 | ENSG00000088325 | 5.320245 | 2.411493 | 3.06E-37 | 2.82E-36 |
| MAP7D2 | ENSG00000184368 | 5.317903 | 2.410857 | 7.78E-16 | 2.47E-15 |
| HMGA2 | ENSG00000149948 | 5.308084 | 2.408191 | 6.86E-07 | 1.25E-06 |
| FAM78A | ENSG00000126882 | 5.307992 | 2.408166 | 1.87E-88 | 1.28E-86 |
| SPI1 | ENSG00000066336 | 5.303191 | 2.406861 | 5.85E-68 | 2.00E-66 |
| IRS4 | ENSG00000133124 | 5.302876 | 2.406775 | 1.87E-03 | 2.64E-03 |
| SYT5 | ENSG00000129990 | 5.302285 | 2.406614 | 1.10E-14 | 3.29E-14 |
| RP11-793H13.10 | ENSG00000267281 | 5.301706 | 2.406457 | 6.98E-37 | 6.34E-36 |
| SPC24 | ENSG00000161888 | 5.299440 | 2.405840 | 2.18E-44 | 2.79E-43 |
| FOXG1 | ENSG00000176165 | 5.298155 | 2.405490 | 8.32E-09 | 1.72E-08 |
| APOA2 | ENSG00000158874 | 5.296806 | 2.405123 | 4.66E-05 | 7.49E-05 |
| AC005779.2 | ENSG00000267545 | 5.294881 | 2.404598 | 1.19E-20 | 4.90E-20 |
| STEAP1B | ENSG00000105889 | 5.279555 | 2.400416 | 2.18E-21 | 9.29E-21 |
| TSHR | ENSG00000165409 | 5.275317 | 2.399258 | 6.81E-35 | 5.66E-34 |
| MAGEB17 | ENSG00000182798 | 5.275134 | 2.399208 | 1.08E-14 | 3.24E-14 |
| AHSP | ENSG00000169877 | 5.272413 | 2.398463 | 2.53E-08 | 5.09E-08 |
| MBOAT4 | ENSG00000177669 | 5.261480 | 2.395469 | 1.43E-63 | 4.15E-62 |
| ATG16L2 | ENSG00000168010 | 5.259937 | 2.395046 | 2.42E-50 | 4.15E-49 |
| SPTA1 | ENSG00000163554 | 5.259524 | 2.394932 | 1.14E-19 | 4.45E-19 |
| BHLHA15 | ENSG00000180535 | 5.251652 | 2.392771 | 1.06E-24 | 5.36E-24 |
| CHI3L2 | ENSG00000064886 | 5.244075 | 2.390688 | 1.81E-19 | 6.99E-19 |
| C1QTNF6 | ENSG00000133466 | 5.240289 | 2.389647 | 1.34E-69 | 4.89E-68 |
| CDC45 | ENSG00000093009 | 5.236846 | 2.388698 | 1.61E-42 | 1.91E-41 |
| CLEC2B | ENSG00000110852 | 5.234155 | 2.387957 | 2.40E-54 | 4.78E-53 |
| DLK2 | ENSG00000171462 | 5.232545 | 2.387513 | 2.09E-34 | 1.70E-33 |
| KRT78 | ENSG00000170423 | 5.229212 | 2.386593 | 1.14E-08 | 2.34E-08 |
| AQP9 | ENSG00000103569 | 5.226261 | 2.385779 | 1.35E-13 | 3.80E-13 |
| HTR3E | ENSG00000186038 | 5.210030 | 2.381292 | 1.07E-06 | 1.93E-06 |
| FAM72C | ENSG00000263513 | 5.209412 | 2.381121 | 1.78E-28 | 1.08E-27 |
| CSTA | ENSG00000121552 | 5.205794 | 2.380118 | 8.80E-46 | 1.22E-44 |
| GP6 | ENSG00000088053 | 5.198014 | 2.377961 | 8.41E-27 | 4.71E-26 |
| PABPC1L | ENSG00000101104 | 5.197027 | 2.377687 | 3.17E-29 | 2.00E-28 |
| CDK18 | ENSG00000117266 | 5.196682 | 2.377591 | 2.62E-64 | 7.80E-63 |
| PIF1 | ENSG00000140451 | 5.195740 | 2.377329 | 3.72E-35 | 3.13E-34 |
| NKX2-8 | ENSG00000136327 | 5.189811 | 2.375682 | 9.07E-06 | 1.54E-05 |
| KLRD1 | ENSG00000134539 | 5.187525 | 2.375046 | 6.10E-63 | 1.70E-61 |
| IL26 | ENSG00000111536 | 5.179782 | 2.372891 | 5.86E-17 | 1.99E-16 |
| RP11-6L6.2 | ENSG00000250673 | 5.176347 | 2.371934 | 5.93E-21 | 2.48E-20 |
| CACNG6 | ENSG00000130433 | 5.174496 | 2.371418 | 1.39E-07 | 2.66E-07 |
| C10orf142 | ENSG00000277288 | 5.174023 | 2.371286 | 1.28E-15 | 4.03E-15 |
| GCKR | ENSG00000084734 | 5.167279 | 2.369405 | 6.29E-10 | 1.41E-09 |
| ABCC3 | ENSG00000108846 | 5.165337 | 2.368862 | 1.63E-45 | 2.22E-44 |
| ASPM | ENSG00000066279 | 5.163238 | 2.368276 | 3.11E-31 | 2.17E-30 |
| OR1K1 | ENSG00000165204 | 5.162271 | 2.368006 | 4.22E-09 | 8.92E-09 |
| CDT1 | ENSG00000167513 | 5.161317 | 2.367739 | 6.12E-51 | 1.08E-49 |
| FGL1 | ENSG00000104760 | 5.155589 | 2.366137 | 3.07E-05 | 4.99E-05 |
| TMEM249 | ENSG00000261587 | 5.154610 | 2.365863 | 6.06E-14 | 1.74E-13 |
| CACNA1F | ENSG00000102001 | 5.154300 | 2.365777 | 1.22E-26 | 6.78E-26 |
| C1orf162 | ENSG00000143110 | 5.152605 | 2.365302 | 1.36E-64 | 4.08E-63 |
| CRLF2 | ENSG00000205755 | 5.150816 | 2.364801 | 6.62E-21 | 2.76E-20 |
| RTBDN | ENSG00000132026 | 5.150091 | 2.364598 | 1.87E-08 | 3.79E-08 |
| PRKCDBP | ENSG00000170955 | 5.148013 | 2.364016 | 2.93E-55 | 6.02E-54 |
| APOBEC3G | ENSG00000239713 | 5.147166 | 2.363778 | 1.33E-66 | 4.30E-65 |
| KIF4B | ENSG00000226650 | 5.139718 | 2.361689 | 4.62E-20 | 1.85E-19 |
| MYH15 | ENSG00000144821 | 5.137997 | 2.361206 | 9.09E-33 | 6.84E-32 |
| F2RL3 | ENSG00000127533 | 5.132527 | 2.359669 | 5.55E-28 | 3.29E-27 |
| SLC6A1 | ENSG00000157103 | 5.130011 | 2.358962 | 8.48E-40 | 8.81E-39 |
| SAMD3 | ENSG00000164483 | 5.127543 | 2.358268 | 9.67E-52 | 1.77E-50 |
| SULT1A3 | ENSG00000261052 | 5.127036 | 2.358125 | 1.77E-19 | 6.84E-19 |
| TRAT1 | ENSG00000163519 | 5.126954 | 2.358102 | 5.39E-29 | 3.36E-28 |
| SEZ6L2 | ENSG00000174938 | 5.123006 | 2.356991 | 5.12E-37 | 4.67E-36 |
| HMOX1 | ENSG00000100292 | 5.121531 | 2.356575 | 2.01E-46 | 2.90E-45 |
| TMEM196 | ENSG00000173452 | 5.119850 | 2.356101 | 4.49E-07 | 8.31E-07 |
| STAC3 | ENSG00000185482 | 5.118478 | 2.355715 | 2.06E-77 | 9.84E-76 |
| CLEC7A | ENSG00000172243 | 5.104044 | 2.351641 | 2.12E-50 | 3.66E-49 |
| CCDC155 | ENSG00000161609 | 5.103472 | 2.351479 | 2.74E-11 | 6.69E-11 |
| PIWIL3 | ENSG00000184571 | 5.101548 | 2.350935 | 3.30E-11 | 8.03E-11 |
| HLA-DQB1 | ENSG00000179344 | 5.093255 | 2.348588 | 5.13E-46 | 7.20E-45 |
| MOGAT3 | ENSG00000106384 | 5.084523 | 2.346112 | 4.37E-15 | 1.33E-14 |
| TUBA3E | ENSG00000152086 | 5.080423 | 2.344949 | 6.75E-06 | 1.15E-05 |
| TBX20 | ENSG00000164532 | 5.077479 | 2.344112 | 1.27E-08 | 2.61E-08 |
| RNASE10 | ENSG00000182545 | 5.076707 | 2.343893 | 2.02E-30 | 1.35E-29 |
| LRTM1 | ENSG00000144771 | 5.076691 | 2.343888 | 1.40E-07 | 2.69E-07 |
| EBI3 | ENSG00000105246 | 5.073829 | 2.343075 | 9.72E-49 | 1.54E-47 |
| FBXO39 | ENSG00000177294 | 5.073276 | 2.342918 | 1.08E-31 | 7.76E-31 |
| MCM10 | ENSG00000065328 | 5.069212 | 2.341761 | 1.73E-41 | 1.96E-40 |
| PGA5 | ENSG00000256713 | 5.069011 | 2.341704 | 1.80E-14 | 5.29E-14 |
| CCDC177 | ENSG00000267909 | 5.068165 | 2.341463 | 4.12E-10 | 9.33E-10 |
| CD244 | ENSG00000122223 | 5.063260 | 2.340066 | 2.09E-50 | 3.61E-49 |
| MLN | ENSG00000096395 | 5.060004 | 2.339139 | 5.35E-12 | 1.37E-11 |
| SERPINE1 | ENSG00000106366 | 5.058738 | 2.338777 | 9.20E-20 | 3.62E-19 |
| TBC1D3L | ENSG00000274512 | 5.057769 | 2.338501 | 4.08E-18 | 1.47E-17 |
| DEF6 | ENSG00000023892 | 5.054324 | 2.337518 | 1.55E-56 | 3.36E-55 |
| VAV1 | ENSG00000141968 | 5.052443 | 2.336981 | 2.04E-58 | 4.68E-57 |
| RDH16 | ENSG00000139547 | 5.051029 | 2.336577 | 1.30E-15 | 4.07E-15 |
| CABP5 | ENSG00000105507 | 5.048125 | 2.335748 | 1.04E-08 | 2.14E-08 |
| KIF4A | ENSG00000090889 | 5.044777 | 2.334790 | 7.95E-43 | 9.57E-42 |
| MAGEA12 | ENSG00000213401 | 5.043805 | 2.334512 | 2.67E-06 | 4.67E-06 |
| MSLN | ENSG00000102854 | 5.042332 | 2.334091 | 1.15E-07 | 2.22E-07 |
| ADA | ENSG00000196839 | 5.040989 | 2.333707 | 1.14E-44 | 1.48E-43 |
| GNGT1 | ENSG00000127928 | 5.037271 | 2.332642 | 2.20E-14 | 6.44E-14 |
| HIST1H2BL | ENSG00000185130 | 5.036865 | 2.332526 | 3.83E-23 | 1.78E-22 |
| MKI67 | ENSG00000148773 | 5.036849 | 2.332521 | 1.57E-34 | 1.28E-33 |
| LILRB2 | ENSG00000131042 | 5.032031 | 2.331141 | 1.03E-67 | 3.48E-66 |
| MUCL1 | ENSG00000172551 | 5.030257 | 2.330632 | 1.27E-04 | 1.99E-04 |
| HES4 | ENSG00000188290 | 5.030249 | 2.330630 | 6.22E-44 | 7.82E-43 |
| ACKR3 | ENSG00000144476 | 5.029168 | 2.330320 | 9.52E-51 | 1.67E-49 |
| SIGLEC9 | ENSG00000129450 | 5.027207 | 2.329757 | 7.40E-60 | 1.80E-58 |
| NCF1 | ENSG00000158517 | 5.026494 | 2.329552 | 5.86E-47 | 8.63E-46 |
| KHDC1L | ENSG00000256980 | 5.018613 | 2.327289 | 2.48E-11 | 6.09E-11 |
| BIRC3 | ENSG00000023445 | 5.018185 | 2.327166 | 1.54E-41 | 1.75E-40 |
| CORO6 | ENSG00000167549 | 5.016808 | 2.326770 | 1.35E-19 | 5.27E-19 |
| IL2RA | ENSG00000134460 | 5.014906 | 2.326223 | 2.26E-23 | 1.06E-22 |
| KRT6C | ENSG00000170465 | 5.012445 | 2.325515 | 9.41E-05 | 1.48E-04 |
| RIMKLA | ENSG00000177181 | 5.010661 | 2.325001 | 1.15E-42 | 1.37E-41 |
| ARID3C | ENSG00000205143 | 4.999142 | 2.321680 | 8.47E-18 | 3.01E-17 |
| CCND1 | ENSG00000110092 | 4.998842 | 2.321594 | 1.94E-61 | 5.02E-60 |
| MZB1 | ENSG00000170476 | 4.991700 | 2.319531 | 3.23E-11 | 7.87E-11 |
| DPYSL4 | ENSG00000151640 | 4.990107 | 2.319071 | 7.19E-13 | 1.93E-12 |
| MATK | ENSG00000007264 | 4.987934 | 2.318442 | 6.68E-50 | 1.12E-48 |
| C6orf141 | ENSG00000197261 | 4.986319 | 2.317975 | 1.52E-13 | 4.25E-13 |
| NCKAP1L | ENSG00000123338 | 4.980425 | 2.316269 | 4.58E-51 | 8.12E-50 |
| NLRP7 | ENSG00000167634 | 4.979593 | 2.316028 | 7.05E-16 | 2.25E-15 |
| SMC1B | ENSG00000077935 | 4.973630 | 2.314299 | 3.44E-26 | 1.86E-25 |
| NT5DC3 | ENSG00000111696 | 4.969670 | 2.313150 | 1.45E-45 | 1.98E-44 |
| KIR3DL2 | ENSG00000240403 | 4.969498 | 2.313100 | 6.28E-26 | 3.37E-25 |
| DPEP2 | ENSG00000167261 | 4.966352 | 2.312186 | 2.34E-71 | 8.93E-70 |
| AMER3 | ENSG00000178171 | 4.958474 | 2.309896 | 1.14E-05 | 1.92E-05 |
| EDA2R | ENSG00000131080 | 4.956256 | 2.309251 | 5.97E-85 | 3.54E-83 |
| C2 | ENSG00000166278 | 4.953733 | 2.308516 | 2.26E-22 | 1.01E-21 |
| LSP1 | ENSG00000130592 | 4.950904 | 2.307692 | 1.40E-51 | 2.54E-50 |
| TDO2 | ENSG00000151790 | 4.949552 | 2.307298 | 4.68E-15 | 1.43E-14 |
| KIAA0101 | ENSG00000166803 | 4.943338 | 2.305486 | 2.52E-49 | 4.11E-48 |
| LRAT | ENSG00000121207 | 4.941315 | 2.304895 | 7.14E-26 | 3.82E-25 |
| ANKK1 | ENSG00000170209 | 4.937804 | 2.303870 | 1.00E-30 | 6.82E-30 |
| CLECL1 | ENSG00000184293 | 4.936856 | 2.303593 | 7.20E-36 | 6.23E-35 |
| SULT1C3 | ENSG00000196228 | 4.936431 | 2.303468 | 4.62E-05 | 7.44E-05 |
| FMNL1 | ENSG00000184922 | 4.928609 | 2.301180 | 2.65E-68 | 9.23E-67 |
| NEUROD2 | ENSG00000171532 | 4.917732 | 2.297993 | 3.71E-12 | 9.57E-12 |
| PRDM1 | ENSG00000057657 | 4.916048 | 2.297499 | 6.36E-82 | 3.53E-80 |
| ADAM29 | ENSG00000168594 | 4.914639 | 2.297085 | 6.42E-16 | 2.05E-15 |
| FBLN7 | ENSG00000144152 | 4.914221 | 2.296963 | 1.51E-30 | 1.02E-29 |
| HSD3B7 | ENSG00000099377 | 4.912865 | 2.296565 | 1.72E-43 | 2.12E-42 |
| HFE2 | ENSG00000168509 | 4.912071 | 2.296331 | 5.48E-08 | 1.08E-07 |
| WNT10B | ENSG00000169884 | 4.911542 | 2.296176 | 2.28E-19 | 8.78E-19 |
| TNFAIP8L2 | ENSG00000163154 | 4.904429 | 2.294085 | 2.47E-58 | 5.64E-57 |
| SLX1B | ENSG00000181625 | 4.903272 | 2.293745 | 7.66E-15 | 2.31E-14 |
| NLRC5 | ENSG00000140853 | 4.899940 | 2.292764 | 1.43E-74 | 6.05E-73 |
| NUTM1 | ENSG00000184507 | 4.898146 | 2.292236 | 1.10E-09 | 2.41E-09 |
| BEST2 | ENSG00000039987 | 4.895117 | 2.291343 | 6.89E-11 | 1.64E-10 |
| KLK13 | ENSG00000167759 | 4.891851 | 2.290380 | 5.28E-10 | 1.19E-09 |
| EHD2 | ENSG00000024422 | 4.888638 | 2.289433 | 8.15E-77 | 3.77E-75 |
| ODF3 | ENSG00000177947 | 4.886135 | 2.288694 | 8.51E-13 | 2.28E-12 |
| LST1 | ENSG00000204482 | 4.880791 | 2.287115 | 6.72E-59 | 1.57E-57 |
| ADAM8 | ENSG00000151651 | 4.880023 | 2.286888 | 1.25E-33 | 9.78E-33 |
| ASIC4 | ENSG00000072182 | 4.878832 | 2.286536 | 3.97E-17 | 1.37E-16 |
| MTCL1 | ENSG00000168502 | 4.872715 | 2.284726 | 2.17E-53 | 4.17E-52 |
| DERL3 | ENSG00000099958 | 4.866369 | 2.282846 | 4.99E-24 | 2.43E-23 |
| ALDOC | ENSG00000109107 | 4.862092 | 2.281577 | 1.19E-35 | 1.02E-34 |
| CDON | ENSG00000064309 | 4.859951 | 2.280942 | 2.04E-45 | 2.77E-44 |
| DAPL1 | ENSG00000163331 | 4.858456 | 2.280498 | 7.15E-06 | 1.22E-05 |
| CD1D | ENSG00000158473 | 4.857986 | 2.280358 | 2.67E-77 | 1.26E-75 |
| PLEK | ENSG00000115956 | 4.857468 | 2.280205 | 3.01E-44 | 3.84E-43 |
| KLK3 | ENSG00000142515 | 4.856764 | 2.279996 | 9.47E-05 | 1.49E-04 |
| GPR15 | ENSG00000154165 | 4.855161 | 2.279519 | 3.47E-11 | 8.43E-11 |
| GPR19 | ENSG00000183150 | 4.852186 | 2.278635 | 8.97E-45 | 1.17E-43 |
| KLRC4-KLRK1 | ENSG00000255819 | 4.851284 | 2.278367 | 1.80E-23 | 8.49E-23 |
| LRRC39 | ENSG00000122477 | 4.850143 | 2.278027 | 5.06E-23 | 2.33E-22 |
| OR51E2 | ENSG00000167332 | 4.848217 | 2.277454 | 8.26E-28 | 4.85E-27 |
| CD300C | ENSG00000167850 | 4.847193 | 2.277150 | 1.74E-47 | 2.63E-46 |
| CENPM | ENSG00000100162 | 4.846467 | 2.276933 | 1.81E-46 | 2.61E-45 |
| C10orf10 | ENSG00000165507 | 4.846139 | 2.276836 | 2.20E-56 | 4.74E-55 |
| MMP25 | ENSG00000008516 | 4.843820 | 2.276145 | 8.65E-43 | 1.04E-41 |
| LRRC25 | ENSG00000175489 | 4.843327 | 2.275999 | 9.15E-41 | 9.94E-40 |
| TREM1 | ENSG00000124731 | 4.841254 | 2.275381 | 5.89E-19 | 2.22E-18 |
| UNC13D | ENSG00000092929 | 4.839376 | 2.274821 | 1.86E-54 | 3.72E-53 |
| OTOG | ENSG00000188162 | 4.833769 | 2.273149 | 5.83E-22 | 2.56E-21 |
| PTGDR | ENSG00000168229 | 4.825399 | 2.270648 | 8.14E-45 | 1.07E-43 |
| ENTHD1 | ENSG00000176177 | 4.825354 | 2.270635 | 7.11E-14 | 2.03E-13 |
| GPR141 | ENSG00000187037 | 4.823055 | 2.269947 | 2.14E-36 | 1.90E-35 |
| RRM2 | ENSG00000171848 | 4.819552 | 2.268899 | 2.29E-32 | 1.69E-31 |
| ADAMTS10 | ENSG00000142303 | 4.815238 | 2.267607 | 2.07E-39 | 2.12E-38 |
| PF4V1 | ENSG00000109272 | 4.801970 | 2.263626 | 6.30E-16 | 2.02E-15 |
| IL10RA | ENSG00000110324 | 4.801617 | 2.263520 | 1.21E-52 | 2.28E-51 |
| PSMB9 | ENSG00000240065 | 4.798865 | 2.262693 | 6.05E-73 | 2.44E-71 |
| GPR150 | ENSG00000178015 | 4.794154 | 2.261276 | 1.86E-23 | 8.78E-23 |
| KLRC4 | ENSG00000183542 | 4.793373 | 2.261041 | 1.37E-20 | 5.62E-20 |
| PRSS37 | ENSG00000165076 | 4.791942 | 2.260611 | 1.93E-21 | 8.24E-21 |
| MSH5 | ENSG00000204410 | 4.791309 | 2.260420 | 5.18E-34 | 4.14E-33 |
| NPIPB3 | ENSG00000169246 | 4.790042 | 2.260038 | 1.71E-34 | 1.39E-33 |
| E2F1 | ENSG00000101412 | 4.787613 | 2.259306 | 6.02E-59 | 1.41E-57 |
| CAV1 | ENSG00000105974 | 4.787185 | 2.259178 | 2.24E-63 | 6.45E-62 |
| MLC1 | ENSG00000100427 | 4.785830 | 2.258769 | 7.17E-44 | 9.00E-43 |
| FCRL5 | ENSG00000143297 | 4.783607 | 2.258099 | 2.44E-11 | 5.98E-11 |
| MCEMP1 | ENSG00000183019 | 4.781331 | 2.257412 | 1.22E-18 | 4.50E-18 |
| FFAR3 | ENSG00000185897 | 4.779635 | 2.256900 | 2.17E-13 | 6.03E-13 |
| LTB4R2 | ENSG00000213906 | 4.776264 | 2.255883 | 2.10E-52 | 3.91E-51 |
| BHMG1 | ENSG00000237452 | 4.775618 | 2.255688 | 6.26E-08 | 1.23E-07 |
| KRT6A | ENSG00000205420 | 4.771269 | 2.254373 | 6.06E-05 | 9.68E-05 |
| GUCY2F | ENSG00000101890 | 4.770989 | 2.254288 | 2.11E-09 | 4.55E-09 |
| PLK2 | ENSG00000145632 | 4.768590 | 2.253563 | 6.85E-73 | 2.76E-71 |
| SLC2A7 | ENSG00000197241 | 4.762984 | 2.251866 | 9.34E-23 | 4.25E-22 |
| DOCK2 | ENSG00000134516 | 4.762780 | 2.251804 | 1.58E-44 | 2.04E-43 |
| ZNF831 | ENSG00000124203 | 4.760976 | 2.251257 | 1.74E-32 | 1.30E-31 |
| POLQ | ENSG00000051341 | 4.758785 | 2.250593 | 2.27E-36 | 2.01E-35 |
| TM4SF19-TCTEX1D2 | ENSG00000273331 | 4.756816 | 2.249996 | 9.08E-13 | 2.42E-12 |
| ANK1 | ENSG00000029534 | 4.756701 | 2.249961 | 8.65E-22 | 3.76E-21 |
| GPRIN1 | ENSG00000169258 | 4.751382 | 2.248347 | 3.86E-45 | 5.15E-44 |
| MS4A6A | ENSG00000110077 | 4.750180 | 2.247982 | 3.11E-51 | 5.56E-50 |
| XKR7 | ENSG00000260903 | 4.745789 | 2.246648 | 4.24E-08 | 8.41E-08 |
| E2F7 | ENSG00000165891 | 4.745115 | 2.246443 | 3.52E-26 | 1.91E-25 |
| SAGE1 | ENSG00000181433 | 4.742958 | 2.245787 | 1.05E-08 | 2.17E-08 |
| PLD4 | ENSG00000166428 | 4.742819 | 2.245745 | 3.51E-35 | 2.96E-34 |
| CHSY3 | ENSG00000198108 | 4.741573 | 2.245366 | 6.88E-52 | 1.26E-50 |
| TMIGD2 | ENSG00000167664 | 4.738601 | 2.244461 | 1.80E-33 | 1.40E-32 |
| TRPM8 | ENSG00000144481 | 4.737490 | 2.244123 | 7.89E-12 | 2.00E-11 |
| MAGEA11 | ENSG00000185247 | 4.733413 | 2.242881 | 6.20E-03 | 8.34E-03 |
| FAM111B | ENSG00000189057 | 4.732912 | 2.242728 | 2.44E-45 | 3.29E-44 |
| RP11-514O12.4 | ENSG00000249141 | 4.732347 | 2.242556 | 1.01E-36 | 9.13E-36 |
| CAPN11 | ENSG00000137225 | 4.731709 | 2.242361 | 9.95E-22 | 4.31E-21 |
| S100Z | ENSG00000171643 | 4.729893 | 2.241808 | 1.59E-41 | 1.81E-40 |
| TBC1D3B | ENSG00000274808 | 4.724166 | 2.240060 | 4.15E-20 | 1.66E-19 |
| CD200R1 | ENSG00000163606 | 4.723060 | 2.239722 | 3.66E-29 | 2.30E-28 |
| SLC45A2 | ENSG00000164175 | 4.717792 | 2.238112 | 3.71E-16 | 1.20E-15 |
| SOX21 | ENSG00000125285 | 4.716162 | 2.237613 | 2.59E-11 | 6.34E-11 |
| CERS1 | ENSG00000223802 | 4.710508 | 2.235883 | 1.33E-12 | 3.51E-12 |
| LCP2 | ENSG00000043462 | 4.708650 | 2.235313 | 6.61E-81 | 3.52E-79 |
| KCNMA1 | ENSG00000156113 | 4.706336 | 2.234604 | 7.78E-31 | 5.31E-30 |
| KIR3DL1 | ENSG00000167633 | 4.704839 | 2.234145 | 3.80E-28 | 2.27E-27 |
| RHOH | ENSG00000168421 | 4.703513 | 2.233739 | 1.86E-38 | 1.83E-37 |
| AZU1 | ENSG00000172232 | 4.702938 | 2.233562 | 6.41E-15 | 1.94E-14 |
| HTR2C | ENSG00000147246 | 4.701602 | 2.233152 | 1.97E-05 | 3.25E-05 |
| KCNV2 | ENSG00000168263 | 4.700458 | 2.232801 | 4.28E-31 | 2.96E-30 |
| NCAPG | ENSG00000109805 | 4.700237 | 2.232733 | 3.30E-36 | 2.90E-35 |
| A3GALT2 | ENSG00000184389 | 4.699912 | 2.232634 | 1.79E-20 | 7.33E-20 |
| MDS2 | ENSG00000197880 | 4.699339 | 2.232458 | 2.11E-38 | 2.06E-37 |
| BUB1 | ENSG00000169679 | 4.694551 | 2.230987 | 5.98E-33 | 4.54E-32 |
| KRT73 | ENSG00000186049 | 4.689718 | 2.229501 | 6.79E-15 | 2.05E-14 |
| SKA1 | ENSG00000154839 | 4.689317 | 2.229378 | 1.48E-34 | 1.21E-33 |
| WAS | ENSG00000015285 | 4.689042 | 2.229293 | 8.92E-57 | 1.95E-55 |
| LGALS9 | ENSG00000168961 | 4.687761 | 2.228899 | 5.02E-79 | 2.52E-77 |
| UBAP1L | ENSG00000246922 | 4.687301 | 2.228757 | 1.93E-37 | 1.80E-36 |
| STMN3 | ENSG00000197457 | 4.682925 | 2.227410 | 9.56E-38 | 9.04E-37 |
| DPEP3 | ENSG00000141096 | 4.682115 | 2.227160 | 4.18E-24 | 2.04E-23 |
| CACNG8 | ENSG00000142408 | 4.677538 | 2.225749 | 1.24E-30 | 8.37E-30 |
| ASF1B | ENSG00000105011 | 4.672989 | 2.224346 | 1.68E-50 | 2.92E-49 |
| DDIT4 | ENSG00000168209 | 4.671024 | 2.223739 | 4.98E-54 | 9.78E-53 |
| NFKBID | ENSG00000167604 | 4.670956 | 2.223718 | 3.33E-36 | 2.92E-35 |
| PRSS57 | ENSG00000185198 | 4.659150 | 2.220067 | 2.18E-17 | 7.60E-17 |
| GRIA1 | ENSG00000155511 | 4.656781 | 2.219333 | 1.22E-10 | 2.86E-10 |
| LGALS1 | ENSG00000100097 | 4.656527 | 2.219254 | 4.27E-51 | 7.60E-50 |
| CD36 | ENSG00000135218 | 4.654146 | 2.218517 | 1.55E-29 | 1.00E-28 |
| PLEKHG4 | ENSG00000196155 | 4.653976 | 2.218464 | 2.90E-36 | 2.55E-35 |
| MMP17 | ENSG00000198598 | 4.651843 | 2.217802 | 9.85E-17 | 3.30E-16 |
| TRIM74 | ENSG00000155428 | 4.648055 | 2.216627 | 2.30E-19 | 8.86E-19 |
| DCLK1 | ENSG00000133083 | 4.647495 | 2.216453 | 8.43E-31 | 5.75E-30 |
| ARHGAP33 | ENSG00000004777 | 4.645340 | 2.215784 | 1.10E-36 | 9.90E-36 |
| TMPRSS11D | ENSG00000153802 | 4.638553 | 2.213675 | 2.74E-12 | 7.13E-12 |
| PPP1R14D | ENSG00000166143 | 4.634626 | 2.212453 | 4.78E-11 | 1.15E-10 |
| YJEFN3 | ENSG00000250067 | 4.634092 | 2.212287 | 8.86E-24 | 4.25E-23 |
| FAM193B | ENSG00000146067 | 4.632389 | 2.211756 | 4.52E-44 | 5.71E-43 |
| HLA-DQA1 | ENSG00000196735 | 4.630795 | 2.211260 | 3.66E-42 | 4.29E-41 |
| LA16c-431H6.6 | ENSG00000261732 | 4.626519 | 2.209927 | 9.32E-25 | 4.72E-24 |
| DTL | ENSG00000143476 | 4.626485 | 2.209916 | 1.18E-47 | 1.80E-46 |
| VASH1 | ENSG00000071246 | 4.623874 | 2.209102 | 1.24E-74 | 5.27E-73 |
| RASSF2 | ENSG00000101265 | 4.622850 | 2.208782 | 2.70E-62 | 7.32E-61 |
| TNR | ENSG00000116147 | 4.619586 | 2.207763 | 2.87E-10 | 6.57E-10 |
| NGF | ENSG00000134259 | 4.619039 | 2.207593 | 2.49E-35 | 2.11E-34 |
| THEMIS | ENSG00000172673 | 4.615998 | 2.206643 | 1.63E-27 | 9.44E-27 |
| SLC29A4 | ENSG00000164638 | 4.612797 | 2.205642 | 8.88E-38 | 8.42E-37 |
| MAPK8IP3 | ENSG00000138834 | 4.612027 | 2.205401 | 1.05E-42 | 1.26E-41 |
| RP11-385D13.1 | ENSG00000251537 | 4.610073 | 2.204790 | 2.17E-17 | 7.58E-17 |
| PHKA2 | ENSG00000044446 | 4.607312 | 2.203925 | 1.56E-81 | 8.57E-80 |
| CRACR2A | ENSG00000130038 | 4.599497 | 2.201476 | 1.33E-53 | 2.58E-52 |
| HSPB8 | ENSG00000152137 | 4.594632 | 2.199949 | 8.28E-53 | 1.56E-51 |
| GPR84 | ENSG00000139572 | 4.593619 | 2.199631 | 6.44E-27 | 3.63E-26 |
| BTK | ENSG00000010671 | 4.592779 | 2.199367 | 6.47E-56 | 1.37E-54 |
| TNFSF11 | ENSG00000120659 | 4.589707 | 2.198402 | 7.65E-14 | 2.18E-13 |
| OTP | ENSG00000171540 | 4.588792 | 2.198114 | 3.27E-11 | 7.96E-11 |
| PLCB2 | ENSG00000137841 | 4.582195 | 2.196039 | 8.41E-50 | 1.41E-48 |
| KLRC3 | ENSG00000205810 | 4.579967 | 2.195337 | 3.22E-18 | 1.17E-17 |
| APOL1 | ENSG00000100342 | 4.576543 | 2.194258 | 3.43E-24 | 1.68E-23 |
| SYNGR4 | ENSG00000105467 | 4.576312 | 2.194185 | 1.41E-17 | 4.95E-17 |
| AHSA2 | ENSG00000173209 | 4.575780 | 2.194018 | 1.35E-38 | 1.33E-37 |
| COL21A1 | ENSG00000124749 | 4.571865 | 2.192783 | 3.03E-25 | 1.58E-24 |
| CYP3A5 | ENSG00000106258 | 4.570935 | 2.192489 | 5.28E-24 | 2.56E-23 |
| HLA-DOB | ENSG00000241106 | 4.568206 | 2.191628 | 2.52E-26 | 1.37E-25 |
| MAGEA3 | ENSG00000221867 | 4.566382 | 2.191052 | 3.31E-03 | 4.57E-03 |
| CTC-479C5.12 | ENSG00000261884 | 4.563441 | 2.190122 | 1.84E-43 | 2.27E-42 |
| MAP3K7CL | ENSG00000156265 | 4.559328 | 2.188821 | 2.05E-40 | 2.20E-39 |
| TMSB10 | ENSG00000034510 | 4.557779 | 2.188331 | 7.03E-66 | 2.22E-64 |
| CALML6 | ENSG00000169885 | 4.555887 | 2.187732 | 1.88E-15 | 5.84E-15 |
| FPR3 | ENSG00000187474 | 4.555333 | 2.187556 | 3.10E-39 | 3.13E-38 |
| KCNK3 | ENSG00000171303 | 4.554076 | 2.187158 | 4.51E-19 | 1.71E-18 |
| ADAMTSL4 | ENSG00000143382 | 4.550762 | 2.186108 | 3.35E-39 | 3.38E-38 |
| ZNF705A | ENSG00000196946 | 4.547862 | 2.185189 | 3.90E-13 | 1.07E-12 |
| PLXNB3 | ENSG00000198753 | 4.547694 | 2.185135 | 3.65E-20 | 1.47E-19 |
| LPAR5 | ENSG00000184574 | 4.547417 | 2.185047 | 4.76E-50 | 8.07E-49 |
| GZMM | ENSG00000197540 | 4.547391 | 2.185039 | 1.10E-40 | 1.18E-39 |
| ADAM11 | ENSG00000073670 | 4.546828 | 2.184860 | 1.46E-33 | 1.14E-32 |
| SLFN13 | ENSG00000154760 | 4.544723 | 2.184192 | 1.19E-77 | 5.80E-76 |
| AOAH | ENSG00000136250 | 4.542860 | 2.183601 | 1.33E-44 | 1.72E-43 |
| TNFRSF14 | ENSG00000157873 | 4.542543 | 2.183500 | 5.37E-70 | 1.97E-68 |
| IL4I1 | ENSG00000104951 | 4.541653 | 2.183218 | 5.25E-35 | 4.38E-34 |
| AGER | ENSG00000204305 | 4.540956 | 2.182996 | 2.00E-37 | 1.86E-36 |
| CD37 | ENSG00000104894 | 4.540377 | 2.182812 | 4.79E-57 | 1.05E-55 |
| AL356289.1 | ENSG00000279096 | 4.538039 | 2.182069 | 8.15E-12 | 2.06E-11 |
| EME1 | ENSG00000154920 | 4.532736 | 2.180382 | 7.47E-43 | 9.00E-42 |
| GPR31 | ENSG00000120436 | 4.530516 | 2.179675 | 5.96E-16 | 1.91E-15 |
| NUSAP1 | ENSG00000137804 | 4.529508 | 2.179354 | 3.85E-68 | 1.32E-66 |
| PLK1 | ENSG00000166851 | 4.529508 | 2.179354 | 1.48E-31 | 1.05E-30 |
| TIMP1 | ENSG00000102265 | 4.526538 | 2.178408 | 1.18E-34 | 9.68E-34 |
| GDF5OS | ENSG00000204183 | 4.525179 | 2.177975 | 3.21E-08 | 6.43E-08 |
| LINC00176 | ENSG00000196421 | 4.524273 | 2.177686 | 2.85E-42 | 3.36E-41 |
| PCDHGC5 | ENSG00000240764 | 4.523305 | 2.177377 | 1.23E-28 | 7.56E-28 |
| LAT2 | ENSG00000086730 | 4.519976 | 2.176315 | 2.52E-68 | 8.81E-67 |
| KIF21B | ENSG00000116852 | 4.517646 | 2.175571 | 1.12E-54 | 2.26E-53 |
| C9orf172 | ENSG00000232434 | 4.514029 | 2.174416 | 1.29E-51 | 2.35E-50 |
| EIF4EBP1 | ENSG00000187840 | 4.510459 | 2.173274 | 2.77E-46 | 3.97E-45 |
| COL5A1 | ENSG00000130635 | 4.510250 | 2.173207 | 3.62E-23 | 1.68E-22 |
| SKA3 | ENSG00000165480 | 4.502000 | 2.170566 | 8.65E-45 | 1.13E-43 |
| TSGA10IP | ENSG00000175513 | 4.499342 | 2.169714 | 3.48E-19 | 1.33E-18 |
| CHRNG | ENSG00000196811 | 4.496652 | 2.168851 | 3.88E-14 | 1.12E-13 |
| BTNL9 | ENSG00000165810 | 4.493674 | 2.167896 | 9.17E-28 | 5.37E-27 |
| PIEZO2 | ENSG00000154864 | 4.493030 | 2.167689 | 2.79E-40 | 2.98E-39 |
| PPP1R3G | ENSG00000219607 | 4.491461 | 2.167185 | 1.87E-61 | 4.84E-60 |
| AC104581.1 | ENSG00000281523 | 4.490236 | 2.166791 | 1.48E-08 | 3.02E-08 |
| ANKRD62 | ENSG00000181626 | 4.488845 | 2.166344 | 5.32E-11 | 1.27E-10 |
| ANLN | ENSG00000011426 | 4.488688 | 2.166294 | 1.15E-26 | 6.41E-26 |
| FAM92B | ENSG00000153789 | 4.487470 | 2.165902 | 1.29E-09 | 2.82E-09 |
| KCNIP2 | ENSG00000120049 | 4.486828 | 2.165696 | 1.88E-32 | 1.40E-31 |
| SIRPB2 | ENSG00000196209 | 4.486056 | 2.165448 | 3.74E-51 | 6.67E-50 |
| CDCA7 | ENSG00000144354 | 4.483764 | 2.164710 | 5.25E-29 | 3.27E-28 |
| CD52 | ENSG00000169442 | 4.483718 | 2.164696 | 4.31E-39 | 4.33E-38 |
| ARHGAP36 | ENSG00000147256 | 4.476153 | 2.162259 | 5.84E-06 | 1.00E-05 |
| SPN | ENSG00000197471 | 4.475365 | 2.162005 | 1.13E-42 | 1.35E-41 |
| CT55 | ENSG00000169551 | 4.473773 | 2.161492 | 5.42E-11 | 1.30E-10 |
| EIF4A1 | ENSG00000161960 | 4.468574 | 2.159815 | 2.91E-38 | 2.83E-37 |
| TTR | ENSG00000118271 | 4.465604 | 2.158855 | 2.23E-04 | 3.41E-04 |
| C8G | ENSG00000176919 | 4.465011 | 2.158664 | 3.65E-15 | 1.12E-14 |
| NFAM1 | ENSG00000235568 | 4.464678 | 2.158556 | 1.97E-63 | 5.71E-62 |
| C3orf36 | ENSG00000221972 | 4.461917 | 2.157664 | 1.05E-26 | 5.86E-26 |
| CD86 | ENSG00000114013 | 4.460306 | 2.157143 | 5.21E-46 | 7.30E-45 |
| PGA4 | ENSG00000229183 | 4.460006 | 2.157045 | 1.89E-07 | 3.59E-07 |
| SIGLEC7 | ENSG00000168995 | 4.458665 | 2.156612 | 2.09E-36 | 1.85E-35 |
| VIP | ENSG00000146469 | 4.457530 | 2.156244 | 7.33E-21 | 3.05E-20 |
| FERMT3 | ENSG00000149781 | 4.455001 | 2.155426 | 1.20E-60 | 3.00E-59 |
| DGKD | ENSG00000077044 | 4.453927 | 2.155078 | 1.72E-81 | 9.37E-80 |
| GRM8 | ENSG00000179603 | 4.448484 | 2.153314 | 1.00E-20 | 4.14E-20 |
| CBLN1 | ENSG00000102924 | 4.443928 | 2.151835 | 2.87E-12 | 7.44E-12 |
| PMFBP1 | ENSG00000118557 | 4.443204 | 2.151600 | 7.90E-32 | 5.71E-31 |
| GLRA1 | ENSG00000145888 | 4.443057 | 2.151553 | 6.94E-11 | 1.65E-10 |
| LCT | ENSG00000115850 | 4.442249 | 2.151290 | 1.20E-11 | 3.01E-11 |
| VGLL2 | ENSG00000170162 | 4.441747 | 2.151127 | 5.50E-04 | 8.14E-04 |
| KCNE3 | ENSG00000175538 | 4.437676 | 2.149804 | 1.29E-54 | 2.59E-53 |
| PKP1 | ENSG00000081277 | 4.437552 | 2.149764 | 2.04E-11 | 5.02E-11 |
| PRR25 | ENSG00000167945 | 4.436546 | 2.149437 | 1.74E-14 | 5.14E-14 |
| MICALL2 | ENSG00000164877 | 4.436126 | 2.149300 | 3.36E-48 | 5.19E-47 |
| MLIP | ENSG00000146147 | 4.435981 | 2.149253 | 1.43E-27 | 8.26E-27 |
| SLC22A16 | ENSG00000004809 | 4.429681 | 2.147203 | 1.37E-16 | 4.55E-16 |
| ASIC3 | ENSG00000213199 | 4.429310 | 2.147082 | 1.41E-26 | 7.78E-26 |
| IL9R | ENSG00000124334 | 4.428539 | 2.146831 | 2.14E-27 | 1.23E-26 |
| RELT | ENSG00000054967 | 4.428154 | 2.146706 | 8.10E-77 | 3.76E-75 |
| SLC16A8 | ENSG00000100156 | 4.428038 | 2.146667 | 2.45E-30 | 1.63E-29 |
| KIAA0408 | ENSG00000189367 | 4.426292 | 2.146099 | 2.03E-22 | 9.08E-22 |
| BATF3 | ENSG00000123685 | 4.425476 | 2.145833 | 1.55E-45 | 2.12E-44 |
| BCAN | ENSG00000132692 | 4.422744 | 2.144942 | 6.98E-20 | 2.76E-19 |
| LY9 | ENSG00000122224 | 4.422409 | 2.144833 | 1.73E-32 | 1.29E-31 |
| PYCARD | ENSG00000103490 | 4.419811 | 2.143985 | 4.93E-42 | 5.74E-41 |
| RNF113B | ENSG00000139797 | 4.413303 | 2.141859 | 1.57E-11 | 3.89E-11 |
| NPIPB12 | ENSG00000169203 | 4.409839 | 2.140726 | 2.33E-24 | 1.15E-23 |
| CARD11 | ENSG00000198286 | 4.403241 | 2.138566 | 7.81E-28 | 4.59E-27 |
| RAB7B | ENSG00000276600 | 4.402398 | 2.138290 | 6.85E-55 | 1.39E-53 |
| ERMN | ENSG00000136541 | 4.399773 | 2.137429 | 2.41E-23 | 1.13E-22 |
| RIN1 | ENSG00000174791 | 4.399286 | 2.137269 | 1.19E-41 | 1.36E-40 |
| DLGAP5 | ENSG00000126787 | 4.397813 | 2.136786 | 4.75E-26 | 2.56E-25 |
| CD5 | ENSG00000110448 | 4.397642 | 2.136730 | 4.93E-36 | 4.29E-35 |
| CD180 | ENSG00000134061 | 4.397232 | 2.136596 | 3.36E-34 | 2.70E-33 |
| APOBEC3C | ENSG00000244509 | 4.396423 | 2.136330 | 8.13E-61 | 2.05E-59 |
| SLC5A1 | ENSG00000100170 | 4.392286 | 2.134972 | 1.91E-11 | 4.71E-11 |
| SLFNL1 | ENSG00000171790 | 4.392087 | 2.134907 | 5.85E-35 | 4.88E-34 |
| COL5A2 | ENSG00000204262 | 4.388566 | 2.133750 | 2.75E-38 | 2.68E-37 |
| LBX2 | ENSG00000179528 | 4.382093 | 2.131620 | 2.20E-42 | 2.61E-41 |
| HIST1H3B | ENSG00000274267 | 4.381696 | 2.131489 | 6.67E-10 | 1.49E-09 |
| PLPPR3 | ENSG00000129951 | 4.378802 | 2.130536 | 8.21E-12 | 2.08E-11 |
| PTX4 | ENSG00000251692 | 4.375765 | 2.129535 | 7.46E-19 | 2.79E-18 |
| ALOX5 | ENSG00000012779 | 4.372468 | 2.128448 | 1.95E-31 | 1.37E-30 |
| RAC2 | ENSG00000128340 | 4.370625 | 2.127840 | 2.66E-41 | 2.99E-40 |
| ZNF469 | ENSG00000225614 | 4.369333 | 2.127413 | 8.61E-47 | 1.26E-45 |
| LINC01125 | ENSG00000228486 | 4.368766 | 2.127226 | 1.10E-36 | 9.90E-36 |
| KIAA0895L | ENSG00000196123 | 4.367384 | 2.126769 | 1.62E-45 | 2.21E-44 |
| GPR25 | ENSG00000170128 | 4.362661 | 2.125208 | 2.97E-18 | 1.08E-17 |
| GOLGA8B | ENSG00000215252 | 4.355736 | 2.122916 | 3.56E-23 | 1.66E-22 |
| ITK | ENSG00000113263 | 4.354576 | 2.122532 | 4.60E-34 | 3.69E-33 |
| ANKLE1 | ENSG00000160117 | 4.353887 | 2.122304 | 7.42E-31 | 5.08E-30 |
| DDI1 | ENSG00000170967 | 4.349495 | 2.120848 | 7.70E-12 | 1.95E-11 |
| TRIM73 | ENSG00000178809 | 4.347526 | 2.120195 | 7.88E-24 | 3.79E-23 |
| HCST | ENSG00000126264 | 4.347477 | 2.120178 | 1.63E-35 | 1.39E-34 |
| NBPF4 | ENSG00000196427 | 4.346175 | 2.119746 | 6.56E-08 | 1.29E-07 |
| MEF2B | ENSG00000213999 | 4.342903 | 2.118660 | 3.67E-35 | 3.09E-34 |
| NDC80 | ENSG00000080986 | 4.341491 | 2.118191 | 7.85E-50 | 1.31E-48 |
| DCST2 | ENSG00000163354 | 4.338940 | 2.117343 | 2.29E-28 | 1.39E-27 |
| TLR8 | ENSG00000101916 | 4.332969 | 2.115356 | 4.89E-27 | 2.77E-26 |
| THEMIS2 | ENSG00000130775 | 4.332459 | 2.115186 | 1.04E-58 | 2.41E-57 |
| TMEM233 | ENSG00000224982 | 4.331239 | 2.114780 | 1.10E-39 | 1.13E-38 |
| BDNF | ENSG00000176697 | 4.330609 | 2.114570 | 4.06E-22 | 1.79E-21 |
| STX16-NPEPL1 | ENSG00000254995 | 4.330332 | 2.114478 | 5.02E-23 | 2.31E-22 |
| APOA1 | ENSG00000118137 | 4.329247 | 2.114116 | 1.25E-05 | 2.10E-05 |
| MCAM | ENSG00000076706 | 4.315683 | 2.109589 | 3.07E-55 | 6.30E-54 |
| DEPDC1 | ENSG00000024526 | 4.312794 | 2.108623 | 3.95E-25 | 2.04E-24 |
| EXOC3L4 | ENSG00000205436 | 4.305863 | 2.106303 | 6.82E-28 | 4.02E-27 |
| AC069063.2 | ENSG00000279380 | 4.303991 | 2.105675 | 7.85E-10 | 1.74E-09 |
| CTRC | ENSG00000162438 | 4.302465 | 2.105163 | 3.26E-12 | 8.43E-12 |
| 12-Sep | ENSG00000140623 | 4.302391 | 2.105139 | 1.09E-08 | 2.24E-08 |
| GRIN2D | ENSG00000105464 | 4.299450 | 2.104152 | 1.03E-30 | 7.03E-30 |
| SLC16A6 | ENSG00000108932 | 4.293197 | 2.102053 | 1.81E-20 | 7.40E-20 |
| FOXL2NB | ENSG00000206262 | 4.292422 | 2.101792 | 2.15E-08 | 4.34E-08 |
| KRT15 | ENSG00000171346 | 4.288449 | 2.100456 | 4.05E-09 | 8.56E-09 |
| ABCB4 | ENSG00000005471 | 4.287392 | 2.100100 | 1.69E-25 | 8.90E-25 |
| CELA1 | ENSG00000139610 | 4.284665 | 2.099182 | 1.26E-15 | 3.97E-15 |
| ANXA2R | ENSG00000177721 | 4.283275 | 2.098714 | 5.21E-49 | 8.34E-48 |
| NUF2 | ENSG00000143228 | 4.280661 | 2.097834 | 4.09E-31 | 2.83E-30 |
| SPINK5 | ENSG00000133710 | 4.279938 | 2.097590 | 4.10E-16 | 1.33E-15 |
| SELPLG | ENSG00000110876 | 4.278918 | 2.097246 | 1.93E-58 | 4.43E-57 |
| DRD4 | ENSG00000069696 | 4.278137 | 2.096983 | 6.10E-31 | 4.19E-30 |
| TICRR | ENSG00000140534 | 4.277469 | 2.096757 | 1.04E-32 | 7.82E-32 |
| GPR18 | ENSG00000125245 | 4.277102 | 2.096634 | 2.74E-30 | 1.82E-29 |
| ARMC12 | ENSG00000157343 | 4.273935 | 2.095565 | 7.17E-25 | 3.66E-24 |
| MMP11 | ENSG00000099953 | 4.271362 | 2.094696 | 5.68E-41 | 6.27E-40 |
| HAVCR1 | ENSG00000113249 | 4.267031 | 2.093233 | 1.08E-16 | 3.61E-16 |
| GLDN | ENSG00000186417 | 4.264885 | 2.092507 | 3.30E-11 | 8.03E-11 |
| NHLH1 | ENSG00000171786 | 4.261476 | 2.091353 | 9.32E-32 | 6.70E-31 |
| ZDHHC19 | ENSG00000163958 | 4.258296 | 2.090276 | 8.39E-22 | 3.65E-21 |
| PTPN22 | ENSG00000134242 | 4.256352 | 2.089618 | 7.10E-40 | 7.41E-39 |
| SLC2A3 | ENSG00000059804 | 4.255076 | 2.089185 | 7.08E-36 | 6.12E-35 |
| SLC16A1 | ENSG00000155380 | 4.250432 | 2.087609 | 1.68E-58 | 3.86E-57 |
| TSPAN32 | ENSG00000064201 | 4.250338 | 2.087577 | 3.10E-44 | 3.94E-43 |
| CREG2 | ENSG00000175874 | 4.247518 | 2.086620 | 1.17E-18 | 4.33E-18 |
| TEN1-CDK3 | ENSG00000261408 | 4.244310 | 2.085530 | 2.93E-35 | 2.47E-34 |
| LMNTD2 | ENSG00000185522 | 4.242022 | 2.084752 | 4.40E-33 | 3.36E-32 |
| COL17A1 | ENSG00000065618 | 4.240718 | 2.084309 | 5.98E-09 | 1.25E-08 |
| IRF4 | ENSG00000137265 | 4.240608 | 2.084271 | 2.62E-19 | 1.01E-18 |
| NAA11 | ENSG00000156269 | 4.239560 | 2.083915 | 9.64E-05 | 1.52E-04 |
| NCF4 | ENSG00000100365 | 4.234775 | 2.082285 | 5.84E-51 | 1.03E-49 |
| ZNF114 | ENSG00000178150 | 4.230493 | 2.080826 | 9.66E-10 | 2.13E-09 |
| PRPH | ENSG00000135406 | 4.230401 | 2.080794 | 1.74E-11 | 4.31E-11 |
| CDC6 | ENSG00000094804 | 4.229188 | 2.080381 | 1.09E-40 | 1.17E-39 |
| UROC1 | ENSG00000159650 | 4.229027 | 2.080326 | 9.23E-15 | 2.77E-14 |
| SLC22A25 | ENSG00000196600 | 4.228966 | 2.080305 | 4.32E-05 | 6.96E-05 |
| RASL10A | ENSG00000100276 | 4.227857 | 2.079926 | 1.07E-32 | 8.03E-32 |
| ZIC4 | ENSG00000174963 | 4.220770 | 2.077506 | 6.16E-07 | 1.13E-06 |
| CIB3 | ENSG00000141977 | 4.217259 | 2.076306 | 5.55E-09 | 1.17E-08 |
| IKZF1 | ENSG00000185811 | 4.214604 | 2.075397 | 4.75E-42 | 5.54E-41 |
| LIMD2 | ENSG00000136490 | 4.208592 | 2.073338 | 1.32E-44 | 1.71E-43 |
| KRTAP5-11 | ENSG00000204571 | 4.208568 | 2.073329 | 2.19E-10 | 5.04E-10 |
| INSM2 | ENSG00000168348 | 4.208429 | 2.073282 | 3.67E-09 | 7.80E-09 |
| APOBEC3D | ENSG00000243811 | 4.205770 | 2.072370 | 6.58E-56 | 1.39E-54 |
| CCNA2 | ENSG00000145386 | 4.205424 | 2.072251 | 5.92E-40 | 6.19E-39 |
| TMEM255A | ENSG00000125355 | 4.202899 | 2.071385 | 5.55E-10 | 1.25E-09 |
| KCNE4 | ENSG00000152049 | 4.202264 | 2.071167 | 4.38E-45 | 5.84E-44 |
| TLR7 | ENSG00000196664 | 4.201549 | 2.070921 | 4.50E-29 | 2.82E-28 |
| RP11-644F5.10 | ENSG00000258311 | 4.199216 | 2.070120 | 2.35E-49 | 3.83E-48 |
| TYMS | ENSG00000176890 | 4.197757 | 2.069619 | 3.05E-80 | 1.61E-78 |
| HAUS7 | ENSG00000213397 | 4.196380 | 2.069145 | 3.22E-22 | 1.43E-21 |
| CBLN4 | ENSG00000054803 | 4.194420 | 2.068471 | 9.93E-17 | 3.32E-16 |
| CKAP2L | ENSG00000169607 | 4.193226 | 2.068061 | 2.25E-29 | 1.43E-28 |
| CLEC5A | ENSG00000258227 | 4.193024 | 2.067991 | 4.35E-28 | 2.60E-27 |
| FCGR2B | ENSG00000072694 | 4.192430 | 2.067787 | 3.32E-29 | 2.09E-28 |
| MGAM2 | ENSG00000257743 | 4.190382 | 2.067082 | 2.63E-18 | 9.57E-18 |
| KLK14 | ENSG00000129437 | 4.189642 | 2.066827 | 8.13E-18 | 2.89E-17 |
| CLEC18A | ENSG00000157322 | 4.189621 | 2.066820 | 1.61E-14 | 4.77E-14 |
| COL6A2 | ENSG00000142173 | 4.189487 | 2.066774 | 4.88E-38 | 4.70E-37 |
| MSR1 | ENSG00000038945 | 4.189154 | 2.066659 | 9.69E-37 | 8.75E-36 |
| SLC18A2 | ENSG00000165646 | 4.189088 | 2.066636 | 1.14E-13 | 3.21E-13 |
| KISS1 | ENSG00000170498 | 4.188436 | 2.066412 | 5.52E-09 | 1.16E-08 |
| GLYATL1P3 | ENSG00000255151 | 4.188112 | 2.066300 | 9.21E-12 | 2.32E-11 |
| CYGB | ENSG00000161544 | 4.187987 | 2.066257 | 2.62E-46 | 3.76E-45 |
| CATSPER1 | ENSG00000175294 | 4.185473 | 2.065391 | 4.00E-35 | 3.36E-34 |
| GAPT | ENSG00000175857 | 4.184278 | 2.064979 | 8.55E-31 | 5.83E-30 |
| SNAP25 | ENSG00000132639 | 4.182707 | 2.064437 | 3.31E-25 | 1.72E-24 |
| ITGAM | ENSG00000169896 | 4.182123 | 2.064236 | 6.14E-41 | 6.75E-40 |
| FGF20 | ENSG00000078579 | 4.181375 | 2.063978 | 5.12E-21 | 2.15E-20 |
| HLA-DPB1 | ENSG00000223865 | 4.178998 | 2.063157 | 8.02E-53 | 1.52E-51 |
| CTD-2568A17.1 | ENSG00000261341 | 4.178813 | 2.063093 | 2.35E-16 | 7.70E-16 |
| PHKG1 | ENSG00000164776 | 4.178194 | 2.062880 | 1.20E-35 | 1.03E-34 |
| PYGL | ENSG00000100504 | 4.177332 | 2.062582 | 1.83E-72 | 7.19E-71 |
| LAX1 | ENSG00000122188 | 4.174241 | 2.061514 | 3.22E-25 | 1.67E-24 |
| KIFC1 | ENSG00000237649 | 4.174001 | 2.061431 | 9.87E-42 | 1.13E-40 |
| RAB33A | ENSG00000134594 | 4.173133 | 2.061131 | 7.94E-36 | 6.86E-35 |
| HCK | ENSG00000101336 | 4.172393 | 2.060875 | 8.19E-56 | 1.72E-54 |
| FBXO41 | ENSG00000163013 | 4.169870 | 2.060002 | 2.19E-26 | 1.20E-25 |
| DDB2 | ENSG00000134574 | 4.169153 | 2.059754 | 8.15E-138 | 1.85E-135 |
| GPC2 | ENSG00000213420 | 4.168951 | 2.059684 | 3.40E-28 | 2.04E-27 |
| PREX2 | ENSG00000046889 | 4.168573 | 2.059553 | 2.60E-26 | 1.41E-25 |
| RP11-561B11.2 | ENSG00000258790 | 4.165953 | 2.058647 | 8.35E-21 | 3.47E-20 |
| SYT12 | ENSG00000173227 | 4.162208 | 2.057349 | 1.75E-15 | 5.44E-15 |
| PPEF2 | ENSG00000156194 | 4.161519 | 2.057110 | 3.44E-15 | 1.06E-14 |
| CLVS1 | ENSG00000177182 | 4.159496 | 2.056409 | 1.75E-14 | 5.16E-14 |
| TRIM46 | ENSG00000163462 | 4.153591 | 2.054359 | 1.82E-22 | 8.18E-22 |
| ARHGAP30 | ENSG00000186517 | 4.151092 | 2.053491 | 3.48E-54 | 6.91E-53 |
| FKBP11 | ENSG00000134285 | 4.146990 | 2.052065 | 6.61E-46 | 9.18E-45 |
| LRRC3B | ENSG00000179796 | 4.145946 | 2.051701 | 2.43E-10 | 5.59E-10 |
| KRT4 | ENSG00000170477 | 4.142480 | 2.050495 | 8.22E-06 | 1.40E-05 |
| NXF5 | ENSG00000126952 | 4.139328 | 2.049396 | 5.29E-17 | 1.81E-16 |
| SCN1B | ENSG00000105711 | 4.135489 | 2.048058 | 2.29E-64 | 6.84E-63 |
| ADAMTS4 | ENSG00000158859 | 4.134918 | 2.047859 | 1.51E-24 | 7.53E-24 |
| CYP26A1 | ENSG00000095596 | 4.134666 | 2.047771 | 8.57E-11 | 2.02E-10 |
| WNT8A | ENSG00000061492 | 4.130140 | 2.046191 | 6.35E-09 | 1.33E-08 |
| BIN2 | ENSG00000110934 | 4.128365 | 2.045571 | 4.55E-63 | 1.28E-61 |
| CRHR1 | ENSG00000120088 | 4.126432 | 2.044895 | 1.14E-05 | 1.93E-05 |
| FOXB1 | ENSG00000171956 | 4.120581 | 2.042848 | 7.28E-07 | 1.33E-06 |
| SFRP2 | ENSG00000145423 | 4.120152 | 2.042698 | 3.34E-09 | 7.11E-09 |
| GOLGA6A | ENSG00000159289 | 4.110297 | 2.039243 | 7.24E-10 | 1.61E-09 |
| GXYLT2 | ENSG00000172986 | 4.106131 | 2.037780 | 8.62E-19 | 3.21E-18 |
| IL32 | ENSG00000008517 | 4.104701 | 2.037277 | 1.36E-37 | 1.27E-36 |
| COL8A1 | ENSG00000144810 | 4.104421 | 2.037179 | 4.08E-31 | 2.83E-30 |
| MAPK15 | ENSG00000181085 | 4.102468 | 2.036492 | 9.98E-21 | 4.13E-20 |
| FOXH1 | ENSG00000160973 | 4.100008 | 2.035627 | 6.73E-19 | 2.52E-18 |
| LHFPL2 | ENSG00000145685 | 4.099902 | 2.035589 | 2.72E-48 | 4.22E-47 |
| XKR3 | ENSG00000172967 | 4.096108 | 2.034254 | 6.68E-10 | 1.49E-09 |
| LECT2 | ENSG00000145826 | 4.095468 | 2.034028 | 5.69E-10 | 1.28E-09 |
| LINGO1 | ENSG00000169783 | 4.092961 | 2.033145 | 7.04E-38 | 6.71E-37 |
| SLC2A1 | ENSG00000117394 | 4.091306 | 2.032561 | 7.02E-43 | 8.46E-42 |
| FAM209A | ENSG00000124103 | 4.089876 | 2.032057 | 8.40E-22 | 3.65E-21 |
| TOP2A | ENSG00000131747 | 4.087033 | 2.031054 | 1.78E-27 | 1.03E-26 |
| NCAPH | ENSG00000121152 | 4.081751 | 2.029188 | 1.09E-37 | 1.03E-36 |
| SCN1A | ENSG00000144285 | 4.081665 | 2.029158 | 3.16E-14 | 9.19E-14 |
| OPRD1 | ENSG00000116329 | 4.079077 | 2.028243 | 1.69E-26 | 9.29E-26 |
| NPPA | ENSG00000175206 | 4.078793 | 2.028142 | 8.40E-21 | 3.48E-20 |
| GBP2 | ENSG00000162645 | 4.076092 | 2.027187 | 1.42E-61 | 3.73E-60 |
| BTN3A2 | ENSG00000186470 | 4.072793 | 2.026019 | 7.67E-68 | 2.60E-66 |
| RDM1 | ENSG00000278023 | 4.069532 | 2.024863 | 3.70E-27 | 2.11E-26 |
| TAS2R13 | ENSG00000212128 | 4.068846 | 2.024620 | 1.82E-08 | 3.69E-08 |
| FAM9C | ENSG00000187268 | 4.066074 | 2.023636 | 2.19E-19 | 8.42E-19 |
| CNIH2 | ENSG00000174871 | 4.065304 | 2.023363 | 1.57E-33 | 1.22E-32 |
| SLC22A10 | ENSG00000184999 | 4.065200 | 2.023326 | 3.57E-06 | 6.20E-06 |
| ADAM12 | ENSG00000148848 | 4.064338 | 2.023021 | 4.07E-15 | 1.24E-14 |
| MILR1 | ENSG00000271605 | 4.063770 | 2.022819 | 2.05E-47 | 3.09E-46 |
| ASMT | ENSG00000196433 | 4.062382 | 2.022326 | 2.15E-19 | 8.27E-19 |
| P2RX5 | ENSG00000083454 | 4.061497 | 2.022012 | 1.54E-18 | 5.67E-18 |
| C22orf42 | ENSG00000205856 | 4.060612 | 2.021697 | 7.68E-13 | 2.06E-12 |
| HOXC13 | ENSG00000123364 | 4.059684 | 2.021367 | 1.06E-15 | 3.35E-15 |
| SLC6A7 | ENSG00000011083 | 4.054692 | 2.019592 | 2.20E-15 | 6.82E-15 |
| POU2F2 | ENSG00000028277 | 4.054134 | 2.019394 | 1.24E-35 | 1.06E-34 |
| C19orf84 | ENSG00000262874 | 4.047762 | 2.017125 | 3.09E-26 | 1.68E-25 |
| KIF14 | ENSG00000118193 | 4.042977 | 2.015418 | 1.15E-24 | 5.82E-24 |
| UGT2B17 | ENSG00000197888 | 4.041425 | 2.014864 | 2.28E-09 | 4.92E-09 |
| XAF1 | ENSG00000132530 | 4.041000 | 2.014712 | 7.22E-36 | 6.24E-35 |
| SP7 | ENSG00000170374 | 4.040138 | 2.014404 | 6.92E-11 | 1.65E-10 |
| RP11-434D12.1 | ENSG00000268279 | 4.038883 | 2.013956 | 4.26E-30 | 2.82E-29 |
| NPIPB4 | ENSG00000185864 | 4.037613 | 2.013503 | 9.73E-36 | 8.38E-35 |
| KRT82 | ENSG00000161850 | 4.036155 | 2.012982 | 1.03E-06 | 1.85E-06 |
| HIST1H3J | ENSG00000197153 | 4.032055 | 2.011515 | 6.40E-12 | 1.63E-11 |
| KRTAP16-1 | ENSG00000212657 | 4.029013 | 2.010426 | 1.46E-09 | 3.19E-09 |
| DNAJC5G | ENSG00000163793 | 4.028169 | 2.010124 | 1.47E-09 | 3.20E-09 |
| CAV2 | ENSG00000105971 | 4.025253 | 2.009079 | 9.58E-79 | 4.78E-77 |
| MASP1 | ENSG00000127241 | 4.024487 | 2.008805 | 8.36E-23 | 3.81E-22 |
| TRIM54 | ENSG00000138100 | 4.021585 | 2.007764 | 5.53E-09 | 1.16E-08 |
| OR2B6 | ENSG00000124657 | 4.021143 | 2.007606 | 3.23E-13 | 8.86E-13 |
| PCDH17 | ENSG00000118946 | 4.018832 | 2.006776 | 4.46E-35 | 3.74E-34 |
| GNG13 | ENSG00000127588 | 4.014336 | 2.005162 | 4.81E-07 | 8.88E-07 |
| LGALS9B | ENSG00000170298 | 4.011229 | 2.004044 | 4.82E-19 | 1.82E-18 |
| ADAMTS2 | ENSG00000087116 | 4.010454 | 2.003766 | 1.14E-34 | 9.40E-34 |
| CDKN3 | ENSG00000100526 | 4.007837 | 2.002824 | 1.51E-32 | 1.12E-31 |
| TRPV2 | ENSG00000187688 | 4.007595 | 2.002737 | 7.53E-92 | 5.51E-90 |
| RHBDF2 | ENSG00000129667 | 4.006255 | 2.002254 | 4.85E-99 | 4.20E-97 |
| CHST13 | ENSG00000180767 | 4.005845 | 2.002107 | 6.05E-22 | 2.65E-21 |
| EGFL8 | ENSG00000241404 | 4.002725 | 2.000983 | 4.29E-24 | 2.09E-23 |
| VSTM2L | ENSG00000132821 | 4.002352 | 2.000848 | 1.58E-07 | 3.03E-07 |
| PRELID2 | ENSG00000186314 | 4.001644 | 2.000593 | 2.17E-73 | 8.92E-72 |
