# Supplementary material for: A Computationally Constructed lncRNA-Associated Competing Triplet Network in Clear Cell Renal Cell Carcinoma
Source: Dis Markers. 2022 Nov 17;2022:8928282. doi: 10.1155/2022/8928282 (PMC9691318; doi:10.1155/2022/8928282)
Supplement: Supplementary Materials — Table S1: the list of upregulated lncRNAs in ccRCC. Table S2: the list of downregulated lncRNAs in ccRCC. Table S3: the list of upregulated mRNAs in ccRCC. Table S4: the list of downregulated mRNAs in ccRCC. Table S5: the list of upregulated miRNAs in ccRCC. Table S6: the list of downregulated miRNAs in ccRCC. Table S7: the list of top 100 dysregulated (50 upregulated and 50 downregulated) lncRNAs in consistent with Figure 1. Table S8: the list of genes coexpressed with HOTTIP in ccRCC. [file 8928282.f1.zip › 8928282.f1/Table S4 (1).docx]

Table S4. The list of down-regulated mRNAs in ccRCC.

| **Gene symbol** | **Ensemb ID** | **Fold Change (FC)  (T/N)** | **log_2_FC (T/N)** | ***P* value** | **FDR** |
| --- | --- | --- | --- | --- | --- |
| AQP2 | ENSG00000167580 | 0.002056 | -8.925668 | 4.27E-112 | 5.07E-110 |
| UMOD | ENSG00000169344 | 0.003051 | -8.356652 | 1.48E-102 | 1.43E-100 |
| SLC12A1 | ENSG00000074803 | 0.003392 | -8.203777 | 8.67E-195 | 6.56E-192 |
| TMEM207 | ENSG00000198398 | 0.003866 | -8.015043 | 1.24E-104 | 1.26E-102 |
| ELF5 | ENSG00000135374 | 0.004540 | -7.782962 | 3.64E-229 | 4.73E-226 |
| CALB1 | ENSG00000104327 | 0.005246 | -7.574681 | 8.57E-232 | 1.30E-228 |
| SEMG2 | ENSG00000124157 | 0.005528 | -7.499126 | 7.66E-153 | 2.57E-150 |
| MUC15 | ENSG00000169550 | 0.006401 | -7.287493 | 1.46E-111 | 1.70E-109 |
| NPHS2 | ENSG00000116218 | 0.006419 | -7.283473 | 6.00E-88 | 3.96E-86 |
| ATP12A | ENSG00000075673 | 0.006533 | -7.258083 | 1.61E-178 | 8.59E-176 |
| LINC00675 | ENSG00000263429 | 0.007133 | -7.131181 | 0.00E+00 | 0.00E+00 |
| UNCX | ENSG00000164853 | 0.008378 | -6.899226 | 1.34E-183 | 7.83E-181 |
| PRR35 | ENSG00000161992 | 0.008678 | -6.848350 | 2.49E-106 | 2.62E-104 |
| SOST | ENSG00000167941 | 0.008745 | -6.837345 | 1.51E-155 | 5.59E-153 |
| RP11-766F14.2 | ENSG00000248713 | 0.009034 | -6.790371 | 8.57E-129 | 1.56E-126 |
| DUSP9 | ENSG00000130829 | 0.009035 | -6.790252 | 1.66E-199 | 1.37E-196 |
| GP2 | ENSG00000169347 | 0.009499 | -6.718039 | 4.18E-133 | 8.63E-131 |
| CPNE6 | ENSG00000100884 | 0.011181 | -6.482768 | 3.98E-151 | 1.29E-148 |
| DDN | ENSG00000181418 | 0.011497 | -6.442567 | 4.71E-248 | 9.50E-245 |
| SLC14A2 | ENSG00000132874 | 0.011526 | -6.438929 | 3.15E-163 | 1.27E-160 |
| VGLL1 | ENSG00000102243 | 0.011692 | -6.418348 | 1.48E-83 | 8.43E-82 |
| FXYD4 | ENSG00000150201 | 0.012019 | -6.378576 | 6.33E-72 | 2.45E-70 |
| KCNJ1 | ENSG00000151704 | 0.012276 | -6.347969 | 1.79E-153 | 6.37E-151 |
| KCNJ10 | ENSG00000177807 | 0.014035 | -6.154802 | 7.16E-249 | 1.62E-245 |
| KLK7 | ENSG00000169035 | 0.014711 | -6.086995 | 1.39E-97 | 1.15E-95 |
| SLC9A4 | ENSG00000180251 | 0.014751 | -6.083042 | 3.38E-81 | 1.84E-79 |
| HS6ST2 | ENSG00000171004 | 0.015115 | -6.047910 | 5.11E-176 | 2.58E-173 |
| NPHS1 | ENSG00000161270 | 0.015135 | -6.046000 | 6.09E-149 | 1.76E-146 |
| SLC22A8 | ENSG00000149452 | 0.015188 | -6.040962 | 7.64E-99 | 6.58E-97 |
| CASR | ENSG00000036828 | 0.015609 | -6.001508 | 1.25E-173 | 6.11E-171 |
| KNG1 | ENSG00000113889 | 0.016985 | -5.879615 | 7.56E-67 | 2.46E-65 |
| CLDN16 | ENSG00000113946 | 0.017695 | -5.820519 | 1.34E-188 | 8.37E-186 |
| C2orf71 | ENSG00000179270 | 0.018049 | -5.791921 | 4.50E-115 | 5.60E-113 |
| ESRRB | ENSG00000119715 | 0.018579 | -5.750159 | 5.61E-240 | 1.02E-236 |
| TRPV5 | ENSG00000127412 | 0.018645 | -5.745101 | 1.55E-211 | 1.41E-208 |
| GPC5 | ENSG00000179399 | 0.018958 | -5.721030 | 2.86E-251 | 7.41E-248 |
| CLDN19 | ENSG00000164007 | 0.019247 | -5.699227 | 2.22E-151 | 7.33E-149 |
| TFAP2B | ENSG00000008196 | 0.019517 | -5.679135 | 1.24E-97 | 1.03E-95 |
| NELL1 | ENSG00000165973 | 0.019729 | -5.663525 | 3.44E-72 | 1.34E-70 |
| ACPP | ENSG00000014257 | 0.021049 | -5.570071 | 0.00E+00 | 0.00E+00 |
| CALML3 | ENSG00000178363 | 0.021150 | -5.563199 | 6.31E-108 | 6.82E-106 |
| RALYL | ENSG00000184672 | 0.021282 | -5.554213 | 1.41E-72 | 5.61E-71 |
| EGF | ENSG00000138798 | 0.022042 | -5.503606 | 2.86E-184 | 1.73E-181 |
| KLK6 | ENSG00000167755 | 0.022246 | -5.490309 | 5.03E-73 | 2.04E-71 |
| SH3GL3 | ENSG00000140600 | 0.022257 | -5.489568 | 4.49E-133 | 9.17E-131 |
| HCRTR2 | ENSG00000137252 | 0.022695 | -5.461488 | 1.35E-118 | 1.87E-116 |
| EHF | ENSG00000135373 | 0.023227 | -5.428066 | 5.09E-135 | 1.11E-132 |
| SLC7A13 | ENSG00000164893 | 0.023377 | -5.418767 | 1.35E-59 | 3.25E-58 |
| SFRP1 | ENSG00000104332 | 0.023971 | -5.382540 | 3.21E-179 | 1.77E-176 |
| RANBP3L | ENSG00000164188 | 0.025295 | -5.305026 | 2.12E-176 | 1.10E-173 |
| TYRP1 | ENSG00000107165 | 0.025437 | -5.296937 | 3.63E-85 | 2.19E-83 |
| ASB15 | ENSG00000146809 | 0.025848 | -5.273827 | 1.07E-98 | 9.19E-97 |
| XPNPEP2 | ENSG00000122121 | 0.026324 | -5.247469 | 9.87E-121 | 1.49E-118 |
| MFSD4A | ENSG00000174514 | 0.026775 | -5.222944 | 0.00E+00 | 0.00E+00 |
| CHP2 | ENSG00000166869 | 0.027723 | -5.172784 | 1.85E-136 | 4.14E-134 |
| HMX2 | ENSG00000188816 | 0.028487 | -5.133566 | 1.26E-43 | 1.56E-42 |
| OLFM3 | ENSG00000118733 | 0.028913 | -5.112135 | 3.21E-75 | 1.39E-73 |
| KCNE1B | ENSG00000276289 | 0.029000 | -5.107818 | 8.23E-130 | 1.54E-127 |
| LINC00371 | ENSG00000226792 | 0.029096 | -5.103044 | 4.79E-132 | 9.35E-130 |
| TMEM52B | ENSG00000165685 | 0.029151 | -5.100323 | 6.21E-133 | 1.25E-130 |
| HELT | ENSG00000187821 | 0.029436 | -5.086295 | 3.73E-81 | 2.02E-79 |
| SIAH3 | ENSG00000215475 | 0.030269 | -5.046019 | 6.78E-138 | 1.58E-135 |
| ENPP6 | ENSG00000164303 | 0.030457 | -5.037065 | 4.07E-228 | 4.92E-225 |
| NRK | ENSG00000123572 | 0.031457 | -4.990482 | 8.25E-116 | 1.06E-113 |
| EDDM3A | ENSG00000181562 | 0.031937 | -4.968639 | 1.87E-146 | 5.21E-144 |
| IRX2 | ENSG00000170561 | 0.032135 | -4.959693 | 4.62E-217 | 4.94E-214 |
| TCF24 | ENSG00000261787 | 0.032481 | -4.944275 | 5.73E-119 | 8.07E-117 |
| KLRG2 | ENSG00000188883 | 0.032506 | -4.943152 | 8.78E-93 | 6.56E-91 |
| CLCNKA | ENSG00000186510 | 0.032634 | -4.937479 | 2.80E-78 | 1.38E-76 |
| CST9 | ENSG00000173335 | 0.032700 | -4.934563 | 6.34E-85 | 3.75E-83 |
| PTGER1 | ENSG00000160951 | 0.033156 | -4.914596 | 1.34E-104 | 1.36E-102 |
| SEMG1 | ENSG00000124233 | 0.034102 | -4.873997 | 4.86E-94 | 3.71E-92 |
| IL36B | ENSG00000136696 | 0.034293 | -4.865925 | 6.18E-37 | 5.63E-36 |
| SLC12A3 | ENSG00000070915 | 0.034533 | -4.855888 | 1.35E-58 | 3.13E-57 |
| GABRA2 | ENSG00000151834 | 0.034687 | -4.849455 | 1.57E-49 | 2.58E-48 |
| SERPINA5 | ENSG00000188488 | 0.034687 | -4.849454 | 1.08E-106 | 1.15E-104 |
| TRPV6 | ENSG00000165125 | 0.035012 | -4.836017 | 3.92E-193 | 2.74E-190 |
| HPD | ENSG00000158104 | 0.035250 | -4.826245 | 5.51E-104 | 5.49E-102 |
| AFM | ENSG00000079557 | 0.035685 | -4.808556 | 4.98E-91 | 3.60E-89 |
| CYP4F2 | ENSG00000186115 | 0.036054 | -4.793688 | 1.09E-64 | 3.31E-63 |
| GGT6 | ENSG00000167741 | 0.036078 | -4.792737 | 1.72E-77 | 8.31E-76 |
| OLFM4 | ENSG00000102837 | 0.036119 | -4.791097 | 5.85E-64 | 1.72E-62 |
| TNNT2 | ENSG00000118194 | 0.037013 | -4.755826 | 2.89E-108 | 3.18E-106 |
| SLC5A2 | ENSG00000140675 | 0.037891 | -4.721995 | 2.37E-120 | 3.50E-118 |
| CNTD2 | ENSG00000105219 | 0.038166 | -4.711558 | 1.19E-149 | 3.65E-147 |
| ABCA4 | ENSG00000198691 | 0.038517 | -4.698367 | 7.94E-142 | 2.03E-139 |
| MCCD1 | ENSG00000204511 | 0.038605 | -4.695082 | 6.52E-64 | 1.91E-62 |
| ADAMTS19 | ENSG00000145808 | 0.040002 | -4.643769 | 1.18E-61 | 3.11E-60 |
| ERVMER34-1 | ENSG00000226887 | 0.040066 | -4.641484 | 8.77E-140 | 2.15E-137 |
| DIO1 | ENSG00000211452 | 0.041204 | -4.601084 | 5.91E-91 | 4.26E-89 |
| ALDOB | ENSG00000136872 | 0.041367 | -4.595365 | 1.01E-69 | 3.69E-68 |
| SLC13A3 | ENSG00000158296 | 0.041404 | -4.594102 | 2.33E-76 | 1.06E-74 |
| CA10 | ENSG00000154975 | 0.041605 | -4.587092 | 4.28E-46 | 6.05E-45 |
| NAT8L | ENSG00000185818 | 0.044189 | -4.500159 | 3.31E-94 | 2.55E-92 |
| COL26A1 | ENSG00000160963 | 0.044402 | -4.493246 | 3.03E-90 | 2.15E-88 |
| CRYAA | ENSG00000160202 | 0.044451 | -4.491641 | 6.52E-46 | 9.07E-45 |
| IYD | ENSG00000009765 | 0.044532 | -4.489010 | 3.16E-131 | 6.08E-129 |
| SCNN1G | ENSG00000166828 | 0.044551 | -4.488395 | 1.53E-48 | 2.40E-47 |
| UGT2A1 | ENSG00000173610 | 0.044566 | -4.487901 | 3.15E-34 | 2.54E-33 |
| SPTBN2 | ENSG00000173898 | 0.045303 | -4.464238 | 8.08E-104 | 8.02E-102 |
| SLC13A2 | ENSG00000007216 | 0.046457 | -4.427962 | 5.00E-52 | 9.26E-51 |
| SLC34A1 | ENSG00000131183 | 0.047106 | -4.407949 | 2.57E-63 | 7.35E-62 |
| SLC4A11 | ENSG00000088836 | 0.047116 | -4.407650 | 8.30E-197 | 6.55E-194 |
| NOS1 | ENSG00000089250 | 0.047133 | -4.407127 | 4.60E-153 | 1.58E-150 |
| SLC36A2 | ENSG00000186335 | 0.047722 | -4.389205 | 1.67E-50 | 2.90E-49 |
| SIM2 | ENSG00000159263 | 0.048213 | -4.374424 | 6.03E-282 | 2.74E-278 |
| NTNG1 | ENSG00000162631 | 0.048549 | -4.364417 | 1.59E-68 | 5.64E-67 |
| IRX1 | ENSG00000170549 | 0.049071 | -4.348976 | 1.06E-104 | 1.08E-102 |
| CYP2B6 | ENSG00000197408 | 0.049246 | -4.343861 | 7.73E-55 | 1.57E-53 |
| KCTD8 | ENSG00000183783 | 0.049440 | -4.338189 | 4.13E-131 | 7.81E-129 |
| DPEP1 | ENSG00000015413 | 0.049650 | -4.332048 | 3.20E-92 | 2.36E-90 |
| FAM3B | ENSG00000183844 | 0.049780 | -4.328276 | 2.27E-117 | 3.05E-115 |
| OVCH2 | ENSG00000183378 | 0.049821 | -4.327092 | 3.35E-73 | 1.36E-71 |
| MRLN | ENSG00000227877 | 0.049838 | -4.326607 | 4.33E-79 | 2.18E-77 |
| GADL1 | ENSG00000144644 | 0.050013 | -4.321548 | 1.44E-98 | 1.23E-96 |
| MT1G | ENSG00000125144 | 0.050687 | -4.302234 | 8.33E-80 | 4.30E-78 |
| PLPPR1 | ENSG00000148123 | 0.050757 | -4.300247 | 1.44E-59 | 3.45E-58 |
| PROX1 | ENSG00000117707 | 0.050793 | -4.299216 | 3.57E-142 | 9.39E-140 |
| FABP1 | ENSG00000163586 | 0.051653 | -4.274998 | 9.42E-57 | 2.05E-55 |
| SLC5A7 | ENSG00000115665 | 0.052079 | -4.263144 | 9.69E-117 | 1.27E-114 |
| ADGRF1 | ENSG00000153292 | 0.052092 | -4.262797 | 5.46E-50 | 9.23E-49 |
| FLRT1 | ENSG00000126500 | 0.053357 | -4.228172 | 2.47E-144 | 6.70E-142 |
| COL4A6 | ENSG00000197565 | 0.053501 | -4.224279 | 4.11E-101 | 3.85E-99 |
| UPP2 | ENSG00000007001 | 0.053737 | -4.217953 | 1.59E-124 | 2.67E-122 |
| MAPK4 | ENSG00000141639 | 0.053948 | -4.212283 | 4.75E-57 | 1.04E-55 |
| FAM19A4 | ENSG00000163377 | 0.054140 | -4.207148 | 1.20E-89 | 8.41E-88 |
| BRINP3 | ENSG00000162670 | 0.054990 | -4.184695 | 2.67E-36 | 2.36E-35 |
| FGF1 | ENSG00000113578 | 0.055003 | -4.184348 | 7.95E-190 | 5.15E-187 |
| RBP2 | ENSG00000114113 | 0.056197 | -4.153367 | 1.26E-222 | 1.43E-219 |
| RASL11B | ENSG00000128045 | 0.056348 | -4.149486 | 3.35E-109 | 3.78E-107 |
| MT1H | ENSG00000205358 | 0.056797 | -4.138035 | 1.36E-51 | 2.47E-50 |
| GAL3ST3 | ENSG00000175229 | 0.057667 | -4.116105 | 6.74E-63 | 1.88E-61 |
| DEFB125 | ENSG00000178591 | 0.058002 | -4.107758 | 5.73E-27 | 3.24E-26 |
| PIK3C2G | ENSG00000139144 | 0.058142 | -4.104279 | 3.36E-36 | 2.95E-35 |
| F11 | ENSG00000088926 | 0.058281 | -4.100821 | 5.65E-77 | 2.63E-75 |
| MYLK3 | ENSG00000140795 | 0.058594 | -4.093115 | 1.25E-253 | 3.77E-250 |
| NT5C1A | ENSG00000116981 | 0.058671 | -4.091205 | 1.09E-140 | 2.72E-138 |
| CH507-152C13.3 | ENSG00000276076 | 0.058873 | -4.086239 | 1.64E-24 | 8.16E-24 |
| PROZ | ENSG00000126231 | 0.058898 | -4.085647 | 2.01E-77 | 9.62E-76 |
| RNF212B | ENSG00000215277 | 0.059253 | -4.076976 | 3.43E-133 | 7.16E-131 |
| WNT9B | ENSG00000158955 | 0.059422 | -4.072859 | 3.42E-139 | 8.27E-137 |
| PSKH2 | ENSG00000147613 | 0.059459 | -4.071951 | 3.26E-42 | 3.83E-41 |
| CRHBP | ENSG00000145708 | 0.059599 | -4.068559 | 8.76E-129 | 1.57E-126 |
| TPPP2 | ENSG00000179636 | 0.059917 | -4.060882 | 3.18E-129 | 5.83E-127 |
| TNNI1 | ENSG00000159173 | 0.060895 | -4.037541 | 4.52E-102 | 4.29E-100 |
| MOGAT2 | ENSG00000166391 | 0.061059 | -4.033643 | 2.26E-57 | 5.02E-56 |
| ACOT12 | ENSG00000172497 | 0.061243 | -4.029308 | 8.27E-66 | 2.59E-64 |
| WNK4 | ENSG00000126562 | 0.062020 | -4.011114 | 1.30E-171 | 6.04E-169 |
| RP11-467J12.4 | ENSG00000277639 | 0.063023 | -3.987973 | 2.41E-153 | 8.41E-151 |
| NXPH2 | ENSG00000144227 | 0.063399 | -3.979389 | 1.22E-23 | 5.83E-23 |
| SCN7A | ENSG00000136546 | 0.064092 | -3.963703 | 2.17E-67 | 7.20E-66 |
| LMX1B | ENSG00000136944 | 0.064288 | -3.959304 | 6.15E-45 | 8.11E-44 |
| FAM151A | ENSG00000162391 | 0.064758 | -3.948803 | 9.01E-84 | 5.18E-82 |
| SLC47A2 | ENSG00000180638 | 0.066301 | -3.914834 | 7.15E-103 | 6.94E-101 |
| FRG2C | ENSG00000172969 | 0.066462 | -3.911321 | 1.37E-46 | 1.98E-45 |
| SGCZ | ENSG00000185053 | 0.066489 | -3.910750 | 2.32E-31 | 1.63E-30 |
| ERBB4 | ENSG00000178568 | 0.066513 | -3.910228 | 1.69E-66 | 5.45E-65 |
| RAG2 | ENSG00000175097 | 0.066717 | -3.905794 | 4.52E-98 | 3.80E-96 |
| HPCAL4 | ENSG00000116983 | 0.066892 | -3.902013 | 1.39E-74 | 5.91E-73 |
| TMPRSS4 | ENSG00000137648 | 0.067166 | -3.896118 | 3.26E-57 | 7.16E-56 |
| TMEM45B | ENSG00000151715 | 0.067294 | -3.893382 | 1.54E-150 | 4.81E-148 |
| UTS2R | ENSG00000181408 | 0.067351 | -3.892166 | 1.91E-41 | 2.16E-40 |
| WNT8B | ENSG00000075290 | 0.067372 | -3.891714 | 1.86E-164 | 7.87E-162 |
| CLIC5 | ENSG00000112782 | 0.067492 | -3.889139 | 1.07E-159 | 4.11E-157 |
| TSPAN8 | ENSG00000127324 | 0.067805 | -3.882468 | 3.11E-65 | 9.51E-64 |
| RP11-190A12.7 | ENSG00000256029 | 0.067942 | -3.879559 | 1.09E-85 | 6.68E-84 |
| STRA6 | ENSG00000137868 | 0.068084 | -3.876531 | 3.49E-87 | 2.26E-85 |
| PRR15 | ENSG00000176532 | 0.068253 | -3.872962 | 8.38E-130 | 1.55E-127 |
| HSPA2 | ENSG00000126803 | 0.069047 | -3.856272 | 1.68E-229 | 2.34E-226 |
| FRG2B | ENSG00000225899 | 0.069315 | -3.850686 | 3.08E-26 | 1.67E-25 |
| GRHL2 | ENSG00000083307 | 0.069328 | -3.850426 | 5.20E-45 | 6.90E-44 |
| IL36RN | ENSG00000136695 | 0.069377 | -3.849394 | 3.74E-33 | 2.87E-32 |
| TACR3 | ENSG00000169836 | 0.069643 | -3.843878 | 5.36E-45 | 7.09E-44 |
| ANKRD34B | ENSG00000189127 | 0.069878 | -3.839020 | 1.03E-66 | 3.34E-65 |
| PCDH15 | ENSG00000150275 | 0.070021 | -3.836062 | 1.54E-36 | 1.38E-35 |
| KLHL14 | ENSG00000197705 | 0.070411 | -3.828056 | 5.56E-164 | 2.30E-161 |
| MYO3B | ENSG00000071909 | 0.070663 | -3.822910 | 1.01E-76 | 4.64E-75 |
| EPN3 | ENSG00000049283 | 0.070987 | -3.816292 | 7.26E-59 | 1.69E-57 |
| C2orf54 | ENSG00000172478 | 0.071734 | -3.801191 | 2.15E-49 | 3.52E-48 |
| B4GALNT2 | ENSG00000167080 | 0.072460 | -3.786679 | 3.01E-63 | 8.54E-62 |
| PPP1R1B | ENSG00000131771 | 0.073135 | -3.773293 | 2.33E-59 | 5.55E-58 |
| ALDH3B2 | ENSG00000132746 | 0.073342 | -3.769207 | 1.31E-52 | 2.46E-51 |
| FMN2 | ENSG00000155816 | 0.074103 | -3.754328 | 9.35E-61 | 2.35E-59 |
| FAM83B | ENSG00000168143 | 0.074225 | -3.751945 | 2.07E-36 | 1.84E-35 |
| SCNN1B | ENSG00000168447 | 0.074293 | -3.750637 | 9.86E-53 | 1.86E-51 |
| PRDM16 | ENSG00000142611 | 0.074878 | -3.739305 | 2.33E-138 | 5.50E-136 |
| PTPRQ | ENSG00000139304 | 0.074980 | -3.737346 | 7.55E-49 | 1.20E-47 |
| GRM1 | ENSG00000152822 | 0.076700 | -3.704621 | 3.73E-85 | 2.24E-83 |
| CR2 | ENSG00000117322 | 0.076909 | -3.700705 | 1.59E-47 | 2.40E-46 |
| SSTR5 | ENSG00000162009 | 0.077096 | -3.697208 | 3.53E-53 | 6.74E-52 |
| LIPH | ENSG00000163898 | 0.077860 | -3.682969 | 7.57E-79 | 3.78E-77 |
| TUBAL3 | ENSG00000178462 | 0.078745 | -3.666669 | 1.20E-31 | 8.54E-31 |
| WNT7B | ENSG00000188064 | 0.079287 | -3.656781 | 1.40E-44 | 1.81E-43 |
| TMPRSS2 | ENSG00000184012 | 0.079358 | -3.655488 | 8.76E-41 | 9.53E-40 |
| DEFB132 | ENSG00000186458 | 0.079683 | -3.649581 | 7.46E-20 | 2.95E-19 |
| CHGB | ENSG00000089199 | 0.079902 | -3.645617 | 6.35E-64 | 1.86E-62 |
| GPC3 | ENSG00000147257 | 0.080401 | -3.636635 | 2.34E-102 | 2.25E-100 |
| HECW1 | ENSG00000002746 | 0.080657 | -3.632050 | 2.75E-86 | 1.73E-84 |
| HS3ST6 | ENSG00000162040 | 0.082201 | -3.604699 | 4.03E-35 | 3.38E-34 |
| SUSD4 | ENSG00000143502 | 0.082211 | -3.604518 | 2.79E-96 | 2.23E-94 |
| CNTN1 | ENSG00000018236 | 0.082322 | -3.602581 | 8.20E-56 | 1.72E-54 |
| INSRR | ENSG00000027644 | 0.082872 | -3.592975 | 1.85E-57 | 4.12E-56 |
| GMNC | ENSG00000205835 | 0.083774 | -3.577353 | 7.72E-47 | 1.13E-45 |
| SLC4A9 | ENSG00000113073 | 0.084131 | -3.571213 | 1.01E-37 | 9.53E-37 |
| CYP27B1 | ENSG00000111012 | 0.084851 | -3.558917 | 1.68E-110 | 1.91E-108 |
| FER1L6 | ENSG00000214814 | 0.084856 | -3.558835 | 9.97E-62 | 2.64E-60 |
| NPY2R | ENSG00000185149 | 0.084868 | -3.558630 | 1.25E-43 | 1.56E-42 |
| CTXN3 | ENSG00000205279 | 0.085190 | -3.553174 | 1.58E-30 | 1.06E-29 |
| C14orf37 | ENSG00000139971 | 0.085199 | -3.553022 | 1.00E-192 | 6.74E-190 |
| TCF21 | ENSG00000118526 | 0.085285 | -3.551572 | 5.62E-160 | 2.22E-157 |
| P2RX2 | ENSG00000187848 | 0.085452 | -3.548742 | 1.14E-72 | 4.55E-71 |
| SLC28A2 | ENSG00000137860 | 0.086447 | -3.532034 | 1.06E-120 | 1.59E-118 |
| FAM167A | ENSG00000154319 | 0.086723 | -3.527436 | 5.80E-119 | 8.10E-117 |
| EYA4 | ENSG00000112319 | 0.086792 | -3.526298 | 2.57E-125 | 4.37E-123 |
| VTCN1 | ENSG00000134258 | 0.086968 | -3.523368 | 1.27E-43 | 1.58E-42 |
| OVOL2 | ENSG00000125850 | 0.087598 | -3.512954 | 1.13E-42 | 1.34E-41 |
| ERICH4 | ENSG00000204978 | 0.087647 | -3.512158 | 3.25E-99 | 2.84E-97 |
| CLUL1 | ENSG00000079101 | 0.087884 | -3.508254 | 5.95E-194 | 4.32E-191 |
| FRG2 | ENSG00000205097 | 0.088096 | -3.504773 | 9.36E-24 | 4.49E-23 |
| MFSD6L | ENSG00000185156 | 0.088299 | -3.501454 | 6.03E-62 | 1.60E-60 |
| ITLN1 | ENSG00000179914 | 0.088476 | -3.498572 | 1.77E-68 | 6.26E-67 |
| ADGRF3 | ENSG00000173567 | 0.089481 | -3.482280 | 5.48E-235 | 9.04E-232 |
| G6PC | ENSG00000131482 | 0.089574 | -3.480769 | 3.60E-44 | 4.56E-43 |
| SLC7A8 | ENSG00000092068 | 0.090705 | -3.462675 | 1.33E-113 | 1.60E-111 |
| BSND | ENSG00000162399 | 0.090842 | -3.460490 | 1.25E-15 | 3.94E-15 |
| C9orf135 | ENSG00000204711 | 0.090934 | -3.459035 | 5.84E-60 | 1.43E-58 |
| SLC9A3 | ENSG00000066230 | 0.091450 | -3.450871 | 1.98E-70 | 7.35E-69 |
| EPB41L4B | ENSG00000095203 | 0.091482 | -3.450372 | 8.20E-58 | 1.84E-56 |
| KLK5 | ENSG00000167754 | 0.091829 | -3.444906 | 1.16E-28 | 7.13E-28 |
| FGF9 | ENSG00000102678 | 0.091934 | -3.443258 | 2.09E-27 | 1.20E-26 |
| MRAP2 | ENSG00000135324 | 0.091987 | -3.442434 | 1.11E-86 | 7.10E-85 |
| TBL1Y | ENSG00000092377 | 0.092043 | -3.441550 | 5.46E-41 | 6.04E-40 |
| ABCA13 | ENSG00000179869 | 0.092284 | -3.437770 | 1.63E-57 | 3.63E-56 |
| PAK6 | ENSG00000137843 | 0.092291 | -3.437667 | 5.01E-60 | 1.23E-58 |
| C1orf64 | ENSG00000183888 | 0.092474 | -3.434812 | 5.48E-38 | 5.26E-37 |
| RAB25 | ENSG00000132698 | 0.092589 | -3.433012 | 3.76E-35 | 3.16E-34 |
| PROM2 | ENSG00000155066 | 0.092839 | -3.429128 | 1.69E-49 | 2.78E-48 |
| NIPAL1 | ENSG00000163293 | 0.093138 | -3.424487 | 1.85E-135 | 4.10E-133 |
| FREM1 | ENSG00000164946 | 0.093280 | -3.422286 | 8.74E-68 | 2.95E-66 |
| TACSTD2 | ENSG00000184292 | 0.094138 | -3.409083 | 7.11E-61 | 1.80E-59 |
| KCNK10 | ENSG00000100433 | 0.094512 | -3.403360 | 1.74E-111 | 2.02E-109 |
| FRMD7 | ENSG00000165694 | 0.095390 | -3.390016 | 2.62E-20 | 1.06E-19 |
| AVPR2 | ENSG00000126895 | 0.095643 | -3.386201 | 1.35E-100 | 1.23E-98 |
| SMIM5 | ENSG00000204323 | 0.095796 | -3.383883 | 1.50E-68 | 5.32E-67 |
| S100A2 | ENSG00000196754 | 0.096034 | -3.380307 | 1.69E-84 | 9.82E-83 |
| MAL | ENSG00000172005 | 0.096053 | -3.380027 | 2.55E-59 | 6.05E-58 |
| FGF10 | ENSG00000070193 | 0.096996 | -3.365938 | 2.93E-44 | 3.74E-43 |
| SPTSSB | ENSG00000196542 | 0.097733 | -3.355016 | 2.07E-64 | 6.21E-63 |
| LHX1 | ENSG00000273706 | 0.097815 | -3.353802 | 5.08E-30 | 3.35E-29 |
| LRRC2 | ENSG00000163827 | 0.097978 | -3.351399 | 2.18E-73 | 8.92E-72 |
| CA8 | ENSG00000178538 | 0.098205 | -3.348063 | 7.52E-91 | 5.40E-89 |
| C7 | ENSG00000112936 | 0.098816 | -3.339106 | 1.32E-65 | 4.10E-64 |
| PAPPA2 | ENSG00000116183 | 0.099692 | -3.326382 | 4.75E-40 | 5.00E-39 |
| SLC2A12 | ENSG00000146411 | 0.099850 | -3.324099 | 1.04E-76 | 4.72E-75 |
| ZNF488 | ENSG00000265763 | 0.099869 | -3.323823 | 1.56E-74 | 6.58E-73 |
| FLJ22763 | ENSG00000241224 | 0.100558 | -3.313899 | 7.75E-41 | 8.47E-40 |
| LMO3 | ENSG00000048540 | 0.101136 | -3.305630 | 7.34E-101 | 6.73E-99 |
| SLC34A3 | ENSG00000198569 | 0.101344 | -3.302669 | 1.77E-58 | 4.07E-57 |
| SYT7 | ENSG00000011347 | 0.101345 | -3.302656 | 4.82E-62 | 1.29E-60 |
| POU3F4 | ENSG00000196767 | 0.101366 | -3.302359 | 2.09E-15 | 6.47E-15 |
| GSTM3 | ENSG00000134202 | 0.101730 | -3.297186 | 2.52E-202 | 2.18E-199 |
| CHL1 | ENSG00000134121 | 0.101804 | -3.296128 | 4.51E-66 | 1.44E-64 |
| HOXB9 | ENSG00000170689 | 0.103579 | -3.271197 | 4.36E-70 | 1.61E-68 |
| RBBP8NL | ENSG00000130701 | 0.103732 | -3.269061 | 9.25E-22 | 4.02E-21 |
| ANKRD2 | ENSG00000165887 | 0.103932 | -3.266288 | 1.02E-49 | 1.69E-48 |
| DPP6 | ENSG00000130226 | 0.104116 | -3.263732 | 9.13E-42 | 1.05E-40 |
| PLG | ENSG00000122194 | 0.104150 | -3.263268 | 1.22E-25 | 6.47E-25 |
| SLC30A2 | ENSG00000158014 | 0.104295 | -3.261254 | 6.30E-44 | 7.91E-43 |
| GPR12 | ENSG00000132975 | 0.104575 | -3.257396 | 1.93E-39 | 1.97E-38 |
| SPATA16 | ENSG00000144962 | 0.105069 | -3.250597 | 7.91E-45 | 1.04E-43 |
| FAM169A | ENSG00000198780 | 0.105653 | -3.242588 | 1.12E-67 | 3.78E-66 |
| DACH2 | ENSG00000126733 | 0.105720 | -3.241678 | 2.28E-65 | 7.00E-64 |
| ACSF2 | ENSG00000167107 | 0.106122 | -3.236200 | 7.67E-173 | 3.67E-170 |
| HSPB7 | ENSG00000173641 | 0.106559 | -3.230272 | 1.14E-65 | 3.55E-64 |
| SLC4A1 | ENSG00000004939 | 0.106787 | -3.227187 | 3.77E-23 | 1.75E-22 |
| LGI2 | ENSG00000153012 | 0.106851 | -3.226327 | 6.97E-142 | 1.81E-139 |
| TMEM213 | ENSG00000214128 | 0.107152 | -3.222269 | 7.72E-19 | 2.88E-18 |
| NHLRC4 | ENSG00000257108 | 0.107267 | -3.220716 | 1.18E-156 | 4.48E-154 |
| NMUR2 | ENSG00000132911 | 0.107399 | -3.218945 | 1.14E-40 | 1.23E-39 |
| ENTPD3 | ENSG00000168032 | 0.107506 | -3.217507 | 1.32E-81 | 7.33E-80 |
| TFCP2L1 | ENSG00000115112 | 0.107956 | -3.211485 | 3.90E-41 | 4.34E-40 |
| TNNC1 | ENSG00000114854 | 0.108128 | -3.209189 | 2.93E-70 | 1.08E-68 |
| AIF1L | ENSG00000126878 | 0.108741 | -3.201030 | 2.45E-120 | 3.59E-118 |
| PTH1R | ENSG00000160801 | 0.108780 | -3.200512 | 2.62E-97 | 2.15E-95 |
| LINC01207 | ENSG00000248771 | 0.108893 | -3.199016 | 1.40E-24 | 7.02E-24 |
| KSR2 | ENSG00000171435 | 0.109037 | -3.197110 | 5.29E-74 | 2.21E-72 |
| DAO | ENSG00000110887 | 0.109243 | -3.194387 | 3.79E-52 | 7.03E-51 |
| SLC30A8 | ENSG00000164756 | 0.109817 | -3.186820 | 1.25E-35 | 1.07E-34 |
| FOLR3 | ENSG00000110203 | 0.110153 | -3.182419 | 8.67E-40 | 9.00E-39 |
| TTPA | ENSG00000137561 | 0.110696 | -3.175330 | 7.15E-41 | 7.83E-40 |
| CRISP2 | ENSG00000124490 | 0.110758 | -3.174517 | 1.39E-17 | 4.87E-17 |
| PTPRO | ENSG00000151490 | 0.111276 | -3.167783 | 9.89E-120 | 1.41E-117 |
| PCDH9 | ENSG00000184226 | 0.111466 | -3.165330 | 2.25E-87 | 1.47E-85 |
| TMEM8C | ENSG00000187616 | 0.111597 | -3.163636 | 1.12E-45 | 1.54E-44 |
| IL11 | ENSG00000095752 | 0.112103 | -3.157100 | 6.77E-38 | 6.46E-37 |
| SIM1 | ENSG00000112246 | 0.112305 | -3.154500 | 6.69E-44 | 8.40E-43 |
| TMEM178A | ENSG00000152154 | 0.112661 | -3.149934 | 9.13E-120 | 1.31E-117 |
| ESRP1 | ENSG00000104413 | 0.113058 | -3.144862 | 1.56E-29 | 1.01E-28 |
| CBLN2 | ENSG00000141668 | 0.113068 | -3.144742 | 8.78E-48 | 1.34E-46 |
| SLC9A2 | ENSG00000115616 | 0.113216 | -3.142845 | 4.05E-31 | 2.81E-30 |
| SLC26A4 | ENSG00000091137 | 0.113523 | -3.138947 | 3.85E-75 | 1.66E-73 |
| CLDN8 | ENSG00000156284 | 0.113613 | -3.137800 | 1.69E-11 | 4.18E-11 |
| APELA | ENSG00000248329 | 0.113839 | -3.134931 | 9.35E-27 | 5.22E-26 |
| WNT7A | ENSG00000154764 | 0.114044 | -3.132337 | 1.42E-45 | 1.95E-44 |
| MYOZ2 | ENSG00000172399 | 0.114113 | -3.131462 | 5.58E-118 | 7.62E-116 |
| ESRRG | ENSG00000196482 | 0.114528 | -3.126231 | 3.49E-79 | 1.77E-77 |
| TMEM61 | ENSG00000143001 | 0.114939 | -3.121060 | 3.11E-25 | 1.62E-24 |
| PAPPA | ENSG00000182752 | 0.115090 | -3.119161 | 3.47E-103 | 3.40E-101 |
| RNF150 | ENSG00000170153 | 0.115215 | -3.117600 | 1.48E-120 | 2.20E-118 |
| CLCNKB | ENSG00000184908 | 0.115432 | -3.114884 | 1.95E-22 | 8.73E-22 |
| TCEAL2 | ENSG00000184905 | 0.115742 | -3.111010 | 6.56E-32 | 4.75E-31 |
| FP325317.1 | ENSG00000277737 | 0.116078 | -3.106828 | 4.53E-46 | 6.38E-45 |
| RIPPLY1 | ENSG00000147223 | 0.116256 | -3.104624 | 1.10E-123 | 1.80E-121 |
| PRSS22 | ENSG00000005001 | 0.116778 | -3.098158 | 2.67E-28 | 1.61E-27 |
| RPRM | ENSG00000177519 | 0.116842 | -3.097373 | 3.61E-29 | 2.27E-28 |
| MYBPH | ENSG00000133055 | 0.117463 | -3.089720 | 1.07E-65 | 3.34E-64 |
| KIAA2022 | ENSG00000050030 | 0.118328 | -3.079134 | 3.88E-51 | 6.91E-50 |
| ALDH6A1 | ENSG00000119711 | 0.118382 | -3.078480 | 1.30E-154 | 4.72E-152 |
| PLA2R1 | ENSG00000153246 | 0.118971 | -3.071315 | 3.42E-165 | 1.51E-162 |
| SULT2B1 | ENSG00000088002 | 0.119037 | -3.070524 | 2.24E-58 | 5.11E-57 |
| SLC16A5 | ENSG00000170190 | 0.119686 | -3.062674 | 5.82E-146 | 1.60E-143 |
| TRPM6 | ENSG00000119121 | 0.119771 | -3.061654 | 6.61E-112 | 7.74E-110 |
| GATA3 | ENSG00000107485 | 0.119943 | -3.059578 | 3.64E-80 | 1.91E-78 |
| FOXJ1 | ENSG00000129654 | 0.119971 | -3.059244 | 1.77E-41 | 2.00E-40 |
| SCN2A | ENSG00000136531 | 0.120320 | -3.055052 | 2.19E-53 | 4.21E-52 |
| RASSF10 | ENSG00000189431 | 0.120589 | -3.051827 | 9.22E-66 | 2.88E-64 |
| HS3ST5 | ENSG00000249853 | 0.120830 | -3.048948 | 7.37E-32 | 5.34E-31 |
| CCDC181 | ENSG00000117477 | 0.121008 | -3.046832 | 1.06E-117 | 1.44E-115 |
| TMEM72 | ENSG00000187783 | 0.121117 | -3.045530 | 4.72E-70 | 1.73E-68 |
| MTURN | ENSG00000180354 | 0.121470 | -3.041333 | 1.39E-213 | 1.33E-210 |
| SLC22A7 | ENSG00000137204 | 0.122106 | -3.033797 | 2.33E-32 | 1.73E-31 |
| TDGF1 | ENSG00000241186 | 0.122217 | -3.032486 | 3.56E-24 | 1.74E-23 |
| L1CAM | ENSG00000198910 | 0.122749 | -3.026221 | 4.02E-35 | 3.38E-34 |
| SOWAHA | ENSG00000198944 | 0.123040 | -3.022798 | 5.56E-50 | 9.39E-49 |
| CSF3 | ENSG00000108342 | 0.123257 | -3.020259 | 5.13E-22 | 2.25E-21 |
| AMPH | ENSG00000078053 | 0.123386 | -3.018744 | 1.87E-77 | 9.00E-76 |
| RPS6KA6 | ENSG00000072133 | 0.123387 | -3.018738 | 7.93E-65 | 2.41E-63 |
| GJA8 | ENSG00000121634 | 0.123399 | -3.018600 | 2.81E-40 | 2.99E-39 |
| CHRNA4 | ENSG00000101204 | 0.123454 | -3.017956 | 6.94E-23 | 3.18E-22 |
| ABAT | ENSG00000183044 | 0.123894 | -3.012818 | 3.20E-122 | 4.97E-120 |
| SLC15A2 | ENSG00000163406 | 0.123907 | -3.012666 | 4.23E-109 | 4.71E-107 |
| USP44 | ENSG00000136014 | 0.124215 | -3.009084 | 4.70E-144 | 1.26E-141 |
| SLC5A11 | ENSG00000158865 | 0.124598 | -3.004642 | 2.12E-61 | 5.46E-60 |
| ALX1 | ENSG00000180318 | 0.125064 | -2.999258 | 4.14E-41 | 4.60E-40 |
| KRT40 | ENSG00000204889 | 0.125319 | -2.996328 | 4.14E-33 | 3.16E-32 |
| SLC52A3 | ENSG00000101276 | 0.125836 | -2.990382 | 3.23E-88 | 2.18E-86 |
| PDILT | ENSG00000169340 | 0.128105 | -2.964599 | 5.83E-63 | 1.63E-61 |
| MYH7 | ENSG00000092054 | 0.128115 | -2.964489 | 6.29E-50 | 1.06E-48 |
| VWA2 | ENSG00000165816 | 0.128244 | -2.963039 | 1.80E-63 | 5.22E-62 |
| FAM46D | ENSG00000174016 | 0.128462 | -2.960583 | 1.37E-42 | 1.63E-41 |
| NDST3 | ENSG00000164100 | 0.128961 | -2.954995 | 7.97E-61 | 2.01E-59 |
| ODAM | ENSG00000109205 | 0.129107 | -2.953364 | 2.32E-12 | 6.04E-12 |
| MRO | ENSG00000134042 | 0.129152 | -2.952857 | 5.48E-106 | 5.72E-104 |
| TAGLN3 | ENSG00000144834 | 0.129644 | -2.947370 | 1.71E-30 | 1.15E-29 |
| DLK1 | ENSG00000185559 | 0.129829 | -2.945321 | 1.19E-21 | 5.14E-21 |
| TDRD5 | ENSG00000162782 | 0.130088 | -2.942443 | 2.82E-38 | 2.75E-37 |
| DEGS2 | ENSG00000168350 | 0.130343 | -2.939616 | 8.32E-90 | 5.88E-88 |
| MTNR1A | ENSG00000168412 | 0.130568 | -2.937130 | 2.75E-36 | 2.42E-35 |
| REEP6 | ENSG00000115255 | 0.130607 | -2.936698 | 4.91E-101 | 4.57E-99 |
| NKX6-2 | ENSG00000148826 | 0.130905 | -2.933403 | 2.46E-50 | 4.22E-49 |
| CCNI2 | ENSG00000205089 | 0.130924 | -2.933201 | 1.47E-125 | 2.54E-123 |
| PIPOX | ENSG00000179761 | 0.131298 | -2.929083 | 3.62E-65 | 1.10E-63 |
| SLC22A6 | ENSG00000197901 | 0.131369 | -2.928305 | 9.61E-27 | 5.36E-26 |
| C10orf82 | ENSG00000165863 | 0.131726 | -2.924388 | 4.39E-64 | 1.30E-62 |
| NKD1 | ENSG00000140807 | 0.132564 | -2.915237 | 1.03E-94 | 7.99E-93 |
| SLC14A1 | ENSG00000141469 | 0.132729 | -2.913446 | 6.63E-86 | 4.12E-84 |
| TMEM30B | ENSG00000182107 | 0.133025 | -2.910225 | 1.32E-54 | 2.65E-53 |
| SCNN1A | ENSG00000111319 | 0.133202 | -2.908312 | 3.94E-43 | 4.80E-42 |
| C1orf116 | ENSG00000182795 | 0.133375 | -2.906438 | 1.02E-40 | 1.11E-39 |
| TCEAL6 | ENSG00000204071 | 0.133474 | -2.905366 | 1.40E-34 | 1.15E-33 |
| MPP7 | ENSG00000150054 | 0.134011 | -2.899573 | 2.26E-113 | 2.70E-111 |
| GUCA1C | ENSG00000138472 | 0.134020 | -2.899483 | 2.48E-21 | 1.05E-20 |
| EPGN | ENSG00000182585 | 0.134413 | -2.895256 | 1.85E-29 | 1.19E-28 |
| TAC1 | ENSG00000006128 | 0.135528 | -2.883341 | 1.66E-19 | 6.45E-19 |
| BMP7 | ENSG00000101144 | 0.135616 | -2.882398 | 1.47E-22 | 6.61E-22 |
| EPCAM | ENSG00000119888 | 0.135674 | -2.881780 | 5.17E-107 | 5.52E-105 |
| ZNF804B | ENSG00000182348 | 0.135889 | -2.879497 | 8.18E-20 | 3.23E-19 |
| PCDHB1 | ENSG00000171815 | 0.136096 | -2.877305 | 5.70E-41 | 6.29E-40 |
| RP11-307N16.6 | ENSG00000273167 | 0.136361 | -2.874501 | 1.01E-78 | 5.03E-77 |
| SEMA6D | ENSG00000137872 | 0.136662 | -2.871317 | 1.53E-115 | 1.93E-113 |
| GRIK5 | ENSG00000105737 | 0.136781 | -2.870055 | 2.26E-43 | 2.78E-42 |
| GJA3 | ENSG00000121743 | 0.137060 | -2.867124 | 6.50E-53 | 1.23E-51 |
| PM20D1 | ENSG00000162877 | 0.137140 | -2.866279 | 7.47E-58 | 1.68E-56 |
| PNMT | ENSG00000141744 | 0.137208 | -2.865567 | 1.02E-42 | 1.22E-41 |
| OXGR1 | ENSG00000165621 | 0.137824 | -2.859098 | 6.39E-26 | 3.43E-25 |
| PCK1 | ENSG00000124253 | 0.137899 | -2.858318 | 5.14E-39 | 5.15E-38 |
| MAP3K15 | ENSG00000180815 | 0.138008 | -2.857171 | 9.83E-52 | 1.79E-50 |
| KLHL1 | ENSG00000150361 | 0.138402 | -2.853066 | 7.16E-19 | 2.68E-18 |
| SLC22A13 | ENSG00000172940 | 0.138509 | -2.851947 | 1.22E-36 | 1.09E-35 |
| RSPO1 | ENSG00000169218 | 0.138828 | -2.848634 | 1.09E-26 | 6.08E-26 |
| NCCRP1 | ENSG00000188505 | 0.139016 | -2.846674 | 3.02E-61 | 7.73E-60 |
| ADGRV1 | ENSG00000164199 | 0.139232 | -2.844437 | 7.25E-87 | 4.67E-85 |
| PDE1A | ENSG00000115252 | 0.139656 | -2.840054 | 9.71E-97 | 7.87E-95 |
| CPAMD8 | ENSG00000160111 | 0.140318 | -2.833225 | 3.63E-141 | 9.16E-139 |
| CRB2 | ENSG00000148204 | 0.140541 | -2.830935 | 3.03E-45 | 4.07E-44 |
| HSD11B2 | ENSG00000176387 | 0.140989 | -2.826345 | 2.07E-54 | 4.12E-53 |
| VSIG8 | ENSG00000243284 | 0.141796 | -2.818115 | 4.70E-85 | 2.81E-83 |
| C1orf87 | ENSG00000162598 | 0.143005 | -2.805858 | 1.03E-27 | 6.02E-27 |
| ADH1C | ENSG00000248144 | 0.143070 | -2.805202 | 6.73E-22 | 2.94E-21 |
| KCNA4 | ENSG00000182255 | 0.144178 | -2.794082 | 8.81E-28 | 5.17E-27 |
| SLC7A14 | ENSG00000013293 | 0.144231 | -2.793544 | 3.83E-29 | 2.41E-28 |
| CLDN14 | ENSG00000159261 | 0.144328 | -2.792579 | 2.70E-78 | 1.33E-76 |
| PAH | ENSG00000171759 | 0.144404 | -2.791814 | 3.75E-24 | 1.83E-23 |
| GPR22 | ENSG00000172209 | 0.144521 | -2.790651 | 9.79E-31 | 6.66E-30 |
| TJP3 | ENSG00000105289 | 0.144558 | -2.790280 | 3.25E-51 | 5.81E-50 |
| ATP1A1 | ENSG00000163399 | 0.144723 | -2.788629 | 7.65E-278 | 2.78E-274 |
| ZNF728 | ENSG00000269067 | 0.145520 | -2.780708 | 1.27E-33 | 9.94E-33 |
| CEACAM7 | ENSG00000007306 | 0.145521 | -2.780697 | 1.62E-19 | 6.29E-19 |
| CYP1A1 | ENSG00000140465 | 0.145653 | -2.779390 | 3.77E-16 | 1.22E-15 |
| PRRG2 | ENSG00000126460 | 0.145920 | -2.776752 | 7.10E-76 | 3.16E-74 |
| DNMT3L | ENSG00000142182 | 0.146224 | -2.773750 | 1.30E-30 | 8.78E-30 |
| ARSH | ENSG00000205667 | 0.146295 | -2.773047 | 1.21E-28 | 7.42E-28 |
| ZPBP | ENSG00000042813 | 0.146609 | -2.769952 | 6.27E-34 | 4.98E-33 |
| SOSTDC1 | ENSG00000171243 | 0.148105 | -2.755312 | 5.21E-29 | 3.26E-28 |
| C5orf67 | ENSG00000225940 | 0.148365 | -2.752781 | 1.84E-67 | 6.10E-66 |
| NOS1AP | ENSG00000198929 | 0.148696 | -2.749563 | 5.28E-96 | 4.19E-94 |
| CPA1 | ENSG00000091704 | 0.149038 | -2.746246 | 2.13E-40 | 2.28E-39 |
| RIMBP2 | ENSG00000060709 | 0.149386 | -2.742885 | 6.62E-39 | 6.61E-38 |
| GRIK2 | ENSG00000164418 | 0.149650 | -2.740338 | 3.47E-67 | 1.13E-65 |
| IGSF11 | ENSG00000144847 | 0.149655 | -2.740290 | 1.45E-40 | 1.56E-39 |
| MAL2 | ENSG00000147676 | 0.150059 | -2.736395 | 1.44E-67 | 4.83E-66 |
| CKM | ENSG00000104879 | 0.150580 | -2.731398 | 3.81E-55 | 7.80E-54 |
| GABRA4 | ENSG00000109158 | 0.151154 | -2.725911 | 9.35E-23 | 4.26E-22 |
| RP11-565P22.6 | ENSG00000254706 | 0.151155 | -2.725899 | 9.25E-54 | 1.80E-52 |
| C1orf226 | ENSG00000239887 | 0.151205 | -2.725426 | 5.24E-100 | 4.73E-98 |
| IL19 | ENSG00000142224 | 0.151287 | -2.724639 | 1.06E-36 | 9.55E-36 |
| SLC6A17 | ENSG00000197106 | 0.151528 | -2.722345 | 3.70E-62 | 9.94E-61 |
| ARL4D | ENSG00000175906 | 0.151788 | -2.719872 | 7.92E-74 | 3.29E-72 |
| CAPSL | ENSG00000152611 | 0.152490 | -2.713214 | 1.66E-43 | 2.06E-42 |
| C16orf89 | ENSG00000153446 | 0.152703 | -2.711202 | 1.10E-32 | 8.28E-32 |
| HTR3B | ENSG00000149305 | 0.152747 | -2.710785 | 5.80E-14 | 1.67E-13 |
| ZP2 | ENSG00000103310 | 0.153287 | -2.705690 | 2.69E-36 | 2.38E-35 |
| FOXA3 | ENSG00000170608 | 0.153625 | -2.702517 | 2.32E-28 | 1.40E-27 |
| NR0B2 | ENSG00000131910 | 0.153813 | -2.700746 | 3.62E-11 | 8.79E-11 |
| CCSER1 | ENSG00000184305 | 0.153857 | -2.700337 | 1.13E-113 | 1.36E-111 |
| ASS1 | ENSG00000130707 | 0.154115 | -2.697920 | 1.34E-115 | 1.71E-113 |
| ASPDH | ENSG00000204653 | 0.154376 | -2.695481 | 1.20E-48 | 1.89E-47 |
| PLA2G3 | ENSG00000100078 | 0.154957 | -2.690065 | 9.15E-18 | 3.24E-17 |
| CNTN5 | ENSG00000149972 | 0.155181 | -2.687973 | 1.47E-33 | 1.15E-32 |
| ATP6V1B1 | ENSG00000116039 | 0.155280 | -2.687056 | 1.40E-19 | 5.45E-19 |
| CTSV | ENSG00000136943 | 0.155324 | -2.686644 | 1.08E-57 | 2.43E-56 |
| CER1 | ENSG00000147869 | 0.155698 | -2.683174 | 1.07E-31 | 7.68E-31 |
| PLCL1 | ENSG00000115896 | 0.155967 | -2.680688 | 4.05E-128 | 7.06E-126 |
| EFHD1 | ENSG00000115468 | 0.156130 | -2.679177 | 4.88E-151 | 1.56E-148 |
| RNF223 | ENSG00000237330 | 0.156424 | -2.676462 | 5.07E-25 | 2.60E-24 |
| DLGAP2 | ENSG00000198010 | 0.156521 | -2.675571 | 6.70E-50 | 1.12E-48 |
| SLC4A8 | ENSG00000050438 | 0.156820 | -2.672819 | 5.31E-165 | 2.29E-162 |
| MAGEE2 | ENSG00000186675 | 0.156895 | -2.672126 | 1.31E-64 | 3.94E-63 |
| LAD1 | ENSG00000159166 | 0.157712 | -2.664640 | 1.18E-46 | 1.72E-45 |
| CCDC185 | ENSG00000178395 | 0.158296 | -2.659301 | 6.70E-20 | 2.65E-19 |
| KCNK13 | ENSG00000152315 | 0.159334 | -2.649875 | 3.59E-132 | 7.08E-130 |
| FAM222A | ENSG00000139438 | 0.160911 | -2.635662 | 1.74E-82 | 9.84E-81 |
| ADAMTS16 | ENSG00000145536 | 0.161123 | -2.633764 | 2.95E-57 | 6.50E-56 |
| MUC6 | ENSG00000184956 | 0.161185 | -2.633214 | 3.02E-42 | 3.55E-41 |
| TTC36 | ENSG00000172425 | 0.161425 | -2.631060 | 1.05E-55 | 2.20E-54 |
| ADH6 | ENSG00000172955 | 0.161834 | -2.627412 | 1.57E-50 | 2.72E-49 |
| AQP5 | ENSG00000161798 | 0.161985 | -2.626065 | 2.55E-21 | 1.08E-20 |
| EMX1 | ENSG00000135638 | 0.162524 | -2.621272 | 8.98E-117 | 1.19E-114 |
| ITGB6 | ENSG00000115221 | 0.162892 | -2.618013 | 2.31E-57 | 5.12E-56 |
| ATP6V0A4 | ENSG00000105929 | 0.163047 | -2.616641 | 8.28E-13 | 2.22E-12 |
| ANGPTL1 | ENSG00000116194 | 0.163188 | -2.615392 | 1.66E-44 | 2.13E-43 |
| HMGCS2 | ENSG00000134240 | 0.163455 | -2.613033 | 4.89E-25 | 2.51E-24 |
| LGR5 | ENSG00000139292 | 0.163915 | -2.608981 | 1.14E-23 | 5.45E-23 |
| FGFBP1 | ENSG00000137440 | 0.164162 | -2.606805 | 4.87E-15 | 1.48E-14 |
| RAP1GAP | ENSG00000076864 | 0.164565 | -2.603271 | 2.50E-71 | 9.50E-70 |
| MECOM | ENSG00000085276 | 0.164743 | -2.601714 | 2.80E-133 | 5.91E-131 |
| SPTB | ENSG00000070182 | 0.164967 | -2.599749 | 2.59E-98 | 2.20E-96 |
| IGSF10 | ENSG00000152580 | 0.165081 | -2.598754 | 1.43E-58 | 3.31E-57 |
| DUSP26 | ENSG00000133878 | 0.165372 | -2.596213 | 4.59E-44 | 5.79E-43 |
| LYPD6B | ENSG00000150556 | 0.165718 | -2.593194 | 1.22E-18 | 4.53E-18 |
| GALNT3 | ENSG00000115339 | 0.166113 | -2.589764 | 8.16E-72 | 3.15E-70 |
| CNGA1 | ENSG00000198515 | 0.166523 | -2.586203 | 4.62E-60 | 1.14E-58 |
| SH3GL2 | ENSG00000107295 | 0.167194 | -2.580405 | 3.37E-20 | 1.36E-19 |
| NUDT10 | ENSG00000122824 | 0.168471 | -2.569429 | 1.43E-63 | 4.15E-62 |
| CAMK2A | ENSG00000070808 | 0.169029 | -2.564655 | 5.02E-40 | 5.27E-39 |
| ATP4B | ENSG00000186009 | 0.169226 | -2.562978 | 3.94E-29 | 2.48E-28 |
| MPPED2 | ENSG00000066382 | 0.170779 | -2.549800 | 6.22E-68 | 2.11E-66 |
| CNKSR1 | ENSG00000142675 | 0.171405 | -2.544518 | 3.54E-42 | 4.15E-41 |
| NYAP1 | ENSG00000166924 | 0.171470 | -2.543975 | 4.17E-78 | 2.05E-76 |
| LYPD6 | ENSG00000187123 | 0.172333 | -2.536730 | 1.90E-37 | 1.77E-36 |
| CPNE4 | ENSG00000196353 | 0.172562 | -2.534812 | 7.30E-28 | 4.29E-27 |
| RASD1 | ENSG00000108551 | 0.173164 | -2.529787 | 3.30E-71 | 1.25E-69 |
| AGTR2 | ENSG00000180772 | 0.173355 | -2.528201 | 1.16E-12 | 3.09E-12 |
| OTOGL | ENSG00000165899 | 0.173480 | -2.527158 | 4.04E-58 | 9.16E-57 |
| PKHD1L1 | ENSG00000205038 | 0.173663 | -2.525635 | 1.69E-42 | 2.01E-41 |
| RNF43 | ENSG00000108375 | 0.173816 | -2.524368 | 8.46E-86 | 5.24E-84 |
| HAO2 | ENSG00000116882 | 0.174170 | -2.521435 | 2.05E-33 | 1.59E-32 |
| GABRG3 | ENSG00000182256 | 0.174173 | -2.521406 | 6.89E-26 | 3.69E-25 |
| AGBL4 | ENSG00000186094 | 0.174575 | -2.518083 | 1.26E-123 | 2.04E-121 |
| FAM46B | ENSG00000158246 | 0.174656 | -2.517414 | 2.22E-61 | 5.71E-60 |
| C5orf38 | ENSG00000186493 | 0.174674 | -2.517263 | 2.81E-31 | 1.96E-30 |
| FAM81A | ENSG00000157470 | 0.175328 | -2.511868 | 7.21E-116 | 9.42E-114 |
| KCNB2 | ENSG00000182674 | 0.175647 | -2.509252 | 2.70E-24 | 1.33E-23 |
| BMPR1B | ENSG00000138696 | 0.176338 | -2.503583 | 3.46E-20 | 1.39E-19 |
| ADH1B | ENSG00000196616 | 0.176784 | -2.499938 | 2.88E-29 | 1.82E-28 |
| ERMP1 | ENSG00000099219 | 0.176956 | -2.498535 | 5.71E-183 | 3.24E-180 |
| CHST6 | ENSG00000183196 | 0.177574 | -2.493506 | 4.48E-33 | 3.41E-32 |
| TUBB2B | ENSG00000137285 | 0.177666 | -2.492764 | 1.27E-55 | 2.64E-54 |
| AGMAT | ENSG00000116771 | 0.178288 | -2.487722 | 4.00E-56 | 8.51E-55 |
| VAT1L | ENSG00000171724 | 0.178653 | -2.484766 | 1.00E-27 | 5.87E-27 |
| RDH8 | ENSG00000080511 | 0.178820 | -2.483423 | 1.20E-10 | 2.80E-10 |
| GPAT3 | ENSG00000138678 | 0.179105 | -2.481119 | 7.23E-51 | 1.27E-49 |
| SLITRK3 | ENSG00000121871 | 0.179506 | -2.477900 | 2.36E-22 | 1.05E-21 |
| PPP1R1A | ENSG00000135447 | 0.180154 | -2.472699 | 1.47E-20 | 6.04E-20 |
| FSTL4 | ENSG00000053108 | 0.180473 | -2.470145 | 1.44E-39 | 1.48E-38 |
| PPP1R36 | ENSG00000165807 | 0.180925 | -2.466536 | 1.10E-48 | 1.73E-47 |
| SRGAP3 | ENSG00000196220 | 0.181613 | -2.461061 | 3.13E-134 | 6.70E-132 |
| AADACL4 | ENSG00000204518 | 0.181817 | -2.459445 | 2.30E-26 | 1.25E-25 |
| TTC29 | ENSG00000137473 | 0.181819 | -2.459424 | 1.04E-40 | 1.13E-39 |
| SFTA2 | ENSG00000196260 | 0.181834 | -2.459304 | 3.01E-20 | 1.22E-19 |
| CGN | ENSG00000143375 | 0.182010 | -2.457910 | 3.74E-68 | 1.29E-66 |
| WT1 | ENSG00000184937 | 0.182149 | -2.456813 | 3.50E-25 | 1.81E-24 |
| DCN | ENSG00000011465 | 0.183002 | -2.450072 | 5.98E-34 | 4.76E-33 |
| LRP1B | ENSG00000168702 | 0.183256 | -2.448067 | 5.43E-28 | 3.22E-27 |
| RBM11 | ENSG00000185272 | 0.183300 | -2.447721 | 1.34E-32 | 1.00E-31 |
| GPR182 | ENSG00000166856 | 0.183381 | -2.447085 | 1.59E-62 | 4.34E-61 |
| PCP4 | ENSG00000183036 | 0.183569 | -2.445604 | 2.06E-13 | 5.72E-13 |
| GREM1 | ENSG00000166923 | 0.183693 | -2.444635 | 4.84E-24 | 2.36E-23 |
| NPAP1 | ENSG00000185823 | 0.183827 | -2.443577 | 2.66E-24 | 1.31E-23 |
| GLOD5 | ENSG00000171433 | 0.183832 | -2.443541 | 2.43E-48 | 3.77E-47 |
| FMO5 | ENSG00000131781 | 0.184135 | -2.441163 | 1.19E-85 | 7.32E-84 |
| PLEKHD1 | ENSG00000175985 | 0.184147 | -2.441074 | 9.40E-34 | 7.42E-33 |
| NDNF | ENSG00000173376 | 0.185070 | -2.433859 | 6.29E-18 | 2.25E-17 |
| AP1M2 | ENSG00000129354 | 0.185419 | -2.431140 | 2.77E-59 | 6.54E-58 |
| FBXO2 | ENSG00000116661 | 0.185664 | -2.429233 | 4.08E-43 | 4.96E-42 |
| PSAT1 | ENSG00000135069 | 0.185813 | -2.428076 | 3.89E-35 | 3.27E-34 |
| PNPLA1 | ENSG00000180316 | 0.185840 | -2.427870 | 8.18E-45 | 1.07E-43 |
| PLPP4 | ENSG00000203805 | 0.185858 | -2.427730 | 1.07E-30 | 7.25E-30 |
| FUT3 | ENSG00000171124 | 0.186969 | -2.419130 | 3.04E-39 | 3.08E-38 |
| GGACT | ENSG00000134864 | 0.188211 | -2.409579 | 8.53E-76 | 3.78E-74 |
| COBLL1 | ENSG00000082438 | 0.188281 | -2.409043 | 2.29E-149 | 6.82E-147 |
| KCNQ2 | ENSG00000075043 | 0.189804 | -2.397419 | 1.40E-20 | 5.76E-20 |
| FAM217A | ENSG00000145975 | 0.189999 | -2.395936 | 4.37E-72 | 1.70E-70 |
| GCGR | ENSG00000215644 | 0.190032 | -2.395688 | 5.74E-10 | 1.28E-09 |
| DOK7 | ENSG00000175920 | 0.190511 | -2.392056 | 4.26E-42 | 4.97E-41 |
| KCNJ13 | ENSG00000115474 | 0.190838 | -2.389576 | 8.98E-18 | 3.19E-17 |
| HPSE2 | ENSG00000172987 | 0.191048 | -2.387990 | 4.69E-37 | 4.29E-36 |
| MARVELD2 | ENSG00000152939 | 0.191070 | -2.387829 | 1.92E-88 | 1.31E-86 |
| DACH1 | ENSG00000276644 | 0.191599 | -2.383837 | 1.91E-77 | 9.20E-76 |
| DDX25 | ENSG00000109832 | 0.191875 | -2.381759 | 4.31E-41 | 4.78E-40 |
| PADI2 | ENSG00000117115 | 0.191928 | -2.381362 | 7.15E-61 | 1.81E-59 |
| CEL | ENSG00000170835 | 0.191996 | -2.380852 | 1.79E-22 | 8.03E-22 |
| NPY5R | ENSG00000164129 | 0.192522 | -2.376902 | 7.41E-58 | 1.67E-56 |
| DNER | ENSG00000187957 | 0.192641 | -2.376014 | 1.85E-19 | 7.17E-19 |
| CLDN11 | ENSG00000013297 | 0.192842 | -2.374506 | 3.56E-54 | 7.05E-53 |
| FOXN1 | ENSG00000109101 | 0.193234 | -2.371580 | 8.83E-30 | 5.76E-29 |
| CCDC160 | ENSG00000203952 | 0.193740 | -2.367804 | 7.33E-66 | 2.30E-64 |
| MELTF | ENSG00000163975 | 0.193814 | -2.367253 | 4.94E-29 | 3.09E-28 |
| SPRR1A | ENSG00000169474 | 0.194222 | -2.364221 | 2.30E-09 | 4.96E-09 |
| LINGO2 | ENSG00000174482 | 0.195832 | -2.352313 | 7.65E-23 | 3.49E-22 |
| ETNPPL | ENSG00000164089 | 0.196292 | -2.348924 | 2.49E-16 | 8.13E-16 |
| CDH16 | ENSG00000166589 | 0.196850 | -2.344834 | 2.58E-77 | 1.22E-75 |
| PEG3 | ENSG00000198300 | 0.196992 | -2.343794 | 4.83E-86 | 3.03E-84 |
| FBP1 | ENSG00000165140 | 0.196995 | -2.343766 | 4.03E-68 | 1.38E-66 |
| PLEKHB1 | ENSG00000021300 | 0.197647 | -2.339002 | 2.62E-56 | 5.61E-55 |
| SHISA2 | ENSG00000180730 | 0.197805 | -2.337848 | 1.90E-33 | 1.48E-32 |
| OVOL1 | ENSG00000172818 | 0.198049 | -2.336071 | 1.21E-46 | 1.76E-45 |
| ANKRD63 | ENSG00000230778 | 0.198319 | -2.334103 | 6.58E-27 | 3.71E-26 |
| NR1I3 | ENSG00000143257 | 0.198684 | -2.331451 | 5.24E-53 | 9.94E-52 |
| ISX | ENSG00000175329 | 0.198785 | -2.330721 | 5.71E-12 | 1.46E-11 |
| GLDC | ENSG00000178445 | 0.199038 | -2.328882 | 1.99E-55 | 4.12E-54 |
| BPI | ENSG00000101425 | 0.199568 | -2.325048 | 1.34E-47 | 2.03E-46 |
| MUC13 | ENSG00000173702 | 0.199625 | -2.324635 | 6.64E-21 | 2.77E-20 |
| AKAP3 | ENSG00000111254 | 0.200014 | -2.321827 | 6.95E-138 | 1.60E-135 |
| PDGFRA | ENSG00000134853 | 0.200120 | -2.321061 | 2.40E-33 | 1.86E-32 |
| SFXN2 | ENSG00000156398 | 0.200181 | -2.320620 | 3.61E-117 | 4.82E-115 |
| SLCO1A2 | ENSG00000084453 | 0.200794 | -2.316210 | 7.05E-15 | 2.13E-14 |
| UMODL1 | ENSG00000177398 | 0.200881 | -2.315589 | 6.45E-20 | 2.56E-19 |
| MAP6 | ENSG00000171533 | 0.200965 | -2.314982 | 4.46E-85 | 2.67E-83 |
| DTX1 | ENSG00000135144 | 0.201242 | -2.312998 | 4.47E-82 | 2.49E-80 |
| TREH | ENSG00000118094 | 0.201823 | -2.308837 | 3.39E-32 | 2.48E-31 |
| CCL11 | ENSG00000172156 | 0.201998 | -2.307589 | 4.14E-22 | 1.83E-21 |
| PMP2 | ENSG00000147588 | 0.202260 | -2.305720 | 2.67E-11 | 6.52E-11 |
| CLDN10 | ENSG00000134873 | 0.202303 | -2.305407 | 3.83E-76 | 1.72E-74 |
| RXFP4 | ENSG00000173080 | 0.202361 | -2.304994 | 7.03E-43 | 8.47E-42 |
| MIOX | ENSG00000100253 | 0.202541 | -2.303711 | 4.39E-28 | 2.62E-27 |
| CLEC3A | ENSG00000166509 | 0.202816 | -2.301757 | 2.69E-14 | 7.85E-14 |
| GMPR | ENSG00000137198 | 0.203045 | -2.300130 | 2.84E-62 | 7.68E-61 |
| DMRT2 | ENSG00000173253 | 0.203049 | -2.300104 | 3.48E-09 | 7.40E-09 |
| LZTS3 | ENSG00000088899 | 0.203805 | -2.294739 | 9.83E-97 | 7.93E-95 |
| MYF6 | ENSG00000111046 | 0.204147 | -2.292316 | 9.24E-23 | 4.21E-22 |
| CYFIP2 | ENSG00000055163 | 0.204150 | -2.292301 | 1.76E-132 | 3.52E-130 |
| PLA2G4F | ENSG00000168907 | 0.204405 | -2.290496 | 4.01E-09 | 8.50E-09 |
| WSCD2 | ENSG00000075035 | 0.204901 | -2.286999 | 2.44E-26 | 1.33E-25 |
| ARC | ENSG00000198576 | 0.205516 | -2.282680 | 5.13E-53 | 9.74E-52 |
| PTCHD3 | ENSG00000182077 | 0.205716 | -2.281274 | 3.15E-21 | 1.33E-20 |
| GABRP | ENSG00000094755 | 0.205729 | -2.281181 | 6.77E-28 | 4.00E-27 |
| CLSTN2 | ENSG00000158258 | 0.206601 | -2.275079 | 3.15E-31 | 2.20E-30 |
| LDHD | ENSG00000166816 | 0.206653 | -2.274719 | 3.05E-47 | 4.56E-46 |
| ACKR2 | ENSG00000144648 | 0.206723 | -2.274229 | 2.47E-66 | 7.95E-65 |
| TOX3 | ENSG00000103460 | 0.207024 | -2.272133 | 1.23E-27 | 7.19E-27 |
| SYN2 | ENSG00000157152 | 0.207079 | -2.271749 | 6.73E-43 | 8.13E-42 |
| UPK1A | ENSG00000105668 | 0.207206 | -2.270861 | 2.60E-17 | 9.02E-17 |
| PLXNA4 | ENSG00000221866 | 0.207367 | -2.269745 | 5.18E-32 | 3.77E-31 |
| RBP3 | ENSG00000265203 | 0.207578 | -2.268274 | 6.83E-20 | 2.70E-19 |
| PCK2 | ENSG00000100889 | 0.207967 | -2.265577 | 1.91E-99 | 1.70E-97 |
| TNS4 | ENSG00000131746 | 0.208180 | -2.264096 | 2.88E-31 | 2.01E-30 |
| CACNA2D2 | ENSG00000007402 | 0.208494 | -2.261923 | 6.76E-122 | 1.04E-119 |
| SIX4 | ENSG00000100625 | 0.208771 | -2.260007 | 3.48E-24 | 1.70E-23 |
| GATM | ENSG00000171766 | 0.208781 | -2.259935 | 1.12E-56 | 2.43E-55 |
| AKAP4 | ENSG00000147081 | 0.209499 | -2.254985 | 5.95E-39 | 5.95E-38 |
| SLC25A47 | ENSG00000140107 | 0.209687 | -2.253692 | 2.18E-36 | 1.93E-35 |
| TMEM174 | ENSG00000164325 | 0.210223 | -2.250010 | 2.70E-19 | 1.03E-18 |
| MOXD1 | ENSG00000079931 | 0.210662 | -2.246997 | 2.86E-37 | 2.65E-36 |
| CAPZA3 | ENSG00000177938 | 0.210734 | -2.246505 | 1.44E-10 | 3.36E-10 |
| RGS6 | ENSG00000182732 | 0.210967 | -2.244912 | 9.60E-49 | 1.52E-47 |
| CWH43 | ENSG00000109182 | 0.211516 | -2.241164 | 9.30E-13 | 2.48E-12 |
| ZNF98 | ENSG00000197360 | 0.211536 | -2.241025 | 2.18E-28 | 1.32E-27 |
| IL1RL1 | ENSG00000115602 | 0.211647 | -2.240271 | 1.91E-28 | 1.16E-27 |
| SHBG | ENSG00000129214 | 0.212124 | -2.237020 | 2.96E-68 | 1.03E-66 |
| PPP2R2B | ENSG00000156475 | 0.212357 | -2.235436 | 2.17E-100 | 1.97E-98 |
| AQP3 | ENSG00000165272 | 0.212421 | -2.234998 | 2.53E-76 | 1.14E-74 |
| COL4A5 | ENSG00000188153 | 0.212486 | -2.234558 | 1.43E-60 | 3.58E-59 |
| SLC6A19 | ENSG00000174358 | 0.212510 | -2.234400 | 1.30E-14 | 3.86E-14 |
| PRODH2 | ENSG00000250799 | 0.212931 | -2.231539 | 4.38E-29 | 2.75E-28 |
| FOXD3 | ENSG00000187140 | 0.212941 | -2.231472 | 1.94E-16 | 6.39E-16 |
| TRPM5 | ENSG00000070985 | 0.213503 | -2.227669 | 1.08E-34 | 8.94E-34 |
| PNLIP | ENSG00000175535 | 0.213706 | -2.226303 | 4.63E-07 | 8.56E-07 |
| LRRN2 | ENSG00000170382 | 0.213959 | -2.224595 | 1.52E-29 | 9.83E-29 |
| HOXB8 | ENSG00000120068 | 0.214209 | -2.222912 | 2.35E-45 | 3.18E-44 |
| SLC26A7 | ENSG00000147606 | 0.214512 | -2.220867 | 1.25E-12 | 3.32E-12 |
| C1orf168 | ENSG00000187889 | 0.214870 | -2.218466 | 3.64E-24 | 1.78E-23 |
| SORCS1 | ENSG00000108018 | 0.215069 | -2.217131 | 1.10E-19 | 4.30E-19 |
| KIRREL2 | ENSG00000126259 | 0.215153 | -2.216566 | 3.35E-22 | 1.49E-21 |
| KCNJ12 | ENSG00000184185 | 0.215193 | -2.216294 | 8.02E-63 | 2.22E-61 |
| RAET1L | ENSG00000155918 | 0.215281 | -2.215707 | 5.45E-22 | 2.39E-21 |
| NAP1L2 | ENSG00000186462 | 0.215432 | -2.214693 | 1.41E-41 | 1.60E-40 |
| SALL3 | ENSG00000256463 | 0.215440 | -2.214640 | 1.05E-07 | 2.03E-07 |
| MT1HL1 | ENSG00000244020 | 0.215759 | -2.212505 | 6.04E-24 | 2.92E-23 |
| PRKAR2B | ENSG00000005249 | 0.216171 | -2.209752 | 1.13E-128 | 2.01E-126 |
| SVOPL | ENSG00000157703 | 0.216630 | -2.206695 | 2.38E-16 | 7.79E-16 |
| C21orf62 | ENSG00000205929 | 0.217599 | -2.200255 | 2.59E-32 | 1.91E-31 |
| LRIT3 | ENSG00000183423 | 0.217628 | -2.200065 | 1.84E-53 | 3.56E-52 |
| ACOT6 | ENSG00000205669 | 0.217835 | -2.198694 | 9.92E-40 | 1.03E-38 |
| WDR49 | ENSG00000174776 | 0.218058 | -2.197217 | 2.21E-39 | 2.25E-38 |
| C8orf4 | ENSG00000176907 | 0.218371 | -2.195145 | 2.66E-63 | 7.59E-62 |
| MAN1C1 | ENSG00000117643 | 0.218423 | -2.194805 | 1.61E-58 | 3.72E-57 |
| SSC4D | ENSG00000146700 | 0.218912 | -2.191578 | 4.93E-66 | 1.57E-64 |
| FAM83E | ENSG00000105523 | 0.219142 | -2.190064 | 8.72E-17 | 2.93E-16 |
| COL4A4 | ENSG00000081052 | 0.219289 | -2.189096 | 8.58E-81 | 4.56E-79 |
| KCNMB2 | ENSG00000197584 | 0.219378 | -2.188508 | 7.20E-29 | 4.46E-28 |
| B4GALNT3 | ENSG00000139044 | 0.219391 | -2.188424 | 1.32E-34 | 1.09E-33 |
| LPA | ENSG00000198670 | 0.219515 | -2.187606 | 3.56E-19 | 1.35E-18 |
| HTR3D | ENSG00000186090 | 0.219740 | -2.186127 | 1.04E-12 | 2.76E-12 |
| DNASE1L3 | ENSG00000163687 | 0.219826 | -2.185563 | 1.17E-52 | 2.19E-51 |
| PHYHD1 | ENSG00000175287 | 0.219989 | -2.184499 | 4.75E-41 | 5.27E-40 |
| RP11-385J1.3 | ENSG00000275163 | 0.220178 | -2.183258 | 1.59E-13 | 4.44E-13 |
| AGR2 | ENSG00000106541 | 0.220389 | -2.181877 | 1.36E-09 | 2.97E-09 |
| PCSK2 | ENSG00000125851 | 0.220521 | -2.181014 | 2.38E-18 | 8.68E-18 |
| TYRO3 | ENSG00000092445 | 0.221048 | -2.177568 | 9.89E-89 | 6.80E-87 |
| KLHL13 | ENSG00000003096 | 0.221296 | -2.175948 | 5.32E-102 | 5.04E-100 |
| SORD | ENSG00000140263 | 0.221814 | -2.172577 | 3.79E-89 | 2.62E-87 |
| SHISA3 | ENSG00000178343 | 0.221832 | -2.172460 | 6.96E-27 | 3.92E-26 |
| ST6GAL1 | ENSG00000073849 | 0.221870 | -2.172215 | 4.19E-87 | 2.71E-85 |
| SUCNR1 | ENSG00000198829 | 0.221893 | -2.172061 | 7.80E-46 | 1.08E-44 |
| HOMER1 | ENSG00000152413 | 0.222152 | -2.170378 | 3.94E-82 | 2.20E-80 |
| AFP | ENSG00000081051 | 0.222172 | -2.170248 | 1.63E-21 | 6.98E-21 |
| C10orf55 | ENSG00000222047 | 0.222704 | -2.166803 | 1.24E-65 | 3.86E-64 |
| DLX3 | ENSG00000064195 | 0.223042 | -2.164615 | 7.52E-42 | 8.65E-41 |
| TMPRSS15 | ENSG00000154646 | 0.223428 | -2.162119 | 2.86E-11 | 6.98E-11 |
| GATA5 | ENSG00000130700 | 0.223506 | -2.161616 | 1.82E-29 | 1.17E-28 |
| ADGRA1 | ENSG00000197177 | 0.223518 | -2.161534 | 1.10E-10 | 2.58E-10 |
| PTGER3 | ENSG00000050628 | 0.223608 | -2.160959 | 3.16E-32 | 2.32E-31 |
| KHDRBS2 | ENSG00000112232 | 0.223710 | -2.160300 | 2.82E-29 | 1.78E-28 |
| TINCR | ENSG00000223573 | 0.224074 | -2.157955 | 2.10E-45 | 2.85E-44 |
| RP11-451G4.2 | ENSG00000240045 | 0.224198 | -2.157154 | 4.62E-17 | 1.58E-16 |
| SYNE4 | ENSG00000181392 | 0.224213 | -2.157056 | 2.49E-30 | 1.66E-29 |
| NUPR2 | ENSG00000185290 | 0.224287 | -2.156583 | 2.17E-08 | 4.38E-08 |
| DPT | ENSG00000143196 | 0.224364 | -2.156085 | 9.64E-27 | 5.37E-26 |
| CDH9 | ENSG00000113100 | 0.224371 | -2.156042 | 3.21E-14 | 9.33E-14 |
| DIRAS1 | ENSG00000176490 | 0.224685 | -2.154027 | 5.39E-19 | 2.03E-18 |
| FAM171A1 | ENSG00000148468 | 0.224696 | -2.153954 | 1.10E-134 | 2.37E-132 |
| GDF3 | ENSG00000184344 | 0.224765 | -2.153511 | 6.94E-47 | 1.02E-45 |
| ROS1 | ENSG00000047936 | 0.224911 | -2.152573 | 3.04E-09 | 6.49E-09 |
| LSAMP | ENSG00000185565 | 0.225260 | -2.150337 | 5.32E-14 | 1.53E-13 |
| SLC6A4 | ENSG00000108576 | 0.225411 | -2.149369 | 3.23E-54 | 6.43E-53 |
| SLC7A3 | ENSG00000165349 | 0.225596 | -2.148189 | 1.82E-14 | 5.36E-14 |
| HMX3 | ENSG00000188620 | 0.225646 | -2.147868 | 1.23E-13 | 3.45E-13 |
| ACY1 | ENSG00000243989 | 0.225883 | -2.146353 | 7.44E-92 | 5.47E-90 |
| CDH3 | ENSG00000062038 | 0.225911 | -2.146172 | 1.11E-23 | 5.31E-23 |
| FOXC1 | ENSG00000054598 | 0.226095 | -2.144996 | 4.94E-119 | 7.01E-117 |
| FAM181B | ENSG00000182103 | 0.226166 | -2.144545 | 1.74E-43 | 2.15E-42 |
| SCGB2A1 | ENSG00000124939 | 0.226593 | -2.141827 | 3.58E-18 | 1.30E-17 |
| PHF21B | ENSG00000056487 | 0.226874 | -2.140039 | 1.44E-19 | 5.60E-19 |
| BMP6 | ENSG00000153162 | 0.226910 | -2.139805 | 5.96E-69 | 2.14E-67 |
| CGNL1 | ENSG00000128849 | 0.227326 | -2.137168 | 3.96E-66 | 1.27E-64 |
| SERPINA4 | ENSG00000100665 | 0.227557 | -2.135700 | 6.11E-13 | 1.65E-12 |
| HS6ST1 | ENSG00000136720 | 0.227812 | -2.134085 | 8.04E-170 | 3.65E-167 |
| PI16 | ENSG00000164530 | 0.228137 | -2.132025 | 9.18E-17 | 3.08E-16 |
| VWA3B | ENSG00000168658 | 0.228297 | -2.131017 | 1.25E-36 | 1.12E-35 |
| MRGPRF | ENSG00000172935 | 0.228440 | -2.130110 | 5.53E-40 | 5.79E-39 |
| IGSF5 | ENSG00000183067 | 0.228992 | -2.126631 | 8.39E-28 | 4.93E-27 |
| SERPINB13 | ENSG00000197641 | 0.229501 | -2.123425 | 4.27E-08 | 8.47E-08 |
| TMC1 | ENSG00000165091 | 0.230999 | -2.114041 | 4.72E-51 | 8.36E-50 |
| LGSN | ENSG00000146166 | 0.231056 | -2.113687 | 2.01E-29 | 1.29E-28 |
| CYS1 | ENSG00000205795 | 0.231461 | -2.111160 | 3.15E-53 | 6.03E-52 |
| BTG2 | ENSG00000159388 | 0.231879 | -2.108556 | 6.75E-111 | 7.71E-109 |
| NUAK2 | ENSG00000163545 | 0.231923 | -2.108280 | 2.23E-77 | 1.06E-75 |
| CLEC4M | ENSG00000104938 | 0.231979 | -2.107931 | 4.78E-21 | 2.01E-20 |
| CXCL17 | ENSG00000189377 | 0.232234 | -2.106348 | 1.19E-12 | 3.16E-12 |
| CACNA2D3 | ENSG00000157445 | 0.232246 | -2.106277 | 9.47E-90 | 6.66E-88 |
| HOXB6 | ENSG00000108511 | 0.232476 | -2.104844 | 1.55E-75 | 6.82E-74 |
| GPM6B | ENSG00000046653 | 0.234020 | -2.095294 | 3.97E-59 | 9.36E-58 |
| CHODL | ENSG00000154645 | 0.234619 | -2.091611 | 2.86E-28 | 1.72E-27 |
| NR3C2 | ENSG00000151623 | 0.235269 | -2.087617 | 1.90E-65 | 5.86E-64 |
| SFTPA1 | ENSG00000122852 | 0.235636 | -2.085370 | 6.62E-10 | 1.48E-09 |
| SCN2B | ENSG00000149575 | 0.235647 | -2.085300 | 7.83E-17 | 2.64E-16 |
| C2orf40 | ENSG00000119147 | 0.236073 | -2.082697 | 1.90E-33 | 1.48E-32 |
| PLAU | ENSG00000122861 | 0.236079 | -2.082661 | 8.74E-60 | 2.12E-58 |
| COL9A2 | ENSG00000049089 | 0.236349 | -2.081010 | 1.03E-44 | 1.34E-43 |
| ANK2 | ENSG00000145362 | 0.236396 | -2.080724 | 7.86E-41 | 8.57E-40 |
| POU2F3 | ENSG00000137709 | 0.237497 | -2.074019 | 7.56E-33 | 5.72E-32 |
| HOXB1 | ENSG00000120094 | 0.237798 | -2.072189 | 1.80E-11 | 4.46E-11 |
| DNASE1 | ENSG00000213918 | 0.237854 | -2.071853 | 6.83E-34 | 5.42E-33 |
| AXDND1 | ENSG00000162779 | 0.237857 | -2.071832 | 2.56E-41 | 2.87E-40 |
| OCLN | ENSG00000197822 | 0.237931 | -2.071385 | 2.53E-42 | 2.99E-41 |
| MT1F | ENSG00000198417 | 0.238085 | -2.070454 | 4.58E-39 | 4.59E-38 |
| LRRC19 | ENSG00000184434 | 0.238256 | -2.069417 | 4.34E-34 | 3.48E-33 |
| PRSS35 | ENSG00000146250 | 0.238361 | -2.068782 | 8.76E-35 | 7.25E-34 |
| TMC4 | ENSG00000167608 | 0.239044 | -2.064654 | 4.81E-33 | 3.66E-32 |
| BSPRY | ENSG00000119411 | 0.239053 | -2.064597 | 3.83E-32 | 2.80E-31 |
| LINC01314 | ENSG00000259417 | 0.239088 | -2.064385 | 3.01E-24 | 1.48E-23 |
| SYT1 | ENSG00000067715 | 0.239161 | -2.063945 | 1.49E-24 | 7.43E-24 |
| KIAA1210 | ENSG00000250423 | 0.239483 | -2.062007 | 1.20E-28 | 7.39E-28 |
| LY6K | ENSG00000160886 | 0.239815 | -2.060005 | 1.82E-29 | 1.17E-28 |
| ATP10B | ENSG00000118322 | 0.241377 | -2.050638 | 4.27E-24 | 2.08E-23 |
| SCIN | ENSG00000006747 | 0.241958 | -2.047170 | 8.90E-30 | 5.81E-29 |
| ACSL6 | ENSG00000164398 | 0.242001 | -2.046918 | 9.35E-38 | 8.86E-37 |
| GPR50 | ENSG00000102195 | 0.242147 | -2.046043 | 1.86E-11 | 4.60E-11 |
| C11orf16 | ENSG00000176029 | 0.242417 | -2.044438 | 6.64E-41 | 7.29E-40 |
| TFAP2C | ENSG00000087510 | 0.242559 | -2.043593 | 5.30E-15 | 1.61E-14 |
| PCDHA12 | ENSG00000251664 | 0.242797 | -2.042180 | 3.11E-22 | 1.38E-21 |
| PRDM14 | ENSG00000147596 | 0.243444 | -2.038337 | 7.30E-12 | 1.85E-11 |
| SLC22A12 | ENSG00000197891 | 0.243454 | -2.038282 | 9.32E-15 | 2.79E-14 |
| NCMAP | ENSG00000184454 | 0.243672 | -2.036989 | 1.16E-24 | 5.86E-24 |
| RDH12 | ENSG00000139988 | 0.243784 | -2.036322 | 7.50E-24 | 3.61E-23 |
| PRL | ENSG00000172179 | 0.243880 | -2.035754 | 1.58E-21 | 6.78E-21 |
| PFN3 | ENSG00000196570 | 0.244875 | -2.029882 | 2.30E-09 | 4.95E-09 |
| 10-Mar | ENSG00000173838 | 0.245178 | -2.028096 | 8.22E-16 | 2.61E-15 |
| ACADSB | ENSG00000196177 | 0.245293 | -2.027419 | 4.73E-107 | 5.09E-105 |
| ZIC3 | ENSG00000156925 | 0.245346 | -2.027110 | 2.09E-13 | 5.81E-13 |
| DCXR | ENSG00000169738 | 0.245670 | -2.025207 | 3.50E-65 | 1.07E-63 |
| SFTPC | ENSG00000168484 | 0.245760 | -2.024675 | 5.00E-09 | 1.05E-08 |
| ALDH4A1 | ENSG00000159423 | 0.246207 | -2.022054 | 3.28E-43 | 4.01E-42 |
| C12orf74 | ENSG00000214215 | 0.246259 | -2.021754 | 5.82E-41 | 6.40E-40 |
| KDF1 | ENSG00000175707 | 0.246353 | -2.021202 | 9.11E-43 | 1.09E-41 |
| MMP3 | ENSG00000149968 | 0.246510 | -2.020282 | 8.65E-10 | 1.92E-09 |
| ILDR1 | ENSG00000145103 | 0.246729 | -2.018999 | 1.74E-56 | 3.75E-55 |
| GCM1 | ENSG00000137270 | 0.247125 | -2.016689 | 6.94E-21 | 2.89E-20 |
| COL4A3 | ENSG00000169031 | 0.247392 | -2.015127 | 2.59E-53 | 4.96E-52 |
| SLC16A7 | ENSG00000118596 | 0.247709 | -2.013281 | 3.82E-28 | 2.28E-27 |
| LRRC52 | ENSG00000162763 | 0.247711 | -2.013271 | 4.86E-06 | 8.37E-06 |
| CASZ1 | ENSG00000130940 | 0.247899 | -2.012175 | 2.66E-99 | 2.36E-97 |
| ANXA3 | ENSG00000138772 | 0.248125 | -2.010859 | 1.57E-29 | 1.01E-28 |
| NTRK2 | ENSG00000148053 | 0.248268 | -2.010032 | 1.85E-34 | 1.50E-33 |
| KIF5A | ENSG00000155980 | 0.248292 | -2.009892 | 1.40E-31 | 9.96E-31 |
| ITLN2 | ENSG00000158764 | 0.249095 | -2.005231 | 1.05E-12 | 2.80E-12 |
| PRR15L | ENSG00000167183 | 0.249560 | -2.002540 | 9.52E-17 | 3.19E-16 |
