# Supplementary material for: A Computationally Constructed lncRNA-Associated Competing Triplet Network in Clear Cell Renal Cell Carcinoma
Source: Dis Markers. 2022 Nov 17;2022:8928282. doi: 10.1155/2022/8928282 (PMC9691318; doi:10.1155/2022/8928282)
Supplement: Supplementary Materials — Table S1: the list of upregulated lncRNAs in ccRCC. Table S2: the list of downregulated lncRNAs in ccRCC. Table S3: the list of upregulated mRNAs in ccRCC. Table S4: the list of downregulated mRNAs in ccRCC. Table S5: the list of upregulated miRNAs in ccRCC. Table S6: the list of downregulated miRNAs in ccRCC. Table S7: the list of top 100 dysregulated (50 upregulated and 50 downregulated) lncRNAs in consistent with Figure 1. Table S8: the list of genes coexpressed with HOTTIP in ccRCC. [file 8928282.f1.zip › 8928282.f1/Table S5 (1).docx]

Table S5. The list of up-regulated miRNAs in ccRCC.

| **Gene symbol** | **Fold Change (FC)  (T/N)** | **log_2_FC (T/N)** | ***P* value** | **FDR** | **Gene symbol** |
| --- | --- | --- | --- | --- | --- |
| hsa-mir-122 | 93.887951 | 6.552868 | 1.85E-79 | 5.67E-78 | hsa-mir-122 |
| hsa-mir-1293 | 17.856132 | 4.158348 | 1.99E-19 | 9.25E-19 | hsa-mir-1293 |
| hsa-mir-875 | 17.148395 | 4.100002 | 1.68E-14 | 5.94E-14 | hsa-mir-875 |
| hsa-mir-891a | 14.495961 | 3.857579 | 3.19E-07 | 6.81E-07 | hsa-mir-891a |
| hsa-mir-4773-1 | 14.326132 | 3.840577 | 2.39E-28 | 1.88E-27 | hsa-mir-4773-1 |
| hsa-mir-4773-2 | 13.896099 | 3.796608 | 2.56E-27 | 1.93E-26 | hsa-mir-4773-2 |
| hsa-mir-155 | 13.094059 | 3.710840 | 5.71E-72 | 1.47E-70 | hsa-mir-155 |
| hsa-mir-885 | 13.062935 | 3.707407 | 2.11E-35 | 1.99E-34 | hsa-mir-885 |
| hsa-mir-891b | 11.664838 | 3.544094 | 2.17E-05 | 3.92E-05 | hsa-mir-891b |
| hsa-mir-4652 | 10.541030 | 3.397944 | 1.54E-21 | 8.64E-21 | hsa-mir-4652 |
| hsa-mir-599 | 10.441989 | 3.384325 | 3.05E-11 | 8.94E-11 | hsa-mir-599 |
| hsa-mir-592 | 9.899389 | 3.307339 | 2.63E-41 | 3.13E-40 | hsa-mir-592 |
| hsa-mir-892a | 9.249464 | 3.209370 | 1.05E-04 | 1.76E-04 | hsa-mir-892a |
| hsa-mir-374c | 9.128400 | 3.190362 | 2.86E-14 | 1.01E-13 | hsa-mir-374c |
| hsa-mir-210 | 8.911311 | 3.155638 | 6.83E-80 | 2.23E-78 | hsa-mir-210 |
| hsa-mir-1269b | 8.452945 | 3.079454 | 1.44E-05 | 2.67E-05 | hsa-mir-1269b |
| hsa-mir-892c | 8.375256 | 3.066133 | 7.29E-05 | 1.25E-04 | hsa-mir-892c |
| hsa-mir-892b | 8.312139 | 3.055220 | 9.95E-05 | 1.68E-04 | hsa-mir-892b |
| hsa-mir-888 | 8.031018 | 3.005583 | 1.52E-04 | 2.50E-04 | hsa-mir-888 |
| hsa-mir-4784 | 7.280772 | 2.864091 | 1.00E-09 | 2.60E-09 | hsa-mir-4784 |
| hsa-mir-224 | 6.322497 | 2.660494 | 2.54E-43 | 3.65E-42 | hsa-mir-224 |
| hsa-mir-890 | 5.947991 | 2.572402 | 3.91E-04 | 6.10E-04 | hsa-mir-890 |
| hsa-mir-3941 | 5.676423 | 2.504982 | 5.26E-34 | 4.86E-33 | hsa-mir-3941 |
| hsa-mir-21 | 5.292397 | 2.403921 | 2.41E-83 | 8.44E-82 | hsa-mir-21 |
| hsa-mir-584 | 4.706489 | 2.234651 | 2.36E-68 | 5.78E-67 | hsa-mir-584 |
| hsa-mir-4772 | 4.574136 | 2.193499 | 1.14E-47 | 1.80E-46 | hsa-mir-4772 |
| hsa-mir-142 | 4.564544 | 2.190471 | 2.99E-44 | 4.57E-43 | hsa-mir-142 |
| hsa-mir-3591 | 4.547169 | 2.184968 | 1.62E-13 | 5.51E-13 | hsa-mir-3591 |
| hsa-mir-452 | 4.354413 | 2.122478 | 5.13E-43 | 7.17E-42 | hsa-mir-452 |
| hsa-mir-6509 | 4.143581 | 2.050878 | 6.10E-33 | 5.42E-32 | hsa-mir-6509 |
| hsa-mir-137 | 4.024095 | 2.008664 | 3.65E-04 | 5.76E-04 | hsa-mir-137 |
| hsa-mir-144 | 4.018587 | 2.006688 | 2.12E-20 | 1.10E-19 | hsa-mir-144 |
