# Supplementary material for: A Computationally Constructed lncRNA-Associated Competing Triplet Network in Clear Cell Renal Cell Carcinoma
Source: Dis Markers. 2022 Nov 17;2022:8928282. doi: 10.1155/2022/8928282 (PMC9691318; doi:10.1155/2022/8928282)
Supplement: Supplementary Materials — Table S1: the list of upregulated lncRNAs in ccRCC. Table S2: the list of downregulated lncRNAs in ccRCC. Table S3: the list of upregulated mRNAs in ccRCC. Table S4: the list of downregulated mRNAs in ccRCC. Table S5: the list of upregulated miRNAs in ccRCC. Table S6: the list of downregulated miRNAs in ccRCC. Table S7: the list of top 100 dysregulated (50 upregulated and 50 downregulated) lncRNAs in consistent with Figure 1. Table S8: the list of genes coexpressed with HOTTIP in ccRCC. [file 8928282.f1.zip › 8928282.f1/Table S6 (1).docx]

Table S6. The list of down-regulated miRNAs in ccRCC.

| **Gene symbol** | **Fold Change (FC)  (T/N)** | **log_2_FC (T/N)** | ***P* value** | **FDR** | **Gene symbol** |
| --- | --- | --- | --- | --- | --- |
| hsa-mir-514b | 0.015757 | -5.987854 | 8.39E-124 | 5.86E-122 | hsa-mir-514b |
| hsa-mir-934 | 0.017804 | -5.811686 | 4.28E-135 | 6.98E-133 | hsa-mir-934 |
| hsa-mir-506 | 0.020903 | -5.580175 | 9.24E-140 | 2.26E-137 | hsa-mir-506 |
| hsa-mir-514a-3 | 0.051057 | -4.291749 | 4.96E-130 | 6.07E-128 | hsa-mir-514a-3 |
| hsa-mir-508 | 0.051553 | -4.277805 | 1.11E-154 | 5.42E-152 | hsa-mir-508 |
| hsa-mir-514a-1 | 0.052022 | -4.264738 | 3.53E-126 | 3.46E-124 | hsa-mir-514a-1 |
| hsa-mir-514a-2 | 0.053807 | -4.216051 | 2.19E-124 | 1.78E-122 | hsa-mir-514a-2 |
| hsa-mir-129-1 | 0.073236 | -3.771307 | 5.79E-76 | 1.66E-74 | hsa-mir-129-1 |
| hsa-mir-129-2 | 0.083646 | -3.579558 | 7.97E-63 | 1.62E-61 | hsa-mir-129-2 |
| hsa-mir-200c | 0.109814 | -3.186868 | 6.36E-43 | 8.63E-42 | hsa-mir-200c |
| hsa-mir-509-3 | 0.110954 | -3.171967 | 1.47E-97 | 7.17E-96 | hsa-mir-509-3 |
| hsa-mir-216b | 0.124054 | -3.010960 | 6.26E-20 | 3.06E-19 | hsa-mir-216b |
| hsa-mir-509-2 | 0.125659 | -2.992413 | 3.62E-85 | 1.48E-83 | hsa-mir-509-2 |
| hsa-mir-509-1 | 0.125805 | -2.990735 | 1.83E-83 | 6.90E-82 | hsa-mir-509-1 |
| hsa-mir-184 | 0.156033 | -2.680074 | 1.45E-13 | 4.95E-13 | hsa-mir-184 |
| hsa-mir-141 | 0.171515 | -2.543592 | 4.61E-21 | 2.48E-20 | hsa-mir-141 |
| hsa-mir-362 | 0.177822 | -2.491498 | 3.86E-102 | 2.36E-100 | hsa-mir-362 |
| hsa-mir-203b | 0.192317 | -2.378444 | 3.54E-21 | 1.93E-20 | hsa-mir-203b |
| hsa-mir-138-1 | 0.221427 | -2.175100 | 1.27E-18 | 5.51E-18 | hsa-mir-138-1 |
| hsa-mir-1251 | 0.221769 | -2.172872 | 8.68E-21 | 4.57E-20 | hsa-mir-1251 |
| hsa-mir-138-2 | 0.246666 | -2.019371 | 7.30E-18 | 3.08E-17 | hsa-mir-138-2 |
