# Supplementary material for: A Computationally Constructed lncRNA-Associated Competing Triplet Network in Clear Cell Renal Cell Carcinoma
Source: Dis Markers. 2022 Nov 17;2022:8928282. doi: 10.1155/2022/8928282 (PMC9691318; doi:10.1155/2022/8928282)
Supplement: Supplementary Materials — Table S1: the list of upregulated lncRNAs in ccRCC. Table S2: the list of downregulated lncRNAs in ccRCC. Table S3: the list of upregulated mRNAs in ccRCC. Table S4: the list of downregulated mRNAs in ccRCC. Table S5: the list of upregulated miRNAs in ccRCC. Table S6: the list of downregulated miRNAs in ccRCC. Table S7: the list of top 100 dysregulated (50 upregulated and 50 downregulated) lncRNAs in consistent with Figure 1. Table S8: the list of genes coexpressed with HOTTIP in ccRCC. [file 8928282.f1.zip › 8928282.f1/Table S7 (1).docx]

Table S7. The list of top 100 dysregulated (50 up-regulated and 50 down-regulated) lncRNAs in consistent with Figure 1.

| RP11-89B16.1 |
| --- |
| RP11-714L20.1 |
| RP11-18H21.3 |
| RP4-735C1.4 |
| FAM215A |
| RP11-128L5.1 |
| LINC01555 |
| RP11-575B7.3 |
| RP11-536G4.1 |
| CTD-2007H18.1 |
| RP1-137K24.1 |
| WSPAR |
| RP11-208K4.1 |
| RP11-527L4.6 |
| RP11-527L4.2 |
| RP11-390N6.1 |
| RP11-516J2.1 |
| LINC01378 |
| RP11-850F7.7 |
| RP11-142A12.1 |
| AC068138.1 |
| RP11-61L19.2 |
| AP000696.2 |
| RP11-317M11.1 |
| RP11-195B3.1 |
| LINC01543 |
| RP11-386B13.4 |
| RP11-531H8.2 |
| LINC01571 |
| RP5-1018K9.1 |
| LINC00864 |
| RP11-752D24.2 |
| LINC01055 |
| LINC01020 |
| RP1-56K13.5 |
| RP4-655J12.4 |
| RP11-573D15.8 |
| RP11-321G12.1 |
| RP11-180C16.1 |
| RP3-466P17.1 |
| PLS3-AS1 |
| RP11-116O18.1 |
| AC003090.1 |
| FOXCUT |
| LINC01598 |
| RP11-211N11.5 |
| RP11-363J20.1 |
| OSTM1-AS1 |
| RP11-380J14.1 |
| RP6-191P20.4 |
| DGCR10 |
| GATM-AS1 |
| RP5-940J5.3 |
| RP11-121A14.2 |
| CDKN2B-AS1 |
| LINC00299 |
| DPP9-AS1 |
| RP11-196G11.2 |
| RP11-181E10.3 |
| LINC00487 |
| AC005264.2 |
| RP13-297E16.4 |
| RP11-626H12.2 |
| RP11-142A23.1 |
| RP11-123K3.9 |
| RP11-340F14.6 |
| RP11-14C10.5 |
| RP11-115N4.1 |
| RP11-547D24.1 |
| SFTA1P |
| RP11-259N19.1 |
| CTD-2026K11.6 |
| CTD-2020K17.1 |
| RP11-789C17.1 |
| AC078883.3 |
| PHKA2-AS1 |
| RP11-848P1.3 |
| AC109826.1 |
| AC133644.2 |
| L3MBTL4-AS1 |
| HIF1A-AS2 |
| RP11-155G14.6 |
| RP11-513G11.2 |
| AC074286.1 |
| RP11-999E24.3 |
| SLC25A5-AS1 |
| TTC21B-AS1 |
| LINC00887 |
| RP11-598F7.5 |
| RP11-598F7.6 |
| GAS6-AS1 |
| DGCR9 |
| SLC16A1-AS1 |
| DARS-AS1 |
| RP11-798M19.6 |
| RP5-1120P11.1 |
| CTD-2015G9.2 |
| MIR210HG |
| PVT1 |
| SNHG12 |
