# Supplementary material for: A Computationally Constructed lncRNA-Associated Competing Triplet Network in Clear Cell Renal Cell Carcinoma
Source: Dis Markers. 2022 Nov 17;2022:8928282. doi: 10.1155/2022/8928282 (PMC9691318; doi:10.1155/2022/8928282)
Supplement: Supplementary Materials — Table S1: the list of upregulated lncRNAs in ccRCC. Table S2: the list of downregulated lncRNAs in ccRCC. Table S3: the list of upregulated mRNAs in ccRCC. Table S4: the list of downregulated mRNAs in ccRCC. Table S5: the list of upregulated miRNAs in ccRCC. Table S6: the list of downregulated miRNAs in ccRCC. Table S7: the list of top 100 dysregulated (50 upregulated and 50 downregulated) lncRNAs in consistent with Figure 1. Table S8: the list of genes coexpressed with HOTTIP in ccRCC. [file 8928282.f1.zip › 8928282.f1/Table S8.docx]

Table S8. The list of genes co-expressed with HOTTIP in ccRCC.

| **Gene symbol** | **Cor** | ***P* value** |
| --- | --- | --- |
| HOXA13 | 0.624614 | 7.21E-60 |
| SERPIND1 | 0.4079853 | 4.09E-23 |
| ALDH1L2 | 0.3878432 | 7.30E-21 |
| AADAC | 0.3826109 | 2.65E-20 |
| ADAM33 | 0.3752459 | 1.56E-19 |
| OSBPL6 | 0.3726825 | 2.87E-19 |
| ASIP | 0.3650474 | 1.70E-18 |
| VTN | 0.3637689 | 2.27E-18 |
| PRSS3 | 0.3603634 | 4.93E-18 |
| GUCY2D | 0.3521813 | 3.05E-17 |
| SFXN5 | 0.3498092 | 5.12E-17 |
| GDNF | 0.343487 | 2.00E-16 |
| GATA4 | 0.3414606 | 3.07E-16 |
| GUCY2C | 0.3380028 | 6.34E-16 |
| BCL2 | -0.336461 | 8.73E-16 |
| SEC14L5 | 0.3362727 | 9.08E-16 |
| ANGPTL6 | 0.3335698 | 1.59E-15 |
| CPN1 | 0.3316202 | 2.36E-15 |
| PROX1 | 0.3304079 | 3.02E-15 |
| EDNRB | -0.329808 | 3.41E-15 |
| CCNE1 | 0.329655 | 3.52E-15 |
| MFSD2A | 0.327671 | 5.25E-15 |
| MTTP | 0.3270281 | 5.97E-15 |
| MAGEC2 | 0.3258326 | 7.59E-15 |
| ASGR1 | 0.3238425 | 1.13E-14 |
| STRIP2 | 0.3233686 | 1.24E-14 |
| RAB15 | 0.3233047 | 1.25E-14 |
| AQP1 | -0.321027 | 1.96E-14 |
| ORC6 | 0.3196852 | 2.55E-14 |
| ENPP4 | -0.319587 | 2.60E-14 |
| SSX1 | 0.319429 | 2.68E-14 |
| PSAT1 | 0.3191539 | 2.83E-14 |
| HOXA11 | 0.3188754 | 2.99E-14 |
| FOXA2 | 0.3180142 | 3.53E-14 |
| ISM2 | 0.3171765 | 4.15E-14 |
| TMPRSS6 | 0.3167141 | 4.54E-14 |
| GRIN2D | 0.3156872 | 5.53E-14 |
| CILP2 | 0.3141199 | 7.46E-14 |
| ACBD7 | 0.3134887 | 8.42E-14 |
| MAT1A | 0.3132413 | 8.82E-14 |
| IGFBPL1 | 0.3119627 | 1.12E-13 |
| PITX1 | 0.3103885 | 1.51E-13 |
| TROAP | 0.3102983 | 1.54E-13 |
| SH3GL3 | 0.3080507 | 2.35E-13 |
| ANGPTL8 | 0.3079901 | 2.37E-13 |
| MAGEA6 | 0.3070101 | 2.85E-13 |
| CENPA | 0.3056788 | 3.64E-13 |
| FBXL3 | -0.305464 | 3.79E-13 |
| SHBG | 0.3031598 | 5.79E-13 |
| PABPC4L | -0.302505 | 6.53E-13 |
| TMEM63C | 0.3022893 | 6.79E-13 |
| SLC16A12 | -0.301909 | 7.28E-13 |
| SHOX2 | 0.3018333 | 7.38E-13 |
| GCKR | 0.3010067 | 8.58E-13 |
| NEIL3 | 0.3000414 | 1.02E-12 |
